# Transferability of polygenic risk scores for metabolic and cardiovascular traits in an underrepresented population

**DOI:** 10.1038/s41525-025-00532-1

**Published:** 2025-11-21

**Authors:** Phongthana Pasookhush, Apinya Surawit, Sophida Suta, Sureeporn Pumeiam, Pichanun Mongkolsucharitkul, Bonggochpass Pinsawas, Suphawan Ophakas, Yuthana Udomphorn, Sissades Tongsima, Pongsakorn Wangkumhang, Tassathorn Poonsin, Korapat Mayurasakorn

**Affiliations:** 1 Siriraj Population Health and Nutrition Research Group, Research Department, Faculty of Medicine Siriraj Hospital, Mahidol University, Bangkok, Thailand; 2 Siriraj Center of Research Excellence for Diabetes and Obesity (SiCORE-DO), Faculty of Medicine Siriraj Hospital, Mahidol University, Bangkok, Thailand; 3 Department of Anesthesiology, Faculty of Medicine Siriraj Hospital, Mahidol University, Bangkok, Thailand; 4 National Biobank of Thailand (NBT), National Center of Genetic Engineering and Biotechnology (BIOTEC), National Science and Technology Development Agency, Pathum Thani, Thailand; 5 National Center of Genetic Engineering and Biotechnology (BIOTEC), National Science and Technology Development Agency, Pathum Thani, Thailand

**Keywords:** Clinical genetics, Public health

## Abstract

Polygenic risk scores (PRSs) are promising tools for genetic risk stratification, but their performance across ancestries remains uncertain. We evaluated 64 published PRSs for eight cardiometabolic traits in 4879 Thai individuals using imputed SNP-array data. Cross-sectional and six-year longitudinal analyses were performed to assess predictive performances. PRSs for type 2 diabetes (T2D) and lipid traits showed the strongest utility, with the best-performing LDL-C and TC scores explaining up to 9.8% and 7.8% of trait variance, respectively. The T2D PRS achieved an area under the curve (AUC) of 0.70 and consistently stratified disease risk over time. In contrast, PRSs for glycemic traits and cardiovascular disease (CVD) had weaker predictive value; notably, the best-performing CVD PRS showed an inverse association with disease risk. Reduced SNP retention and ancestry-related linkage disequilibrium differences contributed to variability. These findings highlight both the potential and current limitations of PRSs in underrepresented Southeast Asian populations.

## Introduction

Cardiometabolic disorders, including type 2 diabetes (T2D), atherogenic dyslipidemia, and cardiovascular disease (CVD), account for nearly one-third of adult mortality in Southeast Asia, and their prevalence in Thailand continues to rise despite nationwide screening and management programs^1^. Conventional risk algorithms capture only part of this burden, particularly among younger adults who have yet to accumulate clinical risk factors^2^.

Polygenic risk scores (PRSs) aggregate the small effects of common genetic variants into a single quantitative estimate of inherited risk^3^. In large biobank studies, individuals in the top 5% of a PRS distribution for T2D or coronary artery disease carried a two- to four-fold higher risk of these conditions, a magnitude comparable to that conferred by some monogenic mutations^4^. Because PRSs often explain risk independently of established clinical variables, they can improve discrimination when added to standard prediction models^5^. These observations have fueled interest in using PRSs to identify high-risk individuals early, thereby enabling timely lifestyle modifications or preventive measures.

Translation to clinical practice, however, is hampered by limited transferability across ancestries. More than 80% of participants in genome-wide association studies (GWAS) are of European descent^6,7^, and PRSs developed from these populations typically lose 40–60% of their predictive accuracy when applied to African, South Asian, or East Asian cohorts^8^. This performance loss is primarily due to inter-ancestry differences in allele frequencies, linkage disequilibrium (LD) structure, and gene–environment interactions^2,9^. Although multi-ancestry GWAS and ancestry-adjusted scoring methods have begun to narrow this gap^10^, Southeast Asian populations remain markedly underrepresented, and previous regional evaluations have been limited to cross-sectional analyses or single traits^11,12^.

To address this disparity, we systematically evaluated 64 published PRSs for eight cardiometabolic traits: T2D, CVD, triglycerides (TG), total cholesterol (TC), high-density lipoprotein cholesterol (HDL-C), low-density lipoprotein cholesterol (LDL-C), fasting blood sugar (FBS), and glycated hemoglobin (HbA1c), in a cohort of 4879 Thai adults. Using imputed single-nucleotide polymorphism (SNP)-array data, we assessed cross-sectional discrimination at baseline, six-year longitudinal prediction, and variant-level trait associations. To our knowledge, this is the first comprehensive multi-trait, longitudinal evaluation of PRS performance in a Thai population. Our findings provide a benchmark for integrating genomic risk stratification into non-communicable disease surveillance and highlight priorities for generating ancestry-matched data to optimize PRS accuracy.

## Results

### Cohort characteristics and genotype imputation

The initial dataset consisted of 4964 individuals from the SIH and SIOH studies, with 659,184 genotyped variants. After quality control, 4879 individuals and 610,509 variants remained for imputation. The genotype imputation yielded 15,408,335 imputed variants, of which 6,467,992 and 3,075,492 passed quality control and INFO score thresholds of ≥0.3 and ≥0.8, respectively.

Among the 4879 individuals, 300 (6.15%) had T2D and 54 (1.11%) had CVD. Baseline characteristics are summarized in Table 1. The majority of individuals were female, with male overrepresented among T2D and CVD cases (Table 1). Individuals with T2D or CVD were significantly older (*p*-value = 2.8 × 10^-39^), had higher BMI (*p*-value = 2.7 × 10^-30^) and WC (*p*-value = 1.1 × 10^-33^), and elevated SBP and DBP (*p*-value = 6.0 × 10^-22^ and 4.0 × 10^-9^) compared to control (Table 1). TG and HDL-C differed significantly among groups (*p*-value = 4.2 × 10^-14^ and 2.3 × 10^-11^), while TC and LDL-C did not (*p*-value = 0.616 and 0.320) (Table 1). Both FBS and HbA1c were significantly higher in the disease groups (*p*-value = 1.4 × 10^-47^ and 2.9 × 10^-61^), as were MAU/Cr ratios (*p*-value = 8.8 × 10^-24^) (Table 1). These findings suggest a greater metabolic burden and a higher risk of renal dysfunction in individuals with T2D and CVD compared to controls.

**Table 1:** Baseline characteristics of the study cohort

Characteristics		Control (*n* = 4543)	T2D (*n* = 300)	CVD (*n* = 54)	*p*-value
Sex					0.222
	male	1037 (22.83)	80 (26.67)	15 (27.78)	
	female	3506 (77.17)	220 (73.33)	39 (72.22)	
Age (year)		35.0 (29.0–41.0)	43.0 (34.8–50.0)	47.0 (42.0–53.0)	2.76 × 10^-39^
BMI (kg/m^2^)		23.2 (20.6–26.5)	27.3 (23.2–31.8)	25.5 (22.2–30.3)	2.74 × 10^-30^
WC (cm)		79.0 (72.0–87.2)	90.0 (80.0–99.0)	83.5 (76.3–98.4)	1.11 × 10^-33^
SBP (mmHg)		117 (108–127)	127 (115–138)	132 (116–140)	6.03 × 10^-22^
DBP (mmHg)		71 (65–78)	75 (68–84)	73 (69–85)	4.01 × 10^-9^
Smoking, yes		426 (9.38)	51 (17.00)	9 (16.67)	4.39 × 10^-5^
Alcohol, yes		3,206 (70.57)	220 (73.33)	34 (62.96)	0.273
**Biochemical measurements**					
FBS (mg/dL)		87 (82–92)	101 (89–127)	92.5 (86.0–103.8)	1.40 × 10^-47^
HbA1c (%)		5.4 (5.2–5.7)	6.0 (5.5–7.2)	5.7 (5.4–6.2)	2.89 × 10^-61^
TC (mg/dL)		190.0 (169.0–212.0)	189.0 (166.3–215.0)	189.0 (157.0–213.0)	0.616
TG (mg/dL)		80.0 (58.0–116.0)	105.0 (73.0–156.8)	90.0 (68.9–129.9)	4.42 × 10^-14^
HDL-C (mg/dL)		60.0 (50.0–72.0)	53.0 (44.6–62.0)	54.5 (45.7–70.1)	2.32 × 10^-11^
LDL-C (mg/dL)		108.8 (88.6–129.6)	107.4 (83.6–134.0)	101.2 (80.4–128.0)	0.320
MAU/Cr ratio		3.9 (2.6–6.6)	7.0 (3.6–19.1)	5.7 (3.5–13.5)	8.81 × 10^-24^

Data are presented as median (Q1–Q3) or number (percentage).

*BMI* body mass index, *WC* waist circumference, *FBS* fasting blood sugar, *HbA1c* hemoglobin A1c, *TC* total cholesterol, *TG* triglyceride; *HDL-C* high density lipoprotein cholesterol, *LDL-C* low density lipoprotein cholesterol, *MAU/Urine Cr ratio* microalbumin/creatinine ratio.

### Performance of PRS for cardiometabolic traits

**Fig. 1:**
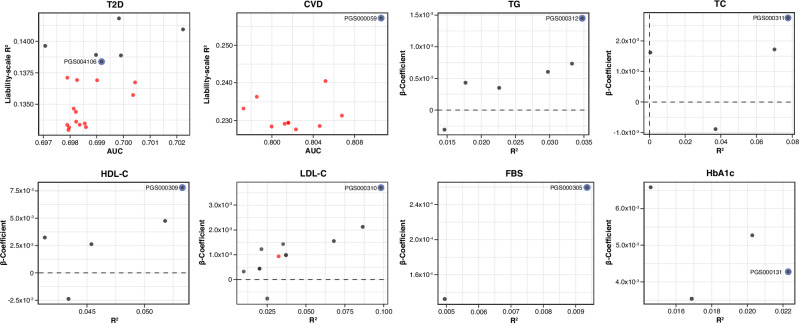
Overall performance of all evaluated PRSs for eight cardiometabolic traits. Performance metrics of all 64 PRSs evaluated across eight cardiometabolic traits: T2D, CVD, TG, TC, HDL-C, LDL-C, FBS, and HbA1c. PRS performance for binary traits is shown using AUC and liability- scale R^2^, while continuous traits are shown using R^2^ and beta coefficients. Each dot represents an individual PRS, with black dots indicating statistically significant associations (FDR < 0.05) and red dot representing non-significant associations. The best-performing PRS for each trait is highlighted with a blue outline and labeled by its PGS catalog identifier. Figures generated using R (ggplot2) and finalized in Adobe Illustrator.

The predictive performance of 64 PRS across eight cardiometabolic traits was evaluated using regression models. For binary traits (T2D and CVD), model performance was measured using OR, AUC and liability-scale R^2^. For continuous traits (TG, TC, HDL-C, LDL-C, FBS, and HbA1c), R^2^ and beta coefficients were used. Of all PRSs, 60.9% were significantly associated with their respective phenotypes at an FDR < 0.05. The predictive performance varied by traits: six of 20 T2D PRSs (30%) were significant, while the majority of glycemic and lipid PRSs passed the FDR threshold (Fig. 1 and Supplementary Table S4-S5). Only one PRS for CVD (PGS000059) met the significance threshold after FDR correction (Fig. 1 and Supplementary Table S4).

The best-performing PRSs were selected based on maximum AUC (binary) or R^2^ (continuous) and statistical significance. PGS000312 was identified as the best-performing PRS for TG (R^2^ = 0.0347; FDR = 2.6 × 10^-312^), PGS000311 for TC (R^2^ = 0.0776; FDR = 7.8 × 10^-60^), PGS000309 for HDL-C (R^2^ = 0.0533; FDR = 5.1 × 10^-85^), and PGS000310 for LDL-C (R^2^ = 0.0982; FDR = 8.7 × 10^-85^) (Table 2). For glycemic traits, PGS000305 was best for FBS (R^2^ = 0.0096; FDR = 5.0 × 10^-324^), and PGS000131 for HbA1c (R^2^ = 0.0222; FDR = 2.3 × 10^-312^), each showing the strongest association with trait levels in the cohort (Table 2). For T2D, PGS000854 had the highest AUC (AUC = 0.702[0.671–0.733]; FDR = 0.0327) (Supplementary Table S4). However, given the similar predictive performance among T2D PRSs, all significant PRSs for T2D were evaluated in further subgroup and follow-up analyses. These led to PGS004106 as the most robust PRS for T2D (AUC = 0.699[0.667–0.731]; FDR = 0.0470) (Table 2), as described in later sections. For CVD, PGS000059 was the best-performing PRS (AUC = 0.811[0.758–0.863]; FDR = 0.0356), and demonstrated an inverse association, with higher PRS values associated with reduced disease risk (OR = 0.358[0.175–0.726]) (Table 2).

**Table 2:** Summary of best performing PRS for each trait

Trait	PRS ID	SNP retention	AUC	Pseudo-R2	Liability-scale R2		OR		*p-value*		FDR
T2D	PGS004106	24/35 (68.6%)	0.699 [0.667–0.731]	0.0323	0.1383		1.586 [1.114–2.260]		0.0106		0.0470*
CVD	PGS000059	27/46 (58.7%)	0.811 [0.758–0.863]	0.0137	0.2573		0.358 [0.175–0.726]		0.0046		0.0356*
**Trait**	**PRS ID**	**SNP retention**	**β-coefficient**			**Adjusted R2**		***p-value***		**FDR**	
TG	PGS000312	136/190 (71.6%)	1.60 × 10^-3^ [1.36 × 10^-3^–1.85 × 10^-3^]			0.0347		7.85 × 10^-313^		2.59 × 10^-312^*	
TC	PGS000311	161/234 (68.8%)	2.82 × 10^-3^ [2.49 × 10^-3^–3.15 × 10^-3^]			0.0776		5.70 × 10^-60^		7.84 × 10^-60^*	
HDL-C	PGS000309	167/247 (67.6%)	9.10 × 10^-3^ [7.99 × 10^-3^–1.02 × 10^-2^]			0.0533		3.22 × 10^-85^		5.06 × 10^-85^*	
LDL-C	PGS000310	133/194 (68.6%)	3.88 × 10^-3^ [3.49 × 10^-3^–4.28 × 10^-3^]			0.0982		5.79 × 10^-85^		8.69 × 10^-85^*	
FBS	PGS000305	17/31 (54.8%)	1.63 × 10^-4^ [2.60 × 10^-4^–3.56 × 10^-4^]			0.0096		5.00 × 10^-324^		5.00 × 10^-324^*	
HbA1c	PGS000131	16/19 (84.2%)	2.40 × 10^-3^ [4.28 × 10^-3^–6.16 × 10^-3^]			0.0222		6.32 × 10^-313^		2.32 × 10^-312^*	

*FDR < 0.05 after Benjamini–Hochberg correction (two-sided).

**Fig. 2:**
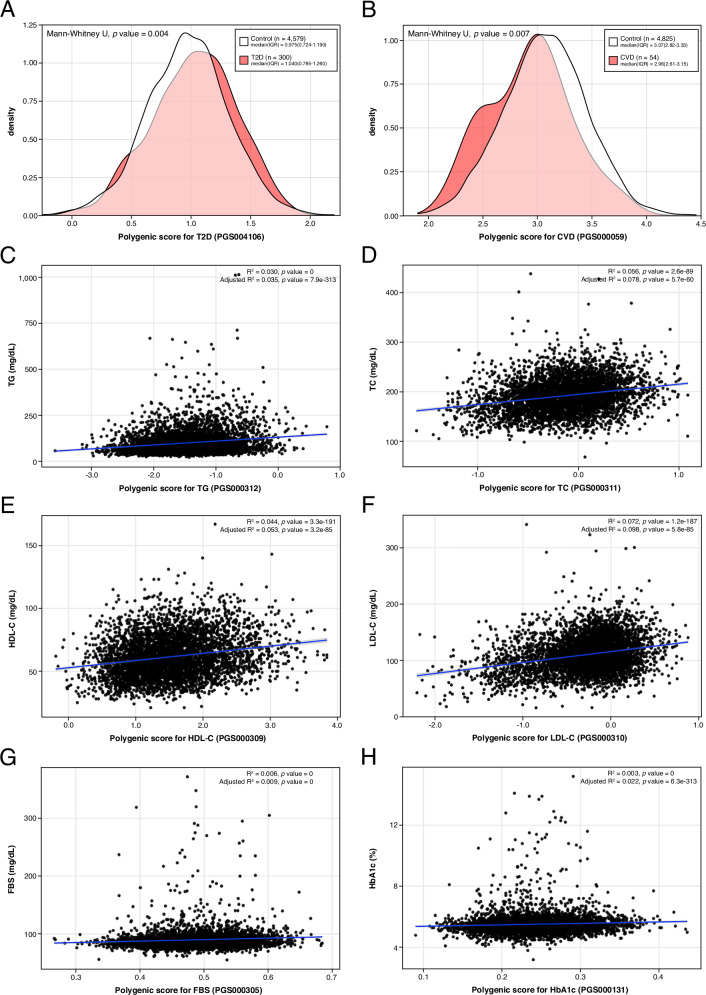
Distribution and trait association of best-performing PRSs. Distribution and phenotype correlation of the best-performing PRSs for T2D (**A**), CVD (**B**), TG (**C**), TC (**D**), HDL-C (**E**), LDL-C (**F**), FBS (**G**), and HbA1c (**H**). Panels A and B show density plots of PRS distribution in cases (red) and controls (white) for T2D and CVD. Median and IQR are provided in the top right of each plot. T2D cases exhibited significantly higher PRS values, while CVD cases showed significantly lower PRS values compared to controls. Panels C-H display scatter plots of continuous traits values against corresponding PRS values. Each plot includes a fitted regression line (blue) with 95% confidence interval (gray). Coefficient of determination (R^2^) and associated *p*-values for both unadjusted and adjusted models (adjusted for age, sex, PC1-10) are presented in the top right corner of each panel. All traits demonstrated significant positive correlation between traits and their respective PRSs. Figures generated using R (ggplot2) and finalized in Adobe Illustrator.

Density and scatter plots confirmed trait-specific associations. Density plots for binary traits (T2D and CVD) revealed significant separation between cases and controls, while scatter plots for continuous traits (TG, TC, HDL-C, LDL-C, FBS and HbA1c) showed linear associations between PRS values and measured phenotypes (Fig. 2). All best-performing PRSs were subsequently included in subgroup and longitudinal analyses to evaluate their consistency across population strata and over time.

To further investigate sources of variability in PRS performance, we compared two metrics of SNP retention: simple SNP retention, defined as the proportion of SNPs retained after imputation quality filtering, and weighted SNP retention, which accounts for the cumulative absolute effect sizes of retained SNPs. Correlation analyses across all traits revealed modest and inconsistent associations between retention metrics and predictive performance (Supplementary Table S14). For LDL-C, weighted SNP retention exhibited a stronger correlation (R^2^ = 0.279; *p*-value = 0.078), suggesting that effect size weighting may better capture the importance of SNP composition for certain traits. However, for most traits, both retention metrics showed weak and non-significant correlations, likely reflecting score heterogeneity and limited sample size.

We compared PRS performance across traits, stratified further by the ancestry of the discovery population (European, East Asian, multi-ancestry, or other). Overall, PRSs developed from multi-ancestry cohorts tended to perform comparably or better than those derived solely from European or East Asian population across most traits (Supplementary Table S15). Notably, no PRS derived from East Asian cohorts ranked as the best-performing PRS for any trait in our dataset (Supplementary Fig. S5). Nonetheless, the number of East Asian PRSs was limited, and no statistically significant differences in predictive performance were observed across ancestry groups (Supplementary Table S15).

### PRS risk stratification and subgroup analysis for cardiometabolic traits

To evaluate the predictive performance of the PRSs, we stratified binary traits by PRS quartiles and continuous traits by PRS deciles. Given the overall performance described earlier, subgroup analyses were performed on all six significant PRSs for T2D, as well as the best-performing PRSs other traits, including CVD, TC, TG, HDL-C, LDL-C, FBS, and HbA1c.

**Fig. 3:**
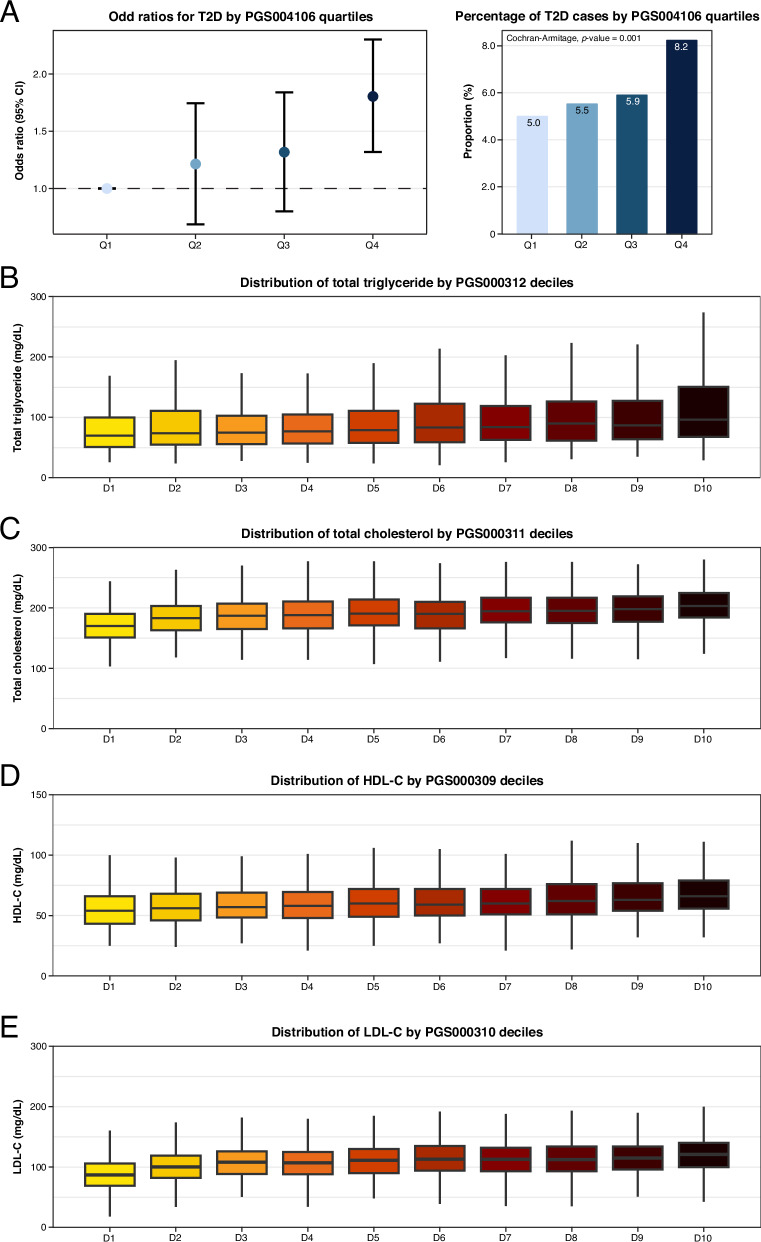
PRS-based risk stratification across polygenic score subgroups. Risk stratification across polygenic score quartiles (binary traits) and deciles (continuous traits) for the best-performing PRS. **A** The left panel shows odds ratios and 95% confidence interval for T2D risk cross PRS quartiles (PGS004106), adjusted for age, sex and PC1-10. The right panel displays the proportion of T2D cases in each PRS quartile. The highest PRS quartile showed significantly increased odds of being T2D and had the highest proportion of T2D cases, while the lower quartiles demonstrated lower odds and proportions. **B–E** Boxplots illustrate distribution of lipid levels across their corresponding PRS deciles (TG with PGS000312, TC with PGS000311, HDL-C with PGS000309 and LDL-C with PGS000310). A consistent positive trend in lipid levels was observed with increasing PRS deciles, demonstrating the utility of PRS in phenotypic stratification. Figures generated using R (ggplot2) and finalized in Adobe Illustrator.

For PRS004106, individuals in higher PRS quartiles exhibited a progressive increase in T2D risk (Fig. 3A). Individuals in the 4th quartile had significantly higher odds of developing T2D, compared to the 1st quartile (OR = 1.75[1.25–2.46]; *p*-value = 0.0013) (Fig. 3A and Supplementary Table S6). Although individuals in the 2nd and 3rd quartiles also had increased odds of T2D, these associations did not reach statistical significance (OR = 1.16[0.81–1.67]; *p*-value = 0.4267 and OR = 1.25[0.87–1.79]; *p*-value = 0.2304, respectively) (Fig. 3A and Supplementary Table S6). In addition, the proportion of T2D cases increased across quartiles (5.0, 5.5, 5.9, and 8.2% for 1st, 2nd, 3rd, and 4th quartiles, respectively; Cochran-Armitage *p*-value = 0.0011), supporting the association between higher PRS and increased T2D risk (Fig. 3A and Supplementary Table S6). Apart from PRS004106, four other PRSs for T2D (PRS000031, PRS000032l, PRS000854, and PRS004225) showed similar risk stratification patterns (Supplementary Table S6). However, further follow-up analyses were conducted to identify the most robust PRS for T2D, which will be discussed in the subsequent section.

For lipid traits, PRS deciles were strongly associated with corresponding lipid levels, demonstrating a linear trend across increasing deciles. Specifically, higher PRS deciles were linked to elevated TG levels for PGS000312 (Fig. 3B), increased TC levels for PGS000311 (Fig. 3C), higher HDL-C levels for PGS000309 (Fig. 3D), and higher LDL-C levels for PGS000310 (Fig. 3E). These results suggest that PRS deciles effectively stratify lipid levels, supporting their potential use for risk stratification.

In contrast, PRSs for FBS and HbA1c exhibited weak and inconsistent trends, limiting their predictive ability in this cohort (Supplementary Fig. S2 and Supplementary Table S6). However, for CVD, PGS000059 demonstrated a statistically significant and directional association. Individuals in higher PRS quartiles had progressively lower odds for developing CVD, with the 4^th^ quartile exhibiting the strongest protective effect, compared to the 1st quartile (OR = 0.40[0.16–0.90]; *p*-value = 0.0326). Individuals in the 2nd and 3rd quartiles also showed a protective trend, although the associations were not statistically significant (OR = 0.89[0.44–1.77]; *p*-value = 0.7390 and OR = 0.60[0.27–1.27]; *p*-value = 0.1893, respectively) (Supplementary Fig. S2 and Supplementary Table S6).

### Follow-up analyses of PRS predictive power over time

**Fig. 4:**
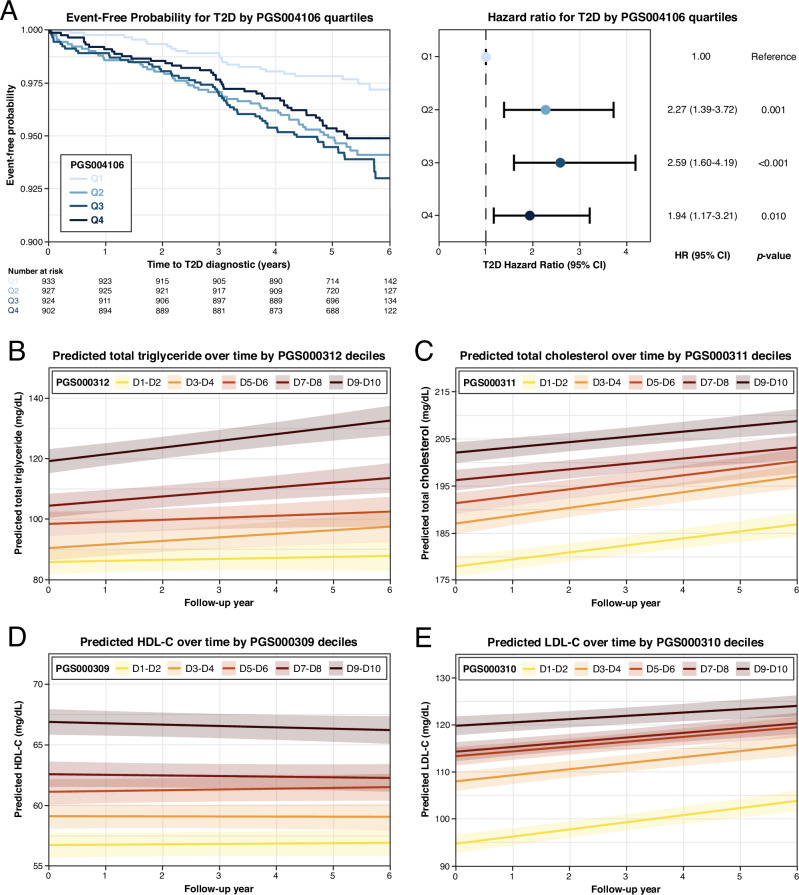
Survival and longitudinal analyses of cardiometabolic traits by polygenic score subgroup. **A** Kaplan-Meier survival curves of event-free probability (left) and adjusted hazard ratios with 95% confidence interval (right) from Cox proportional hazards models for incident T2D by PGS004106 quartiles. The analyses were adjusted for age, sex, and PC1-10. Individuals in higher PRS quartiles (Q2-Q4) exhibited significantly increased risk of developing T2D over time, compared to the lowest quartile. **B–E** Longitudinal prediction of lipid levels using LMM assessments across PRS quintiles. Predicted mean values and 95% confidence are shown for **B** TG (PGS000312), **C** TC (PGS000311), **D** HDL-C (PGS000309) and **E** LDL-C (PGS000310) over a six-year period. Higher PRS quintiles are consistently associated with elevated predicted lipid values, supporting the temporal stability and predictive utility of these scores. Figures generated using R (ggplot2) and finalized in Adobe Illustrator.

To further validate the predictive utility of PRSs, we conducted Kaplan-Meier survival analysis for binary traits and LMM assessments for continuous traits. Kaplan-Meier and Cox models showed that individuals in the 2nd, 3rd, and 4th quartiles had significantly higher risks of developing T2D over time compared to those in the 1st quartile (hazard ratios (HRs) for 1st, 2nd, 3rd, and 4th quartile: 2.27[1.38–3.71]; *p*-value = 0.0015, 2.59[1.63–4.26]]; *p*-value = 7.9 × 10^-5^, 1.94[1.16–3.19]; *p*-value = 0.0112) (Fig. 4A and Supplementary Table S8). Although the HRs did not follow a strictly increasing trend across quartiles—with the 4th quartile showing a slightly lower HR than the 3rd and 2nd—each higher quartile was still associated with a significantly elevated risk compared with the 1st quartile. This suggests that individuals with higher PRS values are consistently at greater risk of developing T2D, even if the risk increment is not linear across all quartiles. These findings support the utility of PRS004106 as the strongest T2D predictor in this cohort. In contrast, the other four tested PRSs for T2D demonstrated inconsistent or weaker associations with T2D risk (Supplementary Table S8).

For continuous traits, LMM confirmed the predictive utility of lipid trait PRSs over six years (Fig. 4B–E and Supplementary Table S9). Higher PRS consistently predicted higher lipid levels over time. Specifically, PGS000312 for TG showed a clear gradient of increasing predicted TG levels from the lowest to highest PRS quintiles (Fig. 4B). Similarly, PGS000311 for TC (Fig. 4C), PGS000309 for HDL-C (Fig. 4D), and PGS000310 for LDL-C (Fig. 4E) each showed well-separated trajectory across quintiles, confirming robust stratification and temporal stability of these PRSs in predicting lipid-related traits.

In contrast to T2D and lipid traits, follow-up analyses for CVD, FBS, and HbA1c revealed limited predictive utility (Supplementary Fig. S3 and Table S8-S9). CVD PRS (PGS000059) showed no longitudinal association in survival models (*p*-value = 0.298, 0.706, and 0.130; Supplementary Fig. S3 and Table S8). For glycemic traits, PGS000305 for FBS and PGS000131 for HbA1c displayed modest upward trends over time, but lacked stratification across PRS quintiles (Supplementary Fig. S3 and Table S9).

### Cohort-specific evaluation of polygenic scores and genome-wide associations

To assess trait relevance, we tested individual SNPs of each best-performing PRS. Several well-established lipid loci showed significant associations with their respective traits after FDR correction (Supplementary Table S11). These included the APOE locus (associated with all four lipid traits), ABCA1 locus (TC, HDL-C, and LDL-C), CETP locus (TC and HDL-C), and APOB locus (TC and LDL-C). These loci are consistent with known biology and support the PRS construction (Supplementary Table S11). In contrast, common glycemic and CVD variants, including rs7903146 (*TCF7L2)*, rs9939609 (*FTO)*, and rs7136259 (*ATP2B1*) did not reach FDR significance (Supplementary Table S11). Although PRS models for FBS, and HbA1c were not predictive in this cohort, some variants in *CDKAL1*, *PLUT*, *CDKN2B-AS1;DMRTA1* were associated with both FBS and HbA1c, suggesting measurable effects on trait variation (Supplementary Table S11).

GWAS was conducted in 4607 unrelated individuals to further explore the genetic architecture of cardiometabolic traits in the cohort. GWAS revealed prominent signals at known loci for T2D (*TMEM18* and *LINC00971*), CVD (*ARHGAP22, MYO1B*, *LINC02056*, and *MOCS1)*, lipid traits (GCKR, APOA5-APOC3 loci for TG, and *APOE*, *CETP*, *LDLR* for LDL-C, HDL-C, and TC), and suggestive signals for FBS and HbA1c (*TSPAN2*, *SPAG17*, *CASR*, and *FTO*) (Supplementary Fig. S4 and Table S12). No novel significant loci were identified.

## Discussion

This study systematically evaluated the performance of 64 publicly available PRSs for eight cardiometabolic traits in a Thai cohort, leveraging both baseline and longitudinal data. While the majority of PRSs showed significant associations with their respective traits, predictive performance varied considerably. The best-performing PRSs for T2D and lipid traits consistently demonstrated robust associations with their corresponding phenotypes both at baseline and during follow-up. In contrast, the best-performing PRSs for CVD and glycemic traits exhibited limited predictive utility, and the leading CVD PRS was directionally protective. Variant-level analyses further reinforced the biological relevance of prioritized variants, particularly to lipid traits. Nonetheless, some variants showed significant associations with glycemic traits, indicating modest genetic effects. These findings highlight both the potential and the current limitations of PRS implementation in underrepresented populations.

One of the key findings of this study is the variable performance of PRS across traits, with PRSs for T2D and lipid traits demonstrating stronger predictive utility than those for CVD and glycemic traits. This pattern is consistent with previous reports, where PRSs derived from large-scale GWAS, especially for T2D and lipid traits, tend to perform better across diverse ancestries, as shown by both internal and external validations (e.g., refs. ^13–17^, (Supplementary Table S3). In contrast, PRSs for CVD and glycemic traits underperformed. This likely reflects phenotypic heterogeneity, smaller GWAS sample sizes, and the multifactorial nature of these outcomes. These findings emphasize the importance of trait-specific and ancestry-related factors in determining PRS transferability.

SNP retention emerged as a tangible constraint on PRS performance. As our genotype data were derived from array-based imputation, we limited analysis to PRSs retaining ≥50% of their variants at INFO ≥ 0.8–a compromise that balances coverage with imputation reliability. Nevertheless, retention rates varied substantially across scores, ranging from 50.5 to 100% depending on trait and the design of the original PRS (Supplementary Table S4 and S5). Although high retention did not guarantee stronger performance, lower retention often coincided with poor predictive utility, especially for FBS and CVD, both of which exhibited low SNP retention and limited phenotypic associations. Reduced retention likely compromises score fidelity by excluding informative loci, therefore attenuating true effect sizes^18^. Nonetheless, a previous study has shown that poorly imputed SNPs can shift an individual’s PRS ranking by an entire decile^19^. Additionally, high imputation accuracy (INFO ≥ 0.8) is recommended for polygenic score analysis to ensure reliable effect estimation and score transferability across populations^3^. Although many modern PRS methods, including PRS-CS and LDpred2 rely on HapMap3 SNPs as their LD scaffold, only approximately 13.3% of these variants were available in our imputed dataset (INFO ≥ 0.8), therefore limiting their applicability in our setting. This limited coverage likely stems from differences in genotyping array, LD patterns, and allele frequency distributions in East Asian populations. To enhance imputation accuracy and haplotype resolution, we employed a custom reference panel from the GAsP project, which was specifically optimized for Asian ancestries. While this strategy provided improved performance for our Thai cohort, it may reduce compatibility with HapMap3-based PRSs or LD-informed Bayesian methods. These highlight the technical challenge of applying published PRSs to populations with different genotyping platforms and imputation reference panels.

Further analyses examining the correlation between simple and weighted SNP retention rates and PRS performance revealed only modest and inconsistent associations across traits (Supplementary Table S14). Notably, weighted retention did not consistently outperform simple retention in explaining performance variability. These findings suggest that the loss of high-effect SNPs is not the primary driver of PRS underperformance in this cohort. Instead, the limited transferability likely stems from ancestry mismatches between the discovery and target populations, reinforcing the importance of ancestry-matched PRS development.

Genetic ancestry discrepancies further impacted PRS performance. LD structure and MAF differences between East Asian and the predominantly European GWAS discovery cohorts reduce PRS transferability and predictive accuracy. A previous study has shown that differences in LD and MAF across populations can explain up to 70–80% of the relative accuracy loss in PRSs for cardiometabolic traits^9^. These effects are most apparent for glycemic traits, where LD differences may prevent tagging of causal variants—i.e., proxy SNPs in European-derived PRSs fail to capture the true causal variants in East Asian genomes, resulting in attenuated effect estimates. Population-specific allele frequencies support this issue: for instance, the well-known *TCF7L2* variant rs7903146 has a MAF of only 0.02 in the Thai population, compared to 0.32 in Europeans, diminishing its predictive contribution^20^. These observations emphasize the importance of ancestry-matched LD reference panels and population-specific GWAS to improve the precision and generalizability of PRS in underrepresented populations.

The performance of PRS stratified by the ancestry composition of the discovery cohorts demonstrated equal or better overall performance for multi-ancestry derived PRSs compared to those from single-ancestry cohorts. Interestingly, there was no East Asian derived PRS ranked as the best-performing PRS for any trait. This likely reflects a combination of factors, including limited availability of East Asian PRSs, smaller discovery sample sizes, and differences in score construction methods. However, given the limited number of PRSs available in each ancestry group, we caution against overinterpreting these findings. Expanding ancestry-balanced PRS resources will be essential for robust assessments of predictive performance across global populations.

The most consistent and clinically promising results emerged from the lipid trait PRSs. The best-performing PRSs explained between 3.5 and 9.8% of the variance in lipid levels at baseline and maintained similar effect sizes in longitudinal mixed-effects models (Table 2 and Supplementary Table S9). These results are consistent with previous multi-ancestry studies, where lipid PRSs have demonstrated relatively stable performance across European, Asian, and African populations^21,22^. Variant-level analyses reinforced these findings: canonical loci such as *APOE*, *ABCA1*, *CETP*, and *APOB* displayed directionally consistent associations with lipid traits in this cohort, suggesting shared biological mechanisms despite differences in genetic background (Supplementary Table S11 and S12). Clinically, lipid PRSs could enable early risk stratification. For example, LDL-C PRS stratification in our cohort produced differences of approximately 20 mg/dL between the highest and lowest PRS deciles (Fig. 4E). Additionally, in European datasets, individuals in the top 5% of LDL-C PRS distributions show elevated LDL-C levels comparable to those caused by pathogenic *LDLR* variants^23,24^. This PRS stratification could support earlier initiation of lipid-lowering therapy or risk screening strategies.

The T2D PRS performed favorably in our cohort, achieving an AUC of approximately 0.70–exceeding the multi-ancestry benchmark reported in the original discovery study (AUC 0.56–0.58, Supplementary Table S3). This result is consistent with prior East-Asian studies: a PRS based on meta-analyses of Japanese and Korean GWAS reported an AUC of 0.68 in an independent Korean cohort^11^, while a score trained exclusively on Korean datasets achieved an AUC of 0.74 after external validation^12^. Differences in PRS performance across studies likely reflects ancestry matching between the discovery and target populations, cohort design (e.g., incident vs. prevalent cases), and PRS construction methods (e.g., genome-wide vs. clumping-and-thresholding)^25^. Nevertheless, our analyses showed that individuals in the highest T2D PRS quartile had a significantly elevated risk of developing T2D—both cross-sectionally and in time-to-event models (Supplementary Table S6 and S8), highlighting its potential clinical utility for screening and risk stratification in this population. Although the HRs for incident T2D did not increase monotonically across quartiles, all non-reference groups showed significantly higher risk than the lowest quartile. This modest variation may reflect stochastic variation, environmental influences, or changes in risk profiles over the extended follow-up period, rather than limitations of the PRS itself^26,27^.

Unlike lipid traits and T2D, the PRSs for CVD and glycemic traits demonstrated limited predictive performance (Supplementary Fig. S2 and S3). Despite statistical associations with their respective traits, the effect sizes were small and stratification patterns across PRS subgroups were less pronounced compared to those for lipid traits and T2D. This reduced performance is likely due to the complex polygenic architecture of the traits and substantial influence of environmental or behavioral factors^8^. Notably, the best-performing CVD PRS (PGS000059) exhibited a directionally protective association, with higher PRS values linked to lower odds of disease. We ruled out common technical artifacts, including strand alignment errors and palindromic SNP misclassification, based on variant matching diagnostics from scoring file logs (Supplementary Table S13). More plausible explanations include the loss of key risk loci during imputation or population-specific LD structures that may have distorted the aggregate effect^9^. An important contextual factor is the sex composition of our cohort, with over 70% of participants and most CVD cases being female. While we initially considered sex-stratified analysis, the number of CVD events was too small to support reliable subgroup modeling without risking unstable estimates and inflated variance—particularly among male participants, who made up less than 30% of the cohort. Nonetheless, the observed protective direction is consistent with findings from the original PRS study, which reported that the score predicted increased coronary heart disease (CHD) risk in men but not in women. In fact, women with higher genetic risk scores exhibited lower CHD incidence and HRs^28^. These results suggest that sex-specific LD patterns, hormonal influences, or differential risk-factor exposures may modulate the net effect of CVD PRSs^29^. While our female-dominant sample likely provided sufficient power to detect a large protective association, further validation in sex-stratified or sex-balanced cohorts is needed to determine whether sex-specific reweighting is required before any clinical use of CVD PRSs in the population.

This study has several strengths. It is among the first to systematically evaluate multiple cardiometabolic PRSs in a Thai population. By incorporating both cross-sectional and longitudinal data, we were able to assess not only baseline associations but also the temporal consistency of PRS-based risk stratification. A key practical strength is the reliance on SNP array data, which is the genotyping strategy commonly used in large-scale non-communicable disease cohorts. While whole-genome sequencing provides richer variant information, it remains financially inaccessible for many population studies. Our findings demonstrate that imputed SNP data can yield meaningful PRS associations and stable longitudinal predictions, reinforcing the feasibility of integrating PRS into existing NCD surveillance frameworks, especially in resource-limited settings. However, several limitations should be noted. Although imputation quality was high, array-based genotyping restricts coverage of rare or population-specific variants. The relatively small number of incident CVD events also constrained statistical power for that outcome. In addition, all PRSs were applied in their original published form without ancestry-specific retraining and with partial SNP retention, which probably underestimates their optimal performance but mirrors the way clinicians would initially deploy published PRSs. Despite these limitations, our study provides a practical framework for evaluating and implementing PRSs in diverse, underrepresented populations using accessible genomic resources.

In conclusion, our findings highlight both the potential and current limitations of applying publicly available PRSs in an underrepresented Thai population. PRSs for lipid traits and T2D demonstrated clinically meaningful associations and longitudinal stability. In contrast, scores for CVD and glycemic traits exhibited poorer transferability, underscoring the critical need for ancestry-matched data and refined methodological approaches. Importantly, the study also demonstrated that imputed SNP-array data can support effective PRS analysis, making population-scale implementation feasible in resource-limited settings. Future efforts should focus on generating large East Asian GWAS datasets, improving imputation reference panels, and optimizing PRS construction methods to enhance accuracy across diverse populations.

## Methods

### Study cohort and phenotyping

This study used secondary data from two cohorts. The Siriraj Health (SIH) study, initiated in 2016, enrolled hospital-affiliated staff, including healthcare workers, researchers, and administrative personnel^30^.The Siriraj One Health (SIOH) study, launched in 2019 and expanded in 2023-2024, builds upon SIH under a One Health framework. SiOH included both hospital staff and community members from surrounding urban areas^31^. Baseline and follow-up clinical and genetic data were collected as part of each cohort’s routine assessments and longitudinal designs. All procedures adhered to the principles of the Declaration of Helsinki and were approved by the Institutional Review Board of the Faculty of Medicine Siriraj Hospital, Mahidol University (COA no. Si 647/2016 for the SIH cohort, and COA no. Si 381/2023 and Si 631/2019 for the SIOH cohort). Written informed consent was obtained from all participants at enrollment in the original studies.

Across both cohorts, genotype data were available for 4964 individuals, generated using the Infinium Asian Screening Array (ASA) v1.0 (Illumina, USA). Genotype quality control (QC) and imputation followed previously described protocols^31^. Briefly, QC steps included exclusion of individuals with sex discrepancies, variants with call rate <90%, individuals with call rate <97%, and variants with Hardy-Weinberg equilibrium (HWE) *p*-value < 10^-6^ using PLINK v1.9^32^. Remaining variants were strand-corrected to the GRCh38 reference genome using bcftools v1.20^33^. Haplotype phasing and genotype imputation were performed using SHAPEIT5^34^ and IMPUTE5^35^, respectively, with a custom reference panel of 15.4 million variants from 1163 individuals derived from the GenomeAsia Pilot (GAsP) project^36^. Post-imputation filtering excluded variants with minor allele frequency (MAF) < 1% or HWE *p*-value < 10^-6^. For downstream analyses, two imputation quality score (INFO) thresholds ( ≥0.3 and ≥0.8) were evaluated, with INFO ≥ 0.8 ultimately selected for PRS analyses. Genetic ancestry of the cohort was previously confirmed using principal component analysis (PCA), incorporating reference populations from the Human Genome Diversity Project (HGDP) and the 1000 Genome Project. The analysis showed that 4858 individuals (99.57%) cluster with East Asian populations, while 21 individuals (0.43%) clustered with Central/South Asian populations, supporting the genetic homogeneity of the Thai Cohort^31^.

Phenotype data included baseline clinical data and laboratory measurements, including T2D and CVD status, smoking and alcohol consumption history, body mass index (BMI), waist circumference (WC), blood pressure (BP), TG, TC, HDL-C, LDL-C, FBS, HbA1c, and microalbumin-to-creatinine ratio (MAU/Cr). T2D was defined by ICD-10 codes E10-E14, HbA1c ≥ 6.5%, or use of glucose-lowering medications, consistent with American Diabetes Association criteria^37^. CVD included coronary artery disease, myocardial infarction, ischemic heart diseases (I20–I25), peripheral artery disease, and stroke (I60–I64). All laboratory measurements were performed in an accredited clinical laboratory at Siriraj Hospital. For individuals on lipid-lowering medications, TC and LDL-C values were adjusted to account for expected pharmacologic reductions by dividing the measured values by 0.8 and 0.7, respectively^38,39^.

Longitudinal follow-up data, including new T2D and CVD diagnoses and repeated trait measurements (TG, TC, HDL-C, LDL-C, FBS, and HbA1c), were obtained from Siriraj Hospital’s electronic medical records over six years. All individuals provided written informed consent. The study was approved by the Institutional Review Board of the Faculty of Medicine Siriraj Hospital, Mahidol University (COA no. Si 647/2016 for the SIH; COA no. Si 804/2024, Si 381/2023 and Si 631/2019 for the SIOH) and conducted in accordance with the Declaration of Helsinki.

### Polygenic score selection and filtering

PRSs for eight cardiometabolic traits were retrieved from the Polygenic Score (PGS) Catalog (accessed November 13, 2024)^18^. Cardiometabolic traits included T2D (MONDO_0005148), CVD (EFO_0001645, EFO_0000319, EFO_0000612, EFO_0000712, EFO_0003144, EFO_0003763, EFO_0003777, EFO_0003875, EFO_0004264, EFO_0008583, EFO_0008586, EFO_0010820, EFO_1001375, HP_0002140, MONDO_0021661), TG (EFO_0004530), TC (EFO_0004574), HDL-C (EFO_0004612), LDL-C (EFO_0004611), FBS (EFO_0008036, OBA_VT0000188), and HbA1c (EFO_0004541). A total of 724 PRSs across these traits were identified (Supplementary Table S2). For each PRS, SNPs were aligned to the imputed genotype data using genomic coordinates (GRCh38), rsIDs, and reported effect alleles. To ensure predictive accuracy while preserving the original PRS composition, only PRS with ≥50% SNP retention after applying the imputation quality filter of INFO ≥ 0.8 were selected for further evaluation. This filtering step yielded 64 PRSs eligible for downstream analyses, including 20 for T2D, 11 for CVD, 6 for TG, 4 for TC, 5 for HDL-C, 12 for LDL-C, 2 for FBS, and 4 for HbA1c (Supplementary Table S3).

To assess the impact of imputation threshold on PRS predictive performance, two imputation quality thresholds (INFO ≥ 0.3 and INFO ≥ 0.8) were compared. For each individual, PRS values were computed by summing the count of effect alleles weighted by published effect sizes. PRS performance metrics (area under the curve (AUC) for binary traits and R^2^ for continuous traits) were similar across thresholds, indicating that using fewer SNP at a higher imputation threshold did not compromise predictive accuracy (Supplementary Fig. 1). Based on this finding, an imputation quality threshold of INFO ≥ 0.8 was used for all subsequent analyses.

### Statistical analysis

Statistical analyses were conducted using R v4.4.2. Visualizations were generated using ggplot2 R package and Adobe Illustrator v29.1 (Adobe, USA). Continuous variables were summarized as median (interquartile range, IQR); categorical variables as counts (percentages). Differences among controls, T2D, and CVD groups were assessed using the Chi-squared test (categorical) and the Kruskal-Wallis with post-hoc pairwise Wilcoxon test (continuous).

For binary traits (T2D and CVD), PRSs were evaluated using logistic regression models, with results reported as odds ratios (ORs) with 95% confidence intervals and corresponding *p*-values. Predictive performance for binary traits was further assessed using the AUC and coefficient of determination on the liability scale (liability-scale R^2^). The liability- scale R^2^ was derived from Nagelkerke’s pseudo-R^2^ using the transformation proposed by Lee et al.^40^, assuming disease prevalence of 10.2% for T2D^1^ and 5.7% for CVD^41^. For continuous traits (TG, TC, HDL-C, LDL-C, FBS, and HbA1c), linear regression was used to assess PRS associations. Model output included beta coefficients, R^2^ values (coefficient of determination), and *p*-values. For trait stratification, PRS quartiles were used for binary outcomes, whereas deciles were initially used for continuous traits to better capture gradient effects. However, for visualization in longitudinal models, PRS quintiles were used to reduce graphical clutter and facilitate interpretability. All regression models were adjusted for age, sex, and the first ten principal components (PCs) derived from genotype data. To address multiple testing, we applied Benjamini-Hochberg false discovery rate (FDR) correction separately for each trait across all tested PRSs. Association with FDR-adjusted *p*-value < 0.05 was considered statistically significant.

For longitudinal analyses, time-to-event outcomes for incident T2D and CVD were assessed using Cox proportional hazards models. PRS quartiles were modeled as categorical variables, with the lowest quartile as a reference, adjusted for age, sex and PC1-10. For continuous traits, linear mixed-effects models (LMMs) were fitted using the lme4 R package to evaluate longitudinal trajectories. Each model included fixed effects for PRS quintiles, follow-up time (year), and their interaction, with random intercepts and slopes for follow-up time at the individual level. All models included the same covariates (age, sex, and PC1-10).

### Bioinformatics analysis

GWAS were performed for each cardiometabolic trait using PLINK v2.0^42^. Related individuals were identified via the KING robust algorithm^43^. One individual from each pair with a kinship coefficient >0.086 (second-degree relatives or closer) was excluded, resulting in a dataset of 4607 unrelated individuals. GWAS models adjusted for age, sex, and PC1-10. Genome-wide significance was defined as *p*-value < 5 × 10⁻⁸; suggestive significance as *p*-value < 1 × 10⁻⁵.

To evaluate the biological relevance of the top PRSs, single SNP associations were tested for all variants included in the best-performing PRS for each trait. These association tests were conducted using the same regression models described above (logistic or linear), adjusting for age, sex, and PC1-10. To correct for multiple testing across SNPs, Benjamini-Hochberg FDR correction was applied separately for each trait. FDR-adjusted *p*-value < 0.05 was considered statistically significant.

## Supplementary information

Supplementary information

## Data Availability

All processed data and scripts used for analyses in this study are available upon reasonable request from the corresponding author.
