# Supplementary material for: Transferability of polygenic risk scores for metabolic and cardiovascular traits in an underrepresented population
Source: NPJ Genom Med. 2025 Nov 21;10:76. doi: 10.1038/s41525-025-00532-1 (PMC12638805; doi:10.1038/s41525-025-00532-1)
Supplement: Supplementary file 1 — Supplementary information [file 41525_2025_532_MOESM1_ESM.pdf]

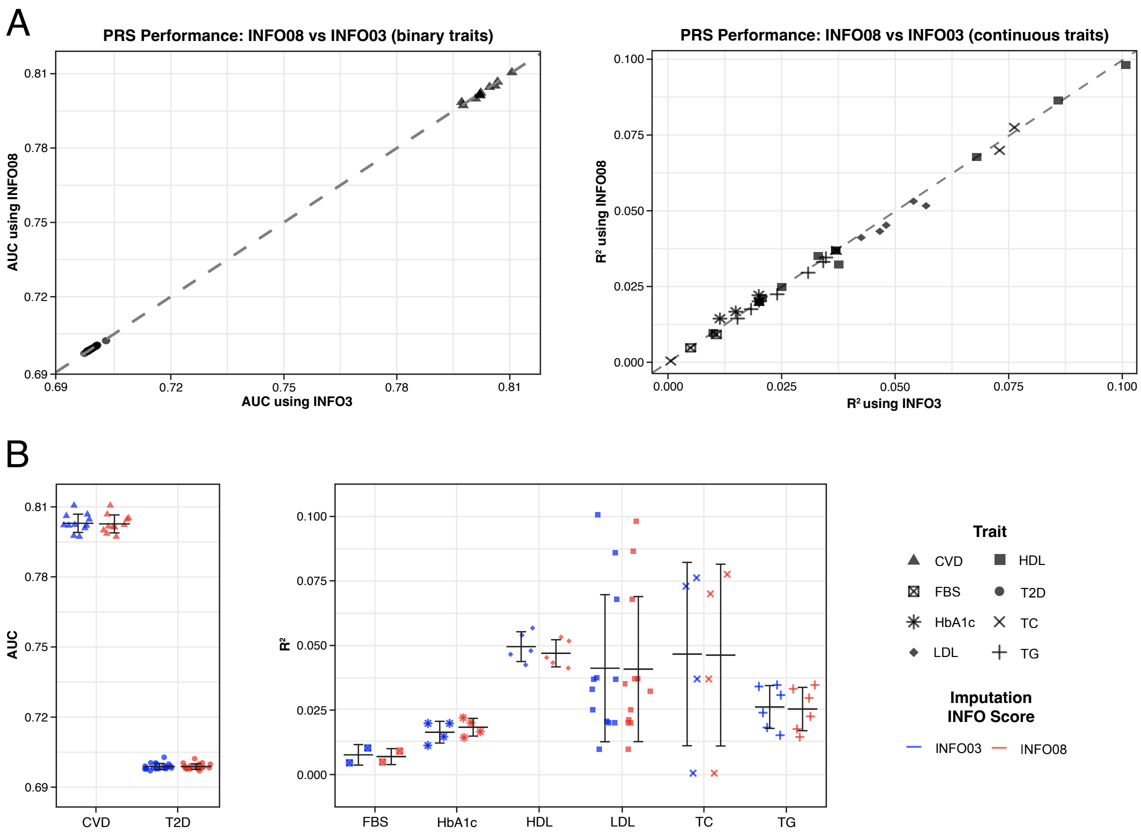

Figure S1. Performance comparison of PRSs for cardiometabolic traits using two imputation quality thresholds. Performance comparison of PRSs constructed using two imputation thresholds, including INFO ≥0.3 and INFO ≥0.8). (A) Performance metrics are shown as AUC (for binary traits, T2D and CVD) and R^2 (for continuous traits, TG, TC, HDL-C, LDL-C, FBS, and HbA1c) under each imputation threshold. Each point represents a PRS metric under both imputation thresholds. The dashed line indicates the identity line, where performances under both thresholds is equal. (B) PRS performance distributions stratified by imputation thresholds, with INFO ≥0.3 shown in blue and INFO ≥0.8 in red. Despite using fewer SNPs, the higher threshold (INFO ≥0.8) retained comparable predictive accuracy, justifying its use for further analyses. Figures generated using R (ggplot2) and finalized in Adobe Illustrator.

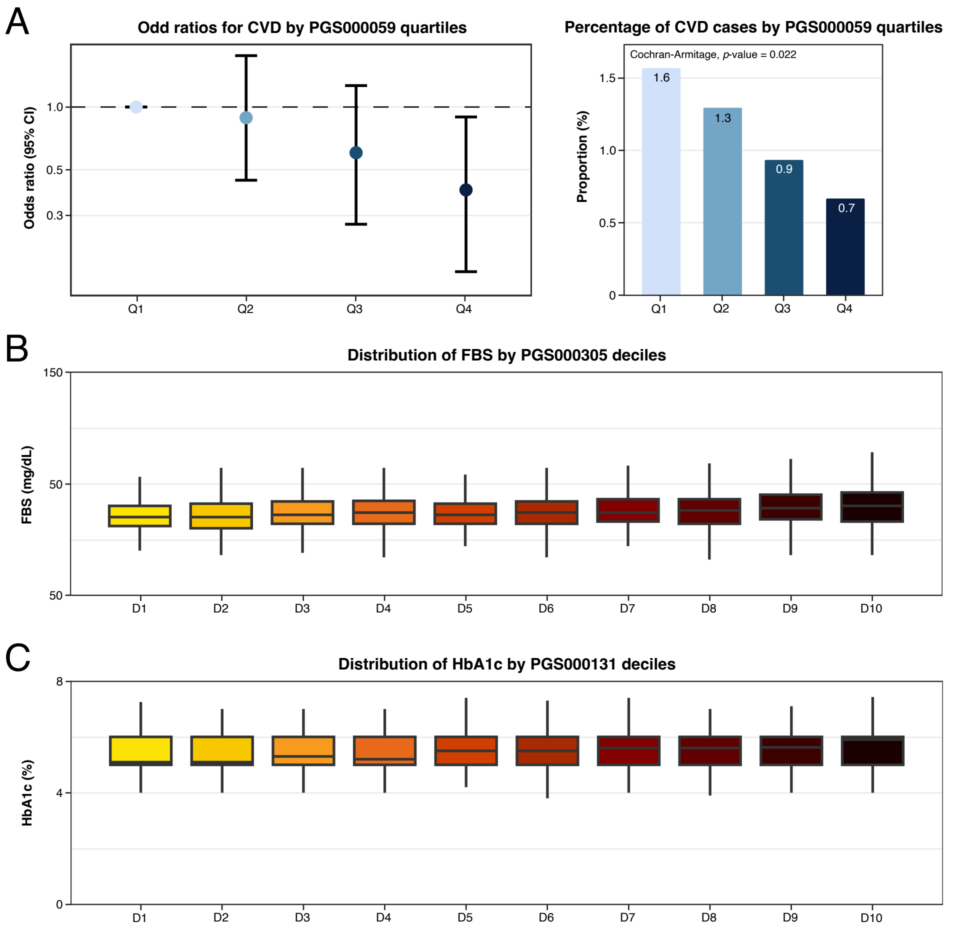

Figure S2. PRS-based risk stratification for traits with modest predictive performance in this cohort. Risk stratification across polygenic score quartiles (binary traits) and deciles (continuous traits) for the best-performing PRSs with modest PRS performances. (A) Odds ratios with 95% confidence interval for CVD cross PRS quartiles (PGS000059), adjusted for age, sex and PC1-10, are shown on the left, with corresponding case proportions per quartile on the right. Notably, the highest PRS quartile exhibited significantly lower odds of CVD and the lowest case proportion, indicating protective effect. (B-C) Boxplots show the distribution of glycemic trait (FBS and HbA1c) across PRS deciles using PGS000305 and PGS000131, respectively. While overall associations were significant, stratification patterns were less pronounced than observed for lipid traits. These findings suggest measurable but limited stratification utility in this population. Figures generated using R (ggplot2) and finalized in Adobe Illustrator.

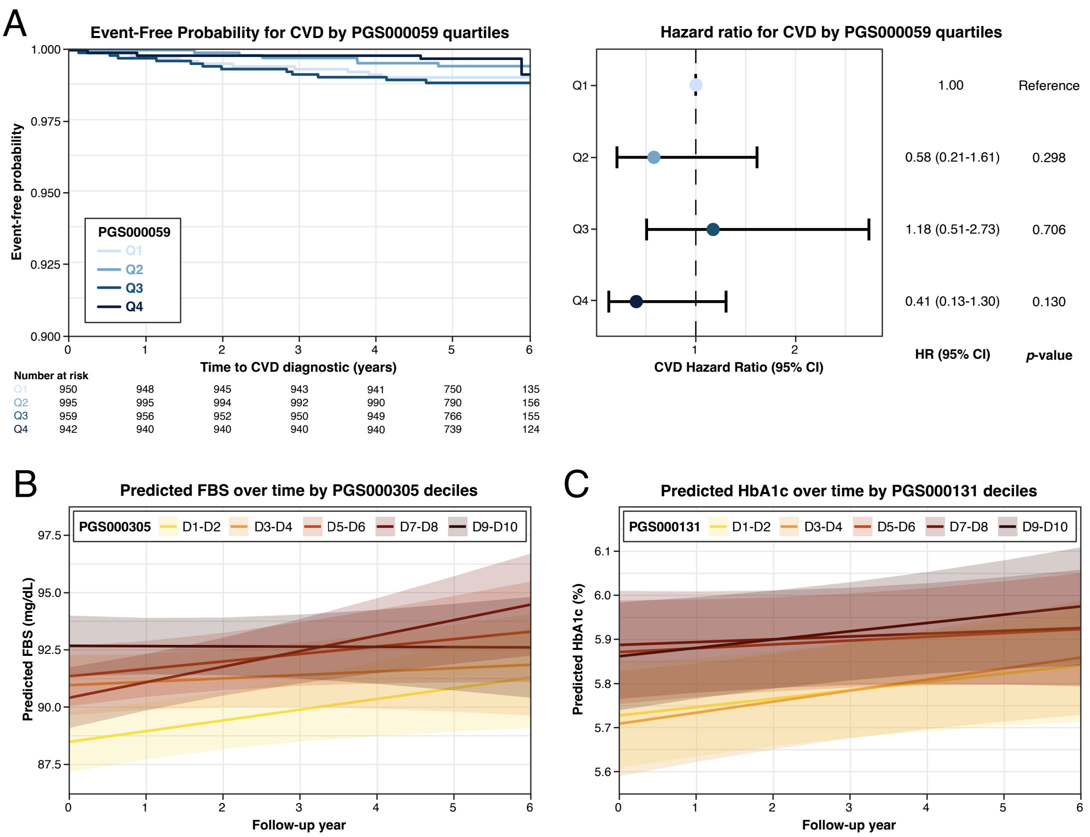

Figure S3. Survival and longitudinal analyses of cardiometabolic traits with modest PRS performance. (A) Kaplan-Meier survival curves (left) and adjusted hazard ratios with 95% confidence interval (right) from Cox proportional hazards models for incident CVD across PRS000059 quartiles, adjusted for age, sex, and PC1-10. No significant differences were observed among PRS quartiles. (B-C) Longitudinal prediction of glycemic traits using LMM assessments across PRS quintiles. Predicted mean values with 95% confidence intervals are shown for (B) FBS (PGS000305) and (C) HbA1c (PGS000131) over a six-year follow-up period. While PRS values were statistically associated with glycemic levels, the predicted values and stratification patterns did not differ between PRS groups, reflecting limited temporal predictive utility in this population. Figures generated using R (ggplot2) and finalized in Adobe Illustrator.

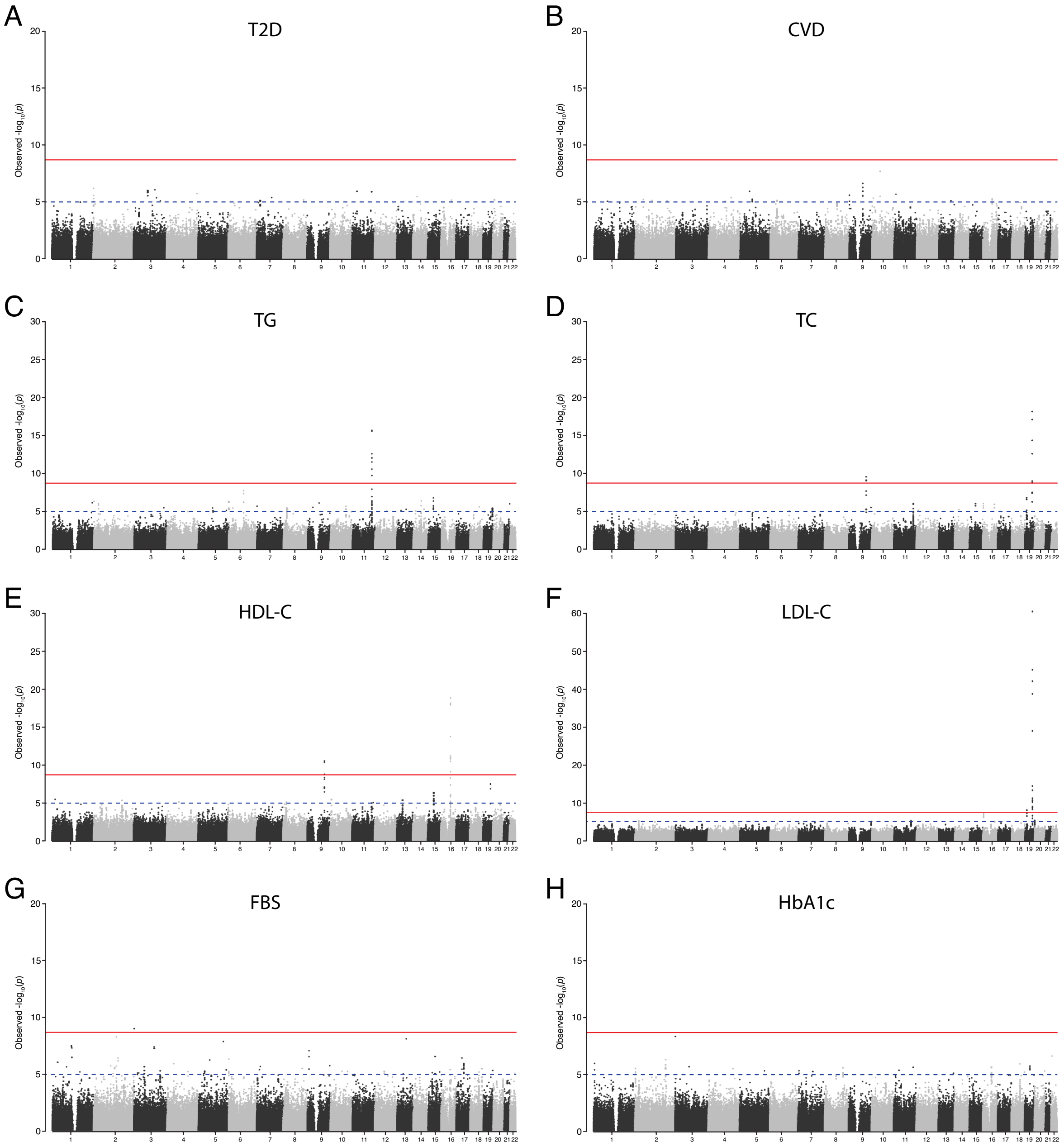

Figure S4. GWAS Manhattan plots for eight cardiometabolic traits. Manhattan plots showing GWAS results for eight cardiometabolic traits, including (A) T2D, (B) CVD, (C) TG, (D) TC, (E) HDL-C, (F) LDL-C, (G) FBS, and (H) HbA1c. The x-axis represent chromosomal positions across the genome, and the y-axis represents $-\log_{10}$(P) values of SNP-trait associations. The red horizontal line marks the genome-wide significance threshold (*p*-value = 5.0×10^{-8},) while the blue dashed line indicates the suggestive significance threshold (*p*-value = 1.0×10^{-5}, blue dashed line). Figures generated using R (ggplot2) and finalized in Adobe Illustrator.

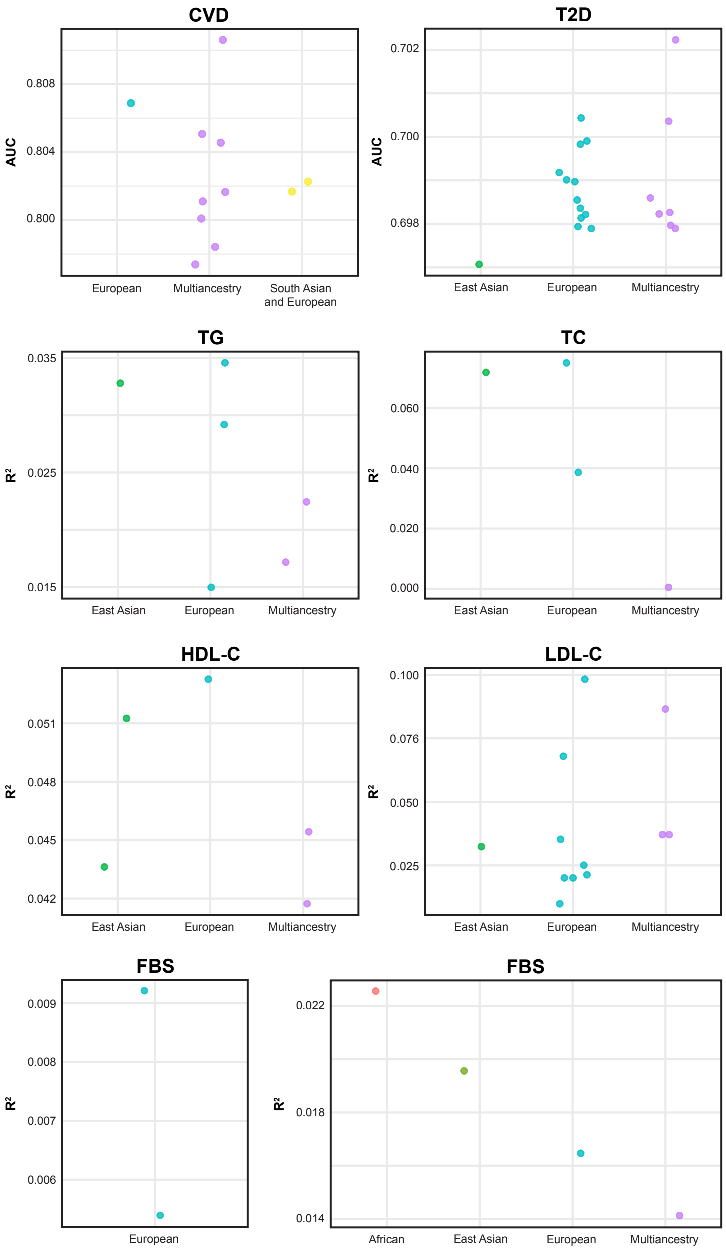

Figure S5. Comparison of PRS predictive performance across traits, stratified by the discovery GWAS ancestry. Scatter plot showing the distribution of PRS performance (AUC for binary traits, R^2 for continuous traits) for each score grouped by discovery ancestry: European, East Asian, Multi-ancestry, and others. The x-axis represents discovery ancestry, and the y-axis represents PRS performance (AUC or R^2). Figures generated using R (ggplot2) and finalized in Adobe Illustrator.

Table S1 Post-hoc analysis for significant baseline demographic variables

Variable	Comparison	p-value
Age	T2D vs. Control	1.61E-28
Age	CVD vs. Control	1.29E-13
Age	CVD vs. T2D	0.022112149
BMI	T2D vs. Control	8.64E-29
BMI	CVD vs. Control	1.60E-03
BMI	CVD vs. T2D	0.470663324
WC	T2D vs. Control	2.62E-32
WC	CVD vs. Control	1.92E-03
WC	CVD vs. T2D	0.34062883
SBP	T2D vs. Control	2.00E-17
SBP	CVD vs. Control	1.20E-06
SBP	CVD vs. T2D	0.672185747
DBP	T2D vs. Control	3.64E-08
DBP	CVD vs. Control	2.46E-02
DBP	CVD vs. T2D	1
TG	T2D vs. Control	5.59E-14
TG	CVD vs. Control	1.86E-01
TG	CVD vs. T2D	0.189500799
HDL-C	T2D vs. Control	1.76E-11
HDL-C	CVD vs. Control	4.23E-01
HDL-C	CVD vs. T2D	0.586501234
FBS	T2D vs. Control	2.77E-45
FBS	CVD vs. Control	1.20E-04
FBS	CVD vs. T2D	0.02394707
HbA1c	T2D vs. Control	9.20E-59
HbA1c	CVD vs. Control	4.48E-05
HbA1c	CVD vs. T2D	0.006932315
MAU/Cr ratio	T2D vs. Control	3.79E-22
MAU/Cr ratio	CVD vs. Control	1.46E-03
MAU/Cr ratio	CVD vs. T2D	0.854144592

Table S2 Polygenic risk score (PRS) for T2D, CVD, TC, TG, HDL-C, LDL-C, FBS, and HbA1c, accessed 13 November 2024

PGS_id	Reported trait	Mapped Trait (EFO label)	Number of Variants	Match rate(%) INFO08	Total Match INFO08	Match rate(%) INFO03	Total Match INFO03	Citation
PGS000014	Type 2 diabetes (T2D)	type 2 diabetes mellitus	6,917,436	13.6	941305	37.6	2602633	Khera AV et al. Nat Genet (2018)
PGS000020	Type 2 diabetes (T2D)	type 2 diabetes mellitus	7,502	19.9	1491	40.4	3034	Läll K et al. Genet Med (2016)
PGS000031	Type 2 diabetes (T2D)	type 2 diabetes mellitus	62	69.4	43	71	44	Vassy JL et al. Diabetes (2014)
PGS000032	Type 2 diabetes (based on SNPs involved in β-cell function)	type 2 diabetes mellitus	20	80	16	80	16	Vassy JL et al. Diabetes (2014)
PGS000033	Type 2 diabetes (based on SNPs involved in insulin resistance)	type 2 diabetes mellitus	10	80	8	80	8	Vassy JL et al. Diabetes (2014)
PGS000036	Type 2 diabetes (T2D)	type 2 diabetes mellitus	171,249	8.8	15120	25.9	44359	Mahajan A et al. Nat Genet (2018)
PGS000125	Type 2 diabetes (T2D)	type 2 diabetes mellitus	80	77.5	62	78.8	63	Qi Q et al. Diabetes (2017)
PGS000330	Type 2 diabetes (T2D)	type 2 diabetes mellitus	6,437,380	14	903884	38.5	2478340	Mars N et al. Nat Med (2020)
PGS000712	T2D (cases vs HbA1c filtered controls)	type 2 diabetes mellitus	183,695	17.9	32842	32	58789	Sinnott-Armstrong N et al. Nat Genet (2021)
PGS000713	Type 2 diabetes (T2D)	type 2 diabetes mellitus	183,830	17.9	32855	32	58818	Sinnott-Armstrong N et al. Nat Genet (2021)
PGS000729	Type 2 diabetes (T2D)	type 2 diabetes mellitus	2,017,388	10.6	213405	29.2	589870	Ritchie SC et al. Nat Metab (2021)
PGS000804	Type 2 diabetes (T2D)	type 2 diabetes mellitus	582	17	99	34.7	202	Polfus LM et al. HGG Adv (2021)
PGS000805	Type 2 diabetes (T2D)	type 2 diabetes mellitus	582	17	99	34.7	202	Polfus LM et al. HGG Adv (2021)
PGS000806	Type 2 diabetes (T2D)	type 2 diabetes mellitus	582	17	99	34.7	202	Polfus LM et al. HGG Adv (2021)
PGS000807	Type 2 diabetes (T2D)	type 2 diabetes mellitus	582	17	99	34.7	202	Polfus LM et al. HGG Adv (2021)
PGS000808	Type 2 diabetes (T2D)	type 2 diabetes mellitus	582	17	99	34.7	202	Polfus LM et al. HGG Adv (2021)
PGS000832	Type 2 diabetes (T2D)	type 2 diabetes mellitus	384	19.8	76	34.9	134	Aly DM et al. Nat Genet (2021)
PGS000848	Type 2 diabetes (based on SNPs associated with adiposity)	type 2 diabetes mellitus	6	83.3	5	83.3	5	Aly DM et al. Nat Genet (2021)
PGS000849	Type 2 diabetes (based on SNPs associated with impared lipid)	type 2 diabetes mellitus	3	100	3	100	3	Aly DM et al. Nat Genet (2021)
PGS000850	Type 2 diabetes (based on SNPs associated with insulin action)	type 2 diabetes mellitus	16	62.5	10	62.5	10	Aly DM et al. Nat Genet (2021)
PGS000851	Type 2 diabetes (based on SNPs associated with insulin action/secretion)	type 2 diabetes mellitus	37	67.6	25	75.7	28	Aly DM et al. Nat Genet (2021)
PGS000852	Type 2 diabetes (based on SNPs associated with insulin secretion)	type 2 diabetes mellitus	8	87.5	7	87.5	7	Aly DM et al. Nat Genet (2021)
PGS000853	Type 2 diabetes (based on SNPs associated with insulin secretion)	type 2 diabetes mellitus	21	76.2	16	85.7	18	Aly DM et al. Nat Genet (2021)
PGS000854	Type 2 diabetes (based on SNPs associated with beta cell function)	type 2 diabetes mellitus	27	63	17	74.1	20	Aly DM et al. Nat Genet (2021)
PGS000855	Type 2 diabetes (based on SNPs associated with lipodystrophy)	type 2 diabetes mellitus	18	77.8	14	77.8	14	Aly DM et al. Nat Genet (2021)
PGS000856	Type 2 diabetes (based on SNPs associated with liver lipids)	type 2 diabetes mellitus	3	100	3	100	3	Aly DM et al. Nat Genet (2021)
PGS000857	Type 2 diabetes (based on SNPs associated with obesity)	type 2 diabetes mellitus	4	75	3	75	3	Aly DM et al. Nat Genet (2021)
PGS000858	Type 2 diabetes (based on SNPs associated with proinsulin level)	type 2 diabetes mellitus	6	100	6	100	6	Aly DM et al. Nat Genet (2021)
PGS000864	Type 2 diabetes (T2D)	type 2 diabetes mellitus	389,243	6.6	25502	16.9	65643	Aly DM et al. Nat Genet (2021)
PGS000868	Type 2 diabetes (T2D)	type 2 diabetes mellitus	221	22.2	49	35.7	79	Aksit MA et al. J Clin Endocrinol Metab (2020)
PGS001294	Non-insulin-dependent diabetes (time-to-event)	type 2 diabetes mellitus	3,496	20.7	722	30.9	1080	Tanigawa Y et al. PLoS Genet (2022)

PGS001295	Type 2 diabetes (T2D)	type 2 diabetes mellitus	385	21.3	82	29.1	112	Tanigawa Y et al. PLoS Genet (2022)
PGS001357	Type 2 diabetes (T2D)	type 2 diabetes mellitus	2,996,761	15	450653	42	1257435	Ye Y et al. Circ Genom Precis Med (2021)
PGS001781	Type 2 diabetes (T2D)	type 2 diabetes mellitus	1,091,673	18.6	203101	39.2	428378	Tamlander M et al. Commun Biol (2022)
PGS001818	Type 2 diabetes (T2D)	type 2 diabetes mellitus	30,745	19.7	6059	34.4	10569	Privé F et al. Am J Hum Genet (2022)
PGS002026	Type 2 diabetes (T2D)	type 2 diabetes mellitus	830,783	18.4	153205	38.9	322974	Privé F et al. Am J Hum Genet (2022)
PGS002243	Type 2 diabetes (T2D)	type 2 diabetes mellitus	6,431,973	14.2	914528	39.1	2514216	Mars N et al. Cell Genom (2022)
PGS002277	Type 2 diabetes (based on SNPs associated with insulin secretion)	type 2 diabetes mellitus	8	87.5	7	87.5	7	Siddiqui MK et al. Diabetologia (2022)
PGS002308	Type 2 diabetes (T2D)	type 2 diabetes mellitus	1,259,754	17.9	226073	37.8	476057	Ge T et al. Genome Med (2022)
PGS002354	Type 2 diabetes (T2D)	type 2 diabetes mellitus	1,109,311	18.7	207730	39.5	437977	Weissbrod O et al. Nat Genet (2022)
PGS002379	Type 2 diabetes (T2D)	type 2 diabetes mellitus	920,930	19.2	177230	39.6	365125	Weissbrod O et al. Nat Genet (2022)
PGS002426	Type 2 diabetes (T2D)	type 2 diabetes mellitus	3,947	5	198	11.2	444	Weissbrod O et al. Nat Genet (2022)
PGS002475	Type 2 diabetes (T2D)	type 2 diabetes mellitus	16,275	3.8	617	9.7	1574	Weissbrod O et al. Nat Genet (2022)
PGS002524	Type 2 diabetes (T2D)	type 2 diabetes mellitus	95,287	3.5	3313	8.8	8417	Weissbrod O et al. Nat Genet (2022)
PGS002573	Type 2 diabetes (T2D)	type 2 diabetes mellitus	673	6.5	44	12.2	82	Weissbrod O et al. Nat Genet (2022)
PGS002622	Type 2 diabetes (T2D)	type 2 diabetes mellitus	293	9.6	28	17.7	52	Weissbrod O et al. Nat Genet (2022)
PGS002671	Type 2 diabetes (T2D)	type 2 diabetes mellitus	258,382	7.8	20090	20.4	52592	Weissbrod O et al. Nat Genet (2022)
PGS002720	Type 2 diabetes (T2D)	type 2 diabetes mellitus	911,809	18.5	168632	39.7	361546	Weissbrod O et al. Nat Genet (2022)
PGS002733	Type 2 diabetes (T2D)	type 2 diabetes mellitus	17	52.9	9	70.6	12	Pezzilli S et al. Diabetes Metab (2022)
PGS002771	Type 2 diabetes (T2D)	type 2 diabetes mellitus	1,091,608	18.6	203098	39.2	428358	Mars N et al. Am J Hum Genet (2022)
PGS002779	Incident type 2 diabetes	type 2 diabetes mellitus	46,353	15.9	7389	34.3	15895	Wong CK et al. PLoS One (2022)
PGS002780	Incident type 2 diabetes	type 2 diabetes mellitus	419,209	15.5	64829	33.5	140296	Wong CK et al. PLoS One (2022)
PGS003089	Type 2 diabetes (T2D)	type 2 diabetes mellitus	488,969	14	68575	39.9	195009	Ma Y et al. Am J Hum Genet (2022)
PGS003090	Type 2 diabetes (T2D)	type 2 diabetes mellitus	1,888	11.1	209	26.2	495	Ma Y et al. Am J Hum Genet (2022)
PGS003091	Type 2 diabetes (T2D)	type 2 diabetes mellitus	2,707	8.6	233	20.1	543	Ma Y et al. Am J Hum Genet (2022)
PGS003092	Type 2 diabetes (T2D)	type 2 diabetes mellitus	10,304,000	10	1028951	26.9	2771108	Ma Y et al. Am J Hum Genet (2022)
PGS003093	Type 2 diabetes (T2D)	type 2 diabetes mellitus	1,113,832	18.8	209152	39.4	438880	Ma Y et al. Am J Hum Genet (2022)
PGS003094	Type 2 diabetes (T2D)	type 2 diabetes mellitus	266,890	12.4	33121	34.1	90974	Ma Y et al. Am J Hum Genet (2022)
PGS003095	Type 2 diabetes (T2D)	type 2 diabetes mellitus	45	44.4	20	53.3	24	Ma Y et al. Am J Hum Genet (2022)
PGS003096	Type 2 diabetes (T2D)	type 2 diabetes mellitus	46	43.5	20	54.3	25	Ma Y et al. Am J Hum Genet (2022)
PGS003097	Type 2 diabetes (T2D)	type 2 diabetes mellitus	229	9.2	21	22.3	51	Ma Y et al. Am J Hum Genet (2022)
PGS003098	Type 2 diabetes (T2D)	type 2 diabetes mellitus	1,116,101	18.8	209832	39.4	439861	Ma Y et al. Am J Hum Genet (2022)
PGS003099	Type 2 diabetes (T2D)	type 2 diabetes mellitus	555,512	12.8	71361	35.6	197590	Ma Y et al. Am J Hum Genet (2022)
PGS003100	Type 2 diabetes (T2D)	type 2 diabetes mellitus	5,693	14.5	824	34.9	1988	Ma Y et al. Am J Hum Genet (2022)
PGS003101	Type 2 diabetes (T2D)	type 2 diabetes mellitus	5,693	14.5	824	34.9	1988	Ma Y et al. Am J Hum Genet (2022)
PGS003102	Type 2 diabetes (T2D)	type 2 diabetes mellitus	5,052,574	13.4	677830	36.9	1865731	Ma Y et al. Am J Hum Genet (2022)
PGS003103	Type 2 diabetes (T2D)	type 2 diabetes mellitus	945,820	17.9	169445	39.4	373118	Ma Y et al. Am J Hum Genet (2022)
PGS003104	Type 2 diabetes (T2D)	type 2 diabetes mellitus	374,510	11.3	42494	30.9	115750	Ma Y et al. Am J Hum Genet (2022)
PGS003105	Type 2 diabetes (T2D)	type 2 diabetes mellitus	187	24.6	46	36.4	68	Ma Y et al. Am J Hum Genet (2022)
PGS003106	Type 2 diabetes (T2D)	type 2 diabetes mellitus	187	24.6	46	36.4	68	Ma Y et al. Am J Hum Genet (2022)
PGS003107	Type 2 diabetes (T2D)	type 2 diabetes mellitus	995	14	139	34.3	341	Ma Y et al. Am J Hum Genet (2022)
PGS003108	Type 2 diabetes (T2D)	type 2 diabetes mellitus	1,118,480	18.8	210360	39.4	440767	Ma Y et al. Am J Hum Genet (2022)
PGS003109	Type 2 diabetes (T2D)	type 2 diabetes mellitus	311,565	10.8	33502	29.7	92426	Ma Y et al. Am J Hum Genet (2022)
PGS003110	Type 2 diabetes (T2D)	type 2 diabetes mellitus	118	21.2	25	31.4	37	Ma Y et al. Am J Hum Genet (2022)
PGS003111	Type 2 diabetes (T2D)	type 2 diabetes mellitus	143	18.2	26	27.3	39	Ma Y et al. Am J Hum Genet (2022)
PGS003112	Type 2 diabetes (T2D)	type 2 diabetes mellitus	222	7.7	17	19.4	43	Ma Y et al. Am J Hum Genet (2022)
PGS003113	Type 2 diabetes (T2D)	type 2 diabetes mellitus	1,117,087	18.8	209935	39.4	440008	Ma Y et al. Am J Hum Genet (2022)

PGS003114	Type 2 diabetes (T2D)	type 2 diabetes mellitus	555,528	12.8	71361	35.6	197590	Ma Y et al. Am J Hum Genet (2022)
PGS003115	Type 2 diabetes (T2D)	type 2 diabetes mellitus	31,462	14.4	4527	35.9	11292	Ma Y et al. Am J Hum Genet (2022)
PGS003116	Type 2 diabetes (T2D)	type 2 diabetes mellitus	31,462	14.4	4527	35.9	11292	Ma Y et al. Am J Hum Genet (2022)
PGS003117	Type 2 diabetes (T2D)	type 2 diabetes mellitus	5,052,993	13.4	677852	36.9	1865760	Ma Y et al. Am J Hum Genet (2022)
PGS003118	Type 2 diabetes (T2D)	type 2 diabetes mellitus	945,921	17.9	169456	39.4	373130	Ma Y et al. Am J Hum Genet (2022)
PGS003119	Type 2 diabetes (T2D)	type 2 diabetes mellitus	407,553	10.6	43264	29	118065	Ma Y et al. Am J Hum Genet (2022)
PGS003120	Type 2 diabetes (T2D)	type 2 diabetes mellitus	193	22.8	44	34.2	66	Ma Y et al. Am J Hum Genet (2022)
PGS003121	Type 2 diabetes (T2D)	type 2 diabetes mellitus	193	22.8	44	34.2	66	Ma Y et al. Am J Hum Genet (2022)
PGS003122	Type 2 diabetes (T2D)	type 2 diabetes mellitus	264	12.1	32	25.4	67	Ma Y et al. Am J Hum Genet (2022)
PGS003123	Type 2 diabetes (T2D)	type 2 diabetes mellitus	1,119,522	18.8	210467	39.4	440923	Ma Y et al. Am J Hum Genet (2022)
PGS003353	Type 2 diabetes (T2D)	type 2 diabetes mellitus	287	16.7	48	30	86	Kim YJ et al. Nat Commun (2022)
PGS003402	Type 2 diabetes (T2D)	type 2 diabetes mellitus	6,838	15.2	1036	34.2	2342	Lamri A et al. Elife (2022)
PGS003443	Type 2 diabetes (T2D)	type 2 diabetes mellitus	1,092,496	18.8	205784	39.7	433343	Huerta-Chagoya A et al. Diabetologia (2023)
PGS003444	Type 2 diabetes (T2D)	type 2 diabetes mellitus	1,001,579	19.2	192109	39.3	393686	Huerta-Chagoya A et al. Diabetologia (2023)
PGS003445	Type 2 diabetes (T2D)	type 2 diabetes mellitus	1,149,210	18.8	215864	39.8	457438	Huerta-Chagoya A et al. Diabetologia (2023)
PGS003728	Type 2 diabetes (T2D)	type 2 diabetes mellitus	183	16.4	30	27.9	51	Wedekind LE et al. Diabetologia (2023)
PGS003729	Type 2 diabetes (T2D)	type 2 diabetes mellitus	293	23.2	68	35.8	105	Wedekind LE et al. Diabetologia (2023)
PGS003730	Type 2 diabetes (T2D)	type 2 diabetes mellitus	287	21.3	61	38	109	Wedekind LE et al. Diabetologia (2023)
PGS003731	Type 2 diabetes (T2D)	type 2 diabetes mellitus	282	21.3	60	37.6	106	Wedekind LE et al. Diabetologia (2023)
PGS003732	Type 2 diabetes (T2D)	type 2 diabetes mellitus	287	21.3	61	38	109	Wedekind LE et al. Diabetologia (2023)
PGS003733	Type 2 diabetes (T2D)	type 2 diabetes mellitus	287	21.3	61	38	109	Wedekind LE et al. Diabetologia (2023)
PGS003734	Type 2 diabetes (T2D)	type 2 diabetes mellitus	280	20.4	57	37.1	104	Wedekind LE et al. Diabetologia (2023)
PGS003735	Type 2 diabetes (T2D)	type 2 diabetes mellitus	276	20.7	57	37.7	104	Wedekind LE et al. Diabetologia (2023)
PGS003751	Type 2 diabetes (T2D)	type 2 diabetes mellitus	354	24.6	87	46.6	165	Shoaib M et al. Genet Epidemiol (2023)
PGS003752	Type 2 diabetes (T2D)	type 2 diabetes mellitus	333	24	80	46.2	154	Shoaib M et al. Genet Epidemiol (2023)
PGS003867	Type 2 diabetes (T2D)	type 2 diabetes mellitus	1,068,166	18.7	199783	39.5	421988	Shim I et al. Nature Communications (2023)
PGS003982	Type 2 diabetes (T2D)	type 2 diabetes mellitus	1,071,764	18.6	198905	39.4	421773	Monti R et al. Am J Hum Genet (2024)
PGS003998	Type 2 diabetes (T2D)	type 2 diabetes mellitus	5,548	22.7	1257	43.3	2402	Monti R et al. Am J Hum Genet (2024)
PGS004014	Type 2 diabetes (T2D)	type 2 diabetes mellitus	95,649	19.2	18320	39.9	38208	Monti R et al. Am J Hum Genet (2024)
PGS004024	Type 2 diabetes (T2D)	type 2 diabetes mellitus	958,046	18.5	176877	39.2	375146	Monti R et al. Am J Hum Genet (2024)
PGS004039	Type 2 diabetes (T2D)	type 2 diabetes mellitus	958,046	18.5	176877	39.2	375146	Monti R et al. Am J Hum Genet (2024)

PGS004052	Type 2 diabetes (T2D)	type 2 diabetes mellitus	800,598	18.9	151515	40.1	321325	Monti R et al. Am J Hum Genet (2024)
PGS004068	Type 2 diabetes (T2D)	type 2 diabetes mellitus	800,598	18.9	151515	40.1	321325	Monti R et al. Am J Hum Genet (2024)
PGS004082	Type 2 diabetes (T2D)	type 2 diabetes mellitus	1,043,329	18.6	193751	39.3	409797	Monti R et al. Am J Hum Genet (2024)
PGS004106	Type 2 diabetes (T2D)	type 2 diabetes mellitus	35	68.6	24	77.1	27	Monti R et al. Am J Hum Genet (2024)
PGS004122	Type 2 diabetes (T2D)	type 2 diabetes mellitus	297	32.3	96	48.8	145	Monti R et al. Am J Hum Genet (2024)
PGS004136	Type 2 diabetes (T2D)	type 2 diabetes mellitus	930,497	18.4	171086	39.5	367736	Monti R et al. Am J Hum Genet (2024)
PGS004152	Type 2 diabetes (T2D)	type 2 diabetes mellitus	1,071,786	18.6	198910	39.4	421781	Monti R et al. Am J Hum Genet (2024)
PGS004181	Type 2 diabetes	type 2 diabetes mellitus	10,202	14.9	1520	31.6	3221	Raben TG et al. Sci Rep (2023)
PGS004182	Type 2 diabetes	type 2 diabetes mellitus	10,778	15	1616	31.4	3380	Raben TG et al. Sci Rep (2023)
PGS004183	Type 2 diabetes	type 2 diabetes mellitus	8,154	15.5	1263	32	2612	Raben TG et al. Sci Rep (2023)
PGS004184	Type 2 diabetes	type 2 diabetes mellitus	9,645	15.1	1460	31.4	3026	Raben TG et al. Sci Rep (2023)
PGS004185	Type 2 diabetes	type 2 diabetes mellitus	3,277	17.2	563	35.1	1150	Raben TG et al. Sci Rep (2023)
PGS004223	Type 2 diabetes	type 2 diabetes mellitus	139	38.8	54	51.1	71	Lin J et al. Sci Total Environ (2023)
PGS004225	Type 2 diabetes	type 2 diabetes mellitus	46	82.6	38	82.6	38	Liu J et al. Nutrients (2023)
PGS004226	Type 2 diabetes	type 2 diabetes mellitus	50	74	37	74	37	Liu J et al. Nutrients (2023)
PGS004310	Type 2 diabetes (T2D)	type 2 diabetes mellitus	7,000	21.3	1493	41.2	2882	Ohta R et al. Nat Commun (2024)
PGS004311	Type 2 diabetes (T2D)	type 2 diabetes mellitus	4,000	20.2	808	40.9	1637	Ohta R et al. Nat Commun (2024)
PGS004312	Type 2 diabetes (T2D)	type 2 diabetes mellitus	7,000	21.4	1498	41.6	2909	Ohta R et al. Nat Commun (2024)
PGS004313	Type 2 diabetes (T2D)	type 2 diabetes mellitus	8,000	21.1	1689	41.1	3285	Ohta R et al. Nat Commun (2024)
PGS004314	Type 2 diabetes (T2D)	type 2 diabetes mellitus	6,000	21.3	1280	41.6	2494	Ohta R et al. Nat Commun (2024)
PGS004323	Type 2 diabetes	type 2 diabetes mellitus	91	25.3	23	33	30	Tan Q et al. J Hazard Mater (2023)
PGS004499	Type 2 diabetes (T2D)	type 2 diabetes mellitus	1,059,939	18.6	197409	39.5	418909	Jung H et al. Commun Biol (2024)
PGS004569	Type 2 diabetes (T2D)	type 2 diabetes mellitus	1,059,939	18.6	197409	39.5	418909	Jung H et al. Commun Biol (2024)
PGS004602	Type 2 diabetes (T2D)	type 2 diabetes mellitus	424	20.8	88	35.6	151	Zhuang P et al. Diabetes Care (2021)
PGS004615	Type 2 diabetes (T2D)	type 2 diabetes mellitus	855,161	17.3	148065	37.3	318800	Ojima T et al. Nat Genet (2024)
PGS004616	Type 2 diabetes (T2D)	type 2 diabetes mellitus	855,161	17.3	148065	37.3	318800	Ojima T et al. Nat Genet (2024)
PGS004617	Type 2 diabetes (T2D)	type 2 diabetes mellitus	855,161	17.3	148065	37.3	318800	Ojima T et al. Nat Genet (2024)
PGS004618	Type 2 diabetes (T2D)	type 2 diabetes mellitus	728,824	14.1	102597	31.7	230810	Ojima T et al. Nat Genet (2024)
PGS004619	Type 2 diabetes (T2D)	type 2 diabetes mellitus	728,824	14.1	102597	31.7	230810	Ojima T et al. Nat Genet (2024)
PGS004620	Type 2 diabetes (T2D)	type 2 diabetes mellitus	728,824	14.1	102597	31.7	230810	Ojima T et al. Nat Genet (2024)
PGS004837	Type 2 diabetes (T2D)	type 2 diabetes mellitus	3,306,136	14.4	476141	36.6	1210727	Truong B et al. Cell Genom (2024)
PGS004838	Type 2 diabetes (T2D)	type 2 diabetes mellitus	6,586,458	14	923204	39.2	2580175	Truong B et al. Cell Genom (2024)
PGS004839	Type 2 diabetes (T2D)	type 2 diabetes mellitus	4,594,694	11	507327	28.1	1292919	Truong B et al. Cell Genom (2024)
PGS004840	Type 2 diabetes (T2D)	type 2 diabetes mellitus	6,586,458	14	923204	39.2	2580175	Truong B et al. Cell Genom (2024)
PGS004859	Type 2 diabetes (T2D)	type 2 diabetes mellitus	1,108,235	18.7	207460	39.3	435091	Deutsch AJ et al. Diabetes Care (2023)
PGS004868	Type 2 diabetes (T2D)	type 2 diabetes mellitus	6,580,804	13.8	907889	38.2	2514818	Yun JS et al. Cardiovasc Diabetol (2022)
PGS004870	Type 2 diabetes (T2D)	type 2 diabetes mellitus	930,506	19.2	178297	40	371817	Jermy B et al. Nat Commun (2024)
PGS004887	Type 2 diabetes (T2D)	type 2 diabetes mellitus	1,117,628	18.8	210374	39.4	440668	Mandla R et al. Genome Med (2024)
PGS004923	Type 2 diabetes (T2D)	type 2 diabetes mellitus	1,349,896	17.6	236964	36.7	495971	Ritchie SC et al. medRxiv (2024) | Pre
PGS005021	Type 2 diabetes (T2D) (PheCode 250.2)	type 2 diabetes mellitus	1,286,612	17.5	225726	36.9	475197	Gunn S et al. HGG Adv (2024)

PGS005022	Type 2 diabetes (T2D) (PheCode 250.2)	type 2 diabetes mellitus	1,286,612	17.5	225726	36.9	475197	Gunn S et al. HGG Adv (2024)
PGS005023	Type 2 diabetes (T2D) (PheCode 250.2)	type 2 diabetes mellitus	1,286,612	17.5	225726	36.9	475197	Gunn S et al. HGG Adv (2024)
PGS005024	Type 2 diabetes (T2D) (PheCode 250.2)	type 2 diabetes mellitus	1,286,612	17.5	225726	36.9	475197	Gunn S et al. HGG Adv (2024)
PGS005025	Type 2 diabetes (T2D) (PheCode 250.2)	type 2 diabetes mellitus	1,286,612	17.5	225726	36.9	475197	Gunn S et al. HGG Adv (2024)
PGS005026	Type 2 diabetes (T2D) (PheCode 250.2)	type 2 diabetes mellitus	1,286,612	17.5	225726	36.9	475197	Gunn S et al. HGG Adv (2024)
PGS005027	Type 2 diabetes (T2D) (PheCode 250.2)	type 2 diabetes mellitus	1,273,897	17.5	223565	36.9	470532	Gunn S et al. HGG Adv (2024)
PGS005028	Type 2 diabetes (T2D) (PheCode 250.2)	type 2 diabetes mellitus	1,273,897	17.5	223565	36.9	470532	Gunn S et al. HGG Adv (2024)
PGS005029	Type 2 diabetes (T2D) (PheCode 250.2)	type 2 diabetes mellitus	1,273,897	17.5	223565	36.9	470532	Gunn S et al. HGG Adv (2024)
PGS005030	Type 2 diabetes (T2D) (PheCode 250.2)	type 2 diabetes mellitus	1,273,897	17.5	223565	36.9	470532	Gunn S et al. HGG Adv (2024)
PGS005031	Type 2 diabetes (T2D) (PheCode 250.2)	type 2 diabetes mellitus	1,273,897	17.5	223565	36.9	470532	Gunn S et al. HGG Adv (2024)
PGS005032	Type 2 diabetes (T2D) (PheCode 250.2)	type 2 diabetes mellitus	1,273,897	17.5	223565	36.9	470532	Gunn S et al. HGG Adv (2024)
PGS005033	Type 2 diabetes (T2D) (PheCode 250.2)	type 2 diabetes mellitus	1,277,826	17.6	224904	37.1	473588	Gunn S et al. HGG Adv (2024)
PGS000010	Coronary heart disease	coronary artery disease	27	74.1	20	74.1	20	Mega JL et al. Lancet (2015)
PGS000011	Coronary artery disease	coronary artery disease	50	74	37	76	38	Tada H et al. Eur Heart J (2015)
PGS000012	Coronary artery disease	coronary artery disease	49310	19.9	9832	40.5	19971	Abraham G et al. Eur Heart J (2016)
PGS000013	Coronary artery disease	coronary artery disease	6630150	14	926074	38.7	2566569	Khera AV et al. Nat Genet (2018)
PGS000018	Coronary artery disease	coronary artery disease	1745179	10.5	183160	29.9	521233	Inouye M et al. J Am Coll Cardiol (2018)
PGS000019	Coronary artery disease	coronary artery disease	192	17.2	33	35.4	68	Paquette M et al. J Clin Lipidol (2017)
PGS000038	Stroke	stroke	90	14.4	13	38.9	35	Rutten-Jacobs LC et al. BMJ (2018)
PGS000039	Ischemic stroke	stroke,<br>Ischemic stroke	3225583	10.1	325144	28.3	914278	Abraham G et al. Nat Commun (2019)
PGS000057	Coronary heart disease	coronary artery disease	57	68.4	39	71.9	41	Natarajan P et al. Circulation (2017)
PGS000058	Coronary artery disease	coronary artery disease	204	37.3	76	50	102	Morieri ML et al. Diabetes Care (2018)
PGS000059	Coronary heart disease	coronary artery disease	46	58.7	27	58.7	27	Hajek C et al. Circ Genom Precis Med (2018)
PGS000116	Coronary artery disease	coronary artery disease	40079	14.2	5672	31.8	12758	Elliott J et al. JAMA (2020)
PGS000117	Cardiovascular disease	cardiovascular disease	297862	13.7	40869	33.1	98620	Elliott J et al. JAMA (2020)
PGS000200	Coronary heart disease	coronary artery disease	28	78.6	22	78.6	22	Tikkanen E et al. Arterioscler Thromb Vasc Biol (2013)
PGS000296	Coronary artery disease	coronary artery disease	6630150	14	926074	38.7	2566569	Wang M et al. J Am Coll Cardiol (2020)
PGS000329	Coronary heart disease	coronary artery disease	6423165	14.1	904117	38.6	2479159	Mars N et al. Nat Med (2020)
PGS000337	Coronary artery disease	coronary artery disease	75028	14.1	10584	32.5	24381	Koyama S et al. Nat Genet (2020)
PGS000349	Coronary artery disease	coronary artery disease	70	71.4	50	75.7	53	Pechlivanis S et al. BMC Med Genet (2020)
PGS000665	Ischemic stroke	stroke,<br>Ischemic stroke	32	28.1	9	53.1	17	Marston NA et al. Circulation (2020)
PGS000709	Heart failure	heart failure	183287	17.9	32775	32	58655	Sinnott-Armstrong N et al. Nat Genet (2021)
PGS000710	Myocardial infarction	myocardial infarction	183566	17.9	32811	32	58729	Sinnott-Armstrong N et al. Nat Genet (2021)
PGS000746	Coronary artery disease	coronary artery disease	1940	15.7	305	39.3	762	Gola D et al. Circ Genom Precis Med (2020)
PGS000747	Coronary artery disease	coronary artery disease	375822	12.5	46990	35.1	131946	Gola D et al. Circ Genom Precis Med (2020)
PGS000748	Coronary artery disease	coronary artery disease	3423987	12.5	427636	35.3	1207915	Gola D et al. Circ Genom Precis Med (2020)

PGS000749	Coronary artery disease	coronary artery disease	1056021	13	137726	36.7	387324	Gola D et al. Circ Genom Precis Med (2020)
PGS000798	Coronary heart disease	coronary artery disease	157	29.9	47	42	66	Severance LM et al. J Cardiovasc Comput Tomogr (2019)
PGS000818	Coronary heart disease	coronary artery disease	138	57.2	79	63	87	Bauer A et al. Genet Epidemiol (2021)
PGS000863	Cardiovascular disease	cardiovascular disease	330	32.1	106	46.1	152	Aly DM et al. Nat Genet (2021)
PGS000899	Coronary heart disease	coronary artery disease	176	36.9	65	50	88	Feitosa MF et al. Circ Genom Precis Med (2021)
PGS000911	Ischemic stroke	stroke,<br>Ischemic stroke	530933	14.9	79262	41.5	220398	O'Sullivan JW et al. Circ Genom Precis Med (2021)
PGS000962	Chronic ischaemic heart disease (time-to-event)	Myocardial Ischemia	2168	21	456	29.2	634	Tanigawa Y et al. PLoS Genet (2022)
PGS001048	NSTEMI (algorithmically-defined)	Non-ST Elevation Myocardial Infarction	687	21.3	146	31.1	214	Tanigawa Y et al. PLoS Genet (2022)
PGS001314	Acute myocardial infarction (time-to-event)	myocardial infarction	1108	21.1	234	30	332	Tanigawa Y et al. PLoS Genet (2022)
PGS001315	Myocardial infarction	myocardial infarction	1788	21.5	385	30	536	Tanigawa Y et al. PLoS Genet (2022)
PGS001316	Myocardial infarction (algorithmically-defined)	myocardial infarction	1831	21.8	400	30.4	556	Tanigawa Y et al. PLoS Genet (2022)
PGS001317	Vascular/heart problems diagnosed by doctor Heart attack	myocardial infarction	1030	20.8	214	28.4	293	Tanigawa Y et al. PLoS Genet (2022)
PGS001335	Vascular/heart problems diagnosed by doctor High BP	cardiovascular disease	14076	18.9	2654	30.9	4352	Tanigawa Y et al. PLoS Genet (2022)
PGS001355	Coronary artery disease	coronary artery disease	2994055	15	450292	42	1256664	Ye Y et al. Circ Genom Precis Med (2021)
PGS001780	Coronary heart disease	coronary artery disease	1090048	18.6	202777	39.2	427682	Tamlander M et al. Commun Biol (2022)
PGS001790	Heart failure	heart failure	910146	19.4	176727	41.5	377471	Wang Y et al. Cell Genom (2023)
PGS001793	Stroke	stroke	910099	19.4	176722	41.5	377466	Wang Y et al. Cell Genom (2023)
PGS001798	Stroke	stroke	884168	19.5	172229	41.5	367143	Wang Y et al. Cell Genom (2023)
PGS001839	Coronary atherosclerosis	coronary atherosclerosis	25425	19.5	4959	33.9	8629	Privé F et al. Am J Hum Genet (2022)
PGS001843	Peripheral vascular disease, unspecified	peripheral vascular disease	242	31	75	47.9	116	Privé F et al. Am J Hum Genet (2022)
PGS001847	Circulatory disease NEC	vascular disease	594	25.6	152	43.3	257	Privé F et al. Am J Hum Genet (2022)
PGS002048	Coronary atherosclerosis	coronary atherosclerosis	762124	18.4	140456	38.9	296089	Privé F et al. Am J Hum Genet (2022)
PGS002053	Cerebrovascular disease	cerebrovascular disorder	599726	18.4	110052	38.7	232085	Privé F et al. Am J Hum Genet (2022)
PGS002055	Peripheral vascular disease, unspecified	peripheral vascular disease	599514	18.4	110310	38.7	232192	Privé F et al. Am J Hum Genet (2022)
PGS002059	Circulatory disease NEC	vascular disease	604572	18.4	110981	38.5	232759	Privé F et al. Am J Hum Genet (2022)
PGS002244	Coronary artery disease	coronary artery disease	6576338	13.8	908253	38.5	2534805	Mars N et al. Cell Genom (2022)
PGS002259	Stroke	stroke	534	50.2	268	60.5	323	Lu X et al. Neurology (2021)
PGS002262	Coronary artery disease	coronary artery disease	540	48.3	261	59.1	319	Lu X et al. Eur Heart J (2022)
PGS002316	Cardiovascular disease	cardiovascular disease	1109311	18.7	207730	39.5	437977	Weissbrod O et al. Nat Genet (2022)
PGS002361	Cardiovascular disease	cardiovascular disease	920929	19.2	177231	39.6	365125	Weissbrod O et al. Nat Genet (2022)
PGS002388	Cardiovascular disease	cardiovascular disease	5621	7.4	414	18	1013	Weissbrod O et al. Nat Genet (2022)
PGS002437	Cardiovascular disease	cardiovascular disease	20431	5.3	1088	13.9	2841	Weissbrod O et al. Nat Genet (2022)
PGS002486	Cardiovascular disease	cardiovascular disease	110640	3.9	4364	10.3	11421	Weissbrod O et al. Nat Genet (2022)
PGS002535	Cardiovascular disease	cardiovascular disease	1218	10.3	126	23.2	282	Weissbrod O et al. Nat Genet (2022)
PGS002584	Cardiovascular disease	cardiovascular disease	637	12.6	80	25.6	163	Weissbrod O et al. Nat Genet (2022)
PGS002633	Cardiovascular disease	cardiovascular disease	381036	8.8	33360	22.6	86182	Weissbrod O et al. Nat Genet (2022)
PGS002682	Cardiovascular disease	cardiovascular disease	972965	18.4	179186	39.4	383246	Weissbrod O et al. Nat Genet (2022)

PGS002724	Ischemic stroke	stroke,<br>Ischemic stroke	1213574	18	218543	38.5	466630	Mishra A et al. Nature (2022)
PGS002725	Ischemic stroke	stroke,<br>Ischemic stroke	6010730	15.9	954094	41.8	2510142	Mishra A et al. Nature (2022)
PGS002770	Stroke	stroke	1088719	18.6	202409	39.2	426574	Mars N et al. Am J Hum Genet (2022)
PGS002775	Incident coronary artery disease	coronary artery disease	1059	29.6	313	46.5	492	Wong CK et al. PLoS One (2022)
PGS002776	Incident coronary artery disease	coronary artery disease	390782	15.8	61738	34.8	135953	Wong CK et al. PLoS One (2022)
PGS002809	Coronary artery disease	coronary artery disease	205	36.6	75	47.8	98	Ahmed R et al. Int J Cardiol Heart Vasc (2022)
PGS003355	Coronary artery disease	coronary artery disease	1532758	10.6	162258	29.3	449805	Aragam KG et al. Nat Genet (2022)
PGS003356	Coronary artery disease	coronary artery disease	2324683	16	373095	38.4	893000	Aragam KG et al. Nat Genet (2022)
PGS003438	Coronary artery disease	coronary artery disease	241	22.4	54	35.3	85	Marston NA et al. JAMA Cardiol (2023)
PGS003446	Coronary artery disease	coronary artery disease	538084	8.8	47490	24.1	129619	Tcheandjieu C et al. Nat Med (2022)
PGS003725	Coronary artery disease	coronary artery disease	1296172	17.6	228063	37	479918	Patel AP et al. Nat Med (2023)
PGS003726	Coronary artery disease	coronary artery disease	1296172	17.6	228063	37	479918	Patel AP et al. Nat Med (2023)
PGS003727	Coronary artery disease	coronary artery disease	1125113	18.9	212115	39.5	444480	Patel AP et al. Nat Med (2023)
PGS003866	Coronary artery disease	coronary artery disease	10440	21.7	2269	41.8	4364	Shim I et al. Nature Communications (2023)
PGS003969	Heart failure	heart failure	39	17.9	7	35.9	14	Rasooly D et al. Nat Commun (2023)
PGS003984	Stroke	stroke	1121845	18.7	209873	39.4	441584	Monti R et al. Am J Hum Genet (2024)
PGS004000	Stroke	stroke	2371	20.8	494	40.5	961	Monti R et al. Am J Hum Genet (2024)
PGS004015	Stroke	stroke	65138	19.5	12724	40	26049	Monti R et al. Am J Hum Genet (2024)
PGS004026	Stroke	stroke	1011468	19	191826	39.5	399407	Monti R et al. Am J Hum Genet (2024)
PGS004041	Stroke	stroke	1011468	19	191826	39.5	399407	Monti R et al. Am J Hum Genet (2024)
PGS004054	Stroke	stroke	852173	19.3	164480	40.2	342714	Monti R et al. Am J Hum Genet (2024)
PGS004070	Stroke	stroke	852173	19.3	164480	40.2	342714	Monti R et al. Am J Hum Genet (2024)
PGS004084	Stroke	stroke	1091747	18.7	204685	39.4	430029	Monti R et al. Am J Hum Genet (2024)
PGS004098	Stroke	stroke	1091747	18.7	204685	39.4	430029	Monti R et al. Am J Hum Genet (2024)
PGS004108	Stroke	stroke	13	46.2	6	61.5	8	Monti R et al. Am J Hum Genet (2024)
PGS004124	Stroke	stroke	5808	21.7	1258	42.6	2474	Monti R et al. Am J Hum Genet (2024)
PGS004138	Stroke	stroke	888649	18.1	161144	38.5	342546	Monti R et al. Am J Hum Genet (2024)
PGS004154	Stroke	stroke	1116976	18.7	209218	39.4	439915	Monti R et al. Am J Hum Genet (2024)
PGS004196	Coronary artery disease	coronary artery disease	3892	16.1	628	32.9	1279	Raben TG et al. Sci Rep (2023)
PGS004197	Coronary artery disease	coronary artery disease	11490	14.7	1689	29.9	3437	Raben TG et al. Sci Rep (2023)
PGS004198	Coronary artery disease	coronary artery disease	5723	15.5	887	31.1	1782	Raben TG et al. Sci Rep (2023)
PGS004199	Coronary artery disease	coronary artery disease	6085	15.3	928	31	1886	Raben TG et al. Sci Rep (2023)

PGS004200	Coronary artery disease	coronary artery disease	8361	14.9	1244	30.3	2533	Raben TG et al. Sci Rep (2023)
PGS004237	Coronary Artery Disease	coronary artery disease	1146511	18.4	211397	39.3	450750	Manikpurage HD et al. Circ Genom Precis Med (2021)
PGS004238	Cardiovascular disease	cardiovascular disease	35	20	7	54.3	19	Cheng CF et al. J Gene Med (2021)
PGS004305	Coronary artery disease	coronary artery disease	3000	21	631	40.5	1215	Ohta R et al. Nat Commun (2024)
PGS004306	Coronary artery disease	coronary artery disease	4000	20.9	837	39.6	1585	Ohta R et al. Nat Commun (2024)
PGS004307	Coronary artery disease	coronary artery disease	4000	22	881	41.5	1661	Ohta R et al. Nat Commun (2024)
PGS004308	Coronary artery disease	coronary artery disease	1500	24	360	42.7	641	Ohta R et al. Nat Commun (2024)
PGS004309	Coronary artery disease	coronary artery disease	3000	22.9	688	42.2	1267	Ohta R et al. Nat Commun (2024)
PGS004321	Coronary heart disease	coronary artery disease	27	74.1	20	74.1	20	Marston NA et al. Circulation (2019)
PGS004322	Ischemic stroke	stroke, Ischemic stroke	30	30	9	53.3	16	McElligott B et al. Front Cardiovasc Med (2023)
PGS004443	Coronary artery disease (CAD)	coronary artery disease	1059939	18.6	197409	39.5	418909	Jung H et al. Commun Biol (2024)
PGS004444	Coronary vascular disease (CVD)	coronary artery disease	1059939	18.6	197409	39.5	418909	Jung H et al. Commun Biol (2024)
PGS004458	I21 (Acute myocardial infarction)	acute myocardial infarction	1059939	18.6	197409	39.5	418909	Jung H et al. Commun Biol (2024)
PGS004459	I25 (Chronic ischemic heart disease)	heart disease	1059939	18.6	197409	39.5	418909	Jung H et al. Commun Biol (2024)
PGS004462	I50 (Heart failure)	heart failure	1059939	18.6	197409	39.5	418909	Jung H et al. Commun Biol (2024)
PGS004513	Coronary artery disease (CAD)	coronary artery disease	1059939	18.6	197409	39.5	418909	Jung H et al. Commun Biol (2024)
PGS004514	Coronary vascular disease (CVD)	coronary artery disease	1059939	18.6	197409	39.5	418909	Jung H et al. Commun Biol (2024)
PGS004528	I21 (Acute myocardial infarction)	acute myocardial infarction	1059939	18.6	197409	39.5	418909	Jung H et al. Commun Biol (2024)
PGS004529	I25 (Chronic ischemic heart disease)	heart disease	1059939	18.6	197409	39.5	418909	Jung H et al. Commun Biol (2024)
PGS004532	I50 (Heart failure)	heart failure	1059939	18.6	197409	39.5	418909	Jung H et al. Commun Biol (2024)
PGS004595	Coronary heart disease	coronary artery disease	164	43.3	71	53.7	88	Oni-Orisan A et al. Clin Pharmacol Ther (2022)
PGS004596	Coronary heart disease	coronary artery disease	64	73.4	47	76.6	49	Peng H et al. Nutrients (2023)
PGS004597	Ischemic stroke	stroke, Ischemic stroke	32	28.1	9	53.1	17	Peng H et al. Nutrients (2023)
PGS004598	Heart failure	heart failure	12	33.3	4	41.7	5	Peng H et al. Nutrients (2023)
PGS004696	Coronary heart disease	coronary artery disease	1289980	17.6	226707	37	477059	Smith JL et al. Circ Genom Precis Med (2024)
PGS004697	Coronary heart disease	coronary artery disease	1120251	18.8	210636	39.4	441200	Smith JL et al. Circ Genom Precis Med (2024)
PGS004698	Coronary heart disease	coronary artery disease	542218	9	48617	24.3	131862	Smith JL et al. Circ Genom Precis Med (2024)
PGS004743	Coronary artery disease	coronary artery disease	3606321	13.4	481817	34.2	1233956	Truong B et al. Cell Genom (2024)
PGS004744	Coronary artery disease	coronary artery disease	7082943	13.7	971110	38.2	2705993	Truong B et al. Cell Genom (2024)
PGS004745	Coronary artery disease	coronary artery disease	4769577	10.5	501451	26.8	1279495	Truong B et al. Cell Genom (2024)
PGS004746	Coronary artery disease	coronary artery disease	6483064	14.6	943619	40.7	2637336	Truong B et al. Cell Genom (2024)
PGS004835	Stroke	stroke	2263784	11.6	262354	28.4	643388	Truong B et al. Cell Genom (2024)
PGS004836	Stroke	stroke	5644266	9.9	557874	24.9	1407712	Truong B et al. Cell Genom (2024)
PGS004879	Coronary heart disease	coronary artery disease	610677	17.5	107117	37.6	229639	Jermy B et al. Nat Commun (2024)
PGS004888	Coronary artery diseae	coronary artery disease	1110046	18.8	208953	39.4	437800	Mandla R et al. Genome Med (2024)
PGS004899	Spontaneous coronary artery dissection	spontaneous coronary artery dissection	7	28.6	2	57.1	4	Saw J et al. Nat Commun (2020)
PGS004919	Coronary artery disease	coronary artery disease	50	74	37	76	38	Sjögren M et al. Int J Cardiol Heart Vasc (2019)
PGS004921	Coronary artery disease	coronary artery disease	161	31.7	51	44.1	71	Huang Y et al. Circ Genom Precis Med (2020)
PGS004925	Coronary heart disease	coronary artery disease	300	25.7	77	40	120	Kim Y et al. J Intern Med (2023)

PGS004941	Coronary artery disease	coronary artery disease	3711629	16.8	623594	43.8	1625953	China Kadoorie Biobank Collaborative Group. et al. Nat Hum Behav (2024)
PGS005034	Myocardial infarction (PheCode 411.2)	myocardial infarction	1286612	17.5	225726	36.9	475197	Gunn S et al. HGG Adv (2024)
PGS005035	Myocardial infarction (PheCode 411.2)	myocardial infarction	1286612	17.5	225726	36.9	475197	Gunn S et al. HGG Adv (2024)
PGS005036	Myocardial infarction (PheCode 411.2)	myocardial infarction	1286612	17.5	225726	36.9	475197	Gunn S et al. HGG Adv (2024)
PGS005037	Myocardial infarction (PheCode 411.2)	myocardial infarction	1286612	17.5	225726	36.9	475197	Gunn S et al. HGG Adv (2024)
PGS005038	Myocardial infarction (PheCode 411.2)	myocardial infarction	1286612	17.5	225726	36.9	475197	Gunn S et al. HGG Adv (2024)
PGS005039	Myocardial infarction (PheCode 411.2)	myocardial infarction	1286612	17.5	225726	36.9	475197	Gunn S et al. HGG Adv (2024)
PGS005040	Myocardial infarction (PheCode 411.2)	myocardial infarction	1273897	17.5	223565	36.9	470532	Gunn S et al. HGG Adv (2024)
PGS005041	Myocardial infarction (PheCode 411.2)	myocardial infarction	1273897	17.5	223565	36.9	470532	Gunn S et al. HGG Adv (2024)
PGS005042	Myocardial infarction (PheCode 411.2)	myocardial infarction	1273897	17.5	223565	36.9	470532	Gunn S et al. HGG Adv (2024)
PGS005043	Myocardial infarction (PheCode 411.2)	myocardial infarction	1273897	17.5	223565	36.9	470532	Gunn S et al. HGG Adv (2024)
PGS005044	Myocardial infarction (PheCode 411.2)	myocardial infarction	1273897	17.5	223565	36.9	470532	Gunn S et al. HGG Adv (2024)
PGS005045	Myocardial infarction (PheCode 411.2)	myocardial infarction	1273897	17.5	223565	36.9	470532	Gunn S et al. HGG Adv (2024)
PGS005046	Myocardial infarction (PheCode 411.2)	myocardial infarction	1273891	17.5	223565	36.9	470531	Gunn S et al. HGG Adv (2024)
PGS000063	Triglycerides	triglyceride measurement	32	78.1	25	81.2	26	Johnson L et al. PLoS One (2015)
PGS000066	Triglycerides (TG)	triglyceride measurement	101	77.2	78	79.2	80	Kuchenbaecker K et al. Nat Commun (2019)
PGS000312	Triglycerides	triglyceride measurement	190	71.6	136	73.7	140	Xie T et al. Circ Genom Precis Med (2020)
PGS000659	Triglycerides	triglyceride measurement	142	23.9	34	46.5	66	Tam CHT et al. Genome Med (2021)
PGS000699	Triglycerides [mmol/L]	triglyceride measurement	16,003	18.3	2932	30.9	4952	Sinnott-Armstrong N et al. Nat Genet (2021)
PGS000826	Triglycerides	triglyceride measurement	769	30.7	236	47.5	365	Zubair N et al. Sci Rep (2019)
PGS000847	Triglycerides (TG)	triglyceride measurement	201	40.3	81	58.2	117	Aly DM et al. Nat Genet (2021)
PGS001979	Triglycerides	triglyceride measurement	71,203	19.7	14034	35.5	25281	Privé F et al. Am J Hum Genet (2022)
PGS002197	Triglycerides	triglyceride measurement	731,035	18.5	135001	38.9	284520	Privé F et al. Am J Hum Genet (2022)
PGS002287	Triglyceride	triglyceride measurement	286	14.4	41	31.2	89	Kamiza AB et al. Nat Med (2022)
PGS002353	Triglycerides	triglyceride measurement	1,109,311	18.7	207730	39.5	437977	Weissbrod O et al. Nat Genet (2022)
PGS002378	Triglycerides	triglyceride measurement	920,927	19.2	177228	39.6	365122	Weissbrod O et al. Nat Genet (2022)
PGS002425	Triglycerides	triglyceride measurement	10,135	6.8	691	16.6	1679	Weissbrod O et al. Nat Genet (2022)
PGS002474	Triglycerides	triglyceride measurement	27,130	5.3	1426	13.5	3651	Weissbrod O et al. Nat Genet (2022)
PGS002523	Triglycerides	triglyceride measurement	122,153	4	4889	10.3	12533	Weissbrod O et al. Nat Genet (2022)
PGS002572	Triglycerides	triglyceride measurement	3,917	8.8	344	19.4	760	Weissbrod O et al. Nat Genet (2022)
PGS002621	Triglycerides	triglyceride measurement	2,757	9.2	254	19.9	548	Weissbrod O et al. Nat Genet (2022)
PGS002670	Triglycerides	triglyceride measurement	379,622	8.4	31914	21.6	82015	Weissbrod O et al. Nat Genet (2022)
PGS002719	Triglycerides	triglyceride measurement	981,472	18.4	180689	39.4	386439	Weissbrod O et al. Nat Genet (2022)
PGS002784	Triglycerides	triglyceride measurement	30,071	7.7	2330	19	5711	Kanoni S et al. Genome Biol (2022)
PGS003144	Triglycerides	triglyceride measurement	762,608	14.6	111600	40.2	306490	Ma Y et al. Am J Hum Genet (2022)
PGS003145	Triglycerides	triglyceride measurement	9,693	9.5	918	24.5	2370	Ma Y et al. Am J Hum Genet (2022)
PGS003146	Triglycerides	triglyceride measurement	13,680	7.7	1049	19.1	2607	Ma Y et al. Am J Hum Genet (2022)
PGS003147	Triglycerides	triglyceride measurement	8,924,773	10.7	951700	29.8	2659235	Ma Y et al. Am J Hum Genet (2022)
PGS003148	Triglycerides	triglyceride measurement	1,113,831	18.8	209152	39.4	438880	Ma Y et al. Am J Hum Genet (2022)
PGS003149	Triglycerides	triglyceride measurement	705,677	15	106014	40.5	285868	Ma Y et al. Am J Hum Genet (2022)
PGS003150	Triglycerides	triglyceride measurement	5,113	10.7	549	25.6	1308	Ma Y et al. Am J Hum Genet (2022)
PGS003151	Triglycerides	triglyceride measurement	6,730	8.8	590	21	1411	Ma Y et al. Am J Hum Genet (2022)
PGS003152	Triglycerides	triglyceride measurement	8,924,752	10.7	951704	29.8	2659232	Ma Y et al. Am J Hum Genet (2022)
PGS003153	Triglycerides	triglyceride measurement	1,113,831	18.8	209152	39.4	438880	Ma Y et al. Am J Hum Genet (2022)
PGS003340	Triglyceride level	triglyceride measurement	65	15.4	10	24.6	16	Kim YJ et al. Nat Commun (2022)

PGS003349	Triglyceride level	triglyceride measurement	76	14.5	11	22.4	17 Kim YJ et al. Nat Commun (2022)
PGS003401	Triglyceride levels	triglyceride measurement	108	74.1	80	76.9	83 Pieri K et al. Int J Cardiol (2022)
PGS003482	Triglycerides	triglyceride measurement	842,513	19	160488	41.4	348986 Zhang Y et al. EBioMedicine (2022)
PGS003801	Log triglycerides	triglyceride measurement	33,687	23.2	7823	41.9	14126 Zhang H et al. Nat Genet (2023)
PGS003802	Log triglycerides	triglyceride measurement	1,492,288	20.9	312500	41.3	616757 Zhang H et al. Nat Genet (2023)
PGS003803	Log triglycerides	triglyceride measurement	116	31.9	37	44	51 Zhang H et al. Nat Genet (2023)
PGS003804	Log triglycerides	triglyceride measurement	1,678,784	18.5	309938	35.8	601073 Zhang H et al. Nat Genet (2023)
PGS003805	Log triglycerides	triglyceride measurement	1,678,784	18.5	309938	35.8	601073 Zhang H et al. Nat Genet (2023)
PGS003806	Log triglycerides	triglyceride measurement	1,155,382	18.7	216544	39.3	453660 Zhang H et al. Nat Genet (2023)
PGS003807	Log triglycerides	triglyceride measurement	1,106,476	18.5	204682	34.3	379285 Zhang H et al. Nat Genet (2023)
PGS003808	Log triglycerides	triglyceride measurement	109	37.6	41	58.7	64 Zhang H et al. Nat Genet (2023)
PGS003809	Log triglycerides	triglyceride measurement	1,189,464	22	262081	41.9	498450 Zhang H et al. Nat Genet (2023)
PGS003810	Log triglycerides	triglyceride measurement	1,189,464	22	262081	41.9	498450 Zhang H et al. Nat Genet (2023)
PGS003811	Log triglycerides	triglyceride measurement	1,155,382	18.7	216544	39.3	453660 Zhang H et al. Nat Genet (2023)
PGS003812	Log triglycerides	triglyceride measurement	563,207	25.8	145162	44.7	251514 Zhang H et al. Nat Genet (2023)
PGS003813	Log triglycerides	triglyceride measurement	40	47.5	19	65	26 Zhang H et al. Nat Genet (2023)
PGS003814	Log triglycerides	triglyceride measurement	1,507,338	21.3	321601	42	632895 Zhang H et al. Nat Genet (2023)
PGS003815	Log triglycerides	triglyceride measurement	1,507,338	21.3	321601	42	632895 Zhang H et al. Nat Genet (2023)
PGS003816	Log triglycerides	triglyceride measurement	1,155,382	18.7	216544	39.3	453660 Zhang H et al. Nat Genet (2023)
PGS003817	Log triglycerides	triglyceride measurement	798,330	23.1	184076	42.8	341719 Zhang H et al. Nat Genet (2023)
PGS003854	Triglycerides	triglyceride measurement	40	70	28	80	32 Li J et al. JAMA Netw Open (2023)
PGS003881	Triglyceride	triglyceride measurement	54,623	19.8	10806	39.9	21815 Shim I et al. Nature Communications (2023)
PGS004342	Triglycerides (mmol/L)	triglyceride measurement	1,059,939	18.6	197409	39.5	418909 Jung H et al. Commun Biol (2024)
PGS004652	Log triglycerides	triglyceride measurement	446	33.2	148	49.6	221 Zhang J et al. Nat Commun (2024)
PGS004653	Log triglycerides	triglyceride measurement	1,682,390	18.4	310383	35.8	601998 Zhang J et al. Nat Commun (2024)
PGS004654	Log triglycerides	triglyceride measurement	1,775,011	18.8	333602	36.6	649054 Zhang J et al. Nat Commun (2024)
PGS004655	Log triglycerides	triglyceride measurement	298,999	21.7	64871	41.8	125074 Zhang J et al. Nat Commun (2024)
PGS004656	Log triglycerides	triglyceride measurement	1,880,060	18.7	351952	36.4	683759 Zhang J et al. Nat Commun (2024)
PGS004657	Log triglycerides	triglyceride measurement	221	29.9	66	59.3	131 Zhang J et al. Nat Commun (2024)
PGS004658	Log triglycerides	triglyceride measurement	1,192,539	22	262500	41.9	499319 Zhang J et al. Nat Commun (2024)
PGS004659	Log triglycerides	triglyceride measurement	1,722,077	18.9	325766	36.8	633421 Zhang J et al. Nat Commun (2024)
PGS004660	Log triglycerides	triglyceride measurement	298,999	21.7	64871	41.8	125074 Zhang J et al. Nat Commun (2024)
PGS004661	Log triglycerides	triglyceride measurement	1,880,060	18.7	351952	36.4	683759 Zhang J et al. Nat Commun (2024)
PGS004662	Log triglycerides	triglyceride measurement	12,717	23	2929	43.9	5587 Zhang J et al. Nat Commun (2024)
PGS004663	Log triglycerides	triglyceride measurement	1,510,317	21.3	321977	42	633727 Zhang J et al. Nat Commun (2024)
PGS004664	Log triglycerides	triglyceride measurement	1,775,011	18.8	333602	36.6	649054 Zhang J et al. Nat Commun (2024)
PGS004665	Log triglycerides	triglyceride measurement	298,999	21.7	64871	41.8	125074 Zhang J et al. Nat Commun (2024)
PGS004666	Log triglycerides	triglyceride measurement	1,880,060	18.7	351952	36.4	683759 Zhang J et al. Nat Commun (2024)
PGS004845	Triglycerides	triglyceride measurement	1,095,976	14.3	157189	30.5	334383 Truong B et al. Cell Genom (2024)
PGS004846	Triglycerides	triglyceride measurement	6,248,300	14.6	911541	40.7	2546053 Truong B et al. Cell Genom (2024)
PGS004847	Triglycerides	triglyceride measurement	4,047,325	12.1	490870	30.8	1247013 Truong B et al. Cell Genom (2024)
PGS004848	Triglycerides	triglyceride measurement	6,127,766	14.8	906800	41.4	2534875 Truong B et al. Cell Genom (2024)
PGS004916	Triglycerides	triglyceride measurement	223	69.1	154	74	165 Trinder M et al. Arterioscler Thromb Vasc Biol (2019)
PGS004937	Triglycerides	triglyceride measurement	8,337	19.8	1649	31.6	2635 Moreno-Grau S et al. Human Genomics (2024)

PGS000192	Cholesterol	low density lipoprotein cholesterol measurement,<br>high density lipoprotein cholesterol measurement	9	66.7	6	66.7	6 Kathiresan S et al. N Engl J Med (2008)
PGS000062	Total cholesterol	total cholesterol measurement	52	76.9	40	76.9	40 Johnson L et al. PLoS One (2015)
PGS000311	Total cholesterol	total cholesterol measurement	234	68.8	161	72.6	170 Xie T et al. Circ Genom Precis Med (2020)
PGS000658	Total cholesterol	total cholesterol measurement	229	14.8	34	38.4	88 Tam CHT et al. Genome Med (2021)
PGS000677	Cholesterol [mmol/L] (statin adjusted)	total cholesterol measurement	17204	18.4	3158	31	5339 Sinnott-Armstrong N et al. Nat Genet (2021)
PGS000831	Total cholesterol	total cholesterol measurement	1032	30	310	47.2	487 Zubair N et al. Sci Rep (2019)
PGS001895	Cholesterol	total cholesterol measurement	16576	23	3805	42.3	7015 Privé F et al. Am J Hum Genet (2022)
PGS002108	Cholesterol	total cholesterol measurement	451160	18.7	84419	39	176039 Privé F et al. Am J Hum Genet (2022)
PGS002286	Total cholesterol	total cholesterol measurement	286	14.4	41	31.2	89 Kamiza AB et al. Nat Med (2022)
PGS002352	Total cholesterol	total cholesterol measurement	1109311	18.7	207730	39.5	437977 Weissbrod O et al. Nat Genet (2022)
PGS002377	Total cholesterol	total cholesterol measurement	920922	19.2	177229	39.6	365121 Weissbrod O et al. Nat Genet (2022)
PGS002424	Total cholesterol	total cholesterol measurement	8526	7.2	613	17.1	1459 Weissbrod O et al. Nat Genet (2022)
PGS002473	Total cholesterol	total cholesterol measurement	22547	5.5	1236	13.6	3066 Weissbrod O et al. Nat Genet (2022)
PGS002522	Total cholesterol	total cholesterol measurement	109026	4	4348	10	10891 Weissbrod O et al. Nat Genet (2022)
PGS002571	Total cholesterol	total cholesterol measurement	3573	8.7	312	19.9	711 Weissbrod O et al. Nat Genet (2022)
PGS002620	Total cholesterol	total cholesterol measurement	2571	9.6	248	20.9	537 Weissbrod O et al. Nat Genet (2022)
PGS002669	Total cholesterol	total cholesterol measurement	309475	8.2	25368	21.3	65996 Weissbrod O et al. Nat Genet (2022)
PGS002718	Total cholesterol	total cholesterol measurement	977049	18.4	179884	39.4	384772 Weissbrod O et al. Nat Genet (2022)
PGS002783	Total cholesterol	total cholesterol measurement	10699	9.9	1062	23.1	2468 Kanoni S et al. Genome Biol (2022)
PGS003134	Total cholesterol	total cholesterol measurement	585328	14.7	86019	40.6	237771 Ma Y et al. Am J Hum Genet (2022)
PGS003135	Total cholesterol	total cholesterol measurement	2822	12.3	348	28.1	794 Ma Y et al. Am J Hum Genet (2022)
PGS003136	Total cholesterol	total cholesterol measurement	3566	10.6	377	23.6	842 Ma Y et al. Am J Hum Genet (2022)
PGS003137	Total cholesterol	total cholesterol measurement	7459292	10.7	795185	29.8	2223385 Ma Y et al. Am J Hum Genet (2022)
PGS003138	Total cholesterol	total cholesterol measurement	1113831	18.8	209152	39.4	438880 Ma Y et al. Am J Hum Genet (2022)
PGS003139	Total cholesterol	total cholesterol measurement	569518	14.7	83494	40.5	230543 Ma Y et al. Am J Hum Genet (2022)
PGS003140	Total cholesterol	total cholesterol measurement	2863	12	343	27.5	786 Ma Y et al. Am J Hum Genet (2022)
PGS003141	Total cholesterol	total cholesterol measurement	3604	10.4	375	23.4	843 Ma Y et al. Am J Hum Genet (2022)
PGS003142	Total cholesterol	total cholesterol measurement	7459288	10.7	795184	29.8	2223383 Ma Y et al. Am J Hum Genet (2022)
PGS003143	Total cholesterol	total cholesterol measurement	1113831	18.8	209152	39.4	438880 Ma Y et al. Am J Hum Genet (2022)
PGS003341	Total cholesterol	total cholesterol measurement	91	6.6	6	19.8	18 Kim YJ et al. Nat Commun (2022)
PGS003350	Total cholesterol	total cholesterol measurement	102	7.8	8	19.6	20 Kim YJ et al. Nat Commun (2022)
PGS003481	Total cholesterol	total cholesterol measurement	842513	19	160488	41.4	348986 Zhang Y et al. EBioMedicine (2022)
PGS003495	Cholesterol	total cholesterol measurement	979739	19.1	187570	40.9	400815 Ding Y et al. bioRxiv (2022) | Pre
PGS003818	Total cholesterol	total cholesterol measurement	4691	25.3	1188	44.1	2069 Zhang H et al. Nat Genet (2023)
PGS003819	Total cholesterol	total cholesterol measurement	1490151	20.9	312042	41.4	616586 Zhang H et al. Nat Genet (2023)
PGS003820	Total cholesterol	total cholesterol measurement	218	33	72	47.7	104 Zhang H et al. Nat Genet (2023)
PGS003821	Total cholesterol	total cholesterol measurement	1687775	18.5	312445	35.8	604558 Zhang H et al. Nat Genet (2023)
PGS003822	Total cholesterol	total cholesterol measurement	1687775	18.5	312445	35.8	604558 Zhang H et al. Nat Genet (2023)
PGS003823	Total cholesterol	total cholesterol measurement	1155228	18.7	216507	39.3	453624 Zhang H et al. Nat Genet (2023)
PGS003824	Total cholesterol	total cholesterol measurement	1122865	18.5	208119	34.3	385364 Zhang H et al. Nat Genet (2023)
PGS003825	Total cholesterol	total cholesterol measurement	230	26.1	60	50	115 Zhang H et al. Nat Genet (2023)
PGS003826	Total cholesterol	total cholesterol measurement	820996	20.4	167492	41.6	341840 Zhang H et al. Nat Genet (2023)
PGS003827	Total cholesterol	total cholesterol measurement	820996	20.4	167492	41.6	341840 Zhang H et al. Nat Genet (2023)
PGS003828	Total cholesterol	total cholesterol measurement	1155228	18.7	216507	39.3	453624 Zhang H et al. Nat Genet (2023)
PGS003829	Total cholesterol	total cholesterol measurement	431780	24	103821	44.7	193018 Zhang H et al. Nat Genet (2023)

PGS003830	Total cholesterol	total cholesterol measurement	32	43.8	14	62.5	20 Zhang H et al. Nat Genet (2023)
PGS003831	Total cholesterol	total cholesterol measurement	1468958	21.3	312297	42.2	620409 Zhang H et al. Nat Genet (2023)
PGS003832	Total cholesterol	total cholesterol measurement	1468958	21.3	312297	42.2	620409 Zhang H et al. Nat Genet (2023)
PGS003833	Total cholesterol	total cholesterol measurement	1155228	18.7	216507	39.3	453624 Zhang H et al. Nat Genet (2023)
PGS003834	Total cholesterol	total cholesterol measurement	770490	22.9	176770	43.2	332745 Zhang H et al. Nat Genet (2023)
PGS003853	Total cholesterol	total cholesterol measurement	60	63.3	38	68.3	41 Li J et al. JAMA Netw Open (2023)
PGS004333	Cholesterol (mmol/L)	total cholesterol measurement	1059939	18.6	197409	39.5	418909 Jung H et al. Commun Biol (2024)
PGS004667	Total cholesterol	total cholesterol measurement	1830	25.4	465	40.9	749 Zhang J et al. Nat Commun (2024)
PGS004668	Total cholesterol	total cholesterol measurement	1691065	18.5	312789	35.8	605327 Zhang J et al. Nat Commun (2024)
PGS004669	Total cholesterol	total cholesterol measurement	1728954	19	328443	37	640273 Zhang J et al. Nat Commun (2024)
PGS004670	Total cholesterol	total cholesterol measurement	158252	22.1	35044	42.3	66912 Zhang J et al. Nat Commun (2024)
PGS004671	Total cholesterol	total cholesterol measurement	1872229	18.7	349919	36.4	681361 Zhang J et al. Nat Commun (2024)
PGS004672	Total cholesterol	total cholesterol measurement	65005	21.4	13902	42	27289 Zhang J et al. Nat Commun (2024)
PGS004673	Total cholesterol	total cholesterol measurement	823148	20.4	167741	41.6	342424 Zhang J et al. Nat Commun (2024)
PGS004674	Total cholesterol	total cholesterol measurement	1728954	19	328443	37	640273 Zhang J et al. Nat Commun (2024)
PGS004675	Total cholesterol	total cholesterol measurement	158252	22.1	35044	42.3	66912 Zhang J et al. Nat Commun (2024)
PGS004676	Total cholesterol	total cholesterol measurement	1872229	18.7	349919	36.4	681361 Zhang J et al. Nat Commun (2024)
PGS004677	Total cholesterol	total cholesterol measurement	2421	25.5	617	44.9	1086 Zhang J et al. Nat Commun (2024)
PGS004678	Total cholesterol	total cholesterol measurement	1471975	21.2	312710	42.2	621295 Zhang J et al. Nat Commun (2024)
PGS004679	Total cholesterol	total cholesterol measurement	1728954	19	328443	37	640273 Zhang J et al. Nat Commun (2024)
PGS004680	Total cholesterol	total cholesterol measurement	158252	22.1	35044	42.3	66912 Zhang J et al. Nat Commun (2024)
PGS004681	Total cholesterol	total cholesterol measurement	1872229	18.7	349919	36.4	681361 Zhang J et al. Nat Commun (2024)
PGS004841	Total cholesterol	total cholesterol measurement	1051726	14.6	153815	31.1	326640 Truong B et al. Cell Genom (2024)
PGS004842	Total cholesterol	total cholesterol measurement	6439132	14.3	918913	39.7	2554448 Truong B et al. Cell Genom (2024)
PGS004843	Total cholesterol	total cholesterol measurement	4136795	12.1	500690	30.9	1276490 Truong B et al. Cell Genom (2024)
PGS000060	HDL cholesterol	low density lipoprotein cholesterol measurement	46	80.4	37	84.8	39 Johnson L et al. PLoS One (2015)
PGS000064	HDL cholesterol	low density lipoprotein cholesterol measurement	120	72.5	87	75	90 Kuchenbaecker K et al. Nat Commun (2019)
PGS000309	HDL cholesterol	low density lipoprotein cholesterol measurement	247	67.6	167	70.9	175 Xie T et al. Circ Genom Precis Med (2020)
PGS000660	HDL cholesterol	low density lipoprotein cholesterol measurement	549	19.3	106	43.9	241 Tam CHT et al. Genome Med (2021)
PGS000686	HDL cholesterol [mmol/L]	low density lipoprotein cholesterol measurement	25069	18.1	4549	31.4	7867 Sinnott-Armstrong N et al. Nat Genet (2021)
PGS000825	HDL cholesterol	low density lipoprotein cholesterol measurement	883	31.1	275	47.2	417 Zubair N et al. Sci Rep (2019)
PGS000845	HDL cholesterol	low density lipoprotein cholesterol measurement	303	35.6	108	55.1	167 Aly DM et al. Nat Genet (2021)
PGS001954	HDL cholesterol	low density lipoprotein cholesterol measurement	85429	20	17056	36.4	31109 Privé F et al. Am J Hum Genet (2022)
PGS002172	HDL cholesterol	low density lipoprotein cholesterol measurement	732902	18.4	135154	38.9	284816 Privé F et al. Am J Hum Genet (2022)
PGS002284	HDL cholesterol	low density lipoprotein cholesterol measurement	286	14.4	41	31.2	89 Kamiza AB et al. Nat Med (2022)
PGS002329	HDL cholesterol	low density lipoprotein cholesterol measurement	1109311	18.7	207730	39.5	437977 Weissbrod O et al. Nat Genet (2022)
PGS002366	HDL cholesterol	low density lipoprotein cholesterol measurement	920924	19.2	177231	39.6	365122 Weissbrod O et al. Nat Genet (2022)

PGS002401	HDL cholesterol	low density lipoprotein cholesterol measurement	15141	6.4	973	16.6	2515	Weissbrod O et al. Nat Genet (2022)
PGS002450	HDL cholesterol	low density lipoprotein cholesterol measurement	35979	5.4	1942	14.1	5056	Weissbrod O et al. Nat Genet (2022)
PGS002499	HDL cholesterol	low density lipoprotein cholesterol measurement	141710	4.2	5904	10.8	15288	Weissbrod O et al. Nat Genet (2022)
PGS002548	HDL cholesterol	low density lipoprotein cholesterol measurement	6518	7.6	495	18.4	1200	Weissbrod O et al. Nat Genet (2022)
PGS002597	HDL cholesterol	low density lipoprotein cholesterol measurement	4738	8.1	386	18.9	896	Weissbrod O et al. Nat Genet (2022)
PGS002646	HDL cholesterol	low density lipoprotein cholesterol measurement	402183	8.6	34412	22.5	90359	Weissbrod O et al. Nat Genet (2022)
PGS002695	HDL cholesterol	low density lipoprotein cholesterol measurement	971682	18.4	179011	39.4	382980	Weissbrod O et al. Nat Genet (2022)
PGS002781	HDL cholesterol	low density lipoprotein cholesterol measurement	1239184	17.7	219255	37.2	460888	Kanoni S et al. Genome Biol (2022)
PGS002954	HDL cholesterol	low density lipoprotein cholesterol measurement	815573	14.8	120354	40.9	333690	Ma Y et al. Am J Hum Genet (2022)
PGS002955	HDL cholesterol	low density lipoprotein cholesterol measurement	16393	9.1	1485	24	3934	Ma Y et al. Am J Hum Genet (2022)
PGS002956	HDL cholesterol	low density lipoprotein cholesterol measurement	25134	6.8	1707	17.4	4366	Ma Y et al. Am J Hum Genet (2022)
PGS002957	HDL cholesterol	low density lipoprotein cholesterol measurement	7449065	10.7	793385	29.8	2218069	Ma Y et al. Am J Hum Genet (2022)
PGS002958	HDL cholesterol	low density lipoprotein cholesterol measurement	1113830	18.8	209152	39.4	438879	Ma Y et al. Am J Hum Genet (2022)
PGS002959	HDL cholesterol	low density lipoprotein cholesterol measurement	799981	14.8	118555	41	327851	Ma Y et al. Am J Hum Genet (2022)
PGS002960	HDL cholesterol	low density lipoprotein cholesterol measurement	17935	9	1609	23.8	4265	Ma Y et al. Am J Hum Genet (2022)
PGS002961	HDL cholesterol	low density lipoprotein cholesterol measurement	28354	6.6	1869	16.9	4803	Ma Y et al. Am J Hum Genet (2022)
PGS002962	HDL cholesterol	low density lipoprotein cholesterol measurement	7449036	10.7	793379	29.8	2218052	Ma Y et al. Am J Hum Genet (2022)
PGS002963	HDL cholesterol	low density lipoprotein cholesterol measurement	1113830	18.8	209152	39.4	438879	Ma Y et al. Am J Hum Genet (2022)
PGS003338	HDL cholesterol	low density lipoprotein cholesterol measurement	79	19	15	35.4	28	Kim YJ et al. Nat Commun (2022)
PGS003347	HDL cholesterol	low density lipoprotein cholesterol measurement	92	25	23	39.1	36	Kim YJ et al. Nat Commun (2022)
PGS003534	HDL cholesterol	low density lipoprotein cholesterol measurement	979739	19.1	187570	40.9	400815	Ding Y et al. bioRxiv (2022) | Pre
PGS003767	HDL cholesterol	low density lipoprotein cholesterol measurement	12656	23.5	2980	42.5	5373	Zhang H et al. Nat Genet (2023)
PGS003768	HDL cholesterol	low density lipoprotein cholesterol measurement	1489018	20.9	311586	41.4	615736	Zhang H et al. Nat Genet (2023)
PGS003769	HDL cholesterol	low density lipoprotein cholesterol measurement	198	28.3	56	44.4	88	Zhang H et al. Nat Genet (2023)
PGS003770	HDL cholesterol	low density lipoprotein cholesterol measurement	1678653	18.5	309941	35.8	600866	Zhang H et al. Nat Genet (2023)

PGS003771	HDL cholesterol	low density lipoprotein cholesterol measurement	1678653	18.5	309941	35.8	600866	Zhang H et al. Nat Genet (2023)
PGS003772	HDL cholesterol	low density lipoprotein cholesterol measurement	1155488	18.7	216574	39.3	453701	Zhang H et al. Nat Genet (2023)
PGS003773	HDL cholesterol	low density lipoprotein cholesterol measurement	1102319	18.5	204005	34.3	377839	Zhang H et al. Nat Genet (2023)
PGS003774	HDL cholesterol	low density lipoprotein cholesterol measurement	117	17.9	21	43.6	51	Zhang H et al. Nat Genet (2023)
PGS003775	HDL cholesterol	low density lipoprotein cholesterol measurement	844599	20.2	170852	41	346029	Zhang H et al. Nat Genet (2023)
PGS003776	HDL cholesterol	low density lipoprotein cholesterol measurement	844599	20.2	170852	41	346029	Zhang H et al. Nat Genet (2023)
PGS003777	HDL cholesterol	low density lipoprotein cholesterol measurement	1155488	18.7	216574	39.3	453701	Zhang H et al. Nat Genet (2023)
PGS003778	HDL cholesterol	low density lipoprotein cholesterol measurement	449110	23.8	107024	43.8	196500	Zhang H et al. Nat Genet (2023)
PGS003779	HDL cholesterol	low density lipoprotein cholesterol measurement	71	38	27	56.3	40	Zhang H et al. Nat Genet (2023)
PGS003780	HDL cholesterol	low density lipoprotein cholesterol measurement	1473121	21.3	313403	42.2	621802	Zhang H et al. Nat Genet (2023)
PGS003781	HDL cholesterol	low density lipoprotein cholesterol measurement	1473121	21.3	313403	42.2	621802	Zhang H et al. Nat Genet (2023)
PGS003782	HDL cholesterol	low density lipoprotein cholesterol measurement	1155488	18.7	216574	39.3	453701	Zhang H et al. Nat Genet (2023)
PGS003783	HDL cholesterol	low density lipoprotein cholesterol measurement	771693	23	177311	43.2	333174	Zhang H et al. Nat Genet (2023)
PGS003856	HDL cholesterol	low density lipoprotein cholesterol measurement	59	66.1	39	71.2	42	Li J et al. JAMA Netw Open (2023)
PGS003875	HDL cholesterol	low density lipoprotein cholesterol measurement	1022259	18.6	190050	38.9	397405	Shim I et al. Nature Communications (2023)
PGS003876	HDL cholesterol	low density lipoprotein cholesterol measurement	1067858	18.7	199462	39.4	421163	Shim I et al. Nature Communications (2023)
PGS003877	HDL cholesterol	low density lipoprotein cholesterol measurement	882001	18.3	161331	38.2	336606	Shim I et al. Nature Communications (2023)
PGS003878	HDL cholesterol	low density lipoprotein cholesterol measurement	958649	18.7	179164	38.1	365376	Shim I et al. Nature Communications (2023)
PGS003879	HDL cholesterol	low density lipoprotein cholesterol measurement	1069677	18.7	199910	39.5	422268	Shim I et al. Nature Communications (2023)
PGS003880	HDL cholesterol	low density lipoprotein cholesterol measurement	1054648	18.7	197363	39.5	416166	Shim I et al. Nature Communications (2023)
PGS003986	HDL cholesterol	low density lipoprotein cholesterol measurement	1138429	18.7	212817	39.3	447702	Monti R et al. Am J Hum Genet (2024)
PGS004002	HDL cholesterol	low density lipoprotein cholesterol measurement	7708	22.4	1730	41.4	3194	Monti R et al. Am J Hum Genet (2024)
PGS004028	HDL cholesterol	low density lipoprotein cholesterol measurement	992696	18.7	185504	39.6	393530	Monti R et al. Am J Hum Genet (2024)
PGS004043	HDL cholesterol	low density lipoprotein cholesterol measurement	578264	18.5	106926	39.2	226715	Monti R et al. Am J Hum Genet (2024)
PGS004056	HDL cholesterol	low density lipoprotein cholesterol measurement	791965	19.5	154092	40.4	320166	Monti R et al. Am J Hum Genet (2024)

PGS004072	HDL cholesterol	low density lipoprotein cholesterol measurement	791965	19.5	154092	40.4	320166	Monti R et al. Am J Hum Genet (2024)
PGS004086	HDL cholesterol	low density lipoprotein cholesterol measurement	1103534	18.7	206707	39.3	433756	Monti R et al. Am J Hum Genet (2024)
PGS004100	HDL cholesterol	low density lipoprotein cholesterol measurement	1103534	18.7	206707	39.3	433756	Monti R et al. Am J Hum Genet (2024)
PGS004110	HDL cholesterol	low density lipoprotein cholesterol measurement	161	40.4	65	62.1	100	Monti R et al. Am J Hum Genet (2024)
PGS004126	HDL cholesterol	low density lipoprotein cholesterol measurement	579	27.5	159	47.3	274	Monti R et al. Am J Hum Genet (2024)
PGS004140	HDL cholesterol	low density lipoprotein cholesterol measurement	875144	18.3	159739	39.7	347188	Monti R et al. Am J Hum Genet (2024)
PGS004156	HDL cholesterol	low density lipoprotein cholesterol measurement	1138452	18.7	212823	39.3	447712	Monti R et al. Am J Hum Genet (2024)
PGS004622	HDL cholesterol	low density lipoprotein cholesterol measurement	15978	19.7	3143	36.9	5890	Zhang J et al. Nat Commun (2024)
PGS004623	HDL cholesterol	low density lipoprotein cholesterol measurement	1683003	18.5	310571	35.8	602203	Zhang J et al. Nat Commun (2024)
PGS004624	HDL cholesterol	low density lipoprotein cholesterol measurement	1688569	19.1	322385	37.2	628355	Zhang J et al. Nat Commun (2024)
PGS004625	HDL cholesterol	low density lipoprotein cholesterol measurement	291154	21.1	61506	41.4	120530	Zhang J et al. Nat Commun (2024)
PGS004626	HDL cholesterol	low density lipoprotein cholesterol measurement	1871796	18.7	349888	36.4	681251	Zhang J et al. Nat Commun (2024)
PGS004627	HDL cholesterol	low density lipoprotein cholesterol measurement	54812	20.7	11373	41.2	22572	Zhang J et al. Nat Commun (2024)
PGS004628	HDL cholesterol	low density lipoprotein cholesterol measurement	847389	20.2	171245	40.9	346985	Zhang J et al. Nat Commun (2024)
PGS004629	HDL cholesterol	low density lipoprotein cholesterol measurement	1731178	19	328457	37	640149	Zhang J et al. Nat Commun (2024)
PGS004630	HDL cholesterol	low density lipoprotein cholesterol measurement	291154	21.1	61506	41.4	120530	Zhang J et al. Nat Commun (2024)
PGS004631	HDL cholesterol	low density lipoprotein cholesterol measurement	1871796	18.7	349888	36.4	681251	Zhang J et al. Nat Commun (2024)
PGS004632	HDL cholesterol	low density lipoprotein cholesterol measurement	9116	23.3	2123	45.2	4120	Zhang J et al. Nat Commun (2024)
PGS004633	HDL cholesterol	low density lipoprotein cholesterol measurement	1476939	21.3	314067	42.2	623233	Zhang J et al. Nat Commun (2024)
PGS004634	HDL cholesterol	low density lipoprotein cholesterol measurement	1731178	19	328457	37	640149	Zhang J et al. Nat Commun (2024)
PGS004635	HDL cholesterol	low density lipoprotein cholesterol measurement	291154	21.1	61506	41.4	120530	Zhang J et al. Nat Commun (2024)
PGS004636	HDL cholesterol	low density lipoprotein cholesterol measurement	1871796	18.7	349888	36.4	681251	Zhang J et al. Nat Commun (2024)
PGS004775	HDL cholesterol	low density lipoprotein cholesterol measurement	1120830	14.2	158702	30.3	339159	Truong B et al. Cell Genom (2024)
PGS004776	HDL cholesterol	low density lipoprotein cholesterol measurement	6463324	14.3	923755	39.8	2574884	Truong B et al. Cell Genom (2024)
PGS004777	HDL cholesterol	low density lipoprotein cholesterol measurement	4547847	11.3	515522	28.9	1314415	Truong B et al. Cell Genom (2024)

PGS004778	HDL cholesterol	low density lipoprotein cholesterol measurement	6463324	14.3	923755	39.8	2574884	Truong B et al. Cell Genom (2024)
PGS004914	HDL cholesterol	low density lipoprotein cholesterol measurement	223	69.1	154	74	165	Trinder M et al. Arterioscler Thromb Vasc Biol (2019)
PGS004932	HDL cholesterol	low density lipoprotein cholesterol measurement	17151	18.8	3217	31.9	5464	Moreno-Grau S et al. Human Genomics (2024)
PGS000061	Low-density lipoprotein (LDL) cholesterol	low density lipoprotein cholesterol measurement	37	73	27	73	27	Johnson L et al. PLoS One (2015)
PGS000065	LDL cholesterol	low density lipoprotein cholesterol measurement	103	69.9	72	73.8	76	Kuchenbaecker K et al. Nat Commun (2019)
PGS000115	LDL cholesterol	low density lipoprotein cholesterol measurement	223	69.1	154	74	165	Trinder M et al. JAMA Cardiol (2020)
PGS000310	LDL cholesterol	low density lipoprotein cholesterol measurement	194	68.6	133	72.7	141	Xie T et al. Circ Genom Precis Med (2020)
PGS000340	LDL cholesterol	low density lipoprotein cholesterol measurement	28	85.7	24	85.7	24	Trinder M et al. Circ Genom Precis Med (2020)
PGS000661	LDL cholesterol	low density lipoprotein cholesterol measurement	84	14.3	12	46.4	39	Tam CHT et al. Genome Med (2021)
PGS000688	LDL cholesterol [mmol/L] (statin adjusted)	low density lipoprotein cholesterol measurement	16184	18.9	3053	31.4	5080	Sinnott-Armstrong N et al. Nat Genet (2021)
PGS000814	LDL cholesterol	low density lipoprotein cholesterol measurement	12	100	12	100	12	Talmud PJ et al. Lancet (2013)
PGS000824	LDL cholesterol	low density lipoprotein cholesterol measurement	809	29	235	47.3	383	Zubair N et al. Sci Rep (2019)
PGS000846	LDL cholesterol	low density lipoprotein cholesterol measurement	275	41.1	113	53.5	147	Aly DM et al. Nat Genet (2021)
PGS000875	LDL cholesterol	low density lipoprotein cholesterol measurement	36	55.6	20	61.1	22	Leal LG et al. Mol Genet Genomic Med (2020)
PGS000886	LDL cholesterol	low density lipoprotein cholesterol measurement	1222318	17.4	212812	36.2	442528	Graham SE et al. Nature (2021)
PGS000887	LDL cholesterol	low density lipoprotein cholesterol measurement	295	12.9	38	23.1	68	Graham SE et al. Nature (2021)
PGS000888	LDL cholesterol	low density lipoprotein cholesterol measurement	1239184	17.7	219255	37.2	460888	Graham SE et al. Nature (2021)
PGS000889	LDL cholesterol	low density lipoprotein cholesterol measurement	9009	9.5	860	21.3	1916	Graham SE et al. Nature (2021)
PGS000890	LDL cholesterol	low density lipoprotein cholesterol measurement	1029158	19.4	200135	39.4	405389	Graham SE et al. Nature (2021)
PGS000891	LDL cholesterol	low density lipoprotein cholesterol measurement	66	18.2	12	36.4	24	Graham SE et al. Nature (2021)
PGS000892	LDL cholesterol	low density lipoprotein cholesterol measurement	1119211	18.8	210397	39.4	440725	Graham SE et al. Nature (2021)
PGS000893	LDL cholesterol	low density lipoprotein cholesterol measurement	5427	9.8	534	21.5	1165	Graham SE et al. Nature (2021)
PGS000894	LDL cholesterol	low density lipoprotein cholesterol measurement	1175595	18.3	215232	38.5	452085	Graham SE et al. Nature (2021)
PGS000895	LDL cholesterol	low density lipoprotein cholesterol measurement	76	25	19	40.8	31	Graham SE et al. Nature (2021)
PGS000896	LDL cholesterol	low density lipoprotein cholesterol measurement	1100062	19.1	210020	40	440174	Graham SE et al. Nature (2021)

PGS000897	LDL cholesterol	low density lipoprotein cholesterol measurement	13	46.2	6	46.2	6	Graham SE et al. Nature (2021)
PGS001933	LDL direct	low density lipoprotein cholesterol measurement	25604	21.9	5609	42	10754	Privé F et al. Am J Hum Genet (2022)
PGS002150	LDL direct	low density lipoprotein cholesterol measurement	360007	19.1	68589	39.3	141452	Privé F et al. Am J Hum Genet (2022)
PGS002274	LDL cholesterol	low density lipoprotein cholesterol measurement	279	66.3	185	71	198	Groenland EH et al. Atherosclerosis (2022)
PGS002285	LDL cholesterol	low density lipoprotein cholesterol measurement	286	14.4	41	31.2	89	Kamiza AB et al. Nat Med (2022)
PGS002337	LDL cholesterol	low density lipoprotein cholesterol measurement	1109311	18.7	207730	39.5	437977	Weissbrod O et al. Nat Genet (2022)
PGS002369	LDL cholesterol	low density lipoprotein cholesterol measurement	920930	19.2	177232	39.6	365127	Weissbrod O et al. Nat Genet (2022)
PGS002409	LDL cholesterol	low density lipoprotein cholesterol measurement	7626	7	534	16.6	1267	Weissbrod O et al. Nat Genet (2022)
PGS002458	LDL cholesterol	low density lipoprotein cholesterol measurement	20708	5.3	1098	13.4	2770	Weissbrod O et al. Nat Genet (2022)
PGS002507	LDL cholesterol	low density lipoprotein cholesterol measurement	105053	3.9	4133	9.8	10314	Weissbrod O et al. Nat Genet (2022)
PGS002556	LDL cholesterol	low density lipoprotein cholesterol measurement	3244	9.1	296	19	615	Weissbrod O et al. Nat Genet (2022)
PGS002605	LDL cholesterol	low density lipoprotein cholesterol measurement	2337	10	233	20.4	477	Weissbrod O et al. Nat Genet (2022)
PGS002654	LDL cholesterol	low density lipoprotein cholesterol measurement	274585	8.3	22673	21.4	58654	Weissbrod O et al. Nat Genet (2022)
PGS002703	LDL cholesterol	low density lipoprotein cholesterol measurement	970081	18.4	178621	39.4	382246	Weissbrod O et al. Nat Genet (2022)
PGS003029	LDL cholesterol	low density lipoprotein cholesterol measurement	549112	15	82236	40.6	222805	Ma Y et al. Am J Hum Genet (2022)
PGS003030	LDL cholesterol	low density lipoprotein cholesterol measurement	2066	13.1	271	27.4	566	Ma Y et al. Am J Hum Genet (2022)
PGS003031	LDL cholesterol	low density lipoprotein cholesterol measurement	2609	10.8	282	23	599	Ma Y et al. Am J Hum Genet (2022)
PGS003032	LDL cholesterol	low density lipoprotein cholesterol measurement	7457930	10.7	795043	29.8	2222418	Ma Y et al. Am J Hum Genet (2022)
PGS003033	LDL cholesterol	low density lipoprotein cholesterol measurement	1113830	18.8	209152	39.4	438879	Ma Y et al. Am J Hum Genet (2022)
PGS003034	LDL cholesterol	low density lipoprotein cholesterol measurement	552845	15	83123	40.5	223870	Ma Y et al. Am J Hum Genet (2022)
PGS003035	LDL cholesterol	low density lipoprotein cholesterol measurement	2288	12.2	280	27.1	621	Ma Y et al. Am J Hum Genet (2022)
PGS003036	LDL cholesterol	low density lipoprotein cholesterol measurement	2935	10.3	302	22.6	664	Ma Y et al. Am J Hum Genet (2022)
PGS003037	LDL cholesterol	low density lipoprotein cholesterol measurement	8918470	10.7	951138	29.8	2657026	Ma Y et al. Am J Hum Genet (2022)
PGS003038	LDL cholesterol	low density lipoprotein cholesterol measurement	1113830	18.8	209152	39.4	438879	Ma Y et al. Am J Hum Genet (2022)
PGS003339	LDL cholesterol	low density lipoprotein cholesterol measurement	65	16.9	11	36.9	24	Kim YJ et al. Nat Commun (2022)

PGS003348	LDL cholesterol	low density lipoprotein cholesterol measurement	78	24.4	19	41	32	Kim YJ et al. Nat Commun (2022)
PGS003403	LDL cholesterol	low density lipoprotein cholesterol measurement	28	85.7	24	85.7	24	Trinder M et al. J Am Coll Cardiol (2019)
PGS003404	LDL cholesterol	low density lipoprotein cholesterol measurement	10	90	9	90	9	Wang J et al. Arterioscler Thromb Vasc Biol (2016)
PGS003405	LDL cholesterol	low density lipoprotein cholesterol measurement	169	37.9	64	42	71	Vanhoye X et al. Transl Res (2022)
PGS003474	LDL levels	low density lipoprotein cholesterol measurement	842513	19	160488	41.4	348986	Zhang Y et al. EBioMedicine (2022)
PGS003517	LDL direct	low density lipoprotein cholesterol measurement	979739	19.1	187570	40.9	400815	Ding Y et al. bioRxiv (2022) | Pre
PGS003784	LDL cholesterol	low density lipoprotein cholesterol measurement	3754	26.4	992	44.6	1674	Zhang H et al. Nat Genet (2023)
PGS003785	LDL cholesterol	low density lipoprotein cholesterol measurement	1490736	20.9	312060	41.4	616423	Zhang H et al. Nat Genet (2023)
PGS003786	LDL cholesterol	low density lipoprotein cholesterol measurement	188	29.8	56	45.7	86	Zhang H et al. Nat Genet (2023)
PGS003787	LDL cholesterol	low density lipoprotein cholesterol measurement	1679610	18.5	310161	35.8	601376	Zhang H et al. Nat Genet (2023)
PGS003788	LDL cholesterol	low density lipoprotein cholesterol measurement	1679610	18.5	310161	35.8	601376	Zhang H et al. Nat Genet (2023)
PGS003789	LDL cholesterol	low density lipoprotein cholesterol measurement	1155363	18.7	216544	39.3	453660	Zhang H et al. Nat Genet (2023)
PGS003790	LDL cholesterol	low density lipoprotein cholesterol measurement	1120053	18.5	207104	34.3	384558	Zhang H et al. Nat Genet (2023)
PGS003791	LDL cholesterol	low density lipoprotein cholesterol measurement	80	31.2	25	50	40	Zhang H et al. Nat Genet (2023)
PGS003792	LDL cholesterol	low density lipoprotein cholesterol measurement	1191112	22	262392	41.9	498918	Zhang H et al. Nat Genet (2023)
PGS003793	LDL cholesterol	low density lipoprotein cholesterol measurement	1191112	22	262392	41.9	498918	Zhang H et al. Nat Genet (2023)
PGS003794	LDL cholesterol	low density lipoprotein cholesterol measurement	1155363	18.7	216544	39.3	453660	Zhang H et al. Nat Genet (2023)
PGS003795	LDL cholesterol	low density lipoprotein cholesterol measurement	564379	25.7	145157	44.6	251716	Zhang H et al. Nat Genet (2023)
PGS003796	LDL cholesterol	low density lipoprotein cholesterol measurement	25	44	11	64	16	Zhang H et al. Nat Genet (2023)
PGS003797	LDL cholesterol	low density lipoprotein cholesterol measurement	1507815	21.3	321649	42	632935	Zhang H et al. Nat Genet (2023)
PGS003798	LDL cholesterol	low density lipoprotein cholesterol measurement	1507815	21.3	321649	42	632935	Zhang H et al. Nat Genet (2023)
PGS003799	LDL cholesterol	low density lipoprotein cholesterol measurement	1155363	18.7	216544	39.3	453660	Zhang H et al. Nat Genet (2023)
PGS003800	LDL cholesterol	low density lipoprotein cholesterol measurement	801576	23	184663	42.8	342880	Zhang H et al. Nat Genet (2023)
PGS003855	LDL cholesterol	low density lipoprotein cholesterol measurement	44	56.8	25	65.9	29	Li J et al. JAMA Netw Open (2023)
PGS003869	LDL cholesterol	low density lipoprotein cholesterol measurement	1022259	18.6	190050	38.9	397405	Shim I et al. Nature Communications (2023)

PGS003870	LDL cholesterol	low density lipoprotein cholesterol measurement	1067857	18.7	199462	39.4	421162	Shim I et al. Nature Communications (2023)
PGS003871	LDL cholesterol	low density lipoprotein cholesterol measurement	882001	18.3	161331	38.2	336606	Shim I et al. Nature Communications (2023)
PGS003872	LDL cholesterol	low density lipoprotein cholesterol measurement	958649	18.7	179164	38.1	365376	Shim I et al. Nature Communications (2023)
PGS003873	LDL cholesterol	low density lipoprotein cholesterol measurement	1069677	18.7	199910	39.5	422268	Shim I et al. Nature Communications (2023)
PGS003874	LDL cholesterol	low density lipoprotein cholesterol measurement	1054648	18.7	197363	39.5	416166	Shim I et al. Nature Communications (2023)
PGS003974	LDL cholesterol	low density lipoprotein cholesterol measurement	886257	17.6	155993	36.4	322185	Hassanin E et al. Front Genet (2023)
PGS003975	LDL cholesterol	low density lipoprotein cholesterol measurement	862971	19.5	167856	39.4	340400	Hassanin E et al. Front Genet (2023)
PGS003976	LDL cholesterol	low density lipoprotein cholesterol measurement	821134	18.9	155118	39.3	322887	Hassanin E et al. Front Genet (2023)
PGS003977	LDL cholesterol	low density lipoprotein cholesterol measurement	826248	19.2	158550	40.1	331589	Hassanin E et al. Front Genet (2023)
PGS003978	LDL cholesterol	low density lipoprotein cholesterol measurement	348056	21.1	73561	39.7	138189	Hassanin E et al. Front Genet (2023)
PGS004637	LDL cholesterol	low density lipoprotein cholesterol measurement	173	38.2	66	50.9	88	Zhang J et al. Nat Commun (2024)
PGS004638	LDL cholesterol	low density lipoprotein cholesterol measurement	1683941	18.4	310670	35.8	602375	Zhang J et al. Nat Commun (2024)
PGS004639	LDL cholesterol	low density lipoprotein cholesterol measurement	1794429	18.7	335500	36.4	652691	Zhang J et al. Nat Commun (2024)
PGS004640	LDL cholesterol	low density lipoprotein cholesterol measurement	107449	22.4	24114	42.2	45297	Zhang J et al. Nat Commun (2024)
PGS004641	LDL cholesterol	low density lipoprotein cholesterol measurement	1879609	18.7	351900	36.4	683698	Zhang J et al. Nat Commun (2024)
PGS004642	LDL cholesterol	low density lipoprotein cholesterol measurement	8643	23.2	2003	41.7	3604	Zhang J et al. Nat Commun (2024)
PGS004643	LDL cholesterol	low density lipoprotein cholesterol measurement	1194231	22	262842	41.9	499803	Zhang J et al. Nat Commun (2024)
PGS004644	LDL cholesterol	low density lipoprotein cholesterol measurement	1354681	20.9	283500	40.4	547012	Zhang J et al. Nat Commun (2024)
PGS004645	LDL cholesterol	low density lipoprotein cholesterol measurement	107449	22.4	24114	42.2	45297	Zhang J et al. Nat Commun (2024)
PGS004646	LDL cholesterol	low density lipoprotein cholesterol measurement	1879609	18.7	351900	36.4	683698	Zhang J et al. Nat Commun (2024)
PGS004647	LDL cholesterol	low density lipoprotein cholesterol measurement	307	31.6	97	48.9	150	Zhang J et al. Nat Commun (2024)
PGS004648	LDL cholesterol	low density lipoprotein cholesterol measurement	1511084	21.3	322121	42	633900	Zhang J et al. Nat Commun (2024)
PGS004649	LDL cholesterol	low density lipoprotein cholesterol measurement	1794429	18.7	335500	36.4	652691	Zhang J et al. Nat Commun (2024)
PGS004650	LDL cholesterol	low density lipoprotein cholesterol measurement	107449	22.4	24114	42.2	45297	Zhang J et al. Nat Commun (2024)
PGS004651	LDL cholesterol	low density lipoprotein cholesterol measurement	1879609	18.7	351900	36.4	683698	Zhang J et al. Nat Commun (2024)

PGS004791	LDL cholesterol	low density lipoprotein cholesterol measurement	1098797	14.8	162653	31.4	344921	Truong B et al. Cell Genom (2024)
PGS004792	LDL cholesterol	low density lipoprotein cholesterol measurement	6580710	14.5	952945	40.4	2658279	Truong B et al. Cell Genom (2024)
PGS004793	LDL cholesterol	low density lipoprotein cholesterol measurement	3946021	12.5	492060	31.8	1255575	Truong B et al. Cell Genom (2024)
PGS004794	LDL cholesterol	low density lipoprotein cholesterol measurement	6433913	14.8	951390	41.2	2653744	Truong B et al. Cell Genom (2024)
PGS004915	LDL cholesterol	low density lipoprotein cholesterol measurement	223	69.1	154	74	165	Trinder M et al. Arterioscler Thromb Vasc Biol (2019)
PGS004936	LDL cholesterol	low density lipoprotein cholesterol measurement	139875	26.9	37576	43.7	61132	Moreno-Grau S et al. Human Genomics (2024)
PGS004969	LDL cholesterol	low density lipoprotein cholesterol measurement	1286612	17.5	225726	36.9	475197	Gunn S et al. HGG Adv (2024)
PGS004970	LDL cholesterol	low density lipoprotein cholesterol measurement	1286612	17.5	225726	36.9	475197	Gunn S et al. HGG Adv (2024)
PGS004971	LDL cholesterol	low density lipoprotein cholesterol measurement	1286612	17.5	225726	36.9	475197	Gunn S et al. HGG Adv (2024)
PGS004972	LDL cholesterol	low density lipoprotein cholesterol measurement	1286612	17.5	225726	36.9	475197	Gunn S et al. HGG Adv (2024)
PGS004973	LDL cholesterol	low density lipoprotein cholesterol measurement	1286612	17.5	225726	36.9	475197	Gunn S et al. HGG Adv (2024)
PGS004974	LDL cholesterol	low density lipoprotein cholesterol measurement	1286612	17.5	225726	36.9	475197	Gunn S et al. HGG Adv (2024)
PGS004975	LDL cholesterol	low density lipoprotein cholesterol measurement	1273897	17.5	223565	36.9	470532	Gunn S et al. HGG Adv (2024)
PGS004976	LDL cholesterol	low density lipoprotein cholesterol measurement	1273897	17.5	223565	36.9	470532	Gunn S et al. HGG Adv (2024)
PGS004977	LDL cholesterol	low density lipoprotein cholesterol measurement	1273897	17.5	223565	36.9	470532	Gunn S et al. HGG Adv (2024)
PGS004978	LDL cholesterol	low density lipoprotein cholesterol measurement	1273897	17.5	223565	36.9	470532	Gunn S et al. HGG Adv (2024)
PGS004979	LDL cholesterol	low density lipoprotein cholesterol measurement	1273897	17.5	223565	36.9	470532	Gunn S et al. HGG Adv (2024)
PGS004980	LDL cholesterol	low density lipoprotein cholesterol measurement	1273897	17.5	223565	36.9	470532	Gunn S et al. HGG Adv (2024)
PGS004981	LDL cholesterol	low density lipoprotein cholesterol measurement	1277825	17.6	224903	37.1	473587	Gunn S et al. HGG Adv (2024)
PGS002730	LDL lowering in response to statin	low density lipoprotein cholesterol measurement, response to statin	35	25.7	9	57.1	20	Mayerhofer E et al. Brain (2022)
PGS000305	Fasting glucose	fasting blood glucose measurement	31	54.8	17	71	22	Xie T et al. Circ Genom Precis Med (2020)
PGS000306	Fasting glucose (body mass index adjusted)	BMI-adjusted fasting blood glucose measurement	19	52.6	10	78.9	15	Xie T et al. Circ Genom Precis Med (2020)
PGS000838	Fasting glucose	fasting blood glucose measurement	224	32.6	73	45.1	101	Aly DM et al. Nat Genet (2021)
PGS001350	Fasting glucose	fasting blood glucose measurement	1023373	18.3	186962	37.7	385473	Chen J et al. Nat Genet (2021)
PGS002924	Fasting plasma glucose	fasting blood glucose measurement	281	5.3	15	19.6	55	Ma Y et al. Am J Hum Genet (2022)
PGS002925	Fasting plasma glucose	fasting blood glucose measurement	13	0	0	7.7	1	Ma Y et al. Am J Hum Genet (2022)
PGS002926	Fasting plasma glucose	fasting blood glucose measurement	13	0	0	7.7	1	Ma Y et al. Am J Hum Genet (2022)
PGS002927	Fasting plasma glucose	fasting blood glucose measurement	5106	7.4	380	19.1	973	Ma Y et al. Am J Hum Genet (2022)

PGS002928	Fasting plasma glucose	fasting blood glucose measurement	392	23.5	92	40.6	159 Ma Y et al. Am J Hum Genet (2022)
PGS002929	Fasting plasma glucose	fasting blood glucose measurement	71791	14.4	10331	32.9	23600 Ma Y et al. Am J Hum Genet (2022)
PGS002930	Fasting plasma glucose	fasting blood glucose measurement	264	22.7	60	38.6	102 Ma Y et al. Am J Hum Genet (2022)
PGS002931	Fasting plasma glucose	fasting blood glucose measurement	273	22.7	62	39.2	107 Ma Y et al. Am J Hum Genet (2022)
PGS002932	Fasting plasma glucose	fasting blood glucose measurement	1202298	13.8	165790	32.4	389297 Ma Y et al. Am J Hum Genet (2022)
PGS002933	Fasting plasma glucose	fasting blood glucose measurement	1007415	19	191874	39.6	399210 Ma Y et al. Am J Hum Genet (2022)
PGS002944	Glucose	fasting blood glucose measurement	519	3.7	19	12.1	63 Ma Y et al. Am J Hum Genet (2022)
PGS002945	Glucose	fasting blood glucose measurement	1479	4.5	67	11.6	172 Ma Y et al. Am J Hum Genet (2022)
PGS002946	Glucose	fasting blood glucose measurement	1553	4.4	68	10.8	168 Ma Y et al. Am J Hum Genet (2022)
PGS002947	Glucose	fasting blood glucose measurement	1501	4.9	74	11.5	172 Ma Y et al. Am J Hum Genet (2022)
PGS002948	Glucose	fasting blood glucose measurement	392	23.5	92	40.6	159 Ma Y et al. Am J Hum Genet (2022)
PGS002949	Glucose	fasting blood glucose measurement	72352	14.3	10349	32.7	23670 Ma Y et al. Am J Hum Genet (2022)
PGS002950	Glucose	fasting blood glucose measurement	38	39.5	15	44.7	17 Ma Y et al. Am J Hum Genet (2022)
PGS002951	Glucose	fasting blood glucose measurement	38	39.5	15	44.7	17 Ma Y et al. Am J Hum Genet (2022)
PGS002952	Glucose	fasting blood glucose measurement	1501	4.9	74	11.5	172 Ma Y et al. Am J Hum Genet (2022)
PGS002953	Glucose	fasting blood glucose measurement	1008035	19	191934	39.6	399303 Ma Y et al. Am J Hum Genet (2022)
PGS003336	Fasting plasma glucose	fasting blood glucose measurement	56	14.3	8	17.9	10 Kim YJ et al. Nat Commun (2022)
PGS003345	Fasting plasma glucose	fasting blood glucose measurement	60	13.3	8	16.7	10 Kim YJ et al. Nat Commun (2022)
PGS000127	HbA1c	HbA1c measurement	21	38.1	8	52.4	11 Wheeler E et al. PLoS Med (2017)
PGS000128	HbA1c	HbA1c measurement	22	36.4	8	50	11 Wheeler E et al. PLoS Med (2017)
PGS000129	HbA1c	HbA1c measurement	17	35.3	6	52.9	9 Wheeler E et al. PLoS Med (2017)
PGS000130	HbA1c	HbA1c measurement	19	84.2	16	94.7	18 Wheeler E et al. PLoS Med (2017)
PGS000131	HbA1c	HbA1c measurement	19	84.2	16	94.7	18 Wheeler E et al. PLoS Med (2017)
PGS000132	HbA1c	HbA1c measurement	19	84.2	16	94.7	18 Wheeler E et al. PLoS Med (2017)
PGS000304	HbA1c	HbA1c measurement	43	60.5	26	69.8	30 Xie T et al. Circ Genom Precis Med (2020)
PGS000685	HbA1c [mmol/mol]	HbA1c measurement	14658	18.2	2670	30.5	4472 Sinnott-Armstrong N et al. Nat Genet (2021)
PGS001352	Glycated haemoglobin levels (HbA1c)	HbA1c measurement	1018836	18.3	185972	37.6	383028 Chen J et al. Nat Genet (2021)
PGS001953	Glycated haemoglobin (HbA1c)	HbA1c measurement	46566	21.3	9909	41	19097 Privé F et al. Am J Hum Genet (2022)
PGS002171	Glycated haemoglobin (HbA1c)	HbA1c measurement	736730	18.5	136187	38.9	286837 Privé F et al. Am J Hum Genet (2022)
PGS002331	HbA1c	HbA1c measurement	1109311	18.7	207730	39.5	437977 Weissbrod O et al. Nat Genet (2022)
PGS002367	HbA1c	HbA1c measurement	920924	19.2	177231	39.6	365124 Weissbrod O et al. Nat Genet (2022)
PGS002403	HbA1c	HbA1c measurement	11872	7.1	844	16.7	1982 Weissbrod O et al. Nat Genet (2022)
PGS002452	HbA1c	HbA1c measurement	30603	5.5	1670	13.7	4204 Weissbrod O et al. Nat Genet (2022)
PGS002501	HbA1c	HbA1c measurement	128425	4.2	5339	10.6	13618 Weissbrod O et al. Nat Genet (2022)
PGS002550	HbA1c	HbA1c measurement	4546	8.8	402	19.6	889 Weissbrod O et al. Nat Genet (2022)
PGS002599	HbA1c	HbA1c measurement	3055	9.6	292	20.1	615 Weissbrod O et al. Nat Genet (2022)
PGS002648	HbA1c	HbA1c measurement	394312	8.6	34072	22.5	88570 Weissbrod O et al. Nat Genet (2022)
PGS002697	HbA1c	HbA1c measurement	989344	18.4	182004	39.3	389252 Weissbrod O et al. Nat Genet (2022)
PGS003337	HbA1c	HbA1c measurement	68	16.2	11	25	27 Kim YJ et al. Nat Commun (2022)
PGS003346	HbA1c	HbA1c measurement	74	17.6	13	25.7	19 Kim YJ et al. Nat Commun (2022)
PGS003471	HbA1c	HbA1c measurement	848979	19	161269	40.9	347133 Zhang Y et al. EBioMedicine (2022)
PGS003533	Glycated haemoglobin (HbA1c)	HbA1c measurement	979739	19.1	187570	40.9	400815 Ding Y et al. bioRxiv (2022) | Pre
PGS003987	HbA1c	HbA1c measurement	1009642	18.6	187665	39.1	394822 Monti R et al. Am J Hum Genet (2024)
PGS004003	HbA1c	HbA1c measurement	4697	22.6	1060	41.6	1956 Monti R et al. Am J Hum Genet (2024)
PGS004029	HbA1c	HbA1c measurement	907906	18.7	169865	39.3	356991 Monti R et al. Am J Hum Genet (2024)

PGS004044	HbA1c	HbA1c measurement	907906	18.7	169865	39.3	356991	Monti R et al. Am J Hum Genet (2024)
PGS004057	HbA1c	HbA1c measurement	514367	17.7	90851	37.6	193224	Monti R et al. Am J Hum Genet (2024)
PGS004073	HbA1c	HbA1c measurement	514367	17.7	90851	37.6	193224	Monti R et al. Am J Hum Genet (2024)
PGS004087	HbA1c	HbA1c measurement	989845	18.6	184336	39.1	387228	Monti R et al. Am J Hum Genet (2024)
PGS004111	HbA1c	HbA1c measurement	51	49	25	68.6	35	Monti R et al. Am J Hum Genet (2024)
PGS004127	HbA1c	HbA1c measurement	246	38.6	95	56.5	139	Monti R et al. Am J Hum Genet (2024)
PGS004141	HbA1c	HbA1c measurement	683029	18.7	127642	39.7	271064	Monti R et al. Am J Hum Genet (2024)
PGS004157	HbA1c	HbA1c measurement	1009664	18.6	187668	39.1	394827	Monti R et al. Am J Hum Genet (2024)
PGS004337	Glycated haemoglobin (mmol/mol)	HbA1c measurement	1059939	18.6	197409	39.5	418909	Jung H et al. Commun Biol (2024)
PGS004701	HbA1c	HbA1c measurement	1107927	14.2	157776	30.4	337013	Truong B et al. Cell Genom (2024)
PGS004702	HbA1c	HbA1c measurement	6652699	14.4	960322	40.3	2679646	Truong B et al. Cell Genom (2024)
PGS004703	HbA1c	HbA1c measurement	3872159	12.6	488836	32.1	1242667	Truong B et al. Cell Genom (2024)
PGS004704	HbA1c	HbA1c measurement	6652699	14.4	960322	40.3	2679646	Truong B et al. Cell Genom (2024)

Table S3 Source information and performance matrics of selected PRS with match rate of more than 50%,

	Souce Information						Performance Matrics				
PGS_id	Reported trait	Mapped Trait (EFO label)	Number of Variants	Development Method	Development Samples	Citation	Sample Set	Trait	Metrics	Covariates	Citation
PGS000031	Type 2 diabetes (T2D)	type 2 diabetes mellitus	62	GWAS, OR	European:69,033 individuals	Vassy JL et al. Diabetes (2014)	European:3,471 individuals	Reported Trait: Incident type 2 diabetes cases	HR: 1.06 [1.04, 1.08]	age, sex, family history (parents), body mass index, systolic blood pressure, fasting glucose, log-HDL cholesterol, log-triglyceride levels	Vassy JL et al. Diabetes (2014)
							European:1,650 individuals	Reported Trait: Incident type 2 diabetes cases	HR: 1.06 [1.02, 1.1]	age, sex, family history (parents), body mass index, systolic blood pressure, fasting glucose, log-HDL cholesterol, log-triglyceride levels	Vassy JL et al. Diabetes (2014)
							African:820 individuals	Reported Trait: Incident type 2 diabetes cases	HR: 1.05 [1.0, 1.09]	age, sex, family history (parents), body mass index, systolic blood pressure, fasting glucose, log-HDL cholesterol, log-triglyceride levels	Vassy JL et al. Diabetes (2014)
PGS000032	Type 2 diabetes (based on SNPs involved in β-cell function)	type 2 diabetes mellitus	20	GWAS, OR	European:69,033 individuals	Vassy JL et al. Diabetes (2014)	European:3,471 individuals	Reported Trait: Incident type 2 diabetes cases	HR: 1.1 [1.06, 1.14]	age, sex, family history (parents), body mass index, systolic blood pressure, fasting glucose, log-HDL cholesterol, log-triglyceride levels	Vassy JL et al. Diabetes (2014)
							European:1,650 individuals	Reported Trait: Incident type 2 diabetes cases	HR: 1.09 [1.02, 1.17]	age, sex, family history (parents), body mass index, systolic blood pressure, fasting glucose, log-HDL cholesterol, log-triglyceride levels	Vassy JL et al. Diabetes (2014)
							African:820 individuals	Reported Trait: Incident type 2 diabetes cases	HR: 1.06 [0.99, 1.15]	age, sex, family history (parents), body mass index, systolic blood pressure, fasting glucose, log-HDL cholesterol, log-triglyceride levels	Vassy JL et al. Diabetes (2014)
							5,740 individuals	Reported Trait: Cystic-fibrosis related diabetes onset	HR: 1.192	PCs(1-4), site of recruitment	Aksit MA et al. J Clin Endocrinol Metab (2020)
PGS000033	Type 2 diabetes (based on SNPs involved in insulin resistance)	type 2 diabetes mellitus	10	GWAS, OR	European:69,033 individuals	Vassy JL et al. Diabetes (2014)	European:3,471 individuals	Reported Trait: Incident type 2 diabetes cases	HR: 0.98 [0.93, 1.04]	age, sex, family history (parents), body mass index, systolic blood pressure, fasting glucose, log-HDL cholesterol, log-triglyceride levels	Vassy JL et al. Diabetes (2014)
							European:1,650 individuals	Reported Trait: Incident type 2 diabetes cases	HR: 1.01 [0.91, 1.12]	age, sex, family history (parents), body mass index, systolic blood pressure, fasting glucose, log-HDL cholesterol, log-triglyceride levels	Vassy JL et al. Diabetes (2014)
							African:820 individuals	Reported Trait: Incident type 2 diabetes cases	HR: 1.06 [0.99, 1.15]	age, sex, family history (parents), body mass index, systolic blood pressure, fasting glucose, log-HDL cholesterol, log-triglyceride levels	Vassy JL et al. Diabetes (2014)
PGS000125	Type 2 diabetes (T2D)	type 2 diabetes mellitus	80	GWAS, OR	Multiancestry:169,298 individuals	Qi Q et al. Diabetes (2017)	Hispanic or Latin American:7,746 individuals	Reported Trait: Type 2 Diabetes	OR: 1.071.06, 1.08	center, age, sex, 5 PCs of ancestry	Qi Q et al. Diabetes (2017)
PGS000848	Type 2 diabetes (based on SNPs associated with adiposity)	type 2 diabetes mellitus	6	GWAS, OR	European:298,957 individuals	Aly DM et al. Nat Genet (2021)	European:3,194 individuals	Reported Trait: Severe Autoimmune Diabetes	OR: 1.11 [1.0, 1.23]	PC1-10	Aly DM et al. Nat Genet (2021)
							European:3,930 individuals	Reported Trait: Severe Insulin-Deficient Diabetes	OR: 1.1 [1.02, 1.18]	PC1-10	Aly DM et al. Nat Genet (2021)
							European:3,869 individuals	Reported Trait: Severe Insulin-Resistant Diabetes	OR: 1.11 [1.03, 1.19]	PC1-10	Aly DM et al. Nat Genet (2021)
							European:4,116 individuals	Reported Trait: Moderate Obesity-related Diabetes	OR: 1.2 [1.12, 1.28]	PC1-10	Aly DM et al. Nat Genet (2021)
							European:5,597 individuals	Reported Trait: Moderate Age-Related Diabetes	OR: 1.06 [1.0, 1.12]	PC1-10	Aly DM et al. Nat Genet (2021)
							South Asian:1,282 individuals	Reported Trait: Type 2 diabetes	OR: 1.14 [1.01, 1.28]	sex	Yajnik CS et al. Lancet Reg Health Southeast Asia (2023)
							South Asian:830 individuals	Reported Trait: BMI	OR: 1.23 [1.07, 1.43]	sex	Yajnik CS et al. Lancet Reg Health Southeast Asia (2023)
PGS000849	Type 2 diabetes (based on SNPs associated with impared lipid)	type 2 diabetes mellitus	3	GWAS, OR	European:298,957 individuals	Aly DM et al. Nat Genet (2021)	European:3,194 individuals	Reported Trait: Severe insulin deficiency diabetes	OR: 1.08 [0.97, 1.2]	PC1-10	Aly DM et al. Nat Genet (2021)
							European:3,930 individuals	Reported Trait: Severe Insulin-Deficient Diabetes	OR: 1.07 [1.0, 1.15]	PC1-10	Aly DM et al. Nat Genet (2021)
							European:3,869 individuals	Reported Trait: Severe Insulin-Resistant Diabetes	OR: 1.09 [1.01, 1.17]	PC1-10	Aly DM et al. Nat Genet (2021)
							European:4,116 individuals	Reported Trait: Moderate Obesity-related Diabetes	OR: 1.0 [0.93, 1.07]	PC1-10	Aly DM et al. Nat Genet (2021)
							European:5,597 individuals	Reported Trait: Moderate Age-Related Diabetes	OR: 1.06 [1.0, 1.12]	PC1-10	Aly DM et al. Nat Genet (2021)
							South Asian:1,282 individuals	Reported Trait: Type 2 diabetes	OR: 1.2 [1.0, 1.44]	age, sex and BMI	Yajnik CS et al. Lancet Reg Health Southeast Asia (2023)
							South Asian:830 individuals	Reported Trait: Severe insulin deficiency diabetes	OR: 1.2 [1.03, 1.4]	sex	Yajnik CS et al. Lancet Reg Health Southeast Asia (2023)
							South Asian:729 individuals	Reported Trait: Mild obesity-related diabetes	OR: 1.26 [1.07, 1.49]	sex	Yajnik CS et al. Lancet Reg Health Southeast Asia (2023)
PGS000850	Type 2 diabetes (based on SNPs associated with insulin action)	type 2 diabetes mellitus	16	GWAS, OR	European:298,957 individuals	Aly DM et al. Nat Genet (2021)	European:3,194 individuals	Reported Trait: Severe Autoimmune Diabetes	OR: 1.08 [0.98, 1.2]	PC1-10	Aly DM et al. Nat Genet (2021)
							European:3,930 individuals	Reported Trait: Severe Insulin-Deficient Diabetes	OR: 1.17 [1.09, 1.25]	PC1-10	Aly DM et al. Nat Genet (2021)
							European:3,869 individuals	Reported Trait: Severe Insulin-Resistant Diabetes	OR: 1.17 [1.09, 1.26]	PC1-10	Aly DM et al. Nat Genet (2021)
							European:4,116 individuals	Reported Trait: Moderate Obesity-related Diabetes	OR: 1.14 [1.07, 1.22]	PC1-10	Aly DM et al. Nat Genet (2021)
							European:5,597 individuals	Reported Trait: Moderate Age-Related Diabetes	OR: 1.16 [1.1, 1.23]	PC1-10	Aly DM et al. Nat Genet (2021)
							South Asian:1,282 individuals	Reported Trait: Type 2 diabetes	OR: 1.16 [1.03, 1.3]	sex	Yajnik CS et al. Lancet Reg Health Southeast Asia (2023)
							South Asian:830 individuals	Reported Trait: Severe insulin deficiency diabetes	OR: 1.17 [1.0, 1.36]	sex	Yajnik CS et al. Lancet Reg Health Southeast Asia (2023)
							South Asian:729 individuals	Reported Trait: Mild age-related diabetes	OR: 1.26 [1.04, 1.53]	sex	Yajnik CS et al. Lancet Reg Health Southeast Asia (2023)
PGS000851	Type 2 diabetes (based on SNPs associated with insulin action/secretion)	type 2 diabetes mellitus	37	GWAS, OR	European:298,957 individuals	Aly DM et al. Nat Genet (2021)	European:3,194 individuals	Reported Trait: Severe Autoimmune Diabetes	OR: 1.24 [1.12, 1.37]	PC1-10	Aly DM et al. Nat Genet (2021)
							European:3,930 individuals	Reported Trait: Severe Insulin-Deficient Diabetes	OR: 1.23 [1.15, 1.32]	PC1-10	Aly DM et al. Nat Genet (2021)
							European:3,869 individuals	Reported Trait: Severe Insulin-Resistant Diabetes	OR: 1.04 [0.97, 1.11]	PC1-10	Aly DM et al. Nat Genet (2021)
							European:4,116 individuals	Reported Trait: Moderate Obesity-related Diabetes	OR: 1.19 [1.12, 1.28]	PC1-10	Aly DM et al. Nat Genet (2021)
							European:5,597 individuals	Reported Trait: Moderate Age-Related Diabetes	OR: 1.19 [1.13, 1.26]	PC1-10	Aly DM et al. Nat Genet (2021)
							South Asian:1,282 individuals	Reported Trait: Type 2 diabetes	OR: 1.07 [0.9, 1.28]	age, sex and BMI	Yajnik CS et al. Lancet Reg Health Southeast Asia (2023)
							South Asian:830 individuals	Reported Trait: Mild obesity-related diabetes	OR: 1.17 [1.01, 1.34]	sex	Yajnik CS et al. Lancet Reg Health Southeast Asia (2023)
							South Asian:729 individuals	Reported Trait: Mild age-related diabetes	OR: 1.45 [1.19, 1.78]	sex	Yajnik CS et al. Lancet Reg Health Southeast Asia (2023)
PGS000852	Type 2 diabetes (based on SNPs associated with insulin secretion)	type 2 diabetes mellitus	8	GWAS, OR	European:298,957 individuals	Aly DM et al. Nat Genet (2021)	European:3,194 individuals	Reported Trait: Severe Autoimmune Diabetes	OR: 1.01 [0.91, 1.12]	PC1-10	Aly DM et al. Nat Genet (2021)
							European:3,930 individuals	Reported Trait: Severe Insulin-Deficient Diabetes	OR: 1.31 [1.22, 1.41]	PC1-10	Aly DM et al. Nat Genet (2021)
							European:3,869 individuals	Reported Trait: Severe Insulin-Resistant Diabetes	OR: 1.04 [0.97, 1.12]	PC1-10	Aly DM et al. Nat Genet (2021)
							European:4,116 individuals	Reported Trait: Moderate Obesity-related Diabetes	OR: 1.26 [1.18, 1.35]	PC1-10	Aly DM et al. Nat Genet (2021)

							European:5,597 individuals	Reported Trait: Moderate Age-Related Diabetes	OR: 1.29 [1.22, 1.37]	PC1-10	Aly DM et al. Nat Genet (2021)
							South Asian:1,282 individuals	Reported Trait: Type 2 diabetes	OR: 1.33 [1.12, 1.58]	age, sex and BMI	Yajnik CS et al. Lancet Reg Health Southeast Asia (2023)
							South Asian:830 individuals	Reported Trait: Severe insulin deficiency diabetes	OR: 1.38 [1.19, 1.59]	sex	Yajnik CS et al. Lancet Reg Health Southeast Asia (2023)
							South Asian:729 individuals	Reported Trait: Mild obesity-related diabetes	OR: 1.25 [1.08, 1.46]	sex	Yajnik CS et al. Lancet Reg Health Southeast Asia (2023)
							South Asian:624 individuals	Reported Trait: Mild age-related diabetes	OR: 1.33 [1.1, 1.61]	sex	Yajnik CS et al. Lancet Reg Health Southeast Asia (2023)
PGS000853	Type 2 diabetes (based on SNPs associated with insulin secretion)	type 2 diabetes mellitus	21	GWAS, OR	European:298,957 individuals	Aly DM et al. Nat Genet (2021)	European:3,194 individuals	Reported Trait: Severe Autoimmune Diabetes	OR: 0.99 [0.9, 1.1]	PC1-10	Aly DM et al. Nat Genet (2021)
							European:3,930 individuals	Reported Trait: Severe Insulin-Deficient Diabetes	OR: 1.26 [1.18, 1.36]	PC1-10	Aly DM et al. Nat Genet (2021)
							European:3,869 individuals	Reported Trait: Severe Insulin-Resistant Diabetes	OR: 1.02 [0.95, 1.09]	PC1-10	Aly DM et al. Nat Genet (2021)
							European:4,116 individuals	Reported Trait: Moderate Obesity-related Diabetes	OR: 1.18 [1.11, 1.27]	PC1-10	Aly DM et al. Nat Genet (2021)
							European:5,597 individuals	Reported Trait: Moderate Age-Related Diabetes	OR: 1.23 [1.17, 1.3]	PC1-10	Aly DM et al. Nat Genet (2021)
							South Asian:1,282 individuals	Reported Trait: Type 2 diabetes	OR: 1.32 [1.11, 1.57]	age, sex and BMI	Yajnik CS et al. Lancet Reg Health Southeast Asia (2023)
							South Asian:729 individuals	Reported Trait: Mild obesity-related diabetes	OR: 1.24 [1.07, 1.45]	sex	Yajnik CS et al. Lancet Reg Health Southeast Asia (2023)
							South Asian:624 individuals	Reported Trait: Mild age-related diabetes	OR: 1.32 [1.1, 1.59]	sex	Yajnik CS et al. Lancet Reg Health Southeast Asia (2023)
PGS000854	Type 2 diabetes (based on SNPs associated with beta cell function)	type 2 diabetes mellitus	27	GWAS, OR	Multiancestry:17,365 individuals	Aly DM et al. Nat Genet (2021)	European:3,194 individuals	Reported Trait: Severe Autoimmune Diabetes	OR: 1.0 [0.91, 1.11]	PC1-10	Aly DM et al. Nat Genet (2021)
							European:3,930 individuals	Reported Trait: Severe Insulin-Deficient Diabetes	OR: 1.32 [1.23, 1.42]	PC1-10	Aly DM et al. Nat Genet (2021)
							European:3,869 individuals	Reported Trait: Severe Insulin-Resistant Diabetes	OR: 1.01 [0.94, 1.08]	PC1-10	Aly DM et al. Nat Genet (2021)
							European:4,116 individuals	Reported Trait: Moderate Obesity-related Diabetes	OR: 1.21 [1.13, 1.3]	PC1-10	Aly DM et al. Nat Genet (2021)
							European:5,597 individuals	Reported Trait: Moderate Age-Related Diabetes	OR: 1.27 [1.2, 1.34]	PC1-10	Aly DM et al. Nat Genet (2021)
							South Asian:1,282 individuals	Reported Trait: Type 2 diabetes	OR: 0.89 [0.79, 1.0]	age, sex and BMI	Yajnik CS et al. Lancet Reg Health Southeast Asia (2023)
							South Asian:729 individuals	Reported Trait: Severe insulin deficiency diabetes	OR: 0.87 [0.75, 1.0]	sex	Yajnik CS et al. Lancet Reg Health Southeast Asia (2023)
							South Asian:624 individuals	Reported Trait: Mild age-related diabetes	OR: 0.81 [0.67, 0.97]	sex	Yajnik CS et al. Lancet Reg Health Southeast Asia (2023)
PGS000855	Type 2 diabetes (based on SNPs associated with lipodystrophy)	type 2 diabetes mellitus	18	GWAS, OR	Multiancestry:17,365 individuals	Aly DM et al. Nat Genet (2021)	European:3,194 individuals	Reported Trait: Severe Autoimmune Diabetes	OR: 1.12 [1.01, 1.25]	PC1-10	Aly DM et al. Nat Genet (2021)
							European:3,930 individuals	Reported Trait: Severe Insulin-Deficient Diabetes	OR: 1.23 [1.15, 1.33]	PC1-10	Aly DM et al. Nat Genet (2021)
							European:3,869 individuals	Reported Trait: Severe Insulin-Resistant Diabetes	OR: 1.15 [1.07, 1.24]	PC1-10	Aly DM et al. Nat Genet (2021)
							European:4,116 individuals	Reported Trait: Moderate Obesity-related Diabetes	OR: 1.14 [1.06, 1.22]	PC1-10	Aly DM et al. Nat Genet (2021)
							European:5,597 individuals	Reported Trait: Moderate Age-Related Diabetes	OR: 1.18 [1.11, 1.24]	PC1-10	Aly DM et al. Nat Genet (2021)
							South Asian:1,282 individuals	Reported Trait: Type 2 diabetes	OR: 1.17 [1.04, 1.31]	age, sex and BMI	Yajnik CS et al. Lancet Reg Health Southeast Asia (2023)
							South Asian:830 individuals	Reported Trait: Severe insulin deficiency diabetes	OR: 1.16 [1.0, 1.34]	sex	Yajnik CS et al. Lancet Reg Health Southeast Asia (2023)
							South Asian:729 individuals	Reported Trait: Mild obesity-related diabetes	OR: 1.18 [1.01, 1.37]	sex	Yajnik CS et al. Lancet Reg Health Southeast Asia (2023)
PGS000856	Type 2 diabetes (based on SNPs associated with liver lipids)	type 2 diabetes mellitus	3	GWAS, OR	Multiancestry:17,365 individuals	Aly DM et al. Nat Genet (2021)	European:3,194 individuals	Reported Trait: Severe Autoimmune Diabetes	OR: 1.35 [1.22, 1.51]	PC1-10	Aly DM et al. Nat Genet (2021)
							European:3,930 individuals	Reported Trait: Severe Insulin-Deficient Diabetes	OR: 1.01 [0.94, 1.08]	PC1-10	Aly DM et al. Nat Genet (2021)
							European:3,869 individuals	Reported Trait: Severe Insulin-Resistant Diabetes	OR: 0.97 [0.91, 1.05]	PC1-10	Aly DM et al. Nat Genet (2021)
							European:4,116 individuals	Reported Trait: Moderate Obesity-related Diabetes	OR: 1.02 [0.95, 1.09]	PC1-10	Aly DM et al. Nat Genet (2021)
							European:5,597 individuals	Reported Trait: Moderate Age-Related Diabetes	OR: 0.95 [0.9, 1.01]	PC1-10	Aly DM et al. Nat Genet (2021)
							South Asian:1,282 individuals	Reported Trait: Type 2 diabetes	OR: 1.29 [1.09, 1.54]	age, sex and BMI	Yajnik CS et al. Lancet Reg Health Southeast Asia (2023)
							South Asian:830 individuals	Reported Trait: Severe insulin deficiency diabetes	OR: 1.24 [1.07, 1.43]	sex	Yajnik CS et al. Lancet Reg Health Southeast Asia (2023)
							South Asian:729 individuals	Reported Trait: Mild obesity-related diabetes	OR: 1.31 [1.12, 1.53]	sex	Yajnik CS et al. Lancet Reg Health Southeast Asia (2023)
							South Asian:482 individuals	Reported Trait: Severe insulin-resistant diabetes	OR: 2.04 [1.26, 3.3]	sex	Yajnik CS et al. Lancet Reg Health Southeast Asia (2023)
PGS000857	Type 2 diabetes (based on SNPs associated with obesity)	type 2 diabetes mellitus	4	GWAS, OR	Multiancestry:17,365 individuals	Aly DM et al. Nat Genet (2021)	European:3,194 individuals	Reported Trait: Severe Autoimmune Diabetes	OR: 1.07 [0.97, 1.19]	PC1-10	Aly DM et al. Nat Genet (2021)
							European:3,930 individuals	Reported Trait: Severe Insulin-Deficient Diabetes	OR: 1.08 [1.01, 1.16]	PC1-10	Aly DM et al. Nat Genet (2021)
							European:3,869 individuals	Reported Trait: Severe Insulin-Resistant Diabetes	OR: 1.13 [1.05, 1.22]	PC1-10	Aly DM et al. Nat Genet (2021)
							European:4,116 individuals	Reported Trait: Moderate Obesity-related Diabetes	OR: 1.19 [1.11, 1.27]	PC1-10	Aly DM et al. Nat Genet (2021)
							European:5,597 individuals	Reported Trait: Moderate Age-Related Diabetes	OR: 1.04 [0.99, 1.1]	PC1-10	Aly DM et al. Nat Genet (2021)
							South Asian:1,282 individuals	Reported Trait: Type 2 diabetes	OR: 0.87 [0.77, 0.99]	sex	Yajnik CS et al. Lancet Reg Health Southeast Asia (2023)
PGS000858	Type 2 diabetes (based on SNPs associated with proinsulin level)	type 2 diabetes mellitus	6	GWAS, OR	Multiancestry:17,365 individuals	Aly DM et al. Nat Genet (2021)	European:3,194 individuals	Reported Trait: Severe Autoimmune Diabetes	OR: 0.94 [0.85, 1.04]	PC1-10	Aly DM et al. Nat Genet (2021)
							European:3,930 individuals	Reported Trait: Severe Insulin-Deficient Diabetes	OR: 1.14 [1.06, 1.22]	PC1-10	Aly DM et al. Nat Genet (2021)
							European:3,869 individuals	Reported Trait: Severe Insulin-Resistant Diabetes	OR: 0.93 [0.87, 1.0]	PC1-10	Aly DM et al. Nat Genet (2021)
							European:4,116 individuals	Reported Trait: Moderate Obesity-related Diabetes	OR: 1.06 [0.99, 1.13]	PC1-10	Aly DM et al. Nat Genet (2021)
							European:5,597 individuals	Reported Trait: Moderate Age-Related Diabetes	OR: 1.1 [1.04, 1.16]	PC1-10	Aly DM et al. Nat Genet (2021)
							South Asian:1,282 individuals	Reported Trait: Type 2 diabetes	OR: 1.22 [1.03, 1.44]	age, sex and BMI	Yajnik CS et al. Lancet Reg Health Southeast Asia (2023)
							South Asian:729 individuals	Reported Trait: Mild obesity-related diabetes	OR: 1.2 [1.03, 1.4]	sex	Yajnik CS et al. Lancet Reg Health Southeast Asia (2023)
							South Asian:624 individuals	Reported Trait: Mild age-related diabetes	OR: 1.23 [1.01, 1.48]	sex	Yajnik CS et al. Lancet Reg Health Southeast Asia (2023)
PGS002277	Type 2 diabetes (based on SNPs associated with insulin secretion)	type 2 diabetes mellitus	8	Partitioned polygenic score: variant from PMID:29632382, weights from PMID:20081858	Multiancestry:488,710 individuals	Siddiqui MK et al. Diabetologia (2022)	South Asian:5,806 individuals	Reported Trait: Age at diagnosis	Z: 5.2		Siddiqui MK et al. Diabetologia (2022)

							South Asian:5,806 individuals	Reported Trait: Age at diagnosis (normal BMI)	β: 37.31 (16.7), R^2: 0.012		Siddiqui MK et al. Diabetologia (2022)
							South Asian:5,806 individuals	Reported Trait: Age at diagnosis (overweight BMI)	β: 32.68 (20.7), R^2: 0.0097		Siddiqui MK et al. Diabetologia (2022)
							South Asian:5,806 individuals	Reported Trait: Age at diagnosis (obese BMI)	β: 18.9 (10.76), R^2: 0.0036		Siddiqui MK et al. Diabetologia (2022)
PGS002733	Type 2 diabetes (T2D)	type 2 diabetes mellitus	17	GWAS with OR≥1.10 and AF ≥10%, log(OR)	European:159,208 individuals	Pezzilli S et al. Diabetes Metab (2022)	European:600 individuals	Reported Trait: Early-onset type 2 diabetes	OR: 1.09 [1.01, 1.18]		Pezzilli S et al. Diabetes Metab (2022)
							European:600 individuals	Reported Trait: Early-onset type 2 diabetes in rare variant carriers	OR: 1.45 [1.15, 1.57]		Pezzilli S et al. Diabetes Metab (2022)
							European:600 individuals	Reported Trait: Early-onset type 2 diabetes in rare variant non-carriers	OR: 1.06 [1.01, 1.13]		Pezzilli S et al. Diabetes Metab (2022)
**PGS004106**	**Type 2 diabetes (T2D)**	**type 2 diabetes mellitus**	**35**	**pt_clump.auto, r2 = 0.1, window = 250kb, p = 1e-10**	**European:159,208 individuals**	**Monti R et al. Am J Hum Genet (2024)**	**European:199,274 individuals**	**Reported Trait: Type 2 Diabetes**	**OR: 1.25413, β: 0.22644, AUROC: 0.56**		**Monti R et al. Am J Hum Genet (2024)**
						**Monti R et al. Am J Hum Genet (2024)**	**European:377,408 individuals**	**Reported Trait: Type 2 Diabetes**	**OR: 1.2706, β: 0.23949, AUROC: 0.57**		**Monti R et al. Am J Hum Genet (2024)**
						**Monti R et al. Am J Hum Genet (2024)**	**South Asian:44,057 individuals**	**Reported Trait: Type 2 Diabetes**	**OR: 1.1856, β: 0.17025, AUROC: 0.55**		**Monti R et al. Am J Hum Genet (2024)**
						**Monti R et al. Am J Hum Genet (2024)**	**European:66,865 individuals**	**Reported Trait: Type 2 Diabetes**	**OR: 1.30688, β: 0.26765, AUROC: 0.58**		**Monti R et al. Am J Hum Genet (2024)**
						**Monti R et al. Am J Hum Genet (2024)**	**South Asian:9,326 individuals**	**Reported Trait: Type 2 Diabetes**	**OR: 1.25033, β: 0.22341, AUROC: 0.56**		**Monti R et al. Am J Hum Genet (2024)**
						**Monti R et al. Am J Hum Genet (2024)**	**European:90,274 individuals**	**Reported Trait: Type 2 Diabetes**	**OR: 1.32877, β: 0.28426, AUROC: 0.58**		**Monti R et al. Am J Hum Genet (2024)**
PGS004225	Type 2 diabetes	type 2 diabetes mellitus	46	GWAS with r2 <0.8and MAF >0.5,	East Asian:974,507 individuals	Liu J et al. Nutrients (2023)	East Asian:5,024 individuals	Reported Trait: Type 2 diabetes	Hazard ratio (HR, high vs low quintile): 2.06 [1.42, 2.97]	age, gender, SBP, DBP, FBG, TC, TG, HDLC, and diabetes family history	Liu J et al. Nutrients (2023)
							East Asian:5,024 individuals	Reported Trait: Type 2 diabetes with lifestyle group	Hazard ratio (HR, poor lifestyle and high PRS vs. ideal lifestyle and low PRS): 3.93 [2.07, 7.44]		Liu J et al. Nutrients (2023)
PGS004226	Type 2 diabetes	type 2 diabetes mellitus	50	GWAS with r2 <0.8and MAF >0.5,	European:1,713,731 individuals	Liu J et al. Nutrients (2023)	East Asian:5,024 individuals	Reported Trait: Type 2 diabetes	Hazard ratio (HR, high vs low quintile): 1.69 [1.17, 2.44]	age, gender, SBP, DBP, FBG, TC, TG, HDLC, and diabetes family history	Liu J et al. Nutrients (2023)
PGS000010	Coronary heart disease	coronary artery disease	27	GWAS, OR	European:86,995 individuals	Mega JL et al. Lancet (2015)	European:42,998 individuals	Reported Trait: Coronary heart disease	HR: 1.21 [1.17, 1.26]	age, sex, diabetes status, smoking, race, family history of coronary heart disease, HDL cholesterol,… LDL cholesterol, and hypertensionShow more	Mega JL et al. Lancet (2015)
							European:4,877 individuals	Reported Trait: Coronary heart disease	HR: 1.14 [1.02, 1.28]	age, sex, diabetes status, smoking, race, family history of coronary heart disease, HDL cholesterol,… LDL cholesterol, and hypertensionShow more	Mega JL et al. Lancet (2015)
							European:23,595 individuals	Reported Trait: Incident coronary heart disease	HR: 1.2 [1.15, 1.25]	age, sex, systolic blood pressure, hypertension treatment, smoking, apoB, apoA-I, prevalent diabetes	Tada H et al. Eur Heart J (2015) | Ext.
							European:12,676 individuals	Reported Trait: Incident coronary artery disease	HR: 1.21 [1.12, 1.3]	—	Abraham G et al. Eur Heart J (2016) | Ext.
							European:3,406 individuals	Reported Trait: Incident coronary artery disease	HR: 1.16 [1.07, 1.26]	—	Abraham G et al. Eur Heart J (2016) | Ext.
							European:4,932 individuals	Reported Trait: Reccurent cardiovascular event (coronary heart disease death, non-fatal myocardial infraction, unstable angina pectoris, coronary artery bypass graft and Percutaneous coronary intervention)	C-index: 0.7	Hypertension, low-density lipoprotein cholesterol, high-density lipoprotein cholesterol, diabetes, s… ex, age, current smokingShow more	Thompson PL et al. BMC Cardiovasc Disord (2022) | Ext.
PGS000011	Coronary artery disease	coronary artery disease	50	GWAS, OR	Multiancestry:365,042 individuals	Tada H et al. Eur Heart J (2015)	European:23,595 individuals	Reported Trait: Incident coronary heart disease	HR: 1.23 [1.18, 1.28]	age, sex, systolic blood pressure, hypertension treatment, smoking, apoB, apoA-I, prevalent diabetes	Tada H et al. Eur Heart J (2015)
							Multiancestry:482,629 individuals	Reported Trait: Incident coronary artery disease	HR: 1.263 [1.247, 1.28]	sex, genetic PCs (1-10), genotyping array	Inouye M et al. J Am Coll Cardiol (2018) | Ext.
							European:7,814 individuals	Reported Trait: Incident coronary artery disease	Hazard Ratio (HR; top 20% of score vs bottom 20%): 1.75 [1.46, 2.1]	age, sex, self reported education level, 5 genetic principal components	Khera AV et al. N Engl J Med (2016) | Ext.
							European:21,222 individuals	Reported Trait: Incident coronary artery disease	Hazard Ratio (HR; top 20% of score vs bottom 20%): 1.94 [1.58, 2.39]	age, self reported education level, treatment (vitamin E vs aspirin), 5 genetic principal components	Khera AV et al. N Engl J Med (2016) | Ext.
							European:22,389 individuals	Reported Trait: Incident coronary artery disease	Hazard Ratio (HR; top 20% of score vs bottom 20%): 1.98 [1.76, 2.23]	age, sex, self reported education level	Khera AV et al. N Engl J Med (2016) | Ext.
							European:4,260 individuals	Reported Trait: Coronary artery calcification	Agatston score (mean, top 20% of GRS): 46.0 [9.0, 54.0] Agatston score (mean, btttom 25% of GRS): 21.0 [18.0, 25.0]	—	Khera AV et al. N Engl J Med (2016) | Ext.
							European:39,758 individuals	Reported Trait: Incident coronary heart disease	HR: 1.2 [1.15, 1.25], C-index: 0.698	sex, eMERGE site, first five ancestry-specific principal components	Dikilitas O et al. Am J Hum Genet (2020) | Ext.
							African:7,070 individuals	Reported Trait: Incident coronary heart disease	HR: 1.05 [0.94, 1.17], C-index: 0.649	sex, eMERGE site, first five ancestry-specific principal components	Dikilitas O et al. Am J Hum Genet (2020) | Ext.
							Hispanic or Latin American:2,194 individuals	Reported Trait: Incident coronary heart disease	HR: 1.13 [0.93, 1.36], C-index: 0.654	sex, eMERGE site, first five ancestry-specific principal components	Dikilitas O et al. Am J Hum Genet (2020) | Ext.
							European:45,645 individuals	Reported Trait: Coronary heart disease (incident and prevalent)	OR: 1.28 [1.25, 1.32], AUROC: 0.75	age at first EHR record, duration of EHR, sex, eMERGE site, first five ancestry-specific principal c… omponentsShow more	Dikilitas O et al. Am J Hum Genet (2020) | Ext.
							African:7,597 individuals	Reported Trait: Coronary heart disease (incident and prevalent)	OR: 1.05 [0.98, 1.14], AUROC: 0.763	age at first EHR record, duration of EHR, sex, eMERGE site, first five ancestry-specific principal c… omponentsShow more	Dikilitas O et al. Am J Hum Genet (2020) | Ext.
							Hispanic or Latin American:2,493 individuals	Reported Trait: Coronary heart disease (incident and prevalent)	OR: 1.2 [1.06, 1.35], AUROC: 0.769	age at first EHR record, duration of EHR, sex, eMERGE site, first five ancestry-specific principal c… omponentsShow more	Dikilitas O et al. Am J Hum Genet (2020) | Ext.

							European:39,758 individuals	Reported Trait: Incident coronary heart disease	HR: 1.2 [1.15, 1.25], C-index: 0.736	sex, eMERGE site, diabetes, hypertension, hyperlipidemia, statin use, first 5 ancestry-specific prin... cipal componentsShow more	Dikilitas O et al. Am J Hum Genet (2020) | Ext.
							African:7,070 individuals	Reported Trait: Incident coronary heart disease	HR: 1.05 [0.94, 1.18], C-index: 0.704	sex, eMERGE site, diabetes, hypertension, hyperlipidemia, statin use, first 5 ancestry-specific prin... cipal componentsShow more	Dikilitas O et al. Am J Hum Genet (2020) | Ext.
							Hispanic or Latin American:2,194 individuals	Reported Trait: Incident coronary heart disease	HR: 1.12 [0.93, 1.36], C-index: 0.708	sex, eMERGE site, diabetes, hypertension, hyperlipidemia, statin use, first 5 ancestry-specific prin... cipal componentsShow more	Dikilitas O et al. Am J Hum Genet (2020) | Ext.
							European:26,203 individuals	Reported Trait: Incident coronary heart disease hospitalization or death	Hazard Ratio (HR, top 25% vs bottom 25%): 1.55 [1.38, 1.76]	Age as timescale, sex, region of residence, calendar year, study batch, PCs(1-10)	Martikainen P et al. J Epidemiol Community Health (2021) | Ext.
							European:26,203 individuals	Reported Trait: Incident coronary heart disease hospitalization or death	Hazard Ratio (HR, top 25% vs bottom 25%): 1.56 [1.38, 1.77]	Age as timescale, sex, region of residence, calendar year, study batch, PCs(1-10), education	Martikainen P et al. J Epidemiol Community Health (2021) | Ext.
							European:26,203 individuals	Reported Trait: Incident coronary heart disease hospitalization or death	Hazard Ratio (HR, top 25% vs bottom 25%): 1.53 [1.35, 1.73]	Age as timescale, sex, region of residence, calendar year, study batch, PCs(1-10), smoking, alcohol ... use, body mass index, high-density lipoprotein and total cholesterol, blood pressure, diabetesShow more	Martikainen P et al. J Epidemiol Community Health (2021) | Ext.
PGS000057	Coronary heart disease	coronary artery disease	57	Hard thresholding with r2 > 0.8	Multiancestry:502,601 individuals	Natarajan P et al. Circulation (2017)	2,440 individuals	Reported Trait: Coronary heart disease (incident)	HR (highest vs. lowest quintile of PGS): 1.66 [1.21, 2.29]	age, sex, diabetes meliitus status, smoking status, LDL cholesterol, HDL cholesterol, systolic blood... pressure, antihypertensive medication status, family history of CHDShow more	Natarajan P et al. Circulation (2017)
							1,154 individuals	Reported Trait: Coronary artery calcification	OR: 1.32 [1.04, 1.68], OR (highest vs. lowest quintile of PGS): 2.51 [1.08, 5.85]	age, sex, diabetes meliitus status, smoking status, LDL cholesterol, HDL cholesterol, systolic blood... pressure, antihypertensive medication status, family history of CHDShow more	Natarajan P et al. Circulation (2017)
							4,392 individuals	Reported Trait: Carotid artery plaque burden	β: 1.097 [1.022, 1.178]	age, sex, diabetes meliitus status, smoking status, LDL cholesterol, HDL cholesterol, systolic blood... pressure, antihypertensive medication status, family history of CHDShow more	Natarajan P et al. Circulation (2017)
PGS000059	Coronary heart disease	coronary artery disease	46	Literature-derived SNP selection, OR	Multiancestry:540,176 individuals	Hajek C et al. Circ Genom Precis Med (2018)	European:1,206 individuals	Reported Trait: Incident coronary heart disease	HR (top vs. bottom quartiles of GRS): 1.92 [1.19, 3.11]<br>p-value (association between risk and incidence): 0.029		Hajek C et al. Circ Genom Precis Med (2018)
							European:1,320 individuals	Reported Trait: Incident coronary heart disease	HR (top vs. bottom quartiles of GRS): 0.76 [0.41, 1.39]<br>p-value (association between risk and incidence): 0.31		Hajek C et al. Circ Genom Precis Med (2018)
**PGS000200**	**Coronary heart disease**	**coronary artery disease**	**28**	**GWAS, OR**	**European and South Asian:126,029 individuals**	**Tikkanen E et al. Arterioscler Thromb Vasc Biol (2013)**	**European:24,124 individuals**	**Reported Trait: Incident cardiovascular disease**	**HR: 1.18 [1.12, 1.24]**	**sex, total cholesterol, high-density lipoprotein–cholesterol, body mass index, systolic blood pressu... re, antihypertensive treatment, smoking, type 2 diabetes mellitusShow more**	**Tikkanen E et al. Arterioscler Thromb Vasc Biol (2013)**
							**European:24,124 individuals**	**Reported Trait: Incident coronary heart disease**	**HR: 1.27 [1.2, 1.35]**	**sex, total cholesterol, high-density lipoprotein–cholesterol, body mass index, systolic blood pressu... re, antihypertensive treatment, smoking, type 2 diabetes mellitusShow more**	**Tikkanen E et al. Arterioscler Thromb Vasc Biol (2013)**
							**European:24,124 individuals**	**Reported Trait: Incident acute coronary syndrome**	**HR: 1.27 [1.18, 1.37]**	**sex, total cholesterol, high-density lipoprotein–cholesterol, body mass index, systolic blood pressu... re, antihypertensive treatment, smoking, type 2 diabetes mellitusShow more**	**Tikkanen E et al. Arterioscler Thromb Vasc Biol (2013)**
							**European:24,124 individuals**	**Reported Trait: Incident cardiovascular disease**	**C-index: 0.84**	**sex, total cholesterol, high-density lipoprotein-cholesterol, body mass index, systolic blood pressu... re, antihypertensive treatment, smoking, type 2 diabetes mellitus, family historyShow more**	**Tikkanen E et al. Arterioscler Thromb Vasc Biol (2013)**
							**European:24,124 individuals**	**Reported Trait: Incident coronary heart disease**	**C-index: 0.856**	**sex, total cholesterol, high-density lipoprotein-cholesterol, body mass index, systolic blood pressu... re, antihypertensive treatment, smoking, type 2 diabetes mellitus, family historyShow more**	**Tikkanen E et al. Arterioscler Thromb Vasc Biol (2013)**
							**European:24,124 individuals**	**Reported Trait: Incident acute coronary syndrome**	**C-index: 0.859**	**sex, total cholesterol, high-density lipoprotein-cholesterol, body mass index, systolic blood pressu... re, antihypertensive treatment, smoking, type 2 diabetes mellitus, family historyShow more**	**Tikkanen E et al. Arterioscler Thromb Vasc Biol (2013)**
							**European:39,758 individuals**	**Reported Trait: Incident coronary heart disease**	**HR: 1.18 [1.13, 1.23], C-index: 0.697**	**sex, eMERGE site, first five ancestry-specific principal components**	**Dikilitas O et al. Am J Hum Genet (2020) | Ext.**
							**African:7,070 individuals**	**Reported Trait: Incident coronary heart disease**	**HR: 1.11 [0.99, 1.24], C-index: 0.652**	**sex, eMERGE site, first five ancestry-specific principal components**	**Dikilitas O et al. Am J Hum Genet (2020) | Ext.**
							**Hispanic or Latin American:2,194 individuals**	**Reported Trait: Incident coronary heart disease**	**HR: 1.14 [0.94, 1.37], C-index: 0.655**	**sex, eMERGE site, first five ancestry-specific principal components**	**Dikilitas O et al. Am J Hum Genet (2020) | Ext.**
							**European:45,645 individuals**	**Reported Trait: Coronary heart disease (incident and prevalent)**	**OR: 1.24 [1.21, 1.28], AUROC: 0.748**	**age at first EHR record, duration of EHR, sex, eMERGE site, first five ancestry-specific principal c... omponentsShow more**	**Dikilitas O et al. Am J Hum Genet (2020) | Ext.**
							**African:7,597 individuals**	**Reported Trait: Coronary heart disease (incident and prevalent)**	**OR: 1.07 [0.99, 1.16], AUROC: 0.763**	**age at first EHR record, duration of EHR, sex, eMERGE site, first five ancestry-specific principal c... omponentsShow more**	**Dikilitas O et al. Am J Hum Genet (2020) | Ext.**
							**Hispanic or Latin American:2,493 individuals**	**Reported Trait: Coronary heart disease (incident and prevalent)**	**OR: 1.27 [1.12, 1.42], AUROC: 0.771**	**age at first EHR record, duration of EHR, sex, eMERGE site, first five ancestry-specific principal c... omponentsShow more**	**Dikilitas O et al. Am J Hum Genet (2020) | Ext.**
							**European:39,758 individuals**	**Reported Trait: Incident coronary heart disease**	**HR: 1.17 [1.12, 1.22], C-index: 0.735**	**sex, eMERGE site, diabetes, hypertension, hyperlipidemia, statin use, first 5 ancestry-specific prin... cipal componentsShow more**	**Dikilitas O et al. Am J Hum Genet (2020) | Ext.**
							**African:7,070 individuals**	**Reported Trait: Incident coronary heart disease**	**HR: 1.11 [0.99, 1.25], C-index: 0.706**	**sex, eMERGE site, diabetes, hypertension, hyperlipidemia, statin use, first 5 ancestry-specific prin... cipal componentsShow more**	**Dikilitas O et al. Am J Hum Genet (2020) | Ext.**

							**Hispanic or Latin American:2,194 individuals**	**Reported Trait: Incident coronary heart disease**	**HR: 1.13 [0.93, 1.37], C-index: 0.709**	**sex, eMERGE site, diabetes, hypertension, hyperlipidemia, statin use, first 5 ancestry-specific prin... cipal componentsShow more**	**Dikilitas O et al. Am J Hum Genet (2020) | Ext.**
PGS000349	Coronary artery disease	coronary artery disease	70	GWAS with p<5e-8, log(HR)	Multincestry:589,596 individuals	Pechlivanis S et al. BMC Med Genet (2020)	European:4,041 individuals	Reported Trait: Incident Coronary Heart Disease	HR: 1.18 [1.06, 1.31]	age, sex	Pechlivanis S et al. BMC Med Genet (2020)
							European:3,748 individuals	Reported Trait: Incident Coronary Heart Disease	HR: 1.18 [1.06, 1.31]	age, sex, cardiovascular risk factors (systolic blood pressure, antihypertensive medication, smoking... , LDL-cholestrol, HDL-cholesterol, lipid lowering medication, BMI and diabetes) and coronary artery calcification.Show more	Pechlivanis S et al. BMC Med Genet (2020)
							European:1,919 individuals	Reported Trait: Incident Coronary Heart Disease in males	HR: 1.25 [1.1, 1.42]	age	Pechlivanis S et al. BMC Med Genet (2020)
							European:1,765 individuals	Reported Trait: Incident Coronary Heart Disease in males	HR: 1.23 [1.07, 1.41]	age, cardiovascular risk factors (systolic blood pressure, antihypertensive medication, smoking, LDL... -cholestrol, HDL-cholesterol, lipid lowering medication, BMI and diabetes) and coronary artery calcification.Show more	Pechlivanis S et al. BMC Med Genet (2020)
							European:2,560 individuals	Reported Trait: Incident Coronary Heart Disease in indiviuals with coronary artery calcification > 0	HR: 1.21 [1.08, 1.36]	age, sex, cardiovascular risk factors (systolic blood pressure, antihypertensive medication, smoking... , LDL-cholestrol, HDL-cholesterol, lipid lowering medication, BMI and diabetes).Show more	Pechlivanis S et al. BMC Med Genet (2020)
							European:1,426 individuals	Reported Trait: Incident Coronary Heart Disease in males with coronary artery calcification > 0	HR: 1.26 [1.09, 1.46]	age, cardiovascular risk factors (systolic blood pressure, antihypertensive medication, smoking, LDL... -cholestrol, HDL-cholesterol, lipid lowering medication, BMI and diabetes).Show more	Pechlivanis S et al. BMC Med Genet (2020)
							European:4,041 individuals	Reported Trait: Coronary artery calcification	OR: 1.18 [1.1, 1.27]	age, sex, cardiovascular risk factors (systolic blood pressure, antihypertensive medication, smoking... , LDL-cholestrol, HDL-cholesterol, lipid lowering medication, BMI and diabetes).Show more	Pechlivanis S et al. BMC Med Genet (2020)
							European:3,748 individuals	Reported Trait: Coronary artery calcification	OR: 1.19 [1.1, 1.29]	age, sex, cardiovascular risk factors (systolic blood pressure, antihypertensive medication, smoking... , LDL-cholestrol, HDL-cholesterol, lipid lowering medication, BMI and diabetes).Show more	Pechlivanis S et al. BMC Med Genet (2020)
PGS000818	Coronary heart disease	coronary artery disease	138	GWAS with p<5e-8, ln(OR)	Multiancestry:1,022,858 individuals	Bauer A et al. Genet Epidemiol (2021)	European:2,909 individuals	Reported Trait: Incident coronary heart disease	C-index: 0.7571 [0.7234, 0.7908]	Age, sex, survey	Bauer A et al. Genet Epidemiol (2021)
							European:2,909 individuals	Reported Trait: Incident coronary heart disease	C-index: 0.792 [0.7622, 0.8219]	Age, sex, survey, Framingham risk score (diabetes status, current and former smoking status, systoli... c blood pressure, antihypertensive medication, HDL cholesterol, total cholesterol)Show more	Bauer A et al. Genet Epidemiol (2021)
							European:1,939 individuals	Reported Trait: Incident coronary heart disease	HR: 1.2341 [1.1137, 1.3676]	—	Bauer A et al. Genet Epidemiol (2021)
							European:1,939 individuals	Reported Trait: Incident coronary heart disease	HR: 1.2126 [1.0766, 1.3659]	Age, sex, survey	Bauer A et al. Genet Epidemiol (2021)
PGS002259	Stroke	stroke	534	metaPRS (elasticnet)	Multiancestry:2,442,550 individuals	Lu X et al. Neurology (2021)	East Asian: 41,006 individuals	Reported Trait: Incident stroke	HR: 1.28 [1.21, 1.36], Hazard Ratio (HR, highest vs lowest quintile): 1.99 [1.66, 2.38]	Sex	Lu X et al. Neurology (2021)
							East Asian: 41,006 individuals	Reported Trait: Incident ischemic stroke	HR: 1.29 [1.2, 1.39], Hazard Ratio (HR, highest vs lowest quintile): 2.13 [1.69, 2.69]	Sex	Lu X et al. Neurology (2021)
							East Asian: 41,006 individuals	Reported Trait: Incident hemorrhagic stroke	HR: 1.3 [1.17, 1.45], Hazard Ratio (HR, highest vs lowest quintile): 1.98 [1.41, 2.77]	Sex	Lu X et al. Neurology (2021)
							East Asian: 41,006 individuals	Reported Trait: Incident stroke	Hazard ratio (HR, high vs low tertile): 3.01 [2.03, 4.45]	Sex, cohort	Cui Q et al. Sci China Life Sci (2023) | Ext.
							East Asian: 41,006 individuals	Reported Trait: Incident stroke with high clinical risk	Hazard ratio (HR, high vs low tertile): 2.12 [1.38, 3.27]	Sex, cohort	Cui Q et al. Sci China Life Sci (2023) | Ext.
PGS004321	Coronary heart disease	coronary artery disease	27	GWAS, OR	Multiancestry:63,731 individuals	Marston NA et al. Circulation (2019)	European:14,298 individuals	Reported Trait: Major vascular events (placebo arm)	HR: 1.1 [1.03, 1.18]		Marston NA et al. Circulation (2019)
							European:14,298 individuals	Reported Trait: Major coronary events (placebo arm)	HR: 1.17 [1.08, 1.26]		Marston NA et al. Circulation (2019)
							European:14,298 individuals	Reported Trait: Major vascular events (evolocumab vs placebo)	p-value (pvalue, evolocumab and high PRS vs. placebo and low PRS): 0.07		Marston NA et al. Circulation (2019)
PGS004596	Coronary heart disease	coronary artery disease	64	GWAS, beta	Multiancestry:187,599 individuals	Peng H et al. Nutrients (2023)	Multiancestry:13,348 individuals	Reported Trait: Incident coronary artery disease in breast cancer survivors	Hazard ratio (HR, top 50% vs bottom 50% of PRS): 1.36 [1.1, 1.67]	Age at diagnosis of breast cancer, race, Townsend Deprivation Index, diabetes, hypertension, antihyp... ertensive medications, insulin treatment, lipid treatments, hormone replacement therapy, menopause, surgical treatment of breast cancer, genetic testing batches, 10 PCsShow more	Peng H et al. Nutrients (2023)
							Multiancestry:13,348 individuals	Reported Trait: Incident coronary artery disease in breast cancer survivors with lifestyle	Hazard ratio (HR, unhealthy lifestyle and PRS in top 50% vs healthy lifestyle and PRS in bottom 50%): 0.37 [0.24, 0.56]	Age at diagnosis of breast cancer, race, Townsend Deprivation Index, diabetes, hypertension, antihyp... ertensive medications, insulin treatment, lipid treatments, hormone replacement therapy, menopause, surgical treatment of breast cancer, genetic testing batches, 10 PCsShow more	Peng H et al. Nutrients (2023)
PGS004919	Coronary artery disease	coronary artery disease	50	GWAS, log(OR)	South Asia and European:170,575 individuals	Sjögren M et al. Int J Cardiol Heart Vasc (2019)	23,594 individuals	Reported Trait: Number of hospitilizations for any cause	Incidence Rate Ratio (IRR, top vs bottom PGS quintiles): 1.1 [1.04, 1.16]	Age, sex, follow up time, hypertension, prevalent diabetes, smoking, ApoA1, ApoB	Sjögren M et al. Int J Cardiol Heart Vasc (2019)
							23,594 individuals	Reported Trait: Number of cardiovascular-related hospitalizations	Incidence Rate Ratio (IRR, top vs bottom PGS quintiles): 1.31 [1.2, 1.43]	Age, sex, follow up time	Sjögren M et al. Int J Cardiol Heart Vasc (2019)
							23,594 individuals	Reported Trait: Number of hospitilization days for any cause	Incidence Rate Ratio (IRR, top vs bottom PGS quintiles): 1.17 [1.08, 1.26]	Age, sex, follow up time, hypertension, prevalent diabetes, smoking, ApoA1 and ApoB	Sjögren M et al. Int J Cardiol Heart Vasc (2019)

							23,594 individuals	Reported Trait: Any hospitilization event	Odds Ratio (OR, top vs bottom PGS quintiles): 1.18 [1.07, 1.3]	Age, sex, follow up time	Sjögren M et al. Int J Cardiol Heart Vasc (2019)
							23,594 individuals	Reported Trait: Cardiovascular-related death	Hazard Ratio (HR, top vs bottom PGS quintiles): 1.44 [1.25, 1.66]	Age, sex, hypertension, prevalent diabetes, smoking, aPoA1, ApoB	Sjögren M et al. Int J Cardiol Heart Vasc (2019)
PGS000063	Triglycerides	triglyceride measurement	32	GWAS with p<5e-8, beta	European:96,598 individuals	Johnson L et al. PLoS One (2015)	European:2,063 individuals	Reported Trait: Serum triglyceride (TG) levels	β: 0.464, Beta (p-value): 1.71e-11	age, age^2, sex, GRS_HDL, GRS_LDL, GRS_TC	Johnson L et al. PLoS One (2015)
							East Asian:666 individuals	Reported Trait: Serum triglyceride (TG) levels	β: 0.337, Beta (p-value): 0.0107	age, age^2, sex, GRS_HDL, GRS_LDL, GRS_TC	Johnson L et al. PLoS One (2015)
							African:1,355 individuals	Reported Trait: Serum triglyceride (TG) levels	β: 0.047, Beta (p-value): 0.526	age, age^2, sex, GRS_HDL, GRS_LDL, GRS_TC	Johnson L et al. PLoS One (2015)
							Hispanic or Latin American:1,256 individuals	Reported Trait: Serum triglyceride (TG) levels	β: 0.5, Beta (p-value): 1.09e-10	age, age^2, sex, GRS_HDL, GRS_LDL, GRS_TC	Johnson L et al. PLoS One (2015)
PGS000066	Triglycerides (TG)	triglyceride measurement	101	GWAS with MAF < 0.01, Imputation r2 < 0.8, clumping based on r2 > 0.1, overlap of SNPs between study cohorts, OR	Multiancestry:331,368 individuals	Kuchenbaecker K et al. Nat Commun (2019)	European:9,962 individuals	Reported Trait: Serum triglyceride (TG) levels	correlation (r): 0.204 [0.183, 0.223]	age, sex	Kuchenbaecker K et al. Nat Commun (2019)
							European:1,641 individuals	Reported Trait: Serum triglyceride (TG) levels	correlation (r): 0.235 [0.176, 0.294]	age, sex	Kuchenbaecker K et al. Nat Commun (2019)
							European:1,945 individuals	Reported Trait: Serum triglyceride (TG) levels	correlation (r): 0.234 [0.175, 0.293]	age, sex	Kuchenbaecker K et al. Nat Commun (2019)
							African:6,407 individuals	Reported Trait: Serum triglyceride (TG) levels	correlation (r): 0.063 [0.038, 0.089]	age, sex	Kuchenbaecker K et al. Nat Commun (2019)
							East Asian:21,295 individuals	Reported Trait: Serum triglyceride (TG) levels	correlation (r): 0.139 [0.1, 0.178]	age, sex, region, 20 PCs of genetic ancestry	Kuchenbaecker K et al. Nat Commun (2019)
**PGS000312**	**Triglycerides**	**triglyceride measurement**	**190**	**GWAS lead variants with r2 < 0.1, beta**	**European:393,811 individuals**	**Xie T et al. Circ Genom Precis Med (2020)**	**European:1,354 individuals**	**Reported Trait: Triglycerides (mmol/l)**	**R^2: 0.0656**	**Sex, age, age^2**	**Xie T et al. Circ Genom Precis Med (2020)**
PGS003401	Triglyceride levels	triglyceride measurement	108	GWAS with r2 < 0.1, beta	European:237,050 individuals	Pieri K et al. Int J Cardiol (2022)	European:460,037 individuals	Reported Trait: Plasma triglyceride concentration	β: 0.142 (0.000753)		Pieri K et al. Int J Cardiol (2022)
							European:6,952 individuals	Reported Trait: Plasma triglyceride concentration	β: 0.122 (0.00495)		Pieri K et al. Int J Cardiol (2022)
PGS003854	Triglycerides	triglyceride measurement	40	GWAS, beta	East Asian:74,297 individuals	Li J et al. JAMA Netw Open (2023)	East Asian:37,317 individuals	Reported Trait: Estimated annual change of triglycerides	p-value (inferior to): 0.001		Li J et al. JAMA Netw Open (2023)
							East Asian:15,664 individuals	Reported Trait: Estimated annual change of triglycerides	p-value (inferior to): 0.001		Li J et al. JAMA Netw Open (2023)
							East Asian:21,653 individuals	Reported Trait: Estimated annual change of triglycerides	p-value (inferior to): 0.001		Li J et al. JAMA Netw Open (2023)
PGS004916	Triglycerides	triglyceride measurement	223	GWAS, beta	Multiancestry:297,626 individuals	Trinder M et al. Arterioscler Thromb Vasc Biol (2019)	European:389,971 individuals	Reported Trait: Triglyceride levels	R^2: 0.1	Age, sex, genotyping array/batch, 4 PCs	Trinder M et al. Arterioscler Thromb Vasc Biol (2019)
PGS000062	Total cholesterol	total cholesterol measurement	52	GWAS with p<5e-8, beta	European:100,184 individuals	Johnson L et al. PLoS One (2015)	European:2,063 individuals	Reported Trait: Triglyceride levels	β: 20.69, Beta (p-value): 0.0108	age, age^2, sex, GRS_HDL, GRS_LDL, GRS_TG	Johnson L et al. PLoS One (2015)
							East Asian:666 individuals	Reported Trait: Triglyceride levels	β: 8.57, Beta (p-value): 0.583	age, age^2, sex, GRS_HDL, GRS_LDL, GRS_TG	Johnson L et al. PLoS One (2015)
							African:1,355 individuals	Reported Trait: Triglyceride levels	β: 1.99, Beta (p-value): 0.852	age, age^2, sex, GRS_HDL, GRS_LDL, GRS_TG	Johnson L et al. PLoS One (2015)
							Hispanic or Latin American:1,256 individuals	Reported Trait: Triglyceride levels	β: -2.62, Beta (p-value): 0.815	age, age^2, sex, GRS_HDL, GRS_LDL, GRS_TG	Johnson L et al. PLoS One (2015)
**PGS000311**	**Total cholesterol**	**total cholesterol measurement**	**234**	**GWAS with r2 < 0.1, beta**	**European:393,811 individuals**	**Xie T et al. Circ Genom Precis Med (2020)**	**European:1,354 individuals**	**Reported Trait: Total cholesterol (mmol/l)**	**R^2: 0.1295**	**Sex, age, age^2**	**Xie T et al. Circ Genom Precis Med (2020)**
PGS003853	Total cholesterol	total cholesterol measurement	60	GWAS, beta	East Asian:78,690 individuals	Li J et al. JAMA Netw Open (2023)	East Asian:37,317 individuals	Reported Trait: Estimated annual change of total cholesterol	p-value (inferior to): 0.001		Li J et al. JAMA Netw Open (2023)
							East Asian:15,664 individuals	Reported Trait: Estimated annual change of total cholesterol	p-value (inferior to): 0.001		Li J et al. JAMA Netw Open (2023)
							East Asian:21,653 individuals	Reported Trait: Estimated annual change of total cholesterol	p-value (inferior to): 0.001		Li J et al. JAMA Netw Open (2023)
PGS000192	Cholesterol	low density lipoprotein cholesterol measurement,<br>high density lipoprotein cholesterol measurement	9	Curated variant associations, Unweighted	Multiancestry:30,795 individuals	Kathiresan S et al. N Engl J Med (2008)	European:4,232 individuals	Reported Trait: Low-density lipoprotein (LDL) levels	Association p-value: 3.00e-24		Kathiresan S et al. N Engl J Med (2008)
							European:4,232 individuals	Reported Trait: High-density lipoprotein (HDL) levels	Association p-value: 2.00e-18		Kathiresan S et al. N Engl J Med (2008)
							European:4,232 individuals	Reported Trait: Incident cardiovascular event	AUROC: 0.8, Hazard Ratio (HR; per allele): 1.15 [1.07, 1.24]	age, sex, family history of MI, LDL cholesterol, HDL cholesterol, triglycerides, blood pressure, bod... y mass index, diabetes status, smoking status, CRP, lipid lowering medicationShow more	Kathiresan S et al. N Engl J Med (2008)
PGS000060	HDL cholesterol	low density lipoprotein cholesterol measurement	46	GWAS with p<5e-8, beta	East Asian:99,900 individuals	Johnson L et al. PLoS One (2015)	European:2,063 individuals	Reported Trait: Serum high-density lipoprotein (HDL) levels	β: 8.39, Beta (p-value): 2.76e-06	age, age^2, sex, GRS_LDL, GRS_TC, GRS_TG	Johnson L et al. PLoS One (2015)
							East Asian:666 individuals	Reported Trait: Serum high-density lipoprotein (HDL) levels	β: 15.58, Beta (p-value): 2.17e-07	age, age^2, sex, GRS_LDL, GRS_TC, GRS_TG	Johnson L et al. PLoS One (2015)
							African:1,355 individuals	Reported Trait: Serum high-density lipoprotein (HDL) levels	β: 7.99, (p-value): 0.00036	age, age^2, sex, GRS_LDL, GRS_TC, GRS_TG	Johnson L et al. PLoS One (2015)
							Hispanic or Latin American:1,256 individuals	Reported Trait: Serum high-density lipoprotein (HDL) levels	β: 10.97, Beta (p-value): 6.48e-10	age, age^2, sex, GRS_LDL, GRS_TC, GRS_TG	Johnson L et al. PLoS One (2015)
PGS000064	HDL cholesterol	low density lipoprotein cholesterol measurement	120	GWAS with MAF < 0.01, Imputation r2 < 0.8, clumping based on r2 > 0.1, overlap of SNPs between study cohorts, OR	Multiancestry:331,368 individuals	Kuchenbaecker K et al. Nat Commun (2019)	European:9,962 individuals	Reported Trait: Serum high-density lipoprotein (HDL) levels	correlation (r): 0.285 [0.265, 0.305]	age, sex	Kuchenbaecker K et al. Nat Commun (2019)

							European:1,641 individuals	Reported Trait: Serum high-density lipoprotein (HDL) levels	correlation (r): 0.279 [0.222, 0.336]	age, sex	Kuchenbaecker K et al. Nat Commun (2019)
							European:1,945 individuals	Reported Trait: Serum high-density lipoprotein (HDL) levels	correlation (r): 0.268 [0.209, 0.327]	age, sex	Kuchenbaecker K et al. Nat Commun (2019)
							African:6,407 individuals	Reported Trait: Serum high-density lipoprotein (HDL) levels	correlation (r): 0.121 [0.098, 0.145]	age, sex	Kuchenbaecker K et al. Nat Commun (2019)
							East Asian:21,295 individuals	Reported Trait: Serum high-density lipoprotein (HDL) levels	correlation (r): 0.18 [0.145, 0.215]	age, sex, region, 20 PCs of genetic ancestry	Kuchenbaecker K et al. Nat Commun (2019)
**PGS000309**	**HDL cholesterol**	**low density lipoprotein cholesterol measurement**	**247**	**GWAS with r2 < 0.1, beta**	**European:393,811 individuals**	**Xie T et al. Circ Genom Precis Med (2020)**	**European:1,354 individuals**	**Reported Trait: High-density lipoprotein (mmol/l)**	**R^2: 0.1149**	**Sex, age, age^2**	**Xie T et al. Circ Genom Precis Med (2020)**
PGS003856	HDL cholesterol	low density lipoprotein cholesterol measurement	59		East Asian:81,045 individuals	Li J et al. JAMA Netw Open (2023)	East Asian:37,317 individuals	Reported Trait: Estimated annual change of HDL cholesterol	p-value (inferior to): 0.001		Li J et al. JAMA Netw Open (2023)
							East Asian:15,664 individuals	Reported Trait: Estimated annual change of HDL cholesterol	p-value (inferior to): 0.001		Li J et al. JAMA Netw Open (2023)
							East Asian:21,653 individuals	Reported Trait: Estimated annual change of HDL cholesterol	p-value (inferior to): 0.001		Li J et al. JAMA Netw Open (2023)
PGS004914	HDL cholesterol	low density lipoprotein cholesterol measurement	223		Multiancestry:297,626 individuals	Trinder M et al. Arterioscler Thromb Vasc Biol (2019)	European:357,202 individuals	Reported Trait: High-density lipoprotein cholesterol levels	R^2: 0.24	Age, sex, genotyping array/batch, 4 PCs	Trinder M et al. Arterioscler Thromb Vasc Biol (2019)
							European:407,558 individuals	Reported Trait: Any infectious hospitilization	HR: 0.94 [0.91, 0.98]	Age, sex, genotyping array/batch, 4 PCs	Trinder M et al. Arterioscler Thromb Vasc Biol (2019)
PGS000061	Low-density lipoprotein (LDL) cholesterol	low density lipoprotein cholesterol measurement	37	GWAS with p<5e-8, beta	European:95,454 individuals	Johnson L et al. PLoS One (2015)	European:2,063 individuals	Reported Trait: Serum low-density lipoprotein (LDL) levels	β: 15.0, Beta (p-value): 0.0352	age, age^2, sex, GRS_HDL, GRS_TC, GRS_TG	Johnson L et al. PLoS One (2015)
							East Asian:666 individuals	Reported Trait: Serum low-density lipoprotein (LDL) levels	β: 5.58, Beta (p-value): 0.697	age, age^2, sex, GRS_HDL, GRS_TC, GRS_TG	Johnson L et al. PLoS One (2015)
							African:1,355 individuals	Reported Trait: Serum low-density lipoprotein (LDL) levels	β: 30.04, Beta (p-value): 0.00282	age, age^2, sex, GRS_HDL, GRS_TC, GRS_TG	Johnson L et al. PLoS One (2015)
							Hispanic or Latin American:1,256 individuals	Reported Trait: Serum low-density lipoprotein (LDL) levels	β: 42.86, Beta (p-value): 2e-05	age, age^2, sex, GRS_HDL, GRS_TC, GRS_TG	Johnson L et al. PLoS One (2015)
PGS000065	LDL cholesterol	low density lipoprotein cholesterol measurement	103	GWAS with MAF < 0.01, Imputation r2 < 0.8, clumping based on r2 > 0.1, overlap of SNPs between study cohorts, OR	Multiancestry:331,368 individuals	Kuchenbaecker K et al. Nat Commun (2019)	European:9,962 individuals	Reported Trait: Serum low-density lipoprotein (LDL) levels	correlation (r): 0.274 [0.254, 0.294]	age, sex	Kuchenbaecker K et al. Nat Commun (2019)
							European:1,641 individuals	Reported Trait: Serum low-density lipoprotein (LDL) levels	correlation (r): 0.229 [0.172, 0.286]	age, sex	Kuchenbaecker K et al. Nat Commun (2019)
							European:1,945 individuals	Reported Trait: Serum low-density lipoprotein (LDL) levels	correlation (r): 0.29 [0.231, 0.349]	age, sex	Kuchenbaecker K et al. Nat Commun (2019)
							African:6,407 individuals	Reported Trait: Serum low-density lipoprotein (LDL) levels	correlation (r): 0.28 [0.257, 0.304]	age, sex	Kuchenbaecker K et al. Nat Commun (2019)
							East Asian:21,295 individuals	Reported Trait: Serum low-density lipoprotein (LDL) levels	correlation (r): 0.198 [0.161, 0.235]	age, sex, region, 20 PCs of genetic ancestry	Kuchenbaecker K et al. Nat Commun (2019)
PGS000115	LDL cholesterol	low density lipoprotein cholesterol measurement	223	GWAS, beta	Multiancestry:297,626 individuals	Trinder M et al. JAMA Cardiol (2020)	European:439,871 individuals	Reported Trait: Serum low density lipoprotein cholesterol (LDL-C) levels	β: 28.01 (0.18), R^2: 0.09	age, sex, 4 PCs of genetic ancestry, genotyping method (array and batch)	Trinder M et al. JAMA Cardiol (2020)
							East Asian:10,640 individuals	Reported Trait: Serum low density lipoprotein cholesterol (LDL-C) levels	β: 21.73 (1.25), R^2: 0.06	age, sex, 4 PCs of genetic ancestry, genotyping method (array and batch)	Trinder M et al. JAMA Cardiol (2020)
							African:4,680 individuals	Reported Trait: Serum low density lipoprotein cholesterol (LDL-C) levels	β: 17.4 (1.91), R^2: 0.04	age, sex, 4 PCs of genetic ancestry, genotyping method (array and batch)	Trinder M et al. JAMA Cardiol (2020)
							Multiancestry:455,191 individuals	Reported Trait: Serum low density lipoprotein cholesterol (LDL-C) levels	β: 27.78 (0.18), R^2: 0.09	age, sex, 4 PCs of genetic ancestry, genotyping method (array and batch)	Trinder M et al. JAMA Cardiol (2020)
							Multiancestry:47,845 individuals	Reported Trait: Cardiovascular disease events	Hazard Ratio (HR; top vs. bottom decile of risk): 1.35 [1.3, 1.4]	age, sex, 4 PCs of genetic ancestry, genotyping method (array and batch)	Trinder M et al. JAMA Cardiol (2020)
							European:33,787 individuals	Reported Trait: Uncontrolled hypercholesterolaemia	Odds Ratio (OR, top vs. bottom quintile): 2.78 [2.58, 3.0]	age, sex, socioeconomic characteristics (education, occupation, Townsend deprivation score, and coun... try of residence), metabolic and lifestyle CVD risk factors (smoking status, body mass index, physical activity in METS, and weekly alcohol consumption), family history of CVD (diagnosis at any age), and the first four principal components of genetic ancestry, genotyping array and systolic blood pressure at baselineShow more	Tapela NM et al. Eur J Prev Cardiol (2021) | Ext.
							European:33,787 individuals	Reported Trait: Incident major adverse cardiovascular events in statin treatment	Hazard Ratio (HR, top vs. bottom quintile): 1.03 [0.92, 1.14]	age, sex, socioeconomic characteristics (education, occupation, Townsend deprivation score, and coun... try of residence), metabolic and lifestyle CVD risk factors (smoking status, body mass index, physical activity in METS, and weekly alcohol consumption), family history of CVD (diagnosis at any age), and the first four principal components of genetic ancestry, genotyping array and systolic blood pressure at baselineShow more	Tapela NM et al. Eur J Prev Cardiol (2021) | Ext.
							European:33,787 individuals	Reported Trait: Incident myocardial infarction in statin treatment	Hazard Ratio (HR, top vs. bottom quintile): 1.08 [0.95, 1.23]	age, sex, socioeconomic characteristics (education, occupation, Townsend deprivation score, and coun... try of residence), metabolic and lifestyle CVD risk factors (smoking status, body mass index, physical activity in METS, and weekly alcohol consumption), family history of CVD (diagnosis at any age), and the first four principal components of genetic ancestry, genotyping array and systolic blood pressure at baselineShow more	Tapela NM et al. Eur J Prev Cardiol (2021) | Ext.
							European:33,787 individuals	Reported Trait: Incident stroke in statin treatment	Hazard Ratio (HR, top vs. bottom quintile): 0.93 [0.77, 1.12]	age, sex, socioeconomic characteristics (education, occupation, Townsend deprivation score, and coun... try of residence), metabolic and lifestyle CVD risk factors (smoking status, body mass index, physical activity in METS, and weekly alcohol consumption), family history of CVD (diagnosis at any age), and the first four principal components of genetic ancestry, genotyping array and systolic blood pressure at baselineShow more	Tapela NM et al. Eur J Prev Cardiol (2021) | Ext.

**PGS000310**	**LDL cholesterol**	**low density lipoprotein cholesterol measurement**	**194**	**GWAS with r2 < 0.1, beta**	**European:390,003 individuals**	**Xie T et al. Circ Genom Precis Med (2020)**	**European:1,354 individuals**	**Reported Trait: Low-density lipoprotein (mmol/l)**	**R^2: 0.1849**	**Sex, age, age^2**	**Xie T et al. Circ Genom Precis Med (2020)**
PGS000340	LDL cholesterol	low density lipoprotein cholesterol measurement	28	GWAS, beta	European:94,595 individuals	Trinder M et al. Circ Genom Precis Med (2020)	Multiancestry:1,120 individuals	Reported Trait: Low-density lipoprotein cholesterol levels in familial hypercholesterolemia mutation carriers	Beta (per 20% increase in PGS): 0.13 [0.072, 0.19]		Trinder M et al. Circ Genom Precis Med (2020)
							European:389,127 individuals	Reported Trait: Low-density lipoprotein cholesterol levels	β: 0.82 (0.006), R^2: 0.074	Age, sex	Trinder M et al. Circ Genom Precis Med (2020)
							Multiancestry:1,120 individuals	Reported Trait: Atherosclerotic cardiovascular disease in familial hypercholesterolemia mutation carriers	Odds Ratio (OR; top 20% vs. rest): 1.48 [1.02, 2.14]	sex	Trinder M et al. Circ Genom Precis Med (2020)
PGS000814	LDL cholesterol	low density lipoprotein cholesterol measurement	12	GWAS lead variants with p<5e-8, beta	European:95,454 individuals	Talmud PJ et al. Lancet (2013)	European:3,020 individuals	Reported Trait: Low-density lipoprotein (LDL) cholesterol	β: 0.33 [0.3, 0.37], R^2: 0.11		Talmud PJ et al. Lancet (2013)
							European:3,020 individuals	Reported Trait: Low-density lipoprotein (LDL) cholesterol	β: 0.34 [0.31, 0.38]	Sex, age, lipid-lowering drug use, body-mass index, diabetes status, smoking status, blood pressure	Talmud PJ et al. Lancet (2013)
							European:3,020 individuals	Reported Trait: Low-density lipoprotein cholesterol level >4.9mmol/L	Risk Ratio (RR, top 10% vs bottom 10%): 4.17 [3.01, 5.78]		Talmud PJ et al. Lancet (2013)
							European:3,660 individuals	Reported Trait: Low-density lipoprotein cholesterol level >4.9mmol/L in individuals who have familial hypercholestrolaemia and no known mutation	AUROC: 0.65 [0.62, 0.68]		Talmud PJ et al. Lancet (2013)
							Multiancestry:967 individuals	Reported Trait: Hypobetalipoproteinemia	Percentage of cases with polygenic etiology (%): 34.0		Rimbert A et al. Arterioscler Thromb Vasc Biol (2020) | Ext.
							Multiancestry:967 individuals	Reported Trait: Liver steatosis	Odds Ratio (OR, polygenic vs monogenic hypobetalipoproteinemia cases): 0.13 [0.1, 1.16]	Age, sex	Rimbert A et al. Arterioscler Thromb Vasc Biol (2020) | Ext.
							European:4,787 individuals	Reported Trait: Low-density lipoprotein cholesterol	AUROC: 0.65		Leal LG et al. Mol Genet Genomic Med (2020) | Ext.
							Multiancestry:1,519 individuals	Reported Trait: Polygenic hypercholesterolemia	AUROC: 0.59 [0.56, 0.62]		Olmastroni E et al. J Am Heart Assoc (2022) | Ext.
							European:353,166 individuals	Reported Trait: LDL-C concentration	R^2: 0.108 [0.105, 0.111]	Age, sex	Gratton J et al. Front Genet (2022) | Ext.
							African:7,082 individuals	Reported Trait: LDL-C concentration	R^2: 0.105 [0.086, 0.124]	Age, sex	Gratton J et al. Front Genet (2022) | Ext.
							South Asian:7,016 individuals	Reported Trait: LDL-C concentration	R^2: 0.049 [0.035, 0.063]	Age, sex	Gratton J et al. Front Genet (2022) | Ext.
							European:353,166 individuals	Reported Trait: LDL-C concentration >4.9 mmol/L	OR: 11.01 [10.08, 12.04]		Gratton J et al. Front Genet (2022) | Ext.
							African:7,082 individuals	Reported Trait: LDL-C concentration >4.9 mmol/L	OR: 10.54 [5.29, 21.67]		Gratton J et al. Front Genet (2022) | Ext.
							South Asian:7,016 individuals	Reported Trait: LDL-C concentration >4.9 mmol/L	OR: 6.64 [2.98, 15.22]		Gratton J et al. Front Genet (2022) | Ext.
							European:353,166 individuals	Reported Trait: Coronary heart disease	OR: 1.76 [1.56, 1.99]		Gratton J et al. Front Genet (2022) | Ext.
							African:7,082 individuals	Reported Trait: Coronary heart disease	OR: 2.26 [0.78, 7.22]		Gratton J et al. Front Genet (2022) | Ext.
							South Asian:7,016 individuals	Reported Trait: Coronary heart disease	OR: 1.24 [0.62, 2.53]		Gratton J et al. Front Genet (2022) | Ext.
							European:353,166 individuals	Reported Trait: Coronary heart disease	OR: 1.25 [1.13, 1.39]		Gratton J et al. Front Genet (2022) | Ext.
							African:7,082 individuals	Reported Trait: Coronary heart disease	OR: 1.09 [0.48, 2.58]		Gratton J et al. Front Genet (2022) | Ext.
							South Asian:7,016 individuals	Reported Trait: Coronary heart disease	OR: 1.98 [0.95, 4.25]		Gratton J et al. Front Genet (2022) | Ext.
							Greater Middle Eastern:6,140 individuals	Reported Trait: Probable vs. unlikely dyslipidemia	p: 0.0003		Gandhi GD et al. J Transl Med (2022) | Ext.
							Multiancestry:237 individuals	Reported Trait: Coronary artery calcium score >0 in potential clinical FH cases	Odds ratio (OR, >80 percentile vs <= 80 percentile): 8.05 [1.65, 39.29]	Smoking, hypertension, waist circumference and lipoprotein(a)	Borg SÁ et al. Atheroscler Plus (2022) | Ext.
							European:89,528 individuals	Reported Trait: LDL-c blood concentration	β: 0.25 [0.25, 0.26]	Age, BMI, sex, age	Vanhoye X et al. Transl Res (2022) | Ext.
PGS000875	LDL cholesterol	low density lipoprotein cholesterol measurement	36	Clumping and Thresholding (C+T) with R^2 = 0.078, p < 1e-20	European:94,595 individuals	Leal LG et al. Mol Genet Genomic Med (2020)	European:4,787 individuals	Reported Trait: Low-density lipoprotein cholesterol	R^2: 0.08	Age, gender, body mass index, ancestry differences captured by the first two components from multidi… mensional scalingShow more	Leal LG et al. Mol Genet Genomic Med (2020)
							European:4,787 individuals	Reported Trait: Severe hypercholesterolemia	Risk Ratio (RR, top 30% vs bottom 30%): 4.8 [2.6, 8.9]		Leal LG et al. Mol Genet Genomic Med (2020)
							European:4,787 individuals	Reported Trait: Low-density lipoprotein cholesterol	AUROC: 0.67		Leal LG et al. Mol Genet Genomic Med (2020)
PGS002274	LDL cholesterol	low density lipoprotein cholesterol measurement	279	GWAS with p<5e-8, beta	European:237,050 individuals	Groenland EH et al. Atherosclerosis (2022)	European:4,416 individuals	Reported Trait: Low-density lipoprotein cholesterol	β: 0.18 [0.15, 0.21]	Age, sex, the first 5 principal components, BMI, T2DM, smoking, alcohol use, systolic blood pressure, eGFR, triglycerides, lipid-lowering medication	Groenland EH et al. Atherosclerosis (2022)
PGS003403	LDL cholesterol	low density lipoprotein cholesterol measurement	28	GWAS, beta	European:94,595 individuals	Trinder M et al. J Am Coll Cardiol (2019)	European:626 individuals	Reported Trait: LDL-C levels	pvalue (High >=80 percentile vs lower		Trinder M et al. J Am Coll Cardiol (2019)
							European:626 individuals	Reported Trait: Cardiovascular disease events in patients with monogenic familial hypercholesterolemia	Adjusted Hazard Ratio (aHR; top 20% vs. remaining): 3.06 [1.56, 5.99]	age, sex, LDL-C, diabetes mellitus, and hypertension	Trinder M et al. J Am Coll Cardiol (2019)
							European:89,528 individuals	Reported Trait: LDL-c blood concentration	β: 0.18 [0.18, 0.18], AUROC: 0.6233 [0.617, 0.63], R^2: 0.1055	Age, BMI, sex, age	Vanhoye X et al. Transl Res (2022) | Ext.
PGS003404	LDL cholesterol	low density lipoprotein cholesterol measurement	10	GWAS, beta	European:19,840 individuals	Wang J et al. Arterioscler Thromb Vasc Biol (2016)	Multiancestry:313 individuals	Reported Trait: FH mutation-negative with severe hypercholesterolemia	OR: 3.02 [1.61, 5.68]		Wang J et al. Arterioscler Thromb Vasc Biol (2016)

							European:89,528 individuals	Reported Trait: LDL-c blood concentration	β: 0.16 [0.15, 0.16], AUROC: 0.6072 [0.6, 0.614], R^2: 0.09317	Age, BMI, sex, age	Vanhoye X et al. Transl Res (2022) | Ext.
PGS003855	LDL cholesterol	low density lipoprotein cholesterol measurement	44	GWAS, beta	East Asian:78,402 individuals	Li J et al. JAMA Netw Open (2023)	East Asian:37,317 individuals	Reported Trait: Estimated annual change of LDL cholesterol	p-value (inferior to): 0.001		Li J et al. JAMA Netw Open (2023)
							East Asian:15,664 individuals	Reported Trait: Estimated annual change of LDL cholesterol	p-value (inferior to): 0.001		Li J et al. JAMA Netw Open (2023)
							East Asian:21,653 individuals	Reported Trait: Estimated annual change of LDL cholesterol	p-value (inferior to): 0.001		Li J et al. JAMA Netw Open (2023)
PGS004915	LDL cholesterol	low density lipoprotein cholesterol measurement	223	GWAS, beta	Multiancestry:297,626 individuals	Trinder M et al. Arterioscler Thromb Vasc Biol (2019)	European:389,564 individuals	Reported Trait: Low-density lipoprotein cholesterol levels	R^2: 0.05	Age, sex, genotyping array/batch, 4 PCs	Trinder M et al. Arterioscler Thromb Vasc Biol (2019)
**PGS000305**	**Fasting glucose**	**fasting blood glucose measurement**	**31**	**GWAS withr2 > 0.1, beta**	**European:146,817 individuals**	**Xie T et al. Circ Genom Precis Med (2020)**	**European:1,354 individuals**	**Reported Trait: Fasting glucose (mmol/l)**	**R^2: 0.0367**	**Sex, age, age^2**	**Xie T et al. Circ Genom Precis Med (2020)**
PGS000306	Fasting glucose (body mass index adjusted)	BMI-adjusted fasting blood glucose measurement	19	GWAS withr2 > 0.1, beta	European:191,084 individuals	Xie T et al. Circ Genom Precis Med (2020)	European:1,354 individuals	Reported Trait: Fasting glucose (mmol/l)	R^2: 0.0095	Sex, age, age^2	Xie T et al. Circ Genom Precis Med (2020)
PGS000130	HbA1c	HbA1c measurement	19	GWAS, beta	European:88,335 individuals	Wheeler E et al. PLoS Med (2017)	European:37,357 individuals	Reported Trait: Incident type 2 diabetes	OR: 1.05 [1.04, 1.06]	age, sex	Wheeler E et al. PLoS Med (2017)
**PGS000131**	**HbA1c**	**HbA1c measurement**	**19**	**GWAS, beta**	**African:7,564 individuals**	**Wheeler E et al. PLoS Med (2017)**	**African:1,906 individuals**	**Reported Trait: Incident type 2 diabetes**	**OR: 1.00 [0.95, 1.05]**	**age, sex**	**Wheeler E et al. PLoS Med (2017)**
PGS000132	HbA1c	HbA1c measurement	19	GWAS, beta	East asian:18,472 individuals	Wheeler E et al. PLoS Med (2017)	East asian:5,073 individuals	Reported Trait: Incident type 2 diabetes	OR: 1.05 [1.02, 1.07]	age, sex	Wheeler E et al. PLoS Med (2017)
PGS000304	HbA1c	HbA1c measurement	43	GWAS withr2 > 0.1, beta	Multiancestry:121,963 individuals	Xie T et al. Circ Genom Precis Med (2020)	European:1,354 individuals	Reported Trait: HbA1c (%)	R^2: 0.0283	Sex, age, age^2	Xie T et al. Circ Genom Precis Med (2020)
							European:288 individuals	Reported Trait: HbA1c (%)	R^2: 0.0277	Sex, age, age^2	Xie T et al. Circ Genom Precis Med (2020)

Table S4 Performance of selected T2D and CVD PRS (binary traits) in the study cohort

PGS_id	Reported trait	Mapped Trait (EFO label)	Number of Variants	Match rate(%) INFO08	Total effect weight	Retained effect weight	Weight retention rate (%)	OR	Lower95%	Upper95%	p-value	FDR	AUC	Lower95%	Upper95%	Pseudo-R2	Liability-scale R2
PGS000031	Type 2 diabetes (T2D)	type 2 diabetes mellitus	62	69.4	5.625698	3.983937	70.8168	1.764314	1.253819	2.483268	0.001123423	0.03270257	0.6998311	0.66837	0.7312922	0.0330542	0.141836
PGS000032	Type 2 diabetes (based on SNPs involved in β-cell function)	type 2 diabetes mellitus	20	80	0.9637	0.7767	80.5956	4.293736	1.481284	12.43259	0.007238645	0.03894081	0.6989699	0.6673292	0.7306107	0.0323774	0.1389318
PGS000033	Type 2 diabetes (based on SNPs involved in insulin resistance)	type 2 diabetes mellitus	10	80	0.3976	0.3027	76.1318	14.07598	2.025306	98.14191	0.007536931	0.03894081	0.6999032	0.6681728	0.7316335	0.0323678	0.1388906
PGS000125	Type 2 diabetes (T2D)	type 2 diabetes mellitus	80	77.5	87	62	71.2644	1.031231	1.00397	1.059268	0.02450704	0.08139856	0.6978969	0.6661777	0.7296161	0.0319541	0.1371154
PGS000848	Type 2 diabetes (based on SNPs associated with adiposity)	type 2 diabetes mellitus	6	83.3	0.468	0.236	50.4274	1.590845	0.1947759	12.81651	0.6636833	0.6636833	0.697937	0.6661623	0.7297116	0.0309868	0.1329647
PGS000849	Type 2 diabetes (based on SNPs associated with impared lipid)	type 2 diabetes mellitus	3	100	0.300487	0.300487	100	1.782646	0.4398784	7.26488	0.4189978	0.5195573	0.697894	0.6660396	0.7297484	0.0310792	0.1333612
PGS000850	Type 2 diabetes (based on SNPs associated with insulin action)	type 2 diabetes mellitus	16	62.5	0.888253	0.486495	54.7698	1.945213	0.4488881	8.493845	0.3749151	0.5195573	0.6985492	0.6668448	0.7302535	0.031106	0.1334762
PGS000851	Type 2 diabetes (based on SNPs associated with insulin action/secretion)	type 2 diabetes mellitus	37	67.6	3.016614	1.667403	55.274	1.324851	0.6752773	2.604213	0.4138076	0.5195573	0.6983584	0.6668617	0.7298552	0.0310821	0.1333737
PGS000852	Type 2 diabetes (based on SNPs associated with insulin secreation)	type 2 diabetes mellitus	8	87.5	0.743096	0.565244	76.066	2.109716	0.7210065	6.000667	0.1670454	0.3236505	0.698215	0.6663229	0.7301072	0.0313213	0.1344001
PGS000853	Type 2 diabetes (based on SNPs associated with insulin secretion)	type 2 diabetes mellitus	21	76.2	1.930009	1.619116	83.8916	1.627925	1.044755	2.505458	0.02888336	0.08139856	0.7004331	0.6686025	0.7322638	0.0318673	0.136743
PGS000854	Type 2 diabetes (based on SNPs associated with beta cell function)	type 2 diabetes mellitus	27	63	13.47	8.56	63.5486	1.158892	1.055344	1.273731	0.002109843	0.03270257	0.7022297	0.671211	0.7332484	0.0328499	0.1409593
PGS000855	Type 2 diabetes (based on SNPs associated with lipodystrophy)	type 2 diabetes mellitus	18	77.8	10.33	7.99	77.3475	1.035041	0.93641	1.1451	0.5020487	0.5521616	0.6985936	0.6668927	0.7302944	0.0310391	0.1331892
PGS000856	Type 2 diabetes (based on SNPs associated with liver lipids)	type 2 diabetes mellitus	3	100	1.61	1.61	100	1.188499	0.9904384	1.429155	0.06475959	0.1544267	0.7003582	0.6689187	0.7317976	0.0316334	0.1357393
PGS000857	Type 2 diabetes (based on SNPs associated with obesity)	type 2 diabetes mellitus	4	75	1.27	0.97	76.378	0.6551176	0.4507395	0.9545048	0.02706306	0.08139856	0.6982616	0.6665706	0.7299527	0.0319113	0.1369318
PGS000858	Type 2 diabetes (based on SNPs associated with proinsulin level)	type 2 diabetes mellitus	6	100	2.57	2.57	100	0.9370479	0.7714728	1.142978	0.5165383	0.5521616	0.6979653	0.6662808	0.7296499	0.0310323	0.13316
PGS002277	Type 2 diabetes (based on SNPs associated with insulin secretion)	type 2 diabetes mellitus	8	87.5	0.1677	0.0897	53.4884	0.04153495	7.59233E-05	24.85833	0.3258989	0.5195573	0.6982274	0.6665377	0.7299171	0.0311396	0.1336204
PGS002733	Type 2 diabetes (T2D)	type 2 diabetes mellitus	17	52.9	2.501238	1.299191	51.9419	1.485589	0.8779134	2.514631	0.1402855	0.2899234	0.698135	0.6662304	0.7300395	0.0313812	0.1346571
**PGS004106**	**Type 2 diabetes (T2D)**	**type 2 diabetes mellitus**	**35**	**68.6**	**4.017**	**2.995**	**74.5581**	**1.585709**	**1.114217**	**2.260499**	**0.01060997**	**0.04698701**	**0.6991789**	**0.6669405**	**0.7314173**	**0.0322521**	**0.1383942**
PGS004225	Type 2 diabetes	type 2 diabetes mellitus	46	82.6	7.11	6.12	86.0759	1.254401	1.072493	1.46717	0.004563	0.03555905	0.6970692	0.6651238	0.7290146	0.0325438	0.1396459
PGS004226	Type 2 diabetes	type 2 diabetes mellitus	50	74	4.95	3.78	76.3636	1.368322	1.034329	1.810423	0.02806228	0.08139856	0.6990122	0.6675272	0.7304971	0.0319069	0.1369129
PGS000010	Coronary heart disease	coronary artery disease	27	74.1	4.085694	2.755614	67.4454	0.6134518	0.2648221	1.417941	0.2531527	0.4359852	0.8067741	0.7521463	0.8614019	0.0123112	0.2313177
PGS000011	Coronary artery disease	coronary artery disease	50	74	4.8171	3.330142	69.1317	0.7053699	0.3270305	1.510091	0.3708041	0.5195573	0.8015889	0.7444081	0.8587698	0.0122095	0.2294069
PGS000057	Coronary heart disease	coronary artery disease	57	68.4	4.91	3.19	64.9695	0.4833931	0.1652142	1.394793	0.1814668	0.33091	0.797252	0.7368099	0.857694	0.0124112	0.2331966
**PGS000059**	**Coronary heart disease**	**coronary artery disease**	**46**	**58.7**	**5.59**	**3.14**	**56.1717**	**0.3583193**	**0.1753619**	**0.7262277**	**0.004588265**	**0.03555905**	**0.8105738**	**0.7579908**	**0.8631568**	**0.0136915**	**0.2572525**
PGS000200	Coronary heart disease	coronary artery disease	28	78.6	3.489286	2.725571	78.1125	0.7716856	0.3220527	1.832249	0.5587266	0.5773508	0.8023258	0.7452897	0.859362	0.0121161	0.2276519
PGS000349	Coronary artery disease	coronary artery disease	70	71.4	6.044436	3.184103	52.6782	0.7348245	0.3236741	1.660065	0.4599296	0.5280673	0.7999693	0.7411261	0.8588125	0.0121574	0.2284279
PGS000818	Coronary heart disease	coronary artery disease	138	57.2	9.1949	5.6658	61.6189	0.7283972	0.3535595	1.502851	0.3903165	0.5195573	0.8012358	0.744066	0.8584057	0.0121959	0.2291513
PGS002259	Stroke	stroke	534	50.2	8.687523	4.248129	48.8992	4.367906	0.9774975	19.91948	0.05511268	0.1423744	0.805189	0.7477306	0.8626475	0.0128006	0.2405132
PGS004321	Coronary heart disease	coronary artery disease	27	74.1	2.46883	1.79921	72.877	0.628031	0.1846564	2.055263	0.4491609	0.5280673	0.8046248	0.7488172	0.8604324	0.0121641	0.2285538
PGS004596	Coronary heart disease	coronary artery disease	64	73.4	4.5299	3.2304	71.3128	0.4610795	0.1805412	1.176801	0.1049993	0.2324985	0.7985415	0.7403616	0.8567214	0.0125779	0.2363288
PGS004919	Coronary artery disease	coronary artery disease	50	74	4.8171	3.330142	69.1317	0.7053699	0.3270305	1.510091	0.3708041	0.5195573	0.8015889	0.7444081	0.8587698	0.0122095	0.2294069

PGS_id	Reported trait	Mapped Trait (EFO label)	Number of Variants	Match rate(%) INFO03	Total effect weight	Retained effect weight	Weight retention rate (%)	OR	Lower95%	Upper95%	p-value	FDR	AUC	Lower95%	Upper95%	Pseudo-R2	Liability-scale R2
PGS000031	Type 2 diabetes (T2D)	type 2 diabetes mellitus	62	71	N/A	N/A	N/A	1.759807	1.252829	2.472466	0.001114206	0.02565791	0.7000553	0.6686135	0.7314971	0.0330569	0.1418476
PGS000032	Type 2 diabetes (based on SNPs involved in β-cell function)	type 2 diabetes mellitus	20	80	N/A	N/A	N/A	4.293736	1.481284	12.43259	0.007238645	0.03894081	0.6989699	0.6673292	0.7306107	0.0323774	0.1389318
PGS000033	Type 2 diabetes (based on SNPs involved in insulin resistance)	type 2 diabetes mellitus	10	80	N/A	N/A	N/A	14.07598	2.025306	98.14191	0.007536931	0.03894081	0.6999032	0.6681728	0.7316335	0.0323678	0.1388906
PGS000125	Type 2 diabetes (T2D)	type 2 diabetes mellitus	80	78.8	N/A	N/A	N/A	1.029672	1.002611	1.057492	0.03144111	0.09063139	0.6977841	0.6661124	0.7294558	0.0318686	0.1367486

PGS000848	Type 2 diabetes (based on SNPs associated with adiposity)	type 2 diabetes mellitus	6	83.3	N/A	N/A	N/A	1.590845	0.1947759	12.81651	0.6636833	0.6636833	0.697937	0.6661623	0.7297116	0.0309868	0.1329647
PGS000849	Type 2 diabetes (based on SNPs associated with impared lipid)	type 2 diabetes mellitus	3	100	N/A	N/A	N/A	1.782646	0.4398784	7.26488	0.4189978	0.5195573	0.697894	0.6660396	0.7297484	0.0310792	0.1333612
PGS000850	Type 2 diabetes (based on SNPs associated with insulin action)	type 2 diabetes mellitus	16	62.5	N/A	N/A	N/A	1.945213	0.4488881	8.493845	0.3749151	0.4842653	0.6985492	0.6668448	0.7302535	0.031106	0.1334762
PGS000851	Type 2 diabetes (based on SNPs associated with insulin action/secretion)	type 2 diabetes mellitus	37	75.7	N/A	N/A	N/A	1.291705	0.660611	2.529778	0.4547628	0.5221351	0.6981954	0.6666661	0.7297247	0.0310604	0.1332806
PGS000852	Type 2 diabetes (based on SNPs associated with insulin secretion)	type 2 diabetes mellitus	8	87.5	N/A	N/A	N/A	2.109716	0.7210065	6.000667	0.1670454	0.2901724	0.698215	0.6663229	0.7301072	0.0313213	0.1344001
PGS000853	Type 2 diabetes (based on SNPs associated with insulin secretion)	type 2 diabetes mellitus	21	85.7	N/A	N/A	N/A	1.601205	1.028341	2.462916	0.03452077	0.09063139	0.7004521	0.66863	0.7322742	0.0318097	0.1364958
PGS000854	Type 2 diabetes (based on SNPs associated with beta cell function)	type 2 diabetes mellitus	27	74.1	N/A	N/A	N/A	1.162212	1.058744	1.276917	0.001655349	0.02565791	0.7028223	0.6718427	0.7338019	0.0329399	0.1413455
PGS000855	Type 2 diabetes (based on SNPs associated with lipodystrophy)	type 2 diabetes mellitus	18	77.8	N/A	N/A	N/A	1.035041	0.93641	1.1451	0.5020487	0.5521616	0.6985936	0.6668927	0.7302944	0.0310391	0.1331892
PGS000856	Type 2 diabetes (based on SNPs associated with liver lipids)	type 2 diabetes mellitus	3	100	N/A	N/A	N/A	1.188499	0.9904384	1.429155	0.06475959	0.1544267	0.7003582	0.6689187	0.7317976	0.0316334	0.1357393
PGS000857	Type 2 diabetes (based on SNPs associated with obesity)	type 2 diabetes mellitus	4	75	N/A	N/A	N/A	0.6551176	0.4507395	0.9545048	0.02706306	0.09063139	0.6982616	0.6665706	0.7299527	0.0319113	0.1369318
PGS000858	Type 2 diabetes (based on SNPs associated with proinsulin level)	type 2 diabetes mellitus	6	100	N/A	N/A	N/A	0.9370479	0.7714728	1.142978	0.5165383	0.5521616	0.6979653	0.6662808	0.7296499	0.0310323	0.13316
PGS002277	Type 2 diabetes (based on SNPs associated with insulin secretion)	type 2 diabetes mellitus	8	87.5	N/A	N/A	N/A	0.04153495	7.59233E-05	24.85833	0.3258989	0.467737	0.6982274	0.6665377	0.7299171	0.0311396	0.1336204
PGS002733	Type 2 diabetes (T2D)	type 2 diabetes mellitus	17	70.6	N/A	N/A	N/A	1.402199	0.8676123	2.269123	0.1680065	0.2901724	0.6978744	0.665942	0.7298068	0.0313273	0.1344258
**PGS004106**	**Type 2 diabetes (T2D)**	**type 2 diabetes mellitus**	**35**	**77.1**	N/A	N/A	N/A	**1.537062**	**1.090053**	**2.169921**	**0.01435512**	**0.06357267**	**0.6992582**	**0.6670653**	**0.7314511**	**0.0321431**	**0.1379264**
PGS004225	Type 2 diabetes	type 2 diabetes mellitus	46	82.6	N/A	N/A	N/A	1.254401	1.072493	1.46717	0.004563	0.03555905	0.6970692	0.6651238	0.7290146	0.0325438	0.1396459
PGS004226	Type 2 diabetes	type 2 diabetes mellitus	50	74	N/A	N/A	N/A	1.368322	1.034329	1.810423	0.02806228	0.09063139	0.6990122	0.6675272	0.7304971	0.0319069	0.1369129
PGS000010	Coronary heart disease	coronary artery disease	27	74.1	N/A	N/A	N/A	0.6134518	0.2648221	1.417941	0.2531527	0.4130386	0.8067741	0.7521463	0.8614019	0.0123112	0.2313177
PGS000011	Coronary artery disease	coronary artery disease	50	76	N/A	N/A	N/A	0.6936338	0.3221932	1.482077	0.3470307	0.467737	0.8020265	0.7450219	0.8590311	0.0122265	0.2297263
PGS000057	Coronary heart disease	coronary artery disease	57	71.9	N/A	N/A	N/A	0.4491473	0.1562578	1.274751	0.1348212	0.2901724	0.7976396	0.7374519	0.8578273	0.0125033	0.2349271
**PGS000059**	**Coronary heart disease**	**coronary artery disease**	**46**	**58.7**	N/A	N/A	N/A	**0.3583193**	**0.1753619**	**0.7262277**	**0.004588265**	**0.03555905**	**0.8105738**	**0.7579908**	**0.8631568**	**0.0136915**	**0.2572525**
PGS000200	Coronary heart disease	coronary artery disease	28	78.6	N/A	N/A	N/A	0.7716856	0.3220527	1.832249	0.5587266	0.5773508	0.8023258	0.7452897	0.859362	0.0121161	0.2276519
PGS000349	Coronary artery disease	coronary artery disease	70	75.7	N/A	N/A	N/A	0.5817041	0.2680462	1.253967	0.1684872	0.2901724	0.8009135	0.7420257	0.8598012	0.0124327	0.2336006
PGS000818	Coronary heart disease	coronary artery disease	138	63	N/A	N/A	N/A	0.6733256	0.3313784	1.37029	0.274441	0.4253835	0.8021762	0.7456502	0.8587022	0.0122881	0.2308837
PGS002259	Stroke	stroke	534	60.5	N/A	N/A	N/A	4.692987	1.125932	20.0093	0.03508312	0.09063139	0.8061178	0.7484916	0.8637441	0.0129597	0.2435025
PGS004321	Coronary heart disease	coronary artery disease	27	74.1	N/A	N/A	N/A	0.628031	0.1846564	2.055263	0.4491609	0.5221351	0.8046248	0.7488172	0.8604324	0.0121641	0.2285538
PGS004596	Coronary heart disease	coronary artery disease	64	76.6	N/A	N/A	N/A	0.5264507	0.2124842	1.303487	0.1649903	0.2901724	0.7972328	0.7384877	0.8559778	0.0124366	0.2336739
PGS004919	Coronary artery disease	coronary artery disease	50	76	N/A	N/A	N/A	0.6936338	0.3221932	1.482077	0.3470307	0.467737	0.8020265	0.7450219	0.8590311	0.0122265	0.2297263

Table S5 Performance of selected TG, TC, HDL-C, LDL-C, FBS and HbA1c PRS (continuous traits) in the study cohort

PGS_id	Reported trait	Mapped Trait (EFO label)	Number of Variants	Match rate(%) INFO08	Total effect weight	Retained effect weight	Weight retention rate (%)	R2 INFO08	p-value R2 INFO08	FDR R2 INFO08	Beta	Lower95%	Upper95%	adjusted-R2 INFO08	p-value adjusted INFO08	FDR adjusted INFO08	Beta	Lower95%	Upper95%
PGS000063	Triglycerides	triglyceride measurement	32	78.1	2.4408	1.4957	61.2791	0.01300987	6.17802E-12	6.37E-12	-0.000308535	-0.000385788	-0.000231281	0.01461754	0.007059025	0.00727962	-0.00032882	-0.000408863	-0.000248776
PGS000066	Triglycerides (TG)	triglyceride measurement	101	77.2	3.69438	3.028012	81.9627	0.01699866	1.2759E-264	2.48E-264	0.000432387	0.00033768	0.000527094	0.017697	4.22993E-45	5.17E-45	0.000464072	0.0003659	0.000562244
**PGS000312**	**Triglycerides**	**triglyceride measurement**	**190**	**71.6**	**16.157**	**10.147**	**62.8025**	**0.03044961**	**5E-324**	**5E-324**	**0.001449824**	**0.00121352**	**0.001686128**	**0.03474752**	**7.854892e-313**	**2.592114e-312**	**0.001604255**	**0.001359761**	**0.001848748**
PGS003401	Triglyceride levels	triglyceride measurement	108	74.1	5.196	3.561	68.5335	0.02540289	5E-324	5E-324	0.000604393	0.000496338	0.000712448	0.02973527	4.8835E-289	1.34E-288	0.000662962	0.000551162	0.000774761
PGS003854	Triglycerides	triglyceride measurement	40	70	2.79497	2.1078	75.414	0.03060738	5E-324	5E-324	0.000733536	0.000614294	0.000852777	0.03326808	5.64492E-92	9.31E-92	0.00078886	0.000665382	0.000912339
PGS004916	Triglycerides	triglyceride measurement	223	69.1	5.373876	3.364339	62.6054	0.01385838	3.359646e-322	7.90505e-322	0.000350848	0.000265725	0.000435971	0.022616	8.64766E-53	1.10E-52	0.000370878	0.000283002	0.000458755
PGS000062	Total cholesterol	total cholesterol measurement	52	76.9	3.5978	2.5733	71.5243	0.0193485	7.62E-153	1.05E-152	-0.000889061	-0.00107149	-0.000706632	0.03707698	6.7122E-116	1.38E-115	-0.000903524	-0.001088533	-0.000718514
PGS000192	Cholesterol	low density lipoprotein cholesterol measurement,<br>high density lipoprotein cholesterol measurement	9	66.7	11	6	54.5455	0.001800168	3.3495E-237	6.14E-237	0.001619989	0.000575876	0.002664101	0.000593829	3.0946E-170	7.29E-170	0.00173814	0.000668908	0.002807373
**PGS000311**	**Total cholesterol**	**total cholesterol measurement**	**234**	**68.8**	**17.559**	**10.825**	**61.6493**	**0.05645229**	**2.63707E-89**	**3.22E-89**	**0.002754817**	**0.002429009**	**0.003080626**	**0.07763166**	**5.70043E-60**	**7.84E-60**	**0.002820532**	**0.00249085**	**0.003150214**
PGS003853	Total cholesterol	total cholesterol measurement	60	63.3	4.176725	2.825514	67.649	0.05876565	2.18E-120	2.77E-120	0.00172179	0.001522434	0.001921145	0.07007298	5.1918E-79	7.45E-79	0.001761606	0.001558807	0.001964405
PGS000060	HDL cholesterol	low density lipoprotein cholesterol measurement	46	80.4	2.8597	2.1732	75.994	0.03192367	1.08103E-31	1.23E-31	-0.002374854	-0.002752824	-0.001996884	0.04338735	1.34745E-17	1.53E-17	-0.002824614	-0.003225738	-0.002423491
PGS000064	HDL cholesterol	low density lipoprotein cholesterol measurement	120	72.5	4.518247	3.415713	75.5982	0.03694335	3.616540e-316	7.956388e-316	0.003233702	0.002756304	0.0037111	0.04132198	1.3177E-143	2.90E-143	0.0036406	0.00313209	0.004149109
**PGS000309**	**HDL cholesterol**	**low density lipoprotein cholesterol measurement**	**247**	**67.6**	**23.344**	**11.908**	**51.011**	**0.04446172**	**3.3285E-191**	**4.99E-191**	**0.007798494**	**0.006752617**	**0.008844371**	**0.05329971**	**3.22032E-85**	**5.06E-85**	**0.009102068**	**0.007990666**	**0.01021347**
PGS003856	HDL cholesterol	low density lipoprotein cholesterol measurement	59	66.1	4.622189	3.476999	75.2241	0.03907112	4.13945E-23	4.41E-23	0.004751754	0.004070257	0.005433251	0.05180037	6.54173E-12	6.96E-12	0.005703256	0.004980526	0.006425987
PGS004914	HDL cholesterol	low density lipoprotein cholesterol measurement	223	69.1	5.850705	3.94731	67.4672	0.0366849	1.05756E-87	1.25E-87	0.002625383	0.002236386	0.003014381	0.04538396	6.64302E-36	7.83E-36	0.003101326	0.002687911	0.00351474
PGS000061	Low-density lipoprotein (LDL) cholesterol	low density lipoprotein cholesterol measurement	37	73	2.7474	1.9692	71.675	0.01471908	1.412E-126	1.86E-126	-0.000769799	-0.000951245	-0.000588354	0.02507896	2.06697E-57	2.73E-57	-0.000812538	-0.000998349	-0.000626727
PGS000065	LDL cholesterol	low density lipoprotein cholesterol measurement	103	69.9	4.565619	3.256206	71.3201	0.06541742	5.9287E-214	1.03E-213	0.002126038	0.001893205	0.002358872	0.08649378	8.7637E-98	1.70E-97	0.002219849	0.001982868	0.00245683
PGS000115	LDL cholesterol	low density lipoprotein cholesterol measurement	223	69.1	5.713965	4.055509	70.9754	0.02423466	6.6766E-200	1.10E-199	0.000985706	0.000804995	0.001166417	0.03711018	5.83225E-94	1.07E-93	0.001020955	0.000836147	0.001205763
**PGS000310**	**LDL cholesterol**	**low density lipoprotein cholesterol measurement**	**194**	**68.6**	**17.02**	**10.731**	**63.0494**	**0.07160186**	**1.2433E-187**	**1.78E-187**	**0.003708779**	**0.003321779**	**0.004095778**	**0.09824274**	**5.79317E-85**	**8.69E-85**	**0.00388333**	**0.003490677**	**0.004275983**
PGS000340	LDL cholesterol	low density lipoprotein cholesterol measurement	28	85.7	1.922	1.576	81.9979	0.008002761	5E-324	5E-324	0.000439065	0.000299095	0.000579036	0.02009403	5E-324	5E-324	0.000507475	0.000364258	0.000650692
PGS000814	LDL cholesterol	low density lipoprotein cholesterol measurement	12	100	2.54	0.74	29.1339	0.05960454	5E-324	5E-324	0.001553113	0.001374398	0.001731827	0.06793292	5E-324	5E-324	0.001662008	0.00147884	0.001845176
PGS000875	LDL cholesterol	low density lipoprotein cholesterol measurement	36	55.6	4.3185	2.6416	61.1694	0.02675402	5E-324	5E-324	0.001431141	0.001181644	0.001680638	0.03526852	5E-324	5E-324	0.001518782	0.001263054	0.00177451
PGS002274	LDL cholesterol	low density lipoprotein cholesterol measurement	279	66.3	11.153797	7.594357	68.0876	0.01586281	3.50716E-28	3.86E-28	0.001226641	0.000948149	0.001505134	0.02131855	1.91724E-12	2.11E-12	0.001251622	0.000965713	0.001537531
PGS003403	LDL cholesterol	low density lipoprotein cholesterol measurement	28	85.7	1.922	1.576	81.9979	0.008002761	5E-324	5E-324	0.000439065	0.000299095	0.000579036	0.02009403	5E-324	5E-324	0.000507475	0.000364258	0.000650692
PGS003404	LDL cholesterol	low density lipoprotein cholesterol measurement	10	90	1.28	1.14	89.0625	0.004389484	5E-324	5E-324	0.000322007	0.000184647	0.000459368	0.009917967	5E-324	5E-324	0.000361494	0.000220476	0.000502512
PGS003855	LDL cholesterol	low density lipoprotein cholesterol measurement	44	56.8	3.888211	2.547248	65.5121	0.03117898	0.03612471	0.03612471	0.000935879	0.000785	0.001086758	0.03238113	0.1634235	0.1634235	0.000988956	0.000833725	0.001144188
PGS004915	LDL cholesterol	low density lipoprotein cholesterol measurement	223	69.1	5.715406	4.056915	70.9821	0.024228	7.4998E-200	1.18E-199	0.000986225	0.000805394	0.001167057	0.0371107	6.13519E-94	1.07E-93	0.001021442	0.000836511	0.001206372
**PGS000305**	**Fasting glucose**	**fasting blood glucose measurement**	**31**	**54.8**	**0.8426**	**0.4198**	**49.822**	**0.006428482**	**5E-324**	**5E-324**	**0.000260904**	**0.000167936**	**0.000353873**	**0.009353519**	**5E-324**	**5E-324**	**0.000259544**	**0.000163425**	**0.000355663**
PGS000306	Fasting glucose (body mass index adjusted)	BMI-adjusted fasting blood glucose measurement	19	52.6	0.397824	0.184379	46.3469	0.005045523	5E-324	5E-324	0.000131976	7.90971E-05	0.000184855	0.004950386	1.994256e-310	5.982768e-310	0.000132364	7.76092E-05	0.000187118
PGS000130	HbA1c	HbA1c measurement	19	84.2	0.284	0.219	77.1127	0.004666325	5E-324	5E-324	0.003271697	0.001957954	0.004585439	0.01687882	5E-324	5E-324	0.003538823	0.002164272	0.004913374
**PGS000131**	**HbA1c**	**HbA1c measurement**	**19**	**84.2**	**0.352**	**0.287**	**81.5341**	**0.003493703**	**5E-324**	**5E-324**	**0.003905384**	**0.002104645**	**0.005706123**	**0.02229605**	**6.323273e-313**	**2.318533e-312**	**0.004277433**	**0.002399648**	**0.006155218**
PGS000132	HbA1c	HbA1c measurement	19	84.2	0.319	0.2631	82.4765	0.007286184	9.6263E-299	1.99E-298	0.005110791	0.003458043	0.006763539	0.02027866	1.1513E-266	2.92E-266	0.005272508	0.003543977	0.00700104
PGS000304	HbA1c	HbA1c measurement	43	60.5	0.848255	0.472285	55.6773	0.005392302	5E-324	5E-324	0.005059265	0.003164731	0.006953799	0.01457626	5E-324	5E-324	0.006583474	0.004598205	0.008568743

PGS_id	Reported trait	Mapped Trait (EFO label)	Number of Variants	Match rate(%) INFO03	Total effect weight	Retained effect weight	Weight retention rate (%)	R2 INFO03	p-value R2 INFO03	FDR R2 INFO03	Beta	Lower95%	Upper95%	adjusted-R2 INFO03	p-value adjusted INFO03	FDR adjusted INFO03	Beta	Lower95%	Upper95%
PGS000063	Triglycerides	triglyceride measurement	32	81.2	N/A	N/A	N/A	0.01311789	1.58938E-64	1.75E-64	-0.000310466	-0.000387884	-0.000233048	0.01529836	7.24758E-11	7.47E-11	-0.000333396	-0.000413586	-0.000253206
PGS000066	Triglycerides (TG)	triglyceride measurement	101	79.2	N/A	N/A	N/A	0.01702845	1.8159E-275	3.52E-275	0.000433107	0.000338325	0.000527888	0.01828175	2.42712E-47	2.97E-47	0.000466809	0.000368587	0.000565031
**PGS000312**	**Triglycerides**	**triglyceride measurement**	**190**	**73.7**	N/A	N/A	N/A	**0.02998947**	**0**	**0**	**0.001469908**	**0.001228456**	**0.001711361**	**0.03475757**	**2.4145E-307**	**7.97E-307**	**0.001631433**	**0.001381673**	**0.001881193**
PGS003401	Triglyceride levels	triglyceride measurement	108	76.9	N/A	N/A	N/A	0.0256106	0	0	0.000621245	0.000510637	0.000731854	0.03084379	7.0641E-298	1.94E-297	0.000687639	0.000573251	0.000802027
PGS003854	Triglycerides	triglyceride measurement	40	80	N/A	N/A	N/A	0.03071303	0	0	0.000772358	0.000647027	0.000897689	0.03417188	4.64415E-91	7.30E-91	0.000832872	0.000703141	0.000962603
PGS004916	Triglycerides	triglyceride measurement	223	74	N/A	N/A	N/A	0.0142719	1.153306e-311	2.537273e-311	0.00035781	0.000272263	0.000443357	0.02402571	1.27711E-51	1.62E-51	0.000381241	0.000292972	0.00046951
PGS000062	Total cholesterol	total cholesterol measurement	52	76.9	N/A	N/A	N/A	0.0193485	7.62E-153	1.09E-152	-0.000889061	-0.00107149	-0.000706632	0.03707698	6.7122E-116	1.38E-115	-0.000903524	-0.001088533	-0.000718514
PGS000192	Cholesterol	low density lipoprotein cholesterol measurement,<br>high density lipoprotein cholesterol measurement	9	66.7	N/A	N/A	N/A	0.001800168	3.3495E-237	6.14E-237	0.001619989	0.000575876	0.002664101	0.000593829	3.0946E-170	7.29E-170	0.00173814	0.000668908	0.002807373
**PGS000311**	**Total cholesterol**	**total cholesterol measurement**	**234**	**72.6**	N/A	N/A	N/A	**0.05860717**	**3.0709E-102**	**3.90E-102**	**0.002830152**	**0.002501997**	**0.003158307**	**0.0762853**	**6.02018E-68**	**9.03E-68**	**0.00289816**	**0.002565482**	**0.003230839**
PGS003853	Total cholesterol	total cholesterol measurement	60	68.3	N/A	N/A	N/A	0.06152974	1.54886E-90	1.89E-90	0.0017906	0.001588269	0.001992932	0.07301175	7.16648E-58	9.85E-58	0.001816999	0.001611195	0.002022803
PGS000060	HDL cholesterol	low density lipoprotein cholesterol measurement	46	84.8	N/A	N/A	N/A	0.03160528	2.36211E-89	2.78E-89	-0.002375069	-0.002755022	-0.001995117	0.04663411	2.94646E-46	3.47E-46	-0.002871999	-0.003274476	-0.002469522
PGS000064	HDL cholesterol	low density lipoprotein cholesterol measurement	120	75	N/A	N/A	N/A	0.0373888	3.9261E-301	8.10E-301	0.003272225	0.00279212	0.003752329	0.04254176	3.2765E-136	7.21E-136	0.003706092	0.003194907	0.004217278

**PGS000309**	**HDL cholesterol**	**low density lipoprotein cholesterol measurement**	**247**	**70.9**	N/A	N/A	N/A	**0.04440206**	**6.3559E-141**	**8.74E-141**	**0.008327976**	**0.007210308**	**0.009445645**	**0.05405642**	**6.66246E-62**	**9.56E-62**	**0.009713451**	**0.008526272**	**0.01090063**
PGS003856	HDL cholesterol	low density lipoprotein cholesterol measurement	59	71.2	N/A	N/A	N/A	0.04265529	1.72515E-29	1.84E-29	0.005213264	0.004498848	0.005927681	0.0568192	4.89648E-15	5.39E-15	0.006218669	0.005461622	0.006975716
PGS004914	HDL cholesterol	low density lipoprotein cholesterol measurement	223	74	N/A	N/A	N/A	0.03712282	3.47971E-78	3.96E-78	0.002662884	0.002270739	0.003055028	0.0480363	1.7849E-31	2.03E-31	0.003180009	0.002763735	0.003596283
PGS000061	Low-density lipoprotein (LDL) cholesterol	low density lipoprotein cholesterol measurement	37	73	N/A	N/A	N/A	0.01471908	1.412E-126	1.86E-126	-0.000769799	-0.000951245	-0.000588354	0.02507896	2.06697E-57	2.73E-57	-0.000812538	-0.000998349	-0.000626727
PGS000065	LDL cholesterol	low density lipoprotein cholesterol measurement	103	73.8	N/A	N/A	N/A	0.06464245	1.3688E-212	2.38E-212	0.002120108	0.001886444	0.002353771	0.08598223	2.2667E-96	4.40E-96	0.00221815	0.001980356	0.002455944
PGS000115	LDL cholesterol	low density lipoprotein cholesterol measurement	223	74	N/A	N/A	N/A	0.02403063	3.6968E-196	5.81E-196	0.000985025	0.000803662	0.001166389	0.03697007	3.20361E-92	5.83E-92	0.001022118	0.000836649	0.001207588
**PGS000310**	**LDL cholesterol**	**low density lipoprotein cholesterol measurement**	**194**	**72.7**	N/A	N/A	N/A	**0.07499261**	**1.1336E-204**	**1.87E-204**	**0.003819728**	**0.003430951**	**0.004208504**	**0.1007575**	**7.92381E-92**	**1.31E-91**	**0.003995431**	**0.003600804**	**0.004390058**
PGS000340	LDL cholesterol	low density lipoprotein cholesterol measurement	28	85.7	N/A	N/A	N/A	0.008002761	5E-324	5E-324	0.000439065	0.000299095	0.000579036	0.02009403	5E-324	5E-324	0.000507475	0.000364258	0.000650692
PGS000814	LDL cholesterol	low density lipoprotein cholesterol measurement	12	100	N/A	N/A	N/A	0.05960454	5E-324	5E-324	0.001553113	0.001374398	0.001731827	0.06793292	5E-324	5E-324	0.001662008	0.00147884	0.001845176
PGS000875	LDL cholesterol	low density lipoprotein cholesterol measurement	36	61.1	N/A	N/A	N/A	0.02742089	5E-324	5E-324	0.001461764	0.001210107	0.00171342	0.03303567	5E-324	5E-324	0.001552671	0.001294343	0.001811
PGS002274	LDL cholesterol	low density lipoprotein cholesterol measurement	279	71	N/A	N/A	N/A	0.01566691	7.19864E-28	7.42E-28	0.001223745	0.000944174	0.001503315	0.02069197	1.21087E-12	1.29E-12	0.001246296	0.000959217	0.001533375
PGS003403	LDL cholesterol	low density lipoprotein cholesterol measurement	28	85.7	N/A	N/A	N/A	0.008002761	5E-324	5E-324	0.000439065	0.000299095	0.000579036	0.02009403	5E-324	5E-324	0.000507475	0.000364258	0.000650692
PGS003404	LDL cholesterol	low density lipoprotein cholesterol measurement	10	90	N/A	N/A	N/A	0.004389484	5E-324	5E-324	0.000322007	0.000184647	0.000459368	0.009917967	5E-324	5E-324	0.000361494	0.000220476	0.000502512
PGS003855	LDL cholesterol	low density lipoprotein cholesterol measurement	44	65.9	N/A	N/A	N/A	0.03420029	0.2906495	0.2906495	0.001028762	0.000870603	0.001186922	0.0375369	0.4491107	0.4491107	0.001069092	0.00090655	0.001231633
PGS004915	LDL cholesterol	low density lipoprotein cholesterol measurement	223	74	N/A	N/A	N/A	0.02402429	4.1138E-196	6.17E-196	0.000985545	0.000804062	0.001167028	0.03697085	3.35506E-92	5.83E-92	0.001022605	0.000837014	0.001208197
**PGS000305**	**Fasting glucose**	**fasting blood glucose measurement**	**31**	**71**	N/A	N/A	N/A	**0.006878122**	**5E-324**	**5E-324**	**0.000291477**	**0.00019098**	**0.000391973**	**0.01062593**	**5E-324**	**5E-324**	**0.000285821**	**0.000181962**	**0.000389681**
PGS000306	Fasting glucose (body mass index adjusted)	BMI-adjusted fasting blood glucose measurement	19	78.9	N/A	N/A	N/A	0.005209747	5E-324	5E-324	0.000166274	0.000100673	0.000231874	0.004971544	5E-324	5E-324	0.000161555	9.36226E-05	0.000229487
PGS000130	HbA1c	HbA1c measurement	19	94.7	N/A	N/A	N/A	0.004884704	5E-324	5E-324	0.003484223	0.002115633	0.004852814	0.01490387	5E-324	5E-324	0.003732597	0.002299065	0.005166129
**PGS000131**	**HbA1c**	**HbA1c measurement**	**19**	**94.7**	N/A	N/A	N/A	**0.00341895**	**1.609644e-314**	**3.794161e-314**	**0.003895861**	**0.002081014**	**0.005710707**	**0.01998989**	**2.7891E-282**	**7.08E-282**	**0.004242243**	**0.002347588**	**0.006136899**
PGS000132	HbA1c	HbA1c measurement	19	94.7	N/A	N/A	N/A	0.007502177	5E-324	5E-324	0.005207041	0.003547109	0.006866972	0.02004048	1.2051E-300	3.62E-300	0.005365115	0.003628671	0.007101559
PGS000304	HbA1c	HbA1c measurement	43	69.8	N/A	N/A	N/A	0.006158665	5E-324	5E-324	0.005665209	0.003676364	0.007654053	0.01142268	5E-324	5E-324	0.007226036	0.005137802	0.009314269

Table S6 Association between PRS and disease risk across quartiles for T2D and CVD

Trait	PRS	Quartile	OR	2.50%	97.50%	p-value	case	percentage	Cochran-Armitage test for trend
T2D	PGS000031	Q1	Ref	Ref	Ref	NA	56	4.59	0.001653
T2D	PGS000031	Q2	1.307201	0.9099071	1.887377	0.1491844	73	5.98	
T2D	PGS000031	Q3	1.299619	0.9072381	1.871992	0.1552459	77	6.32	
T2D	PGS000031	Q4	1.734138	1.227559	2.47043	0.001990112	94	7.7	
T2D	PGS000032	Q1	Ref	Ref	Ref	NA	65	5.31	0.01003
T2D	PGS000032	Q2	0.9923901	0.6922387	1.423067	0.9667868	66	5.42	
T2D	PGS000032	Q3	1.109882	0.7817987	1.578912	0.5602002	75	6.16	
T2D	PGS000032	Q4	1.492685	1.070787	2.092444	0.01887703	94	7.7	
T2D	PGS000033	Q1	Ref	Ref	Ref	NA	69	5.66	0.04746
T2D	PGS000033	Q2	0.9571865	0.6745058	1.358678	0.8061399	70	5.51	
T2D	PGS000033	Q3	1.051495	0.7400083	1.494373	0.7790139	70	5.91	
T2D	PGS000033	Q4	1.391529	1.000137	1.944036	0.05093867	91	7.56	
T2D	PGS000854	Q1	Ref	Ref	Ref	NA	59	4.8	0.001591
T2D	PGS000854	Q2	1.251187	0.8698876	1.805197	0.2278952	69	5.68	
T2D	PGS000854	Q3	1.282195	0.8996338	1.835958	0.1710757	77	6.3	
T2D	PGS000854	Q4	1.701268	1.209628	2.410471	0.002473081	95	7.83	
**T2D**	**PGS004106**	**Q1**	**Ref**	**Ref**	**Ref**	**NA**	**61**	**4.98**	**0.001055**
**T2D**	**PGS004106**	**Q2**	**1.159746**	**0.8050437**	**1.674672**	**0.4267156**	**67**	**5.51**	
**T2D**	**PGS004106**	**Q3**	**1.245972**	**0.8706735**	**1.789277**	**0.2304171**	**72**	**5.89**	
**T2D**	**PGS004106**	**Q4**	**1.745824**	**1.24708**	**2.463607**	**0.00130832**	**100**	**8.22**	
T2D	PGS004225	Q1	Ref	Ref	Ref	NA	59	4.84	0.008584
T2D	PGS004225	Q2	1.243377	0.8715939	1.781434	0.231326	76	6.23	
T2D	PGS004225	Q3	1.180233	0.8233366	1.697724	0.3683903	72	5.89	
T2D	PGS004225	Q4	1.569727	1.114689	2.226249	0.01046826	93	7.65	
**CVD**	**PGS000059**	**Q1**	**Ref**	**Ref**	**Ref**	**NA**	**19**	**1.56**	**0.02205**
**CVD**	**PGS000059**	**Q2**	**0.8901336**	**0.4437484**	**1.765**	**0.7389642**	**16**	**1.28**	
**CVD**	**PGS000059**	**Q3**	**0.6010332**	**0.2721499**	**1.265422**	**0.1892544**	**11**	**0.922**	
**CVD**	**PGS000059**	**Q4**	**0.3980959**	**0.1613321**	**0.895484**	**0.03261855**	**8**	**0.656**	

Table S7 Distribution of cardiometabolic traits (TG, TC, HDL-C, LDL-C, FBS, and HbA1c) across PRS deciles

Trait	PRS	Decile	Median(Q1-Q3)	Min	Max
LDL-C	PGS000310	D1	87(69-106)	18	341.14
LDL-C	PGS000310	D2	100.2(82-119)	16	291.71
LDL-C	PGS000310	D3	108(88.45-126)	19	248.43
LDL-C	PGS000310	D4	107(88-125)	34.2	254.57
LDL-C	PGS000310	D5	111.235(90-130)	16	323
LDL-C	PGS000310	D6	113(94-135)	39	294
LDL-C	PGS000310	D7	112.9(93-132)	35.4	225
LDL-C	PGS000310	D8	112.5(93-134)	35	205
LDL-C	PGS000310	D9	115(96-134)	51	299
LDL-C	PGS000310	D10	120.855(100-140.0525)	36	301
HDL-C	PGS000309	D1	54(43.25-66)	25	115
HDL-C	PGS000309	D2	56(46-68)	24	106
HDL-C	PGS000309	D3	57(48.375-69)	27	123
HDL-C	PGS000309	D4	58(48-69.5)	21	123
HDL-C	PGS000309	D5	60(49-72)	25	131
HDL-C	PGS000309	D6	59(50-72)	27	126
HDL-C	PGS000309	D7	60(51-72)	21	128
HDL-C	PGS000309	D8	62(51-76)	22	140
HDL-C	PGS000309	D9	63(54-77)	32	167
HDL-C	PGS000309	D10	66(55.75-79)	32	143
TG	PGS000312	D1	70(51.25-100)	26	302
TG	PGS000312	D2	74(55-111)	24	668
TG	PGS000312	D3	75(56-103.5)	28	525
TG	PGS000312	D4	77(57-107)	25	662
TG	PGS000312	D5	79(58-112.75)	24	646
TG	PGS000312	D6	84(59-124)	21	402
TG	PGS000312	D7	85(63.5-122)	26	598
TG	PGS000312	D8	91(62-129.25)	31	635
TG	PGS000312	D9	87(64.25-128.75)	35	428
TG	PGS000312	D10	98(68-157.25)	29	1013
TC	PGS000311	D1	170(151-190)	103	347.5
TC	PGS000311	D2	183(163-204)	98	437.5
TC	PGS000311	D3	187(165-207)	114	283
TC	PGS000311	D4	188(166-210.5)	114	321.88
TC	PGS000311	D5	190.5(171-213.75)	107	318
TC	PGS000311	D6	190(166-210)	111	289
TC	PGS000311	D7	194.5(176-216.75)	68	325
TC	PGS000311	D8	195(175-217)	106	376
TC	PGS000311	D9	198(177-219)	96	427
TC	PGS000311	D10	203(184.25-224.875)	110	378
FBS	PGS000305	D1	85(81-90)	56	319
FBS	PGS000305	D2	85(80-91)	61	217
FBS	PGS000305	D3	86(82-92)	62	223
FBS	PGS000305	D4	87(82-93)	61	372
FBS	PGS000305	D5	86.785(82-92)	66	348
FBS	PGS000305	D6	87(82-92)	67	288
FBS	PGS000305	D7	88(83-93)	67	270
FBS	PGS000305	D8	88(82.75-93)	55	274
FBS	PGS000305	D9	89(84-95)	68	295
FBS	PGS000305	D10	90(83.625-96)	63	305
HbA1c	PGS000131	D1	5(5-6)	4	11
HbA1c	PGS000131	D2	5(5-6)	4	14.1
HbA1c	PGS000131	D3	5.3(5-6)	4	9
HbA1c	PGS000131	D4	5.2(5-6)	4	11.05
HbA1c	PGS000131	D5	5.5(5-6)	4.2	11
HbA1c	PGS000131	D6	5.5(5-6)	3.8	14
HbA1c	PGS000131	D7	5.7(5-6)	3.2	14
HbA1c	PGS000131	D8	5.6(5-6)	3.9	15
HbA1c	PGS000131	D9	5.675(5-6)	4	13
HbA1c	PGS000131	D10	6(5-6)	4	12

Table S8 Cox propotional hazard models of T2D PRSs

Trait	PRS	Quartile	HR	2.50%	97.50%	p-value
T2D	PGS000031	Q1	Ref	Ref	Ref	NA
T2D	PGS000031	Q2	1.220	0.774	1.923	3.91E-01
T2D	PGS000031	Q3	1.589	1.035	2.439	3.41E-02
T2D	PGS000031	Q4	1.440	0.927	2.235	1.05E-01
T2D	PGS000032	Q1	Ref	Ref	Ref	NA
T2D	PGS000032	Q2	1.241	0.809	1.905	3.23E-01
T2D	PGS000032	Q3	0.947	0.600	1.494	8.14E-01
T2D	PGS000032	Q4	1.536	1.018	2.317	4.08E-02
T2D	PGS000854	Q1	Ref	Ref	Ref	NA
T2D	PGS000854	Q2	0.811	0.532	1.238	3.32E-01
T2D	PGS000854	Q3	0.920	0.615	1.378	6.87E-01
T2D	PGS000854	Q4	0.968	0.647	1.449	8.75E-01
**T2D**	**PGS004106**	**Q1**	**Ref**	**Ref**	**Ref**	**NA**
**T2D**	**PGS004106**	**Q2**	**2.274**	**1.384**	**3.705**	**1.15E-03**
**T2D**	**PGS004106**	**Q3**	**2.593**	**1.628**	**4.260**	**7.93E-05**
**T2D**	**PGS004106**	**Q4**	**1.944**	**1.160**	**3.190**	**1.12E-02**
T2D	PGS004225	Q1	Ref	Ref	Ref	NA
T2D	PGS004225	Q2	0.951	0.610	1.483	8.24E-01
T2D	PGS004225	Q3	1.270	0.838	1.925	2.61E-01
T2D	PGS004225	Q4	1.277	0.841	1.940	2.52E-01
**CVD**	**PGS000059**	**Q1**	**Ref**	**Ref**	**Ref**	**NA**
**CVD**	**PGS000059**	**Q2**	**0.582**	**0.210**	**1.614**	**2.98E-01**
**CVD**	**PGS000059**	**Q3**	**1.176**	**0.506**	**2.734**	**7.06E-01**
**CVD**	**PGS000059**	**Q4**	**0.407**	**0.127**	**1.303**	**1.30E-01**

Table S9 Longitudinal predicted value of cardiometabolic traits (TG, TC, HDL-C, LDL-C, FBS, and HbA1c) by PI

Trait	PRS	Decile	Year	Predicted	SD	2.50%	97.50%
TG	PGS000312	D1-D2	BL	85.7896059	2.0674626	81.7372051	89.8420067
TG	PGS000312	D1-D2	1	86.1266029	1.91550044	82.3720608	89.8811451
TG	PGS000312	D1-D2	2	86.4636	1.86088322	82.8161122	90.1110878
TG	PGS000312	D1-D2	3	86.800597	1.91197146	83.0529719	90.548222
TG	PGS000312	D1-D2	4	87.137594	2.06091908	83.0980191	91.1771689
TG	PGS000312	D1-D2	5	87.474591	2.28869923	82.9885478	91.9606342
TG	PGS000312	D1-D2	6	87.8115881	2.57447177	82.7654066	92.8577695
TG	PGS000312	D3-D4	BL	90.4032657	2.06421269	86.357235	94.4492963
TG	PGS000312	D3-D4	1	91.5848692	1.9124083	87.8363879	95.3333505
TG	PGS000312	D3-D4	2	92.7664728	1.85738127	89.1258491	96.4070965
TG	PGS000312	D3-D4	3	93.9480763	1.90752535	90.2091661	97.6869866
TG	PGS000312	D3-D4	4	95.1296799	2.05515665	91.1013998	99.15796
TG	PGS000312	D3-D4	5	96.3112835	2.28142782	91.8394928	100.783074
TG	PGS000312	D3-D4	6	97.492887	2.56561593	92.4640638	102.52171
TG	PGS000312	D5-D6	BL	98.3986716	2.0491839	94.3820987	102.415245
TG	PGS000312	D5-D6	1	99.0743095	1.89729047	95.3554605	102.793159
TG	PGS000312	D5-D6	2	99.7499475	1.84004546	96.1433034	103.356591
TG	PGS000312	D5-D6	3	100.425585	1.88608672	96.7286966	104.122474
TG	PGS000312	D5-D6	4	101.101223	2.0283931	97.125402	105.077045
TG	PGS000312	D5-D6	5	101.776861	2.24876271	97.3690969	106.184625
TG	PGS000312	D5-D6	6	102.452499	2.52685348	97.4996535	107.405345
TG	PGS000312	D7-D8	BL	104.430648	2.02051045	100.470277	108.391018
TG	PGS000312	D7-D8	1	105.961204	1.87105911	102.293771	109.628638
TG	PGS000312	D7-D8	2	107.491761	1.8159371	103.932371	111.05115
TG	PGS000312	D7-D8	3	109.022317	1.86353391	105.369634	112.675
TG	PGS000312	D7-D8	4	110.552873	2.00655312	106.61986	114.485886
TG	PGS000312	D7-D8	5	112.08343	2.22668317	107.718943	116.447916
TG	PGS000312	D7-D8	6	113.613986	2.50366653	108.706589	118.521383
TG	PGS000312	D9-D10	BL	119.199603	2.02929709	115.22201	123.177196
TG	PGS000312	D9-D10	1	121.438255	1.87970728	117.753871	125.12264
TG	PGS000312	D9-D10	2	123.676908	1.82390701	120.101896	127.251919
TG	PGS000312	D9-D10	3	125.91556	1.87030977	122.249595	129.581525
TG	PGS000312	D9-D10	4	128.154212	2.01185612	124.210805	132.09762
TG	PGS000312	D9-D10	5	130.392864	2.23050577	126.020885	134.764844
TG	PGS000312	D9-D10	6	132.631517	2.50615872	127.719235	137.543799
TC	PGS000311	D1-D2	BL	178.005706	1.10535837	175.83911	180.172301
TC	PGS000311	D1-D2	1	179.490038	1.02067947	177.489421	181.490656
TC	PGS000311	D1-D2	2	180.974371	0.9820187	179.049531	182.89921
TC	PGS000311	D1-D2	3	182.458703	0.99475604	180.508898	184.408509
TC	PGS000311	D1-D2	4	183.943036	1.05703506	181.871158	186.014914
TC	PGS000311	D1-D2	5	185.427368	1.16090971	183.151888	187.702849
TC	PGS000311	D1-D2	6	186.911701	1.2964198	184.370609	189.452793
TC	PGS000311	D3-D4	BL	187.100724	1.12309995	184.899353	189.302094
TC	PGS000311	D3-D4	1	188.759967	1.03634394	186.728646	190.791288
TC	PGS000311	D3-D4	2	190.41921	0.99449136	188.469923	192.368497
TC	PGS000311	D3-D4	3	192.078453	1.00317817	190.11214	194.044767
TC	PGS000311	D3-D4	4	193.737697	1.06116393	191.657726	195.817667
TC	PGS000311	D3-D4	5	195.39694	1.16108586	193.121114	197.672766
TC	PGS000311	D3-D4	6	197.056183	1.29325981	194.521285	199.591081

TC	PGS000311	D5-D6	BL		191.415276	1.13029468	189.199803	193.630749
TC	PGS000311	D5-D6		1	192.889955	1.04285613	190.84587	194.934041
TC	PGS000311	D5-D6		2	194.364635	1.00081047	192.402962	196.326308
TC	PGS000311	D5-D6		3	195.839315	1.00984364	193.859936	197.818693
TC	PGS000311	D5-D6		4	197.313994	1.06866114	195.219328	199.40866
TC	PGS000311	D5-D6		5	198.788674	1.1697773	196.495812	201.081536
TC	PGS000311	D5-D6		6	200.263353	1.30338449	197.70861	202.818097
TC	PGS000311	D7-D8	BL		196.292153	1.11446162	194.107714	198.476592
TC	PGS000311	D7-D8		1	197.439749	1.02821324	195.424365	199.455134
TC	PGS000311	D7-D8		2	198.587346	0.98791795	196.650943	200.523748
TC	PGS000311	D7-D8		3	199.734942	0.99915113	197.776522	201.693362
TC	PGS000311	D7-D8		4	200.882538	1.06027625	198.804308	202.960769
TC	PGS000311	D7-D8		5	202.030135	1.16345612	199.749663	204.310607
TC	PGS000311	D7-D8		6	203.177731	1.2987056	200.632159	205.723303
TC	PGS000311	D9-D10	BL		202.124672	1.11803021	199.933239	204.316106
TC	PGS000311	D9-D10		1	203.235241	1.03216786	201.212105	205.258377
TC	PGS000311	D9-D10		2	204.345809	0.99104863	202.40327	206.288348
TC	PGS000311	D9-D10		3	205.456378	1.00020611	203.495889	207.416866
TC	PGS000311	D9-D10		4	206.566946	1.058336	204.492518	208.641374
TC	PGS000311	D9-D10		5	207.677514	1.15808717	205.407566	209.947463
TC	PGS000311	D9-D10		6	208.788083	1.28983901	206.25989	211.316276
HDL-C	PGS000309	D1-D2	BL		56.7523824	0.5234649	55.7263471	57.7784177
HDL-C	PGS000309	D1-D2		1	56.7834242	0.50426302	55.7950261	57.7718222
HDL-C	PGS000309	D1-D2		2	56.8144659	0.49567796	55.8428953	57.7860365
HDL-C	PGS000309	D1-D2		3	56.8455077	0.49825883	55.8688784	57.822137
HDL-C	PGS000309	D1-D2		4	56.8765495	0.51183674	55.8733063	57.8797926
HDL-C	PGS000309	D1-D2		5	56.9075912	0.53557596	55.8578172	57.9573652
HDL-C	PGS000309	D1-D2		6	56.938633	0.5682043	55.8249047	58.0523613
HDL-C	PGS000309	D3-D4	BL		59.1283297	0.5221288	58.1049133	60.1517462
HDL-C	PGS000309	D3-D4		1	59.120139	0.50277091	58.1346657	60.1056124
HDL-C	PGS000309	D3-D4		2	59.1119484	0.4939821	58.1437018	60.0801949
HDL-C	PGS000309	D3-D4		3	59.1037577	0.49632414	58.1309206	60.0765948
HDL-C	PGS000309	D3-D4		4	59.095567	0.5096436	58.0966226	60.0945114
HDL-C	PGS000309	D3-D4		5	59.0873764	0.53311834	58.0424195	60.1323332
HDL-C	PGS000309	D3-D4		6	59.0791857	0.56548505	57.9707873	60.187584
HDL-C	PGS000309	D5-D6	BL		61.1343537	0.52836185	60.09872	62.1699875
HDL-C	PGS000309	D5-D6		1	61.1983563	0.50880922	60.2010473	62.1956653
HDL-C	PGS000309	D5-D6		2	61.2623589	0.49985127	60.2826083	62.2421095
HDL-C	PGS000309	D5-D6		3	61.3263614	0.50205541	60.3422905	62.3104324
HDL-C	PGS000309	D5-D6		4	61.390364	0.51527844	60.3803749	62.4003532
HDL-C	PGS000309	D5-D6		5	61.4543666	0.53870955	60.3984505	62.5102827
HDL-C	PGS000309	D5-D6		6	61.5183692	0.57109367	60.3989775	62.6377609
HDL-C	PGS000309	D7-D8	BL		62.5827921	0.53237729	61.5392877	63.6262965
HDL-C	PGS000309	D7-D8		1	62.5316649	0.51269556	61.5267383	63.5365914
HDL-C	PGS000309	D7-D8		2	62.4805376	0.50388846	61.4928737	63.4682015
HDL-C	PGS000309	D7-D8		3	62.4294104	0.50652356	61.4365815	63.4222392
HDL-C	PGS000309	D7-D8		4	62.3782831	0.52042708	61.3582022	63.3983641
HDL-C	PGS000309	D7-D8		5	62.3271559	0.54473688	61.2594257	63.3948861
HDL-C	PGS000309	D7-D8		6	62.2760286	0.57814174	61.1428221	63.4092352
HDL-C	PGS000309	D9-D10	BL		66.8843649	0.5360892	65.8335849	67.9351449
HDL-C	PGS000309	D9-D10		1	66.7724587	0.51635463	65.7603601	67.7845573

HDL-C	PGS000309	D9-D10		2	66.6605524	0.50744423	65.665919	67.6551859
HDL-C	PGS000309	D9-D10		3	66.5486462	0.50992573	65.5491488	67.5481436
HDL-C	PGS000309	D9-D10		4	66.4367399	0.52363721	65.4103668	67.463113
HDL-C	PGS000309	D9-D10		5	66.3248337	0.54773596	65.251225	67.3984423
HDL-C	PGS000309	D9-D10		6	66.2129274	0.58093071	65.0742542	67.3516006
LDL-C	PGS000310	D1-D2	BL		94.7212371	1.00582804	92.749729	96.6927452
LDL-C	PGS000310	D1-D2		1	96.241189	0.92154331	94.4348862	98.0474919
LDL-C	PGS000310	D1-D2		2	97.7611409	0.87774743	96.0406817	99.4816002
LDL-C	PGS000310	D1-D2		3	99.2810929	0.88050298	97.5552325	101.006953
LDL-C	PGS000310	D1-D2		4	100.801045	0.929396	98.97935	102.62274
LDL-C	PGS000310	D1-D2		5	102.320997	1.017799	100.326025	104.315969
LDL-C	PGS000310	D1-D2		6	103.840949	1.13652943	101.613255	106.068642
LDL-C	PGS000310	D3-D4	BL		108.014494	1.02852278	105.998502	110.030485
LDL-C	PGS000310	D3-D4		1	109.298779	0.94212091	107.452142	111.145415
LDL-C	PGS000310	D3-D4		2	110.583064	0.89776985	108.823359	112.342769
LDL-C	PGS000310	D3-D4		3	111.867349	0.90169604	110.099948	113.634749
LDL-C	PGS000310	D3-D4		4	113.151634	0.95330319	111.283079	115.020189
LDL-C	PGS000310	D3-D4		5	114.435919	1.04555457	112.386544	116.485294
LDL-C	PGS000310	D3-D4		6	115.720204	1.16886609	113.429128	118.01128
LDL-C	PGS000310	D5-D6	BL		113.36302	1.00630756	111.390572	115.335468
LDL-C	PGS000310	D5-D6		1	114.390212	0.92152607	112.583943	116.196481
LDL-C	PGS000310	D5-D6		2	115.417404	0.87714464	113.698126	117.136682
LDL-C	PGS000310	D5-D6		3	116.444596	0.8793021	114.72109	118.168103
LDL-C	PGS000310	D5-D6		4	117.471788	0.92767379	115.653469	119.290108
LDL-C	PGS000310	D5-D6		5	118.498981	1.01567818	116.508165	120.489796
LDL-C	PGS000310	D5-D6		6	119.526173	1.1341264	117.303189	121.749156
LDL-C	PGS000310	D7-D8	BL		114.326761	1.03340589	112.301198	116.352324
LDL-C	PGS000310	D7-D8		1	115.326934	0.94639201	113.471926	117.181943
LDL-C	PGS000310	D7-D8		2	116.327108	0.90130591	114.560472	118.093744
LDL-C	PGS000310	D7-D8		3	117.327281	0.90443975	115.554503	119.10006
LDL-C	PGS000310	D7-D8		4	118.327454	0.9553191	116.454948	120.199961
LDL-C	PGS000310	D7-D8		5	119.327628	1.04700635	117.275407	121.379849
LDL-C	PGS000310	D7-D8		6	120.327801	1.16994633	118.034607	122.620995
LDL-C	PGS000310	D9-D10	BL		119.833254	1.02284954	117.828382	121.838126
LDL-C	PGS000310	D9-D10		1	120.53811	0.93734995	118.700824	122.375395
LDL-C	PGS000310	D9-D10		2	121.242965	0.89207948	119.494414	122.991516
LDL-C	PGS000310	D9-D10		3	121.947821	0.89317622	120.19712	123.698522
LDL-C	PGS000310	D9-D10		4	122.652676	0.94047799	120.80926	124.496093
LDL-C	PGS000310	D9-D10		5	123.357532	1.02762398	121.343302	125.371762
LDL-C	PGS000310	D9-D10		6	124.062387	1.14555679	121.816999	126.307776
FBS	PGS000305	D1-D2	BL		88.4869024	0.6751725	87.1635062	89.8102987
FBS	PGS000305	D1-D2		1	88.9553364	0.62902814	87.7223871	90.1882857
FBS	PGS000305	D1-D2		2	89.4237704	0.64303001	88.1633763	90.6841646
FBS	PGS000305	D1-D2		3	89.8922044	0.71364661	88.4933957	91.2910132
FBS	PGS000305	D1-D2		4	90.3606384	0.82649142	88.7406441	91.9806327
FBS	PGS000305	D1-D2		5	90.8290724	0.96689055	88.9338837	92.7242611
FBS	PGS000305	D1-D2		6	91.2975064	1.12457072	89.093251	93.5017618
FBS	PGS000305	D3-D4	BL		90.9610554	0.66876255	89.6502232	92.2718875
FBS	PGS000305	D3-D4		1	91.1094643	0.62178164	89.8907188	92.3282098
FBS	PGS000305	D3-D4		2	91.2578732	0.63697909	90.0093394	92.5064071
FBS	PGS000305	D3-D4		3	91.4062822	0.7103754	90.0138852	92.7986791

FBS	PGS000305	D3-D4		4	91.5546911	0.82661052	89.9344633	93.1749188
FBS	PGS000305	D3-D4		5	91.7031	0.97041104	89.8010109	93.6051892
FBS	PGS000305	D3-D4		6	91.8515089	1.13131389	89.6340364	94.0689815
FBS	PGS000305	D5-D6	BL		91.3482423	0.66808396	90.0387403	92.6577444
FBS	PGS000305	D5-D6		1	91.6729679	0.62256934	90.4526784	92.8932573
FBS	PGS000305	D5-D6		2	91.9976934	0.63734137	90.7484495	93.2469373
FBS	PGS000305	D5-D6		3	92.3224189	0.7086399	90.9334237	93.7114141
FBS	PGS000305	D5-D6		4	92.6471444	0.82188322	91.0361826	94.2581063
FBS	PGS000305	D5-D6		5	92.9718699	0.96237653	91.0855291	94.8582108
FBS	PGS000305	D5-D6		6	93.2965955	1.1199108	91.1014739	95.491717
FBS	PGS000305	D7-D8	BL		90.413178	0.67695258	89.0862927	91.7400634
FBS	PGS000305	D7-D8		1	91.0913524	0.6297596	89.8569694	92.3257354
FBS	PGS000305	D7-D8		2	91.7695268	0.64452524	90.5062019	93.0328517
FBS	PGS000305	D7-D8		3	92.4477012	0.71743409	91.0414686	93.8539337
FBS	PGS000305	D7-D8		4	93.1258755	0.83336298	91.4924124	94.7593387
FBS	PGS000305	D7-D8		5	93.8040499	0.97711786	91.8888148	95.719285
FBS	PGS000305	D7-D8		6	94.4822243	1.13820386	92.2512468	96.7132018
FBS	PGS000305	D9-D10	BL		92.6120758	1.12313363	90.4106373	94.8135144
FBS	PGS000305	D9-D10		1	92.6229166	0.96568947	90.7300821	94.5157511
FBS	PGS000305	D9-D10		2	92.6337574	0.82555442	91.0155997	94.2519151
FBS	PGS000305	D9-D10		3	92.6445981	0.71300838	91.2470404	94.0421559
FBS	PGS000305	D9-D10		4	92.6554389	0.642712	91.3956681	93.9152098
FBS	PGS000305	D9-D10		5	92.6662797	0.62899397	91.4333974	93.899162
FBS	PGS000305	D9-D10		6	92.6771205	0.67531105	91.3534527	94.0007882
HbA1c	PGS000131	D1-D2	BL		5.72775593	0.06068468	5.60877995	5.84673191
HbA1c	PGS000131	D1-D2		1	5.74680159	0.05696844	5.63511151	5.85849166
HbA1c	PGS000131	D1-D2		2	5.76584724	0.05500024	5.65801595	5.87367853
HbA1c	PGS000131	D1-D2		3	5.7848929	0.05496816	5.6771245	5.89266129
HbA1c	PGS000131	D1-D2		4	5.80393855	0.05687548	5.69243074	5.91544636
HbA1c	PGS000131	D1-D2		5	5.8229842	0.06053917	5.7042935	5.94167491
HbA1c	PGS000131	D1-D2		6	5.84202986	0.06566591	5.71328787	5.97077184
HbA1c	PGS000131	D3-D4	BL		5.70912394	0.06077624	5.58996844	5.82827943
HbA1c	PGS000131	D3-D4		1	5.73412823	0.0570573	5.62226396	5.8459925
HbA1c	PGS000131	D3-D4		2	5.75913253	0.05515436	5.65099908	5.86726597
HbA1c	PGS000131	D3-D4		3	5.78413682	0.05525538	5.67580532	5.89246832
HbA1c	PGS000131	D3-D4		4	5.80914111	0.05734976	5.69670345	5.92157878
HbA1c	PGS000131	D3-D4		5	5.83414541	0.06123331	5.7140938	5.95419701
HbA1c	PGS000131	D3-D4		6	5.8591497	0.06659375	5.72858864	5.98971076
HbA1c	PGS000131	D5-D6	BL		5.87159623	0.06010258	5.75376149	5.98943096
HbA1c	PGS000131	D5-D6		1	5.880226	0.05644969	5.76955297	5.99089902
HbA1c	PGS000131	D5-D6		2	5.88885576	0.05451144	5.78198279	5.99572874
HbA1c	PGS000131	D5-D6		3	5.89748553	0.05447118	5.79069149	6.00427958
HbA1c	PGS000131	D5-D6		4	5.9061153	0.05633298	5.79567109	6.01655951
HbA1c	PGS000131	D5-D6		5	5.91474507	0.0599198	5.79726869	6.03222145
HbA1c	PGS000131	D5-D6		6	5.92337484	0.06494645	5.7960434	6.05070628
HbA1c	PGS000131	D7-D8	BL		5.88777868	0.06246184	5.76531847	6.01023889
HbA1c	PGS000131	D7-D8		1	5.89417152	0.05857722	5.77932735	6.00901568
HbA1c	PGS000131	D7-D8		2	5.90056436	0.05650567	5.78978158	6.01134713
HbA1c	PGS000131	D7-D8		3	5.90695719	0.05644717	5.79628911	6.01762528
HbA1c	PGS000131	D7-D8		4	5.91335003	0.05840777	5.79883808	6.02786199
HbA1c	PGS000131	D7-D8		5	5.91974287	0.06219681	5.79780227	6.04168347

HbA1c	PGS000131	D7-D8		6	5.92613571	0.06750712	5.79378393	6.05848749
HbA1c	PGS000131	D9-D10	BL		5.86217838	0.06247757	5.73968732	5.98466943
HbA1c	PGS000131	D9-D10		1	5.88103814	0.05867648	5.76599937	5.99607691
HbA1c	PGS000131	D9-D10		2	5.8998979	0.05672466	5.78868578	6.01111002
HbA1c	PGS000131	D9-D10		3	5.91875766	0.05681304	5.80737226	6.03014306
HbA1c	PGS000131	D9-D10		4	5.93761742	0.05893245	5.8220768	6.05315804
HbA1c	PGS000131	D9-D10		5	5.95647718	0.06287783	5.83320141	6.07975296
HbA1c	PGS000131	D9-D10		6	5.97533695	0.06833364	5.84136473	6.10930917

Table S10 Linear mixed model (LMM) summary for cardiometabolic traits (TG, TC, HDL-C, LDL-C, FBS, and HbA1c) by PRS quintil

Trait	PRS	Variable	Estimate	Std_Error	DF	t_value	p_value
TG	PGS000312	(Intercept)	81.7114186	4.38959042	4051.06747	18.6148161	2.82E-74
TG	PGS000312	D3-D4	4.61365976	2.92041309	3808.22202	1.57979697	0.114236419
TG	PGS000312	D5-D6	12.6090657	2.90951501	3801.77124	4.33373456	1.50E-05
TG	PGS000312	D7-D8	18.641042	2.89022345	3803.46586	6.44968886	1.26E-10
TG	PGS000312	D9-D10	33.4099972	2.89601455	3794.50185	11.5365433	2.75E-30
TG	PGS000312	FU_year	0.33699702	0.44665652	3581.56439	0.7544881	0.450605794
TG	PGS000312	age	0.95660663	0.1004038	3745.578	9.5275938	2.80E-21
TG	PGS000312	sex	-38.803789	1.96999547	3771.31245	-19.6974	2.72E-82
TG	PGS000312	PC1	-446.21958	85.4020362	3744.51751	-5.224929	1.84E-07
TG	PGS000312	PC2	75.9747082	64.2539861	3752.68435	1.18241237	0.237117028
TG	PGS000312	PC3	313.484913	103.796079	3734.62926	3.02019994	0.002543178
TG	PGS000312	PC4	-56.179232	69.3934749	3819.50638	-0.8095751	0.418234833
TG	PGS000312	PC5	-262.34997	141.442753	3722.18165	-1.8548138	0.063701848
TG	PGS000312	PC6	71.3184927	69.0952804	3735.14384	1.03217604	0.302056486
TG	PGS000312	PC7	-155.90088	108.932256	3715.99687	-1.4311728	0.152464789
TG	PGS000312	PC8	93.8595273	102.719729	3614.94651	0.91374391	0.360912364
TG	PGS000312	PC9	-29.197057	57.7290111	3599.82615	-0.5057606	0.613055715
TG	PGS000312	PC10	-117.26521	60.2789646	3694.24394	-1.9453754	0.051805575
TG	PGS000312	D3-D4:FU_year	0.84460654	0.63048827	3587.1773	1.33960705	0.18045799
TG	PGS000312	D5-D6:FU_year	0.33864089	0.62625699	3604.98619	0.54073789	0.588721664
TG	PGS000312	D7-D8:FU_year	1.19355932	0.62338611	3588.37636	1.91463895	0.055617999
TG	PGS000312	D9-D10:FU_year	1.90165523	0.62327615	3571.46221	3.05106367	0.002297077
TC	PGS000311	(Intercept)	164.3312	2.35350303	4177.79004	69.824087	0
TC	PGS000311	D3-D4	9.09501821	1.57388042	3831.71667	5.77872251	8.13E-09
TC	PGS000311	D5-D6	13.4095702	1.58074408	3842.60522	8.48307476	3.09E-17
TC	PGS000311	D7-D8	18.2864472	1.57067633	3854.91776	11.6424032	8.18E-31
TC	PGS000311	D9-D10	24.1189668	1.57692974	3852.35773	15.29489	2.59E-51
TC	PGS000311	FU_year	1.48433255	0.22650022	3464.45743	6.55333831	6.46E-11
TC	PGS000311	age	0.49197929	0.05389387	3858.00936	9.12866935	1.09E-19
TC	PGS000311	sex	-5.2125126	1.0574832	3879.70421	-4.9291683	8.60E-07
TC	PGS000311	PC1	-124.77393	45.8930286	3856.33885	-2.7187993	0.006581262
TC	PGS000311	PC2	-6.2010604	34.5207025	3873.76923	-0.1796331	0.857450021
TC	PGS000311	PC3	34.6302609	55.7499532	3846.24417	0.62117112	0.534523849
TC	PGS000311	PC4	-21.355584	37.1822729	3912.36698	-0.5743485	0.565764994
TC	PGS000311	PC5	-84.187503	75.9432248	3834.03953	-1.1085584	0.267690248
TC	PGS000311	PC6	-32.723209	37.0464457	3845.72699	-0.8833022	0.377128203
TC	PGS000311	PC7	4.82027183	58.5004659	3826.56232	0.08239715	0.93433521
TC	PGS000311	PC8	28.3305981	54.9941661	3696.53723	0.51515643	0.606474518
TC	PGS000311	PC9	22.6076331	30.9711853	3708.46233	0.72995699	0.465462583
TC	PGS000311	PC10	-24.655955	32.328599	3803.23004	-0.762667	0.445709331
TC	PGS000311	D3-D4:FU_year	0.1749106	0.32008681	3421.98386	0.54644739	0.584794069
TC	PGS000311	D5-D6:FU_year	-0.009653	0.32143605	3424.55479	-0.0300308	0.976044245
TC	PGS000311	D7-D8:FU_year	-0.3367362	0.32101706	3473.58364	-1.0489667	0.294266433
TC	PGS000311	D9-D10:FU_year	-0.3737642	0.31935926	3420.66254	-1.1703564	0.241939104
HDL-C	PGS000309	(Intercept)	50.5957557	1.1732565	3997.72396	43.1242066	0
HDL-C	PGS000309	D3-D4	2.37594732	0.73931715	3858.51602	3.21370514	0.00132113
HDL-C	PGS000309	D5-D6	4.38197133	0.74390535	3859.93447	5.89049576	4.18E-09
HDL-C	PGS000309	D7-D8	5.83040971	0.74625053	3863.64568	7.81293883	7.15E-15
HDL-C	PGS000309	D9-D10	10.1319825	0.74903442	3858.52853	13.5267249	9.12E-41
HDL-C	PGS000309	FU_year	0.03104177	0.07466514	3265.68853	0.41574642	0.677622886
HDL-C	PGS000309	age	-0.0995707	0.02726266	3858.74323	-3.6522719	0.000263381
HDL-C	PGS000309	sex	12.4468391	0.53468081	3868.2027	23.2790086	2.71E-112
HDL-C	PGS000309	PC1	142.238206	23.1815717	3855.60441	6.135831	9.33E-10
HDL-C	PGS000309	PC2	-11.705993	17.4487377	3862.47779	-0.6708791	0.502337678
HDL-C	PGS000309	PC3	-20.99433	28.2075202	3851.6682	-0.7442813	0.456751709
HDL-C	PGS000309	PC4	0.78670114	18.765448	3883.34726	0.04192285	0.966562352
HDL-C	PGS000309	PC5	-0.5286522	38.4366009	3844.3156	-0.0137539	0.989027054
HDL-C	PGS000309	PC6	-28.569501	18.7619227	3848.73071	-1.5227384	0.127906314

HDL-C	PGS000309	PC7	-1.0467482	29.6076594	3838.25628	-0.035354	0.97179933
HDL-C	PGS000309	PC8	-21.387731	27.9687752	3764.86921	-0.7647003	0.44449796
HDL-C	PGS000309	PC9	-3.1378608	15.7625802	3771.09768	-0.1990703	0.842218521
HDL-C	PGS000309	PC10	27.8079378	16.416674	3825.1049	1.69388378	0.090368782
HDL-C	PGS000309	D3-D4:FU_year	-0.0392324	0.10542058	3297.8446	-0.3721516	0.709803901
HDL-C	PGS000309	D5-D6:FU_year	0.03296081	0.10580914	3289.12274	0.31151193	0.755431216
HDL-C	PGS000309	D7-D8:FU_year	-0.082169	0.10668386	3304.10759	-0.7702103	0.44123021
HDL-C	PGS000309	D9-D10:FU_year	-0.142948	0.10675633	3286.84965	-1.3390121	0.180659324
LDL-C	PGS000310	(Intercept)	87.7291204	2.12127858	4218.77992	41.3567182	3.78611041072002e-314
LDL-C	PGS000310	D3-D4	13.2932564	1.43730264	3830.40013	9.24875251	3.68E-20
LDL-C	PGS000310	D5-D6	18.6417828	1.42112635	3820.86829	13.1176111	1.75E-38
LDL-C	PGS000310	D7-D8	19.605524	1.44412022	3842.53745	13.5761023	4.84E-41
LDL-C	PGS000310	D9-D10	25.1120168	1.43879971	3835.84229	17.4534487	1.05E-65
LDL-C	PGS000310	FU_year	1.51995191	0.20450729	3419.88931	7.43226266	1.34E-13
LDL-C	PGS000310	age	0.4036346	0.04819405	3852.93401	8.37519508	7.63E-17
LDL-C	PGS000310	sex	-9.6879349	0.94507278	3873.96509	-10.250994	2.39E-24
LDL-C	PGS000310	PC1	-171.87921	41.0621767	3847.11437	-4.1858281	2.90E-05
LDL-C	PGS000310	PC2	-5.8497409	30.8825967	3871.38422	-0.1894187	0.849774609
LDL-C	PGS000310	PC3	1.84465955	49.8459386	3835.11123	0.03700722	0.970481176
LDL-C	PGS000310	PC4	-2.2990795	33.3592919	3934.98784	-0.0689187	0.945057824
LDL-C	PGS000310	PC5	-23.81042	67.8736429	3821.6449	-0.3508051	0.725753904
LDL-C	PGS000310	PC6	-16.794242	33.1134414	3835.25486	-0.507173	0.612062619
LDL-C	PGS000310	PC7	34.6306449	52.257827	3812.94726	0.66268819	0.507570328
LDL-C	PGS000310	PC8	19.9470021	49.0238261	3647.46689	0.40688383	0.684117187
LDL-C	PGS000310	PC9	41.1046825	27.6517932	3682.66167	1.48651056	0.137229731
LDL-C	PGS000310	PC10	-32.267871	28.8935607	3786.77877	-1.1167842	0.264157476
LDL-C	PGS000310	D3-D4:FU_year	-0.2356668	0.29348172	3404.4598	-0.8030034	0.422028806
LDL-C	PGS000310	D5-D6:FU_year	-0.4927598	0.28915532	3404.01069	-1.7041353	0.088447067
LDL-C	PGS000310	D7-D8:FU_year	-0.5197786	0.29374067	3431.11433	-1.7695151	0.076896754
LDL-C	PGS000310	D9-D10:FU_year	-0.8150963	0.29016157	3361.61502	-2.809112	0.00499654
FBS	PGS000305	(Intercept)	77.8023436	1.47696812	4280.64329	52.6770638	0
FBS	PGS000305	D3-D4	2.47415293	0.95030632	4903.58798	2.603532	0.009254674
FBS	PGS000305	D5-D6	2.8613399	0.94907729	4881.93684	3.01486498	0.002584158
FBS	PGS000305	D7-D8	1.92627559	0.95530223	4878.45152	2.01640436	0.043812409
FBS	PGS000305	D9-D10	4.19021802	0.95546983	4879.88314	4.38550533	1.18E-05
FBS	PGS000305	FU_year	0.46843399	0.19747493	6629.38468	2.37211878	0.01771479
FBS	PGS000305	age	0.39919871	0.03401703	3941.32732	11.7352591	2.77E-31
FBS	PGS000305	sex	-4.6866133	0.67024142	4018.81023	-6.9924256	3.15E-12
FBS	PGS000305	PC1	-75.238842	28.9713572	3963.25616	-2.5970078	0.009438738
FBS	PGS000305	PC2	16.0875404	21.7179465	3918.26417	0.74074869	0.458890217
FBS	PGS000305	PC3	64.7973733	35.196679	3943.38833	1.84100816	0.065695438
FBS	PGS000305	PC4	-35.147427	23.6073026	4064.46598	-1.4888371	0.136607866
FBS	PGS000305	PC5	-38.795269	47.9644706	3928.03012	-0.8088335	0.418659934
FBS	PGS000305	PC6	34.7018778	23.4023346	3934.47567	1.48283829	0.138197472
FBS	PGS000305	PC7	15.7286446	36.9722986	3929.21734	0.42541701	0.670556008
FBS	PGS000305	PC8	79.9935893	35.1309144	3980.36892	2.27701415	0.022838217
FBS	PGS000305	PC9	-9.2792853	19.5167156	3784.19421	-0.4754532	0.634491411
FBS	PGS000305	PC10	-26.667111	20.470786	3920.7822	-1.3026911	0.192756743
FBS	PGS000305	D3-D4:FU_year	-0.3200251	0.28084018	6745.87909	-1.1395273	0.254523738
FBS	PGS000305	D5-D6:FU_year	-0.1437085	0.27868991	6653.1761	-0.5156572	0.606110964
FBS	PGS000305	D7-D8:FU_year	0.20974038	0.28148394	6720.02264	0.7451238	0.456222943
FBS	PGS000305	D9-D10:FU_year	-0.4792748	0.27914277	6658.70532	-1.7169521	0.086034424
HbA1c	PGS000131	(Intercept)	5.16580474	0.13483677	1419.02415	38.3115426	4.37E-221
HbA1c	PGS000131	D3-D4	-0.018632	0.08573375	1365.59754	-0.217324	0.827988355
HbA1c	PGS000131	D5-D6	0.14384029	0.08500835	1361.92882	1.69207258	0.09086088
HbA1c	PGS000131	D7-D8	0.16002275	0.08715159	1373.46099	1.83614255	0.066552623
HbA1c	PGS000131	D9-D10	0.13442244	0.08725242	1370.54676	1.54061568	0.123641188
HbA1c	PGS000131	FU_year	0.01904565	0.01041272	1210.88679	1.8290756	0.06763411
HbA1c	PGS000131	age	0.01755159	0.00290231	1335.0418	6.04745824	1.91E-09
HbA1c	PGS000131	sex	-0.1850658	0.05843314	1328.7986	-3.1671373	0.001574498

HbA1c	PGS000131	PC1	-5.2014399	2.51150505	1335.74489	-2.0710449	0.038546267
HbA1c	PGS000131	PC2	3.01734597	2.02964674	1366.94441	1.48663603	0.137341573
HbA1c	PGS000131	PC3	1.59370466	3.03603877	1341.26765	0.52492895	0.599719332
HbA1c	PGS000131	PC4	-1.447394	2.02540517	1353.72143	-0.7146195	0.474967398
HbA1c	PGS000131	PC5	1.71518817	4.03646426	1335.80846	0.42492341	0.670960942
HbA1c	PGS000131	PC6	1.11548611	2.30741186	1368.65895	0.48343606	0.628863474
HbA1c	PGS000131	PC7	2.06584607	3.20326543	1344.39855	0.64491879	0.519089845
HbA1c	PGS000131	PC8	2.37562151	2.18453581	1233.18361	1.08747199	0.277040768
HbA1c	PGS000131	PC9	-1.4727069	1.8650552	1360.58947	-0.7896318	0.429880418
HbA1c	PGS000131	PC10	-0.3167016	1.54745875	1338.79735	-0.2046592	0.837869518
HbA1c	PGS000131	D3-D4:FU_year	0.00595864	0.01485787	1219.3419	0.40104262	0.688459012
HbA1c	PGS000131	D5-D6:FU_year	-0.0104159	0.01462029	1188.94421	-0.7124268	0.476340327
HbA1c	PGS000131	D7-D8:FU_year	-0.0126528	0.01497612	1212.92028	-0.8448658	0.398352375
HbA1c	PGS000131	D9-D10:FU_year	-0.0001859	0.01503466	1210.81222	-0.0123643	0.990137033

Table S11 Significant SNPs associated T2D, CVD, and cardiometabolic traits (TG, TC, HDL-C, LDL-C, FBS, and HbA1c) with *p* -value < 1 x 10^{-5}

Trait	PGS ID	SNP ID	Cytoband	Gene	Genic region	Alleles	Original PRS effect wei	MAF	Beta/OR	0.025	0.975	p-value	FDR
CVD	PGS000059	exm-rs9970807	1p32.2	PLPP3	intronic	A/G	0.12	0.03618	1.176	0.4596	3.008	0.7354	0.87469215
CVD	PGS000059	rs6544713	2p21	ABCG8	intronic	A/G	0.06	0.02666	1.008	0.3096	3.28	0.9898	0.99699444
CVD	PGS000059	rs2123536	2p24.1	OSR1;LINC00954	intergenic	A/G	0.11	0.2937	0.9514	0.6223	1.454	0.8179	0.905975
CVD	PGS000059	rs273909	5q31.1	MIR3936HG	ncRNA_intro	G/A	0.08	0.0644	1.258	0.6327	2.5	0.5131	0.76850849
CVD	PGS000059	rs10947789	6p21.2	KCNK5	intronic	G/A	0.06	0.1982	0.8005	0.476	1.346	0.4014	0.70056676
CVD	PGS000059	rs6903956	6p24.1	ADTRP	intronic	A/G	0.41	0.06705	0.5994	0.2439	1.473	0.2647	0.58523797
CVD	PGS000059	rs9349379	6p24.1	PHACTR1	UTR3	A/G	0.13	0.3768	1.244	0.8403	1.843	0.2752	0.59322914
CVD	PGS000059	rs2048327	6q25.3	SLC22A3	intronic	G/A	0.06	0.4543	1.078	0.733	1.587	0.7015	0.86328261
CVD	PGS000059	rs2107595	7p21.1	HDAC9;TWIST1	intergenic	A/G	0.08	0.3122	1.021	0.673	1.548	0.9229	0.97377
CVD	PGS000059	rs11556924	7q32.2	ZC3HC1	exonic	A/G	0.08	0.07092	1.433	0.7544	2.721	0.2719	0.59322914
CVD	PGS000059	rs264	8p21.3	LPL	intronic	A/G	0.07	0.2133	0.809	0.4885	1.34	0.4102	0.70426364
CVD	PGS000059	rs10757278	9p21.3	CDKN2B-AS1;DMRTA1	intergenic	A/G	0.25	0.3971	1.336	0.9065	1.968	0.1433	0.42597397
CVD	PGS000059	rs579459	9q34.2	ABO;SURF6	intergenic	G/A	0.1	0.1515	1.075	0.6363	1.817	0.7865	0.89314024
CVD	PGS000059	rs501120	10q11.21	LINC00841;LINC02881	intergenic	G/A	0.29	0.2956	1.49	1.01	2.199	0.04443	0.20144833
CVD	PGS000059	rs1412444	10q23.31	LIPA	intronic	A/G	0.09	0.3754	1.065	0.7128	1.592	0.7575	0.88557038
CVD	PGS000059	rs12413409	10q24.32	CNNM2	intronic	A/G	0.11	0.2598	0.8483	0.5429	1.325	0.4699	0.74920438
CVD	PGS000059	rs7136259	12q21.33	ATP2B1	intronic	A/G	0.1	0.3185	0.5117	0.3165	0.8274	0.006284	0.05178334
CVD	PGS000059	rs9319428	13q12.3	FLT1	intronic	A/G	0.06	0.4504	0.9803	0.6605	1.455	0.9214	0.97377
CVD	PGS000059	rs9515203	13q34	COL4A2	intronic	G/A	0.08	0.09587	1.252	0.6885	2.278	0.461	0.74284901
CVD	PGS000059	rs56062135	15q22.33	SMAD3	intronic	T/C	0.07	0.05093	0.9864	0.4189	2.323	0.975	0.99463427
CVD	PGS000059	rs1994016	15q25.1	ADAMTS7	intronic	A/G	0.17	0.1516	0.9258	0.5339	1.605	0.7837	0.89314024
CVD	PGS000059	exm-rs17514846	15q26.1	FURIN	intronic	A/C	0.06	0.2031	0.7463	0.4421	1.26	0.2733	0.59322914
CVD	PGS000059	rs12936587	17p11.2	PEMT;SMCR2	intergenic	A/G	0.07	0.1413	1.947	1.21	3.134	0.006052	0.05149664
CVD	PGS000059	19:11188247-G-A	19p13.2	SMARCA4;LDLR-AS1	intergenic	A/G	0.13	0.0204	1.379	0.4199	4.526	0.5965	0.79640779
CVD	PGS000059	rs4420638	19q13.32	APOC1	downstream	G/A	0.1	0.1397	1.114	0.6565	1.889	0.69	0.85397338
FBS	PGS000305	rs10811661	9p21.3	CDKN2B-AS1;DMRTA1	intergenic	G/A	0.0233	0.3969	-1.149	-1.883	-0.4146	0.002176	0.02196374
FBS	PGS000305	rs11619319	13q12.2	PLUT	ncRNA_intro	G/A	0.0194	0.4238	1.409	0.6891	2.13	0.0001273	0.00197315
FBS	PGS000305	rs340874	1q32.3	PROX1-AS1	ncRNA_intro	G/A	0.013	0.4455	0.5597	-0.1649	1.284	0.1301	0.40331
FBS	PGS000305	rs6072275	20q12	PLCG1-AS1	ncRNA_intro	A/G	0.0156	0.008813	-2.686	-6.566	1.194	0.1749	0.47441625
FBS	PGS000305	rs780094	2p23.3	GCKR	intronic	A/G	0.029	0.3594	-0.6883	-1.427	0.0505	0.06792	0.26797527
FBS	PGS000305	rs560887	2q31.1	G6PC2	intronic	A/G	0.075	0.03711	-0.5534	-2.449	1.342	0.5672	0.77996392
FBS	PGS000305	rs11715915	3p21.31	AMT	exonic	T/C	0.0118	0.06497	0.7417	-0.7008	2.184	0.3136	0.62777835
FBS	PGS000305	rs11708067	3q21.1	ADCY5	intronic	G/A	0.027	0.02662	0.3364	-1.912	2.585	0.7693	0.89314024
FBS	PGS000305	rs4869272	5q15	LOC101929710	ncRNA_intro	G/A	0.0175	0.3594	-0.2647	-1.007	0.4781	0.485	0.75715827
FBS	PGS000305	rs9368222	6p22.3	CDKAL1	intronic	A/C	0.014	0.335	1.009	0.2566	1.762	0.008612	0.06229347
FBS	PGS000305	rs4607517	7p13	GCK;YKT6	intergenic	A/G	0.062	0.1301	0.953	-0.1247	2.031	0.08313	0.30818932
FBS	PGS000305	rs2191349	7p21.2	DGKB;AGMO	intergenic	C/A	0.03	0.2885	-0.7874	-1.58	0.005258	0.0516	0.22246093
FBS	PGS000305	rs7034200	9p24.2	GLIS3	intronic	A/C	0.018	0.4577	0.1058	-0.6031	0.8147	0.7699	0.89314024
FBS	PGS000305	rs10885122	10q25.2	HEAT2;GPAM	intergenic	A/C	0.022	0.07584	-0.3221	-1.657	1.012	0.6362	0.82667904
FBS	PGS000305	rs174550	11q12.2	FADS1	intronic	A/G	0.017	0.3076	0.5561	-0.227	1.339	0.1641	0.46447435
FBS	PGS000305	exm-rs576674	13q13.1	LINC00423;KL	intergenic	G/A	0.0172	0.2498	-0.2026	-1.027	0.6214	0.6299	0.82342349
FBS	PGS000305	rs11071657	15q22.2	C2CD4A;C2CD4B	intergenic	G/A	0.008	0.3768	-0.7015	-1.427	0.02457	0.05834	0.23886377
HbA1c	PGS000131	rs13387347	2q24.3	SPC25;G6PC2	intergenic	G/A	-0.007	0.4131	0.05907	0.02817	0.08998	0.0001815	0.00256862
HbA1c	PGS000131	rs7756992	6p22.3	CDKAL1	intronic	G/A	0.007	0.4216	0.05007	0.01963	0.08051	0.001273	0.01479863
HbA1c	PGS000131	rs2383208	9p21.3	CDKN2B-AS1;DMRTA1	intergenic	G/A	0.005	0.3909	-0.04874	-0.07967	-0.01782	0.002019	0.020863

HbA1c	PGS000131	rs11619319	13q12.2	PLUT	ncRNA_intro	G/A	0.016	0.4238	0.0561	0.02562	0.08658	0.0003124	0.00415046
HbA1c	PGS000131	rs560887	2q31.1	G6PC2	intronic	A/G	0.04	0.03711	-0.04297	-0.1229	0.03694	0.292	0.60947788
HbA1c	PGS000131	rs11708067	3q21.1	ADCY5	intronic	G/A	0.012	0.02662	0.004483	-0.09107	0.1	0.9267	0.97377
HbA1c	PGS000131	rs8192675	3q26.2	SLC2A2	intronic	G/A	0.029	0.2364	-0.03	-0.06557	0.005564	0.09833	0.33883
HbA1c	PGS000131	rs4607517	7p13	GCK;YKT6	intergenic	A/G	0.046	0.1301	0.05971	0.01427	0.1051	0.01003	0.06914305
HbA1c	PGS000131	7:44247258-C-T	7p13	YKT6	intronic	A/G	0.017	0.4558	-0.008895	-0.03917	0.02138	0.5648	0.77996392
HbA1c	PGS000131	rs2191349	7p21.2	DGKB;AGMO	intergenic	C/A	0.009	0.2885	-0.02704	-0.06052	0.006432	0.1134	0.37097186
HbA1c	PGS000131	rs11558471	8q24.11	SLC30A8	UTR3	G/A	0.019	0.4423	-0.03104	-0.0607	-0.001383	0.04028	0.1914035
HbA1c	PGS000131	rs579459	9q34.2	ABO;SURF6	intergenic	G/A	0.027	0.1515	-0.008785	-0.05082	0.03325	0.6821	0.85226336
HbA1c	PGS000131	GSA-rs17747324	10q25.2	TCF7L2	intronic	G/A	-0.004	0.03571	-0.02174	-0.1023	0.05881	0.5969	0.79640779
HbA1c	PGS000131	rs2237896	11p15.4	KCNQ1	intronic	A/G	-0.009	0.3042	-0.02408	-0.05687	0.008713	0.1502	0.43847623
HbA1c	PGS000131	rs174577	11q12.2	FADS2	intronic	C/A	0.018	0.3058	0.01027	-0.02278	0.04332	0.5425	0.77813974
HbA1c	PGS000131	exm-rs576674	13q13.1	LINC00423;KL	intergenic	G/A	0.022	0.2498	-0.004203	-0.03887	0.03046	0.8122	0.90505692
HDL-C	PGS000309	rs442177	4q22.1	AFF1	intronic	C/A	-0.018	0.4135	0.9295	0.2817	1.577	0.004944	0.04349384
HDL-C	PGS000309	rs643381	6q24.1	LINC01625;FILNC1	intergenic	A/C	0.023	0.3598	1.234	0.5688	1.9	0.0002811	0.00381242
HDL-C	PGS000309	rs4917014	7p12.2	SPATA48;IKZF1	intergenic	C/A	0.017	0.2051	1.273	0.4588	2.087	0.002193	0.02196374
HDL-C	PGS000309	rs972283	7q32.2	KLF14;MIR29A	intergenic	A/G	-0.031	0.3289	0.9884	0.3056	1.671	0.004569	0.04074547
HDL-C	PGS000309	rs301	8p21.3	LPL	intronic	G/A	0.11	0.223	1.415	0.6424	2.188	0.0003357	0.00428511
HDL-C	PGS000309	rs326	8p21.3	LPL	intronic	G/A	0.11	0.225	1.459	0.6898	2.228	0.0002034	0.00281731
HDL-C	PGS000309	rs13702	8p21.3	LPL	UTR3	G/A	0.11	0.2219	1.484	0.7117	2.257	0.0001686	0.00243908
HDL-C	PGS000309	rs10096633	8p21.3	LPL;SLC18A1	intergenic	A/G	0.14	0.1278	1.969	1.005	2.934	0.0000637	0.00111013
HDL-C	PGS000309	rs4149268	9q31.1	ABCA1	intronic	A/G	-0.034	0.3628	-1.817	-2.487	-1.147	1.108E-07	4.5163E-06
HDL-C	PGS000309	rs1883025	9q31.1	ABCA1	intronic	A/G	-0.067	0.2555	-2.49	-3.221	-1.759	2.701E-11	1.5979E-09
HDL-C	PGS000309	rs2075291	11q23.3	APOA5	exonic	A/C	-0.29	0.03743	-3.814	-5.505	-2.123	0.00001003	0.00023948
HDL-C	PGS000309	rs10468017	15q21.3	AQP9;LIPC	intergenic	A/G	0.11	0.1941	1.331	0.5152	2.147	0.001394	0.01592095
HDL-C	PGS000309	rs1532085	15q21.3	AQP9;LIPC	intergenic	A/G	-0.096	0.4751	1.656	1.014	2.298	4.425E-07	1.6022E-05
HDL-C	PGS000309	rs1800588	15q21.3	LIPC	upstream	A/G	0.12	0.4015	1.436	0.7845	2.087	0.00001583	0.00034286
HDL-C	PGS000309	rs173539	16q13	HERPUD1;CETP	intergenic	A/G	0.23	0.2252	2.662	1.898	3.427	9.75E-12	7.0525E-10
HDL-C	PGS000309	rs247616	16q13	HERPUD1;CETP	intergenic	A/G	0.24	0.1807	3.736	2.91	4.562	1.077E-18	1.4062E-16
HDL-C	PGS000309	rs3764261	16q13	HERPUD1;CETP	intergenic	A/C	0.24	0.1807	3.844	3.016	4.673	1.372E-19	2.2297E-17
HDL-C	PGS000309	rs9939224	16q13	CETP	intronic	A/C	0.2	0.1386	-2.31	-3.226	-1.394	7.905E-07	2.7102E-05
HDL-C	PGS000309	rs5882	16q13	CETP	exonic	G/A	-0.092	0.4235	1.482	0.8309	2.133	8.362E-06	0.00020932
HDL-C	PGS000309	rs2303790	16q13	CETP	exonic	G/A	0.37	0.01979	7.832	5.566	10.1	1.418E-11	9.2442E-10
HDL-C	PGS000309	rs7241918	18q21.1	LIPG;ACAA2	intergenic	C/A	0.077	0.1563	-1.288	-2.168	-0.4074	0.004162	0.03763142
HDL-C	PGS000309	rs7412	19q13.32	APOE	exonic	A/G	0.098	0.08845	3.223	2.083	4.364	3.208E-08	1.3931E-06
HDL-C	PGS000309	rs445925	19q13.32	APOE;APOC1	intergenic	A/G	0.04	0.1098	2.758	1.736	3.779	1.274E-07	4.8634E-06
HDL-C	PGS000309	rs7679	20q13.12	PCIF1	UTR3	G/A	-0.056	0.06387	-2.25	-3.55	-0.9497	0.0007001	0.00844009
HDL-C	PGS000309	exm194032	2p16.2	TSPYL6	exonic	A/G	0.04	0.02285	-0.1377	-2.261	1.985	0.8989	0.96247352
HDL-C	PGS000309	rs676210	2p24.1	APOB	exonic	G/A	0.06	0.3095	0.1443	-0.5505	0.8391	0.684	0.85226336
HDL-C	PGS000309	rs533617	2p24.1	APOB	exonic	G/A	0.085	0.003382	2.26	-3.281	7.802	0.4241	0.70974062
HDL-C	PGS000309	rs1367117	2p24.1	APOB	exonic	A/G	-0.02	0.1396	-0.06036	-0.9871	0.8664	0.8984	0.96247352
HDL-C	PGS000309	exm229906	2q21.3	LCT	exonic	A/G	-0.019	0.4789	0.4121	-0.2284	1.053	0.2073	0.50355336
HDL-C	PGS000309	rs13389219	2q24.3	COBLL1	intronic	A/G	0.035	0.109	-0.3142	-1.34	0.7111	0.5481	0.77813974
HDL-C	PGS000309	rs12328675	2q24.3	COBLL1	UTR3	G/A	0.05	0.0159	0.4464	-2.126	3.019	0.7338	0.87469215
HDL-C	PGS000309	rs7607980	2q24.3	COBLL1	exonic	G/A	0.05	0.01189	0.6061	-2.332	3.545	0.686	0.85226336
HDL-C	PGS000309	rs1047891	2q34	CPS1	exonic	A/C	-0.027	0.2099	-0.004712	-0.7933	0.7839	0.9907	0.99699444
HDL-C	PGS000309	rs2943641	2q36.3	LOC646736;MIR5702	intergenic	A/G	-0.036	0.1178	-0.02478	-1.011	0.9615	0.9607	0.99110379

HDL-C	PGS000309	rs9311651	3p14.3	DNAH12	exonic	G/A	0.019	0.009941	1.801	-1.424	5.026	0.2737	0.59322914
HDL-C	PGS000309	rs13326165	3p21.1	STAB1	intronic	A/G	-0.025	0.05456	1.683	0.2738	3.092	0.01928	0.10921086
HDL-C	PGS000309	3:47282303	3p21.31	KIF9	exonic	G/A	-0.015	0.4457	0.5563	-0.08767	1.2	0.0905	0.32371154
HDL-C	PGS000309	rs6762477	3p21.31	RBM6	intronic	G/A	0.025	0.1654	0.2896	-0.5756	1.155	0.5118	0.76850849
HDL-C	PGS000309	rs2606736	3p25.3	ATG7	intronic	G/A	0.025	0.3207	-0.4613	-1.145	0.222	0.1858	0.49101741
HDL-C	PGS000309	rs6805251	3q13.33	GSK3B	intronic	G/A	0.02	0.3806	-0.5194	-1.185	0.1464	0.1263	0.39720435
HDL-C	PGS000309	rs17404153	3q22.1	DNAJC13	intronic	A/C	-0.028	0.141	0.02149	-0.903	0.946	0.9637	0.99110379
HDL-C	PGS000309	rs645040	3q22.3	MSL2;PCCB	intergenic	C/A	-0.021	0.1654	-0.6872	-1.55	0.1754	0.1185	0.38189851
HDL-C	PGS000309	rs10019888	4p15.2	SMIM20;RBPJ	intergenic	G/A	-0.027	0.008508	1.177	-2.208	4.561	0.4956	0.76251247
HDL-C	PGS000309	rs2602836	4q23	LOC100507053	ncRNA_intro	A/G	0.019	0.1181	-0.09022	-1.093	0.9128	0.8601	0.93165574
HDL-C	PGS000309	rs13107325	4q24	SLC39A8	exonic	A/G	-0.074	0.000415	1.976	-15.82	19.77	0.8277	0.90712576
HDL-C	PGS000309	rs459193	5q11.2	C5orf67	downstream	G/A	-0.02	0.4378	-0.8821	-1.53	-0.2342	0.007642	0.05852873
HDL-C	PGS000309	rs9686661	5q11.2	C5orf67	ncRNA_intro	A/G	-0.032	0.1961	-1.108	-1.911	-0.3061	0.006802	0.05400124
HDL-C	PGS000309	exm-rs4976033	5q13.1	PIK3R1;LINC02198	intergenic	G/A	-0.015	0.4934	-0.6376	-1.272	-0.003587	0.04878	0.21456608
HDL-C	PGS000309	rs6905288	6p21.1	VEGFA;LINC02537	intergenic	G/A	-0.024	0.2989	0.836	0.135	1.537	0.01946	0.10921086
HDL-C	PGS000309	rs998584	6p21.1	VEGFA;LINC02537	intergenic	C/A	-0.026	0.4311	0.5351	-0.1149	1.185	0.1067	0.35621385
HDL-C	PGS000309	rs35349911	6p21.1	VEGFA;LINC02537	intergenic	G/A	-0.017	0.4022	0.5624	-0.09492	1.22	0.09363	0.330365
HDL-C	PGS000309	rs2894342	6p21.31	MLN;LINC01016	intergenic	A/C	0.017	0.1726	0.3935	-0.4741	1.261	0.3741	0.67419862
HDL-C	PGS000309	rs2814982	6p21.31	SPDEF;ILRUN	intergenic	A/G	-0.028	0.04912	-0.7539	-2.234	0.7261	0.3181	0.62943191
HDL-C	PGS000309	rs11755393	6p21.31	UHRF1BP1	exonic	G/A	-0.027	0.4438	-0.1286	-0.774	0.5168	0.6962	0.86001176
HDL-C	PGS000309	rs9275596	6p21.32	HLA-DQB1;HLA-DQA2	intergenic	G/A	0.023	0.09854	-0.8081	-2.121	0.5045	0.2276	0.53879127
HDL-C	PGS000309	rs1150754	6p21.33	TNXB	intronic	A/G	-0.024	0.01948	-1.736	-3.993	0.5204	0.1316	0.40503255
HDL-C	PGS000309	exm521119	6p22.2	SLC17A2	exonic	A/G	-0.025	0.01383	-0.3955	-3.128	2.337	0.7767	0.89314024
HDL-C	PGS000309	rs9376090	6q23.3	HBS1L;MYB	intergenic	G/A	-0.016	0.1501	0.06393	-0.8427	0.9705	0.8901	0.95777702
HDL-C	PGS000309	rs1084651	6q26	LPA;PLG	intergenic	A/G	-0.031	0.452	-0.5395	-1.182	0.1026	0.09965	0.33964476
HDL-C	PGS000309	rs1997243	7p22.3	C7orf50	intronic	G/A	0.026	0.01128	2.604	-0.5766	5.784	0.1086	0.35805
HDL-C	PGS000309	rs11761941	7p22.3	GPR146	exonic	A/G	0.028	0.01301	2.607	-0.3424	5.556	0.08327	0.30818932
HDL-C	PGS000309	rs2240466	7q11.23	BAZ1B	UTR3	A/G	0.043	0.1069	-0.5599	-1.597	0.477	0.2899	0.60947788
HDL-C	PGS000309	rs1178979	7q11.23	BAZ1B	UTR3	G/A	0.035	0.1076	-0.6175	-1.652	0.4174	0.2423	0.5613427
HDL-C	PGS000309	rs4731702	7q32.2	KLF14;MIR29A	intergenic	A/G	0.033	0.3215	0.9442	0.2591	1.629	0.006936	0.05440164
HDL-C	PGS000309	exm670797	7q36.1	GIMAP7	exonic	A/G	-0.017	0.1316	0.2601	-0.6883	1.209	0.5909	0.79640779
HDL-C	PGS000309	rs17173637	7q36.1	TMEM176A;AOC1	intergenic	G/A	-0.036	0.04141	-0.08721	-1.674	1.499	0.9142	0.97182724
HDL-C	PGS000309	rs1801177	8p21.3	LPL	exonic	A/G	-0.2	0.0008198	-7.148	-17.99	3.693	0.1963	0.49531512
HDL-C	PGS000309	rs268	8p21.3	LPL	exonic	G/A	-0.26	0.0007181	-5.745	-23.44	11.95	0.5245	0.7763175
HDL-C	PGS000309	rs4841132	8p23.1	LOC157273	ncRNA_exoni	A/G	0.1	0.03069	-2.073	-3.974	-0.1716	0.03267	0.16615758
HDL-C	PGS000309	rs2293889	8q23.3	TRPS1	intronic	A/C	0.029	0.1885	0.1839	-0.64	1.008	0.6618	0.84146836
HDL-C	PGS000309	rs2954033	8q24.13	TRIB1;LINC00861	intergenic	A/G	0.047	0.2775	-0.7102	-1.423	0.003016	0.05104	0.2215136
HDL-C	PGS000309	rs2954038	8q24.13	TRIB1;LINC00861	intergenic	C/A	0.048	0.2754	-0.6894	-1.406	0.02754	0.05954	0.24225338
HDL-C	PGS000309	rs10968576	9p21.1	LINGO2	intronic	G/A	-0.017	0.1126	-0.3316	-1.353	0.6899	0.5247	0.7763175
HDL-C	PGS000309	rs643531	9p22.3	TTC39B	intronic	C/A	0.053	0.0279	-2.497	-4.423	-0.5707	0.0111	0.07299091
HDL-C	PGS000309	rs3890182	9q31.1	ABCA1	intronic	A/G	-0.075	0.06848	-1.777	-3.05	-0.504	0.006247	0.05178334
HDL-C	PGS000309	rs2230808	9q31.1	ABCA1	exonic	A/G	0.027	0.4522	-0.5589	-1.193	0.07566	0.08437	0.30856669
HDL-C	PGS000309	rs145183203	9q31.1	ABCA1	exonic	A/G	-0.25	0.0001025	-13.85	-44.44	16.74	0.3749	0.67419862
HDL-C	PGS000309	rs687621	9q34.2	ABO	intronic	G/A	0.015	0.4054	-0.3476	-1	0.3049	0.2965	0.61471815
HDL-C	PGS000309	rs970548	10q11.22	MARCHF8	intronic	C/A	0.026	0.08171	-0.3594	-1.521	0.8016	0.544	0.77813974
HDL-C	PGS000309	rs2068888	10q23.33	CYP26A1;MYOF	intergenic	G/A	0.023	0.1926	-0.4778	-1.294	0.338	0.2511	0.57155979
HDL-C	PGS000309	kgp9182400	10q24.31	ERLIN1	exonic	G/A	0.016	0.07197	-0.3616	-1.587	0.8637	0.563	0.77996392

HDL-C	PGS000309	rs2255141	10q25.2	GPAM	intronic	A/G	-0.027	0.1791	0.5974	-0.2544	1.449	0.1693	0.47302275
HDL-C	PGS000309	exm-rs7076938	10q25.3	NHLRC2;ADRB1	intergenic	G/A	0.019	0.2787	-0.05961	-0.7771	0.6579	0.8706	0.93990149
HDL-C	PGS000309	rs740363	10q25.3	HSPA12A	intronic	A/G	0.014	0.1638	-0.6127	-1.478	0.2523	0.1651	0.46528182
HDL-C	PGS000309	rs3136441	11p11.2	F2	intronic	A/G	0.054	0.4147	0.1052	-0.5481	0.7585	0.7523	0.88557038
HDL-C	PGS000309	rs61731956	11p11.2	NR1H3	exonic	A/G	0.47	0.0001025	20.6	-10.47	51.68	0.1939	0.49531512
HDL-C	PGS000309	rs10838738	11p11.2	MTCH2	intronic	G/A	-0.032	0.2232	-0.4467	-1.224	0.33	0.2597	0.5789887
HDL-C	PGS000309	rs16928809	11p15.4	SLC22A18	intronic	A/G	-0.029	0.07128	0.06675	-1.161	1.294	0.9151	0.97182724
HDL-C	PGS000309	rs2923084	11p15.4	CAND1.11	ncRNA_intro	G/A	-0.026	0.4802	-0.2239	-0.8712	0.4234	0.4978	0.76251247
HDL-C	PGS000309	rs11246602	11q11	OR4C46;OR4A5	intergenic	G/A	0.034	0.138	0.08447	-0.8316	1.001	0.8566	0.9301705
HDL-C	PGS000309	rs174546	11q12.2	FADS1	UTR3	G/A	-0.042	0.3075	0.07943	-0.6255	0.7844	0.8252	0.90712576
HDL-C	PGS000309	rs174547	11q12.2	FADS1	intronic	A/G	-0.042	0.3076	0.08058	-0.6239	0.7851	0.8226	0.90712576
HDL-C	PGS000309	rs174550	11q12.2	FADS1	intronic	A/G	-0.042	0.3076	0.08058	-0.6239	0.7851	0.8226	0.90712576
HDL-C	PGS000309	rs12801636	11q13.1	PCNX3	intronic	A/G	0.024	0.3553	0.3564	-0.3196	1.032	0.3015	0.62112816
HDL-C	PGS000309	rs499974	11q13.5	MOGAT2;LOC283214	intergenic	A/C	-0.026	0.2979	-0.2747	-0.9852	0.4358	0.4487	0.73025925
HDL-C	PGS000309	rs746463	11q22.3	ZC3H12C	intronic	G/A	-0.017	0.149	0.2896	-0.6063	1.186	0.5264	0.77706667
HDL-C	PGS000309	rs76353203	11q23.3	APOC3	exonic	A/G	0.88	0.0001025	32.68	2.072	63.3	0.03644	0.1776647
HDL-C	PGS000309	exm2264455	11q23.3	SIK3	intronic	C/A	-0.018	0.2457	-0.933	-1.678	-0.1881	0.01413	0.08675944
HDL-C	PGS000309	rs7350481	11q23.3	LINC02702;BUD13	intergenic	A/G	0.098	0.247	-0.5876	-1.324	0.1487	0.1179	0.38185522
HDL-C	PGS000309	rs11820589	11q23.3	BUD13	exonic	A/G	-0.087	0.008633	-2.304	-5.827	1.218	0.1998	0.4991
HDL-C	PGS000309	rs10790162	11q23.3	BUD13	intronic	A/G	0.11	0.2204	-0.6454	-1.416	0.1255	0.1009	0.34211406
HDL-C	PGS000309	rs10047462	11q23.3	APOA1-AS	ncRNA_intro	C/A	0.023	0.4824	0.3983	-0.2395	1.036	0.221	0.53081538
HDL-C	PGS000309	rs7941030	11q24.1	MIR100HG;UBASH3B	intergenic	G/A	0.024	0.344	-0.1629	-0.8517	0.5258	0.6429	0.8304125
HDL-C	PGS000309	rs8177399	11q24.2	TIRAP	exonic	A/G	-0.05	0.0007175	-6.403	-18.93	6.119	0.3163	0.62777835
HDL-C	PGS000309	rs7134375	12p12.2	LINC02468;PDE3A	intergenic	A/C	0.021	0.2266	0.3704	-0.3955	1.136	0.3433	0.64667254
HDL-C	PGS000309	rs4883201	12p13.31	PHC1	intronic	G/A	-0.03	0.2937	0.01837	-0.687	0.7238	0.9593	0.99110379
HDL-C	PGS000309	rs11613352	12q13.3	R3HDM2	intronic	A/G	0.028	0.0708	0.6553	-0.597	1.908	0.3051	0.6233325
HDL-C	PGS000309	rs1106766	12q13.3	R3HDM2	intronic	A/G	0.032	0.06723	0.6889	-0.5927	1.97	0.2921	0.60947788
HDL-C	PGS000309	rs3184504	12q24.12	SH2B3	exonic	A/G	0.027	0.006251	-2.047	-6.093	1.998	0.3213	0.63192236
HDL-C	PGS000309	rs1183910	12q24.31	HNF1A	intronic	A/G	0.015	0.2865	0.2588	-0.4514	0.969	0.4751	0.75070413
HDL-C	PGS000309	rs7310409	12q24.31	HNF1A	intronic	A/G	-0.016	0.3142	-0.1555	-0.8478	0.5367	0.6597	0.84094932
HDL-C	PGS000309	rs4759375	12q24.31	SBNO1	intronic	A/G	0.051	0.3135	0.5802	-0.1182	1.279	0.1035	0.3491114
HDL-C	PGS000309	rs838880	12q24.31	SCARB1	UTR3	A/G	-0.029	0.4707	-0.4436	-1.082	0.195	0.1734	0.47441625
HDL-C	PGS000309	rs10483776	14q23.3	FUT8	intronic	G/A	-0.02	0.06621	0.1223	-1.172	1.417	0.8531	0.9287092
HDL-C	PGS000309	rs4983559	14q32.33	ZBTB42;LINC00638	intergenic	A/G	-0.027	0.1712	-0.4158	-1.263	0.4315	0.3362	0.64391559
HDL-C	PGS000309	rs113298164	15q21.3	LIPC	exonic	A/G	0.33	0.0001025	-20.17	-50.81	10.48	0.1971	0.49541351
HDL-C	PGS000309	rs7200543	16p13.11	PDXDC1	exonic	G/A	-0.019	0.3765	-0.2667	-0.9225	0.3891	0.4254	0.70977315
HDL-C	PGS000309	kgp3151253	16p13.13	RMI2;LOC400499	intergenic	A/C	0.044	0.04837	-1.591	-3.093	-0.08908	0.03793	0.18280654
HDL-C	PGS000309	rs1421085	16q12.2	FTO	intronic	G/A	-0.022	0.243	-0.2654	-1.025	0.4946	0.4938	0.76251247
HDL-C	PGS000309	rs9989419	16q13	HERPUD1;CETP	intergenic	A/G	0.13	0.2533	-0.4208	-1.16	0.3184	0.2646	0.58523797
HDL-C	PGS000309	rs8060686	16q22.1	EDC4	exonic	G/A	0.056	0.08824	1.537	0.397	2.676	0.008254	0.06176269
HDL-C	PGS000309	rs16942887	16q22.1	PSKH1	intronic	A/G	0.08	0.05076	2.048	0.5852	3.511	0.006091	0.05149664
HDL-C	PGS000309	rs2925979	16q23.2	CMIP	intronic	A/G	0.041	0.3874	0.1434	-0.5095	0.7964	0.6668	0.84415107
HDL-C	PGS000309	rs2292642	17q25.3	PGS1	exonic	G/A	0.028	0.4665	-0.2588	-0.8927	0.3751	0.4237	0.70974062
HDL-C	PGS000309	rs4129767	17q25.3	PGS1	intronic	A/G	0.026	0.4188	0.3217	-0.3237	0.967	0.3287	0.63825045
HDL-C	PGS000309	rs142545730	18q21.1	LIPG	exonic	A/G	0.38	0.00041	0.1296	-21.54	21.79	0.9906	0.99699444
HDL-C	PGS000309	rs77960347	18q21.1	LIPG	exonic	G/A	0.26	0.0001026	-0.4223	-31.08	30.24	0.9785	0.99531797
HDL-C	PGS000309	rs8099014	18q21.31	NEDD4L;MIR122	intergenic	C/A	0.015	0.2521	-0.405	-1.144	0.3345	0.2831	0.60624375

HDL-C	PGS000309	rs489693	18q21.32	PMAIP1;MC4R	intergenic	A/C	-0.019	0.1902	-0.3798	-1.203	0.443	0.3657	0.66686471
HDL-C	PGS000309	rs12970134	18q21.32	PMAIP1;MC4R	intergenic	A/G	-0.019	0.1454	-0.6427	-1.563	0.2771	0.1709	0.47441625
HDL-C	PGS000309	rs737337	19p13.2	DOCK6	exonic	G/A	-0.058	0.1918	-1.073	-1.89	-0.2559	0.01009	0.06914305
HDL-C	PGS000309	rs891088	19p13.2	INSR	intronic	G/A	0.015	0.2487	0.3425	-0.3924	1.078	0.361	0.66200282
HDL-C	PGS000309	kgp4537709	19p13.2	CLEC4M	exonic	A/G	0.016	0.1109	-0.2493	-1.277	0.7785	0.6346	0.8262492
HDL-C	PGS000309	rs6511720	19p13.2	LDLR	intronic	A/C	0.024	0.0205	-0.9418	-3.2	1.316	0.4137	0.70426364
HDL-C	PGS000309	rs731839	19q13.11	PEPD	intronic	A/G	0.017	0.431	0.09157	-0.5534	0.7365	0.7808	0.89314024
HDL-C	PGS000309	rs769449	19q13.32	APOE	intronic	A/G	-0.098	0.1041	-1.406	-2.454	-0.3588	0.008533	0.06229347
HDL-C	PGS000309	exm1479047	19q13.32	BCAM	exonic	A/G	0.051	0.0002063	-11.74	-33.39	9.921	0.2882	0.60915
HDL-C	PGS000309	rs157580	19q13.32	TOMM40	intronic	G/A	-0.026	0.4829	-0.2711	-0.9183	0.3761	0.4117	0.70426364
HDL-C	PGS000309	rs439401	19q13.32	APOE;APOC1	intergenic	A/G	-0.022	0.4959	-0.2528	-0.9049	0.3993	0.4474	0.73025925
HDL-C	PGS000309	rs8111071	19q13.32	RSPH6A	intronic	G/A	-0.029	0.005025	-0.5653	-5.067	3.936	0.8056	0.90110928
HDL-C	PGS000309	rs2303108	19q13.32	ZC3H4	intronic	G/A	-0.015	0.3599	-0.08068	-0.7482	0.5868	0.8127	0.90505692
HDL-C	PGS000309	rs17695224	19q13.41	FPR3	intronic	A/G	-0.028	0.2253	-0.7049	-1.47	0.06023	0.07103	0.27593263
HDL-C	PGS000309	exm1503916	19q13.42	LILRB5	exonic	G/A	-0.029	0.1783	-0.5526	-1.398	0.2925	0.2001	0.4991
HDL-C	PGS000309	rs12740374	1p13.3	CELSR2	UTR3	A/C	0.045	0.07159	-0.5958	-1.853	0.6617	0.3531	0.65489487
HDL-C	PGS000309	rs646776	1p13.3	CELSR2	downstream	G/A	-0.043	0.0753	-0.7263	-1.91	0.457	0.229	0.5401413
HDL-C	PGS000309	exm2260857	1p22.1	MTF2	intronic	G/A	-0.021	0.385	0.4285	-0.2294	1.086	0.2018	0.49951255
HDL-C	PGS000309	rs4660293	1p34.3	PABPC4	intronic	G/A	-0.04	0.1152	-0.9319	-1.946	0.0823	0.07178	0.27593263
HDL-C	PGS000309	rs12748152	1p36.11	PIGV;ZDHHC18	intergenic	A/G	-0.043	0.02289	-0.9527	-3.094	1.188	0.3832	0.68533846
HDL-C	PGS000309	rs267738	1q21.3	CERS2	exonic	C/A	0.024	0.01619	0.3667	-2.159	2.893	0.776	0.89314024
HDL-C	PGS000309	rs267733	1q21.3	ANXA9	exonic	G/A	0.021	0.01507	-0.01266	-2.632	2.607	0.9924	0.99699444
HDL-C	PGS000309	rs12145743	1q23.1	RRNAD1	intronic	C/A	0.017	0.1089	-0.009209	-1.034	1.015	0.9859	0.99699444
HDL-C	PGS000309	rs1011731	1q24.3	DNM3	intronic	G/A	0.015	0.1851	-0.384	-1.213	0.4449	0.3639	0.66544635
HDL-C	PGS000309	rs4650994	1q25.2	C1orf220	ncRNA_intro	G/A	-0.019	0.4201	-0.329	-0.9857	0.3278	0.3263	0.63696647
HDL-C	PGS000309	rs1689800	1q25.3	ZNF648;LINC01344	intergenic	G/A	-0.025	0.2131	-0.3746	-1.154	0.4047	0.3462	0.64763276
HDL-C	PGS000309	rs2785980	1q41	LYPLAL1-AS1	ncRNA_intro	G/A	0.016	0.2003	0.5476	-0.2672	1.362	0.1879	0.49125663
HDL-C	PGS000309	rs4846914	1q42.13	GALNT2	intronic	A/G	0.049	0.2439	0.8132	0.06115	1.565	0.03412	0.17116292
HDL-C	PGS000309	rs10489615	1q42.13	GALNT2	intronic	G/A	0.046	0.2668	0.8326	0.1009	1.564	0.02579	0.13649829
HDL-C	PGS000309	rs1800961	20q13.12	HNF4A	exonic	A/G	-0.14	0.01138	-2.407	-5.448	0.6339	0.1209	0.38771379
HDL-C	PGS000309	rs181362	22q11.21	UBE2L3	intronic	G/A	-0.028	0.4242	0.08553	-0.5631	0.7341	0.7961	0.89728193
HDL-C	PGS000309	rs4823006	22q12.1	ZNRF3	UTR3	A/G	0.014	0.4179	0.06881	-0.5785	0.7161	0.835	0.91358824
HDL-C	PGS000309	rs17738527	22q12.2	SEC14L4	exonic	T/C	-0.018	0.03115	-1.06	-2.9	0.7795	0.2588	0.5789887
HDL-C	PGS000309	rs738322	22q13.1	PLA2G6	intronic	G/A	0.02	0.4233	0.37	-0.2871	1.027	0.2698	0.5913798
LDL-C	PGS000310	rs12740374	1p13.3	CELSR2	UTR3	A/C	-0.16	0.07159	-4.859	-7.351	-2.366	0.0001349	0.00199591
LDL-C	PGS000310	rs646776	1p13.3	CELSR2	downstream	G/A	0.16	0.0753	-4.827	-7.173	-2.481	0.00005621	0.00104532
LDL-C	PGS000310	rs4245791	2p21	ABCG8	intronic	G/A	-0.072	0.02696	5.893	1.957	9.829	0.003359	0.03169143
LDL-C	PGS000310	rs676210	2p24.1	APOB	exonic	G/A	-0.039	0.3095	-2.074	-3.452	-0.6953	0.003209	0.03072146
LDL-C	PGS000310	rs1367117	2p24.1	APOB	exonic	A/G	0.11	0.1396	2.688	0.8511	4.525	0.004149	0.03763142
LDL-C	PGS000310	rs3846662	5q13.3	HMGCR	intronic	A/G	0.065	0.4673	-1.983	-3.264	-0.7015	0.002434	0.02400809
LDL-C	PGS000310	rs3798220	6q25.3	LPA	exonic	G/A	0.11	0.06405	-4.085	-6.662	-1.508	0.001902	0.02029839
LDL-C	PGS000310	rs1883025	9q31.1	ABCA1	intronic	A/G	-0.024	0.2555	-2.244	-3.7	-0.7874	0.002544	0.02471857
LDL-C	PGS000310	rs635634	9q34.2	ABO;SURF6	intergenic	A/G	0.077	0.1469	3.985	2.188	5.781	0.00001405	0.00031652
LDL-C	PGS000310	exm2272816	19p13.2	SPC24	exonic	A/G	0.024	0.2684	-3.01	-4.452	-1.569	0.00004317	0.00085222
LDL-C	PGS000310	rs769449	19q13.32	APOE	intronic	A/G	0.19	0.1041	7.826	5.757	9.894	1.431E-13	1.3299E-11
LDL-C	PGS000310	rs7412	19q13.32	APOE	exonic	A/G	-0.54	0.08845	-18.44	-20.65	-16.22	3.461E-58	2.2525E-55
LDL-C	PGS000310	rs445925	19q13.32	APOE;APOC1	intergenic	A/G	-0.32	0.1098	-13.64	-15.64	-11.65	4.305E-40	1.4029E-37

LDL-C	PGS000310	rs7515577	1p22.1	EVI5	intronic	C/A	0.03	0.03601	-1.165	-4.578	2.248	0.5036	0.76598972
LDL-C	PGS000310	rs10889353	1p31.3	DOCK7	intronic	C/A	-0.045	0.2645	-1.446	-2.893	0.0003996	0.05012	0.21898067
LDL-C	PGS000310	rs11206510	1p32.3	BSND;PCSK9	intergenic	G/A	-0.07	0.04683	-3.887	-6.934	-0.8393	0.01246	0.07875204
LDL-C	PGS000310	rs2479409	1p32.3	PCSK9	upstream	A/G	-0.047	0.4232	-0.3392	-1.637	0.9582	0.6084	0.80665662
LDL-C	PGS000310	rs505151	1p32.3	PCSK9	exonic	G/A	-0.09	0.03229	1.12	-2.462	4.701	0.54	0.77813974
LDL-C	PGS000310	rs10903129	1p36.11	MACO1	intronic	G/A	0.028	0.1998	-0.6981	-2.292	0.8956	0.3907	0.69274891
LDL-C	PGS000310	rs12748152	1p36.11	PIGV;ZDHHC18	intergenic	A/G	0.031	0.02289	-1.284	-5.543	2.975	0.5546	0.77813974
LDL-C	PGS000310	rs267733	1q21.3	ANXA9	exonic	G/A	-0.025	0.01507	-4.382	-9.577	0.8133	0.09837	0.33883
LDL-C	PGS000310	exm-rs11125936	2p15	TMEM17;EHBP1	intergenic	G/A	-0.028	0.1327	0.8582	-1.012	2.728	0.3684	0.66804568
LDL-C	PGS000310	rs1260326	2p23.3	GCKR	exonic	A/G	-0.023	0.3761	0.5731	-0.7314	1.878	0.3893	0.69244344
LDL-C	PGS000310	rs541041	2p24.1	APOB;TDRD15	intergenic	G/A	0.12	0.01989	-5.705	-10.26	-1.148	0.01417	0.08675944
LDL-C	PGS000310	rs533617	2p24.1	APOB	exonic	G/A	-0.13	0.003382	-4.178	-15.17	6.815	0.4563	0.7394194
LDL-C	PGS000310	rs17526895	2q14.1	CCDC93;INSIG2	intergenic	G/A	-0.054	0.003897	-0.393	-10.72	9.935	0.9406	0.98004144
LDL-C	PGS000310	rs10490626	2q14.1	CCDC93;INSIG2	intergenic	A/G	-0.053	0.003894	-0.3908	-10.72	9.939	0.9409	0.98004144
LDL-C	PGS000310	rs2030746	2q14.2	LINC01101;GLI2	intergenic	G/A	0.014	0.4586	-0.8526	-2.117	0.4117	0.1863	0.49101741
LDL-C	PGS000310	rs2287623	2q31.1	ABCB11	intronic	G/A	-0.021	0.2396	0.8673	-0.6059	2.341	0.2486	0.57059579
LDL-C	PGS000310	rs1250229	2q35	FN1;LOC102724849	intergenic	A/G	-0.024	0.1674	-0.8527	-2.569	0.864	0.3304	0.63825045
LDL-C	PGS000310	rs887829	2q37.1	UGT1A10;UGT1A3;UGT1A4;U	intronic	A/G	-0.022	0.1454	-0.5011	-2.378	1.376	0.6009	0.79997117
LDL-C	PGS000310	rs11563251	2q37.1	UGT1A1;UGT1A10;UGT1A3;U	intronic	A/G	0.034	0.1637	-0.4639	-2.18	1.252	0.5962	0.79640779
LDL-C	PGS000310	rs13315871	3p14.3	PXK	intronic	A/G	-0.038	0.007995	-5.369	-12.49	1.751	0.1395	0.4188
LDL-C	PGS000310	rs2251219	3p21.1	PBRM1	exonic	G/A	0.016	0.3224	-0.3815	-1.756	0.9928	0.5864	0.79365156
LDL-C	PGS000310	rs7640978	3p22.3	CMTM6	intronic	A/G	-0.033	0.09686	-2.043	-4.27	0.1829	0.0721	0.27593263
LDL-C	PGS000310	rs17404153	3q22.1	DNAJC13	intronic	A/C	-0.034	0.141	-0.03984	-1.879	1.799	0.9661	0.99200489
LDL-C	PGS000310	rs6831256	4p16.3	DOK7	intronic	G/A	0.022	0.4068	-0.2275	-1.529	1.074	0.732	0.87469215
LDL-C	PGS000310	rs976002	4q13.2	TMPRSS11E	exonic	G/A	0.023	0.07856	-0.05665	-2.45	2.337	0.963	0.99110379
LDL-C	PGS000310	rs3816873	4q23	MTTP	exonic	G/A	-0.017	0.2295	-0.5107	-2.047	1.025	0.5147	0.76850849
LDL-C	PGS000310	rs13107325	4q24	SLC39A8	exonic	A/G	-0.035	0.000415	5.933	-29.36	41.23	0.7418	0.87719891
LDL-C	PGS000310	rs13146272	4q35.1	CYP4V2	exonic	C/A	-0.015	0.4861	0.007049	-1.269	1.283	0.9914	0.99699444
LDL-C	PGS000310	rs4530754	5q23.2	CSNK1G3	intronic	A/G	0.017	0.3315	-0.05513	-1.397	1.287	0.9358	0.98004144
LDL-C	PGS000310	rs1016988	5q31.1	SLC22A5;IRF1-AS1	intergenic	G/A	-0.02	0.2953	-0.5434	-1.935	0.8478	0.444	0.72807053
LDL-C	PGS000310	rs6882076	5q33.3	TIMD4	upstream	A/G	0.039	0.2888	-0.9419	-2.364	0.4804	0.1944	0.49531512
LDL-C	PGS000310	rs351855	5q35.2	FGFR4	exonic	A/G	-0.018	0.4621	-1.518	-2.797	-0.2389	0.02006	0.1116159
LDL-C	PGS000310	rs13192471	6p21.32	HLA-DQB1;HLA-DQA2	intergenic	G/A	0.038	0.1559	-0.6448	-2.426	1.136	0.4779	0.75273841
LDL-C	PGS000310	kgp11460585	6p21.33	HLA-B;MICA-AS1	intergenic	A/G	0.018	0.3697	0.4527	-0.9644	1.87	0.5313	0.77813974
LDL-C	PGS000310	rs1264562	6p22.1	RPP21;HLA-E	intergenic	C/A	0.015	0.3276	1.253	-0.4577	2.963	0.1512	0.439425
LDL-C	PGS000310	rs3757354	6p22.3	DTNBP1;MYLIP	intergenic	A/G	-0.033	0.3911	-0.8182	-2.133	0.4964	0.2226	0.53081538
LDL-C	PGS000310	rs9370867	6p22.3	MYLIP	exonic	A/G	-0.033	0.08356	2.268	-0.02445	4.56	0.05256	0.22436098
LDL-C	PGS000310	rs1999930	6q22.1	FRK;NT5DC1	intergenic	A/G	-0.018	0.01425	0.1618	-5.144	5.467	0.9523	0.98717723
LDL-C	PGS000310	rs9376090	6q23.3	HBS1L;MYB	intergenic	G/A	-0.025	0.1501	-0.3246	-2.125	1.476	0.7238	0.87469215
LDL-C	PGS000310	rs12208357	6q25.3	SLC22A1	exonic	A/G	0.058	0.001051	-1.87	-21.13	17.39	0.8491	0.92590302
LDL-C	PGS000310	rs1564348	6q25.3	SLC22A1	intronic	G/A	0.047	0.01866	-1.423	-6.143	3.297	0.5546	0.77813974
LDL-C	PGS000310	rs7758229	6q25.3	SLC22A3	intronic	A/C	0.016	0.3176	0.9615	-0.408	2.331	0.1689	0.47302275
LDL-C	PGS000310	rs7770628	6q25.3	LPA	intronic	G/A	-0.031	0.1267	0.3299	-1.56	2.22	0.7323	0.87469215
LDL-C	PGS000310	rs11550029	7p13	NUDCD3	exonic	A/G	0.02	0.01342	2.049	-3.525	7.624	0.4712	0.74920438
LDL-C	PGS000310	rs35803101	7p13	NPC1L1	exonic	A/G	-0.16	0.0005126	14.59	-12.66	41.84	0.2941	0.61169042
LDL-C	PGS000310	rs4722551	7p15.2	MIR148A;NFE2L3	intergenic	G/A	0.04	0.02798	4.404	0.5515	8.256	0.0251	0.13393525
LDL-C	PGS000310	rs12670798	7p15.3	DNAH11	intronic	A/G	0.033	0.4603	-0.007834	-1.276	1.26	0.9903	0.99699444

LDL-C	PGS000310	rs4921914	8p22	NAT2;PSD3	intergenic	G/A	-0.022	0.3933	1.557	0.2522	2.863	0.0194	0.10921086
LDL-C	PGS000310	rs1495741	8p22	NAT2;PSD3	intergenic	G/A	-0.022	0.397	1.513	0.209	2.816	0.023	0.124775
LDL-C	PGS000310	rs4841132	8p23.1	LOC157273	ncRNA_exoni	A/G	0.057	0.03069	-1.818	-5.591	1.956	0.3452	0.64762305
LDL-C	PGS000310	rs10102164	8q11.23	SOX17;RP1	intergenic	A/G	0.031	0.2227	0.3527	-1.163	1.868	0.6484	0.83585822
LDL-C	PGS000310	rs2081687	8q12.1	UBXN2B;CYP7A1	intergenic	A/G	-0.028	0.2908	0.9464	-0.4531	2.346	0.1851	0.49101741
LDL-C	PGS000310	rs2293889	8q23.3	TRPS1	intronic	A/C	-0.015	0.1885	1.959	0.3256	3.593	0.01879	0.10825035
LDL-C	PGS000310	rs2737229	8q23.3	TRPS1	intronic	C/A	-0.022	0.3773	0.346	-0.9662	1.658	0.6054	0.80431714
LDL-C	PGS000310	rs2954033	8q24.13	TRIB1;LINC00861	intergenic	A/G	-0.035	0.2775	1.476	0.05888	2.893	0.04127	0.19328612
LDL-C	PGS000310	rs2954038	8q24.13	TRIB1;LINC00861	intergenic	C/A	-0.042	0.2754	1.356	-0.0676	2.779	0.06198	0.24753975
LDL-C	PGS000310	rs77375493	9p24.1	JAK2	exonic	A/C	-0.3	0.0001025	26.04	-34.69	86.77	0.4007	0.70056676
LDL-C	PGS000310	rs3780181	9p24.2	VLDLR	intronic	G/A	-0.037	0.1093	-2.659	-4.695	-0.6229	0.01051	0.07053619
LDL-C	PGS000310	rs4149268	9q31.1	ABCA1	intronic	A/G	-0.015	0.3628	-1.562	-2.896	-0.2273	0.02184	0.12049017
LDL-C	PGS000310	rs3890182	9q31.1	ABCA1	intronic	A/G	-0.027	0.06848	0.0437	-2.487	2.575	0.973	0.99463427
LDL-C	PGS000310	rs687621	9q34.2	ABO	intronic	G/A	0.043	0.4054	1.734	0.4384	3.029	0.008736	0.062496
LDL-C	PGS000310	rs12355784	10q21.3	JMJD1C	intronic	A/C	0.018	0.2788	0.5903	-0.8334	2.014	0.4165	0.70426364
LDL-C	PGS000310	rs2068888	10q23.33	CYP26A1;MYOF	intergenic	G/A	-0.016	0.1926	-0.4081	-2.024	1.208	0.6206	0.81776121
LDL-C	PGS000310	rs2255141	10q25.2	GPAM	intronic	A/G	-0.028	0.1791	0.7287	-0.9612	2.419	0.3981	0.70044081
LDL-C	PGS000310	rs1891110	10q26.13	FAM24B	exonic	A/G	0.021	0.4605	0.593	-0.6785	1.864	0.3607	0.66200282
LDL-C	PGS000310	rs10128711	11p15.1	SPTY2D1	intronic	A/G	0.025	0.4934	0.3967	-0.9032	1.697	0.5498	0.77813974
LDL-C	PGS000310	exm2264444	11p15.1	SPTY2D1	intronic	C/A	0.025	0.4859	0.2302	-1.06	1.52	0.7265	0.87469215
LDL-C	PGS000310	rs174546	11q12.2	FADS1	UTR3	G/A	-0.053	0.3075	1.479	0.08093	2.878	0.03819	0.18280654
LDL-C	PGS000310	rs174547	11q12.2	FADS1	intronic	A/G	-0.053	0.3076	1.491	0.09356	2.889	0.03657	0.1776647
LDL-C	PGS000310	rs174550	11q12.2	FADS1	intronic	A/G	-0.053	0.3076	1.491	0.09356	2.889	0.03657	0.1776647
LDL-C	PGS000310	rs7350481	11q23.3	LINC02702;BUD13	intergenic	A/G	-0.028	0.247	1.635	0.1738	3.096	0.02835	0.146475
LDL-C	PGS000310	rs10790162	11q23.3	BUD13	intronic	A/G	-0.033	0.2204	1.908	0.3788	3.438	0.01451	0.08746306
LDL-C	PGS000310	rs7941030	11q24.1	MIR100HG;UBASH3B	intergenic	G/A	0.014	0.344	-0.5692	-1.942	0.8032	0.4163	0.70426364
LDL-C	PGS000310	rs8177399	11q24.2	TIRAP	exonic	A/G	0.061	0.0007175	-12.74	-37.59	12.11	0.3149	0.62777835
LDL-C	PGS000310	rs11220462	11q24.2	ST3GAL4	intronic	A/G	0.043	0.3339	0.1785	-1.154	1.511	0.7929	0.89659427
LDL-C	PGS000310	rs4883201	12p13.31	PHC1	intronic	G/A	-0.022	0.2937	-0.7959	-2.196	0.6039	0.2652	0.58523797
LDL-C	PGS000310	exm1022117	12q21.1	RAB21	exonic	A/G	0.057	0.0001026	30.86	-29.9	91.62	0.3196	0.63048364
LDL-C	PGS000310	rs3184504	12q24.12	SH2B3	exonic	A/G	0.027	0.006251	-2.165	-10.19	5.861	0.597	0.79640779
LDL-C	PGS000310	rs1169288	12q24.31	HNF1A	exonic	C/A	0.037	0.299	-0.003861	-1.408	1.4	0.9957	0.998
LDL-C	PGS000310	rs1183910	12q24.31	HNF1A	intronic	A/G	0.036	0.2865	0.6855	-0.7238	2.095	0.3404	0.6462689
LDL-C	PGS000310	rs7310409	12q24.31	HNF1A	intronic	A/G	-0.027	0.3142	0.5657	-0.8069	1.938	0.4193	0.7071614
LDL-C	PGS000310	rs11057830	12q24.31	SCARB1	intronic	A/G	0.023	0.07821	0.7199	-1.657	3.096	0.5527	0.77813974
LDL-C	PGS000310	rs4942486	13q13.1	BRCA2	intronic	A/G	-0.022	0.488	1.759	0.4943	3.023	0.00643	0.05232413
LDL-C	PGS000310	rs8017377	14q12	NYNRIN	exonic	A/G	0.023	0.05912	2.028	-0.6722	4.729	0.1411	0.42135826
LDL-C	PGS000310	rs173539	16q13	HERPUD1;CETP	intergenic	A/G	-0.033	0.2252	-0.7966	-2.321	0.728	0.3058	0.6233325
LDL-C	PGS000310	rs247616	16q13	HERPUD1;CETP	intergenic	A/G	-0.032	0.1807	-1.247	-2.9	0.407	0.1396	0.4188
LDL-C	PGS000310	rs3764261	16q13	HERPUD1;CETP	intergenic	A/C	-0.032	0.1807	-1.365	-3.023	0.292	0.1064	0.35621385
LDL-C	PGS000310	rs9939224	16q13	CETP	intronic	A/C	-0.023	0.1386	-0.3037	-2.125	1.517	0.7438	0.87719891
LDL-C	PGS000310	rs34832584	16q22.2	PMFBP1	exonic	T/G	0.02	0.03115	-0.1509	-3.887	3.585	0.9369	0.98004144
LDL-C	PGS000310	rs314253	17p13.1	DLG4	UTR3	G/A	-0.02	0.4013	-0.2461	-1.529	1.037	0.7069	0.86636842
LDL-C	PGS000310	rs1801689	17q24.2	APOH	exonic	C/A	0.1	0.0004099	7.244	-23.18	37.67	0.6407	0.8292161
LDL-C	PGS000310	exm1349142	17q24.2	ABCA6	exonic	G/A	0.19	0.0001025	4.955	-55.84	65.75	0.8731	0.9410399
LDL-C	PGS000310	rs2125345	17q25.1	UNK	intronic	G/A	-0.024	0.4242	0.322	-0.9574	1.601	0.6218	0.81776121
LDL-C	PGS000310	rs4129767	17q25.3	PGS1	intronic	A/G	0.017	0.4188	0.8483	-0.4326	2.129	0.1944	0.49531512

LDL-C	PGS000310	rs77960347	18q21.1	LIPG	exonic	G/A	0.083	0.0001026	13.84	-47	74.67	0.6558	0.84094932
LDL-C	PGS000310	rs7241918	18q21.1	LIPG;ACAA2	intergenic	C/A	0.02	0.1563	0.5937	-1.157	2.344	0.5063	0.7683014
LDL-C	PGS000310	rs58542926	19p13.11	TM6SF2	exonic	A/G	-0.1	0.1166	-1.293	-3.288	0.7024	0.2042	0.50342368
LDL-C	PGS000310	rs6511720	19p13.2	LDLR	intronic	A/C	-0.21	0.0205	-2.801	-7.291	1.689	0.2215	0.53081538
LDL-C	PGS000310	rs11669576	19p13.2	LDLR	exonic	A/G	0.058	0.0001025	0.9193	-59.83	61.67	0.9763	0.99463427
LDL-C	PGS000310	exm1479047	19q13.32	BCAM	exonic	A/G	-0.27	0.0002063	14.11	-28.81	57.03	0.5195	0.77213356
LDL-C	PGS000310	rs157580	19q13.32	TOMM40	intronic	G/A	0.072	0.4829	0.7496	-0.5347	2.034	0.2527	0.57319756
LDL-C	PGS000310	rs492602	19q13.33	FUT2	exonic	G/A	0.028	0.02819	-0.1856	-4.07	3.699	0.9254	0.97377
LDL-C	PGS000310	rs2287922	19q13.33	RASIP1	exonic	A/G	0.026	0.03284	-0.3593	-4.001	3.283	0.8467	0.92483507
LDL-C	PGS000310	rs2328223	20p12.1	BANF2;SNX5	intergenic	C/A	0.03	0.1649	1.781	0.06707	3.496	0.04174	0.194091
LDL-C	PGS000310	rs364585	20p12.1	LINC01722;SPTLC3	intergenic	A/G	0.019	0.4228	-1.138	-2.426	0.1497	0.08332	0.30818932
LDL-C	PGS000310	rs2902940	20q12	LINC01370;MAFB	intergenic	G/A	-0.027	0.305	0.412	-0.9712	1.795	0.5594	0.77813974
LDL-C	PGS000310	rs1800961	20q13.12	HNF4A	exonic	A/G	-0.054	0.01138	4.113	-1.92	10.15	0.1815	0.48825
LDL-C	PGS000310	rs4809330	20q13.33	ZGPAT	intronic	G/A	-0.015	0.4568	0.04441	-1.213	1.302	0.9448	0.98253163
LDL-C	PGS000310	exm2268318	20q13.33	TCEA2	intronic	G/A	-0.014	0.4137	0.7072	-0.5883	2.003	0.2847	0.60767115
LDL-C	PGS000310	rs5763662	22q12.2	MTMR3	intronic	A/G	0.077	0.121	0.8343	-1.131	2.799	0.4053	0.70426364
LDL-C	PGS000310	rs138777	22q12.3	TOM1	intronic	G/A	-0.015	0.4991	1.149	-0.1251	2.423	0.07722	0.29057931
LDL-C	PGS000310	rs13268	22q13.31	FBLN1	exonic	G/A	-0.053	0.0005135	-10.9	-38.11	16.3	0.4323	0.71792679
LDL-C	PGS000310	22:46627603	22q13.31	PPARA	intronic	A/G	0.031	0.004202	-4.268	-14.35	5.818	0.407	0.70426364
T2D	PGS004106	rs7578597	2p21	THADA	exonic	G/A	-0.13	0.02513	0.8807	0.4916	1.578	0.6695	0.84465988
T2D	PGS004106	rs7615580	3p25.2	TAMM41;SYN2	intergenic	C/T	-0.15	0.02316	0.8127	0.4362	1.514	0.5137	0.76850849
T2D	PGS004106	rs17036160	3p25.2	PPARG	intronic	A/G	0.14	0.02914	0.603	0.3166	1.148	0.1237	0.39091602
T2D	PGS004106	rs11708067	3q21.1	ADCY5	intronic	G/A	-0.11	0.02662	0.8278	0.4703	1.457	0.5122	0.76850849
T2D	PGS004106	rs4402960	3q27.2	IGF2BP2	intronic	A/C	-0.14	0.2715	1.098	0.9098	1.325	0.3301	0.63825045
T2D	PGS004106	rs734312	4p16.1	WFS1	exonic	G/A	-0.094	0.2437	0.9891	0.8134	1.203	0.9124	0.97182724
T2D	PGS004106	rs6931514	6p22.3	CDKAL1	intronic	G/A	0.17	0.4173	1.053	0.8829	1.255	0.5679	0.77996392
T2D	PGS004106	rs1635852	7p15.1	JAZF1	intronic	G/A	-0.092	0.2405	0.9708	0.7935	1.188	0.7732	0.89314024
T2D	PGS004106	rs3802177	8q24.11	SLC30A8	UTR3	A/G	0.11	0.4365	0.895	0.7566	1.059	0.1955	0.49531512
T2D	PGS004106	rs4977756	9p21.3	CDKN2B-AS1	ncRNA_intro	G/A	-0.073	0.2037	0.9997	0.8095	1.235	0.9974	0.998
T2D	PGS004106	rs10811661	9p21.3	CDKN2B-AS1;DMRTA1	intergenic	G/A	-0.13	0.3969	0.8582	0.7191	1.024	0.09018	0.32371154
T2D	PGS004106	rs7018475	9p21.3	CDKN2B-AS1;DMRTA1	intergenic	C/A	0.12	0.3723	0.9576	0.8045	1.14	0.6259	0.82149375
T2D	PGS004106	JHU_10.114745787	10q25.2	TCF7L2	intronic	A/G	0.17	0.05967	0.8214	0.5659	1.192	0.3008	0.62112816
T2D	PGS004106	rs7903146	10q25.2	TCF7L2	intronic	A/G	-0.29	0.04919	1.062	0.7277	1.55	0.7545	0.88557038
T2D	PGS004106	rs10885410	10q25.2	TCF7L2	intronic	A/G	0.1	0.06794	1.102	0.7996	1.52	0.5519	0.77813974
T2D	PGS004106	GSA-rs11196229	10q25.3	TCF7L2	intronic	A/G	0.086	0.07472	1.45	1.084	1.939	0.01234	0.07875204
T2D	PGS004106	rs6585827	10q26.13	PLEKHA1	intronic	G/A	0.085	0.4035	1.117	0.9406	1.327	0.2069	0.50355336
T2D	PGS004106	rs163184	11p15.4	KCNQ1	intronic	C/A	0.09	0.4224	1.024	0.8645	1.212	0.7857	0.89314024
T2D	PGS004106	rs2237896	11p15.4	KCNQ1	intronic	A/G	0.22	0.3042	0.9595	0.7959	1.157	0.6647	0.84350819
T2D	PGS004106	exm-rs11063069	12p13.32	CCND2-AS1	ncRNA_intro	G/A	0.098	0.04944	1.015	0.6929	1.487	0.9387	0.98004144
T2D	PGS004106	rs1215451	13q31.1	LINC01080;SPRY2	intergenic	A/G	0.085	0.1956	1.066	0.8608	1.319	0.5591	0.77813974
T2D	PGS004106	rs7178572	15q24.3	HMG20A	intronic	G/A	0.082	0.3337	1.061	0.8898	1.264	0.5114	0.76850849
T2D	PGS004106	rs17817449	16q12.2	FTO	intronic	C/A	0.12	0.244	1.293	1.069	1.564	0.008217	0.06176269
T2D	PGS004106	rs4420638	19q13.32	APOC1	downstream	G/A	-0.11	0.1397	0.9171	0.7174	1.172	0.4898	0.76100191
TC	PGS000311	rs12740374	1p13.3	CELSR2	UTR3	A/C	-0.13	0.07159	-4.93	-7.615	-2.245	0.0003229	0.00420416
TC	PGS000311	rs646776	1p13.3	CELSR2	downstream	G/A	0.13	0.0753	-5.154	-7.681	-2.628	0.00006482	0.00111013
TC	PGS000311	rs1260326	2p23.3	GCKR	exonic	A/G	-0.057	0.3761	2.066	0.6633	3.468	0.003907	0.0363351
TC	PGS000311	rs1260333	2p23.3	GCKR;C2orf16	intergenic	A/G	-0.045	0.3744	2.108	0.6228	3.592	0.005425	0.047089

TC	PGS000311	rs3846662	5q13.3	HMGCR	intronic	A/G	0.062	0.4673	-2.443	-3.821	-1.065	0.0005156	0.00633312
TC	PGS000311	rs3798220	6q25.3	LPA	exonic	G/A	0.11	0.06405	-4.41	-7.185	-1.635	0.001852	0.0200942
TC	PGS000311	rs4149268	9q31.1	ABCA1	intronic	A/G	-0.031	0.3628	-3.353	-4.788	-1.918	4.764E-06	0.00012912
TC	PGS000311	rs1883025	9q31.1	ABCA1	intronic	A/G	-0.054	0.2555	-4.933	-6.496	-3.37	6.697E-10	3.3552E-08
TC	PGS000311	rs635634	9q34.2	ABO;SURF6	intergenic	A/G	0.073	0.1469	3.138	1.202	5.074	0.001495	0.01649568
TC	PGS000311	rs7350481	11q23.3	LINC02702;BUD13	intergenic	A/G	-0.082	0.247	3.315	1.745	4.886	0.00003586	0.00073034
TC	PGS000311	rs10790162	11q23.3	BUD13	intronic	A/G	-0.09	0.2204	3.282	1.636	4.927	0.00009398	0.00152985
TC	PGS000311	rs10468017	15q21.3	AQP9;LIPC	intergenic	A/G	0.048	0.1941	2.747	1.005	4.489	0.002007	0.020863
TC	PGS000311	rs1532085	15q21.3	AQP9;LIPC	intergenic	A/G	-0.042	0.4751	3.443	2.071	4.814	9.009E-07	2.9328E-05
TC	PGS000311	exm2272816	19p13.2	SPC24	exonic	A/G	0.019	0.2684	-2.82	-4.372	-1.268	0.0003736	0.00467718
TC	PGS000311	rs737337	19p13.2	DOCK6	exonic	G/A	-0.036	0.1918	-3.566	-5.309	-1.824	0.0000615	0.00111013
TC	PGS000311	rs769449	19q13.32	APOE	intronic	A/G	0.16	0.1041	6.986	4.755	9.217	9.076E-10	4.2222E-08
TC	PGS000311	rs7412	19q13.32	APOE	exonic	A/G	-0.37	0.08845	-13.4	-15.83	-10.98	4.789E-27	1.0394E-24
TC	PGS000311	rs445925	19q13.32	APOE;APOC1	intergenic	A/G	-0.21	0.1098	-9.611	-11.79	-7.436	6.364E-18	6.9006E-16
TC	PGS000311	rs7515577	1p22.1	EVI5	intronic	C/A	0.031	0.03601	-1.542	-5.219	2.134	0.411	0.70426364
TC	PGS000311	rs10889353	1p31.3	DOCK7	intronic	C/A	-0.072	0.2645	-1.872	-3.43	-0.3148	0.01852	0.1076475
TC	PGS000311	rs11206510	1p32.3	BSND;PCSK9	intergenic	G/A	-0.064	0.04683	-3.206	-6.484	0.07143	0.05527	0.23364136
TC	PGS000311	rs2479409	1p32.3	PCSK9	upstream	A/G	-0.04	0.4232	0.4326	-0.9637	1.829	0.5437	0.77813974
TC	PGS000311	rs505151	1p32.3	PCSK9	exonic	G/A	-0.08	0.03229	1.334	-2.514	5.182	0.4969	0.76251247
TC	PGS000311	rs10903129	1p36.11	MACO1	intronic	G/A	0.028	0.1998	-0.2697	-1.982	1.443	0.7577	0.88557038
TC	PGS000311	rs1077514	1p36.12	ASAP3	intronic	G/A	0.027	0.4271	0.1893	-1.187	1.565	0.7875	0.89314024
TC	PGS000311	exm-rs11125936	2p15	TMEM17;EHBP1	intergenic	G/A	-0.026	0.1327	0.5908	-1.423	2.604	0.5652	0.77996392
TC	PGS000311	rs4245791	2p21	ABCG8	intronic	G/A	-0.068	0.02696	4.295	0.06422	8.525	0.04668	0.20765571
TC	PGS000311	rs1049817	2p23.3	GTF3C2	exonic	G/A	-0.022	0.2179	-0.194	-1.849	1.461	0.8183	0.905975
TC	PGS000311	rs676210	2p24.1	APOB	exonic	G/A	-0.037	0.3095	-1.522	-3.007	-0.03738	0.04456	0.20144833
TC	PGS000311	rs1367117	2p24.1	APOB	exonic	A/G	0.092	0.1396	2.59	0.6126	4.568	0.01029	0.06977906
TC	PGS000311	rs533617	2p24.1	APOB	exonic	G/A	-0.11	0.003382	-3.544	-15.39	8.303	0.5577	0.77813974
TC	PGS000311	rs541041	2p24.1	APOB;TDRD15	intergenic	G/A	0.11	0.01989	-4.751	-9.661	0.1599	0.058	0.23886377
TC	PGS000311	rs6734238	2q14.1	IL1F10;IL1RN	intergenic	G/A	-0.014	0.1098	-0.8336	-3.061	1.393	0.4632	0.74455111
TC	PGS000311	rs17526895	2q14.1	CCDC93;INSIG2	intergenic	G/A	-0.047	0.003897	-8.054	-19.18	3.075	0.1561	0.44656316
TC	PGS000311	rs10490626	2q14.1	CCDC93;INSIG2	intergenic	A/G	-0.047	0.003894	-8.055	-19.18	3.075	0.1561	0.44656316
TC	PGS000311	rs2030746	2q14.2	LINC01101;GLI2	intergenic	G/A	0.014	0.4586	-0.6507	-2.012	0.7103	0.3488	0.65062693
TC	PGS000311	exm229906	2q21.3	LCT	exonic	A/G	-0.022	0.4789	-0.1762	-1.545	1.192	0.8008	0.89728193
TC	PGS000311	rs2287623	2q31.1	ABCB11	intronic	G/A	-0.021	0.2396	1.145	-0.4384	2.729	0.1564	0.44656316
TC	PGS000311	rs6435161	2q33.2	FAM117B	intronic	G/T	-0.027	0.06671	0.3044	-2.435	3.044	0.8276	0.90712576
TC	PGS000311	rs887829	2q37.1	UGT1A10;UGT1A3;UGT1A4;U(	intronic	A/G	-0.023	0.1454	-0.5684	-2.583	1.446	0.5804	0.78960875
TC	PGS000311	rs11563251	2q37.1	UGT1A1;UGT1A10;UGT1A3;U(	intronic	A/G	0.037	0.1637	-0.8959	-2.742	0.9499	0.3415	0.6462689
TC	PGS000311	rs13315871	3p14.3	PXK	intronic	A/G	-0.032	0.007995	-5.067	-12.74	2.606	0.1956	0.49531512
TC	PGS000311	rs2073498	3p21.31	RASSF1	exonic	A/C	-0.025	0.03966	-0.5203	-4.069	3.028	0.7738	0.89314024
TC	PGS000311	rs7640978	3p22.3	CMTM6	intronic	A/G	-0.031	0.09686	-1.091	-3.491	1.309	0.3731	0.67419862
TC	PGS000311	rs11708067	3q21.1	ADCY5	intronic	G/A	-0.018	0.02662	-0.2013	-4.529	4.126	0.9274	0.97377
TC	PGS000311	exm384446	4p16.3	HGFAC	exonic	A/C	0.025	0.317	0.7088	-0.7582	2.176	0.3437	0.64667254
TC	PGS000311	rs6831256	4p16.3	DOK7	intronic	G/A	0.016	0.4068	0.3836	-1.017	1.784	0.5915	0.79640779
TC	PGS000311	rs976002	4q13.2	TMPRSS11E	exonic	G/A	0.029	0.07856	-0.4585	-3.037	2.12	0.7274	0.87469215
TC	PGS000311	rs442177	4q22.1	AFF1	intronic	C/A	0.013	0.4135	0.3142	-1.071	1.7	0.6567	0.84094932
TC	PGS000311	rs13107325	4q24	SLC39A8	exonic	A/G	-0.043	0.000415	11.91	-26.12	49.93	0.5395	0.77813974
TC	PGS000311	rs6054	4q31.3	FGB	exonic	A/G	0.14	0.001128	15.14	-6.707	36.98	0.1745	0.47441625

TC	PGS000311	rs4530754	5q23.2	CSNK1G3	intronic	A/G		0.015	0.3315	-0.4533	-1.897	0.9905	0.5383	0.77813974
TC	PGS000311	rs6882076	5q33.3	TIMD4	upstream	A/G		0.044	0.2888	-0.7875	-2.319	0.7438	0.3135	0.62777835
TC	PGS000311	rs6905288	6p21.1	VEGFA;LINC02537	intergenic	G/A		0.014	0.2989	-1.159	-2.657	0.3391	0.1295	0.40331
TC	PGS000311	rs2814982	6p21.31	SPDEF;ILRUN	intergenic	A/G		-0.025	0.04912	-0.5487	-3.71	2.613	0.7338	0.87469215
TC	PGS000311	rs9271366	6p21.32	HLA-DRB1;HLA-DQA1	intergenic	G/A		0.024	0.1898	-0.5176	-2.29	1.255	0.5672	0.77996392
TC	PGS000311	rs13192471	6p21.32	HLA-DQB1;HLA-DQA2	intergenic	G/A		0.038	0.1559	-1.647	-3.563	0.2692	0.09214	0.32777672
TC	PGS000311	rs9275596	6p21.32	HLA-DQB1;HLA-DQA2	intergenic	G/A		0.018	0.09854	-0.3381	-3.144	2.468	0.8133	0.90505692
TC	PGS000311	rs3130564	6p21.33	PSORS1C1	intronic	A/G		-0.028	0.000615	-0.8265	-27.62	25.97	0.9518	0.98717723
TC	PGS000311	kgp11460585	6p21.33	HLA-B;MICA-AS1	intergenic	A/G		0.028	0.3697	0.009558	-1.516	1.535	0.9902	0.99699444
TC	PGS000311	exm-rs11751198	6p21.33	VARS1	intronic	A/G		0.047	0.00861	-4.602	-11.93	2.728	0.2186	0.52902825
TC	PGS000311	rs1150754	6p21.33	TNXB	intronic	A/G		-0.028	0.01948	-2.905	-7.726	1.916	0.2376	0.55242
TC	PGS000311	rs3757354	6p22.3	DTNBP1;MYLIP	intergenic	A/G		-0.031	0.3911	-0.5269	-1.942	0.8881	0.4655	0.74457125
TC	PGS000311	rs9370867	6p22.3	MYLIP	exonic	A/G		-0.027	0.08356	2.41	-0.05971	4.88	0.05586	0.234612
TC	PGS000311	rs1999930	6q22.1	FRK;NT5DC1	intergenic	A/G		-0.019	0.01425	-0.3518	-6.07	5.366	0.904	0.96634483
TC	PGS000311	rs9376090	6q23.3	HBS1L;MYB	intergenic	G/A		-0.03	0.1501	-0.6402	-2.578	1.298	0.5173	0.77062311
TC	PGS000311	rs12208357	6q25.3	SLC22A1	exonic	A/G		0.05	0.001051	2.36	-18.39	23.11	0.8236	0.90712576
TC	PGS000311	rs1564348	6q25.3	SLC22A1	intronic	G/A		0.045	0.01866	-2.464	-7.535	2.607	0.3409	0.6462689
TC	PGS000311	rs7758229	6q25.3	SLC22A3	intronic	A/C		0.015	0.3176	1.18	-0.2952	2.655	0.117	0.380835
TC	PGS000311	rs7770628	6q25.3	LPA	intronic	G/A		-0.031	0.1267	0.6798	-1.355	2.715	0.5126	0.76850849
TC	PGS000311	rs1084651	6q26	LPA;PLG	intergenic	A/G		-0.023	0.452	-1.043	-2.416	0.3287	0.1362	0.41240093
TC	PGS000311	rs35803101	7p13	NPC1L1	exonic	A/G		-0.14	0.0005126	10.03	-19.33	39.4	0.5031	0.76598972
TC	PGS000311	rs4722551	7p15.2	MIR148A;NFE2L3	intergenic	G/A		0.027	0.02798	3.815	-0.3364	7.967	0.07174	0.27593263
TC	PGS000311	rs12670798	7p15.3	DNAH11	intronic	A/G		0.032	0.4603	-0.4102	-1.775	0.9543	0.5557	0.77813974
TC	PGS000311	rs1997243	7p22.3	C7orf50	intronic	G/A		0.027	0.01128	1.271	-5.528	8.07	0.714	0.8704382
TC	PGS000311	rs11761941	7p22.3	GPR146	exonic	A/G		0.028	0.01301	0.3104	-5.996	6.617	0.9232	0.97377
TC	PGS000311	rs4921914	8p22	NAT2;PSD3	intergenic	G/A		-0.034	0.3933	1.199	-0.2048	2.603	0.0942	0.330365
TC	PGS000311	rs1495741	8p22	NAT2;PSD3	intergenic	G/A		-0.034	0.397	1.197	-0.2053	2.599	0.09439	0.330365
TC	PGS000311	rs4841132	8p23.1	LOC157273	ncRNA_exoni	A/G		0.076	0.03069	-1.895	-5.956	2.165	0.3604	0.66200282
TC	PGS000311	rs10102164	8q11.23	SOX17;RP1	intergenic	A/G		0.03	0.2227	0.4196	-1.212	2.051	0.6143	0.81282378
TC	PGS000311	rs2081687	8q12.1	UBXN2B;CYP7A1	intergenic	A/G		-0.033	0.2908	1.439	-0.06744	2.945	0.06124	0.24753975
TC	PGS000311	rs2737229	8q23.3	TRPS1	intronic	C/A		-0.024	0.3773	-0.07015	-1.483	1.343	0.9225	0.97377
TC	PGS000311	rs2954033	8q24.13	TRIB1;LINC00861	intergenic	A/G		-0.046	0.2775	1.05	-0.4745	2.574	0.1772	0.47866058
TC	PGS000311	rs2954038	8q24.13	TRIB1;LINC00861	intergenic	C/A		-0.052	0.2754	0.9433	-0.5886	2.475	0.2275	0.53879127
TC	PGS000311	rs643531	9p22.3	TTC39B	intronic	C/A		0.027	0.0279	-1.187	-5.3	2.927	0.5719	0.78297794
TC	PGS000311	rs77375493	9p24.1	JAK2	exonic	A/C		-0.32	0.0001025	14.69	-50.76	80.15	0.66	0.84094932
TC	PGS000311	rs3780181	9p24.2	VLDLR	intronic	G/A		-0.03	0.1093	-2.088	-4.28	0.1038	0.06194	0.24753975
TC	PGS000311	rs2230808	9q31.1	ABCA1	exonic	A/G		0.017	0.4522	-1.259	-2.615	0.09744	0.06895	0.2704003
TC	PGS000311	rs3890182	9q31.1	ABCA1	intronic	A/G		-0.057	0.06848	-1.483	-4.206	1.24	0.2858	0.60795342
TC	PGS000311	rs687621	9q34.2	ABO	intronic	G/A		0.044	0.4054	1.833	0.4386	3.227	0.01001	0.06914305
TC	PGS000311	rs10904908	10p13	VIM-AS1	ncRNA_intro	A/G		0.017	0.2994	-0.4633	-1.954	1.027	0.5424	0.77813974
TC	PGS000311	rs970548	10q11.22	MARCHF8	intronic	C/A		0.02	0.08171	0.4405	-2.041	2.922	0.7279	0.87469215
TC	PGS000311	rs2068888	10q23.33	CYP26A1;MYOF	intergenic	G/A		-0.018	0.1926	-0.2915	-2.034	1.451	0.7431	0.87719891
TC	PGS000311	kgp9182400	10q24.31	ERLIN1	exonic	G/A		0.014	0.07197	0.337	-2.281	2.955	0.8008	0.89728193
TC	PGS000311	rs2255141	10q25.2	GPAM	intronic	A/G		-0.028	0.1791	1.349	-0.4707	3.168	0.1463	0.43095611
TC	PGS000311	rs1891110	10q26.13	FAM24B	exonic	A/G		0.019	0.4605	0.7971	-0.5716	2.166	0.2538	0.57351073
TC	PGS000311	rs10838738	11p11.2	MTCH2	intronic	G/A		-0.016	0.2232	-0.2849	-1.943	1.374	0.7363	0.87469215
TC	PGS000311	rs10128711	11p15.1	SPTY2D1	intronic	A/G		0.024	0.4934	0.6881	-0.7118	2.088	0.3354	0.64391559

TC	PGS000311	exm2264444	11p15.1	SPTY2D1	intronic	C/A	0.025	0.4859	0.5815	-0.8075	1.971	0.4119	0.70426364
TC	PGS000311	rs174546	11q12.2	FADS1	UTR3	G/A	-0.047	0.3075	1.044	-0.4618	2.55	0.1743	0.47441625
TC	PGS000311	rs174547	11q12.2	FADS1	intronic	A/G	-0.047	0.3076	1.047	-0.4576	2.552	0.1727	0.47441625
TC	PGS000311	rs174550	11q12.2	FADS1	intronic	A/G	-0.047	0.3076	1.047	-0.4576	2.552	0.1727	0.47441625
TC	PGS000311	rs10047462	11q23.3	APOA1-AS	ncRNA_intro	C/A	-0.044	0.4824	1.749	0.3884	3.11	0.01179	0.07599297
TC	PGS000311	exm2264455	11q23.3	SIK3	intronic	C/A	-0.036	0.2457	-2.208	-3.8	-0.6167	0.006566	0.05277119
TC	PGS000311	rs11820589	11q23.3	BUD13	exonic	A/G	0.072	0.008633	4.095	-3.438	11.63	0.2867	0.60795342
TC	PGS000311	rs7941030	11q24.1	MIR100HG;UBASH3B	intergenic	G/A	0.019	0.344	-0.2804	-1.759	1.198	0.7101	0.86730788
TC	PGS000311	rs11220462	11q24.2	ST3GAL4	intronic	A/G	0.029	0.3339	0.6356	-0.8002	2.071	0.3856	0.68774137
TC	PGS000311	rs4883201	12p13.31	PHC1	intronic	G/A	-0.029	0.2937	-0.5999	-2.107	0.9071	0.4353	0.72106947
TC	PGS000311	rs3184504	12q24.12	SH2B3	exonic	A/G	0.033	0.006251	-3.223	-11.87	5.425	0.4651	0.74457125
TC	PGS000311	rs1169288	12q24.31	HNF1A	exonic	C/A	0.037	0.299	0.1975	-1.309	1.704	0.7972	0.89728193
TC	PGS000311	rs1183910	12q24.31	HNF1A	intronic	A/G	0.035	0.2865	1.006	-0.5116	2.523	0.194	0.49531512
TC	PGS000311	rs7310409	12q24.31	HNF1A	intronic	A/G	-0.03	0.3142	0.615	-0.8633	2.093	0.4149	0.70426364
TC	PGS000311	rs11057830	12q24.31	SCARB1	intronic	A/G	0.024	0.07821	0.5368	-2.023	3.097	0.6811	0.85226336
TC	PGS000311	rs4942486	13q13.1	BRCA2	intronic	A/G	-0.019	0.488	1.766	0.4054	3.126	0.01099	0.07299091
TC	PGS000311	rs8017377	14q12	NYNRIN	exonic	A/G	0.022	0.05912	0.8169	-2.093	3.727	0.5822	0.78960875
TC	PGS000311	rs8014204	14q24.3	PROX2	intronic	A/G	0.015	0.4263	0.2863	-1.101	1.674	0.6859	0.85226336
TC	PGS000311	rs1800588	15q21.3	LIPC	upstream	A/G	0.056	0.4015	1.743	0.3494	3.136	0.01426	0.08675944
TC	PGS000311	rs113298164	15q21.3	LIPC	exonic	A/G	0.13	0.0001025	38.89	-26.61	104.4	0.2446	0.5646617
TC	PGS000311	rs247616	16q13	HERPUD1;CETP	intergenic	A/G	0.044	0.1807	1.919	0.1405	3.698	0.03451	0.17149626
TC	PGS000311	rs3764261	16q13	HERPUD1;CETP	intergenic	A/C	0.043	0.1807	1.857	0.07409	3.64	0.04127	0.19328612
TC	PGS000311	rs9939224	16q13	CETP	intronic	A/C	0.04	0.1386	-2.023	-3.981	-0.06526	0.04289	0.19802404
TC	PGS000311	rs9989419	16q13	HERPUD1;CETP	intergenic	A/G	0.027	0.2533	-0.82	-2.4	0.76	0.3091	0.62686636
TC	PGS000311	rs173539	16q13	HERPUD1;CETP	intergenic	A/G	0.042	0.2252	1.261	-0.379	2.901	0.1319	0.40503255
TC	PGS000311	rs5882	16q13	CETP	exonic	G/A	-0.025	0.4235	0.2327	-1.161	1.627	0.7436	0.87719891
TC	PGS000311	rs8060686	16q22.1	EDC4	exonic	G/A	0.022	0.08824	0.9886	-1.447	3.424	0.4263	0.70977315
TC	PGS000311	rs16942887	16q22.1	PSKH1	intronic	A/G	0.024	0.05076	-0.4923	-3.617	2.632	0.7575	0.88557038
TC	PGS000311	rs34832584	16q22.2	PMFBP1	exonic	T/G	0.02	0.03115	-0.694	-4.709	3.321	0.7348	0.87469215
TC	PGS000311	rs314253	17p13.1	DLG4	UTR3	G/A	-0.02	0.4013	-0.1979	-1.578	1.183	0.7787	0.89314024
TC	PGS000311	rs1801689	17q24.2	APOH	exonic	C/A	0.069	0.0004099	2.238	-30.55	35.02	0.8936	0.95995644
TC	PGS000311	exm1349142	17q24.2	ABCA6	exonic	G/A	0.15	0.0001025	9.835	-55.68	75.35	0.7686	0.89314024
TC	PGS000311	rs2125345	17q25.1	UNK	intronic	G/A	-0.018	0.4242	0.565	-0.8114	1.941	0.4211	0.70836202
TC	PGS000311	rs4129767	17q25.3	PGS1	intronic	A/G	0.018	0.4188	0.8325	-0.5462	2.211	0.2367	0.5523
TC	PGS000311	rs77960347	18q21.1	LIPG	exonic	G/A	0.18	0.0001026	18.88	-46.68	84.44	0.5725	0.78297794
TC	PGS000311	rs7241918	18q21.1	LIPG;ACAA2	intergenic	C/A	0.048	0.1563	0.03154	-1.851	1.914	0.9738	0.99463427
TC	PGS000311	rs58542926	19p13.11	TM6SF2	exonic	A/G	-0.13	0.1166	-2.59	-4.737	-0.4435	0.01808	0.10603676
TC	PGS000311	rs874628	19p13.11	MPV17L2	exonic	G/A	0.015	0.06285	-2.024	-4.832	0.7848	0.1579	0.44887729
TC	PGS000311	rs6511720	19p13.2	LDLR	intronic	A/C	-0.18	0.0205	-3.187	-8.012	1.639	0.1956	0.49531512
TC	PGS000311	rs11669576	19p13.2	LDLR	exonic	A/G	0.05	0.0001025	13.74	-51.73	79.21	0.6809	0.85226336
TC	PGS000311	exm1479047	19q13.32	BCAM	exonic	A/G	-0.17	0.0002063	2.706	-43.55	48.96	0.9087	0.96977656
TC	PGS000311	rs157580	19q13.32	TOMM40	intronic	G/A	0.073	0.4829	-0.4248	-1.808	0.9585	0.5473	0.77813974
TC	PGS000311	rs439401	19q13.32	APOE;APOC1	intergenic	A/G	0.034	0.4959	-0.3436	-1.738	1.051	0.6291	0.82342349
TC	PGS000311	rs2280401	19q13.33	RPS11	intronic	A/G	-0.022	0.2222	-1.811	-3.439	-0.1817	0.02941	0.1507552
TC	PGS000311	rs492602	19q13.33	FUT2	exonic	G/A	0.03	0.02819	0.8277	-3.356	5.011	0.6982	0.86084886
TC	PGS000311	rs2287922	19q13.33	RASIP1	exonic	A/G	0.029	0.03284	-1.359	-5.284	2.566	0.4973	0.76251247
TC	PGS000311	exm2272834	19q13.33	RCN3	intronic	A/G	0.02	0.2731	0.541	-0.9935	2.076	0.4896	0.76100191

TC	PGS000311	rs364585	20p12.1	LINC01722;SPTLC3	intergenic	A/G	0.016	0.4228	-0.332	-1.718	1.054	0.6387	0.8282743
TC	PGS000311	rs2902940	20q12	LINC01370;MAFB	intergenic	G/A	-0.024	0.305	0.9605	-0.527	2.448	0.2057	0.50342368
TC	PGS000311	rs1800961	20q13.12	HNF4A	exonic	A/G	-0.092	0.01138	3.324	-3.177	9.826	0.3163	0.62777835
TC	PGS000311	rs181362	22q11.21	UBE2L3	intronic	G/A	-0.02	0.4242	1.089	-0.2973	2.475	0.1237	0.39091602
TC	PGS000311	22:30776095	22q12.2	RNF215	exonic	A/G	-0.018	0.1586	-0.7092	-2.576	1.158	0.4566	0.7394194
TC	PGS000311	rs17738527	22q12.2	SEC14L4	exonic	T/C	-0.017	0.03115	0.9917	-2.939	4.923	0.621	0.81776121
TC	PGS000311	rs138777	22q12.3	TOM1	intronic	G/A	-0.019	0.4991	1.159	-0.2129	2.531	0.09783	0.33883
TC	PGS000311	22:46627603	22q13.31	PPARA	intronic	A/G	0.025	0.004202	-1.453	-12.32	9.414	0.7933	0.89659427
TG	PGS000312	rs1260326	2p23.3	GCKR	exonic	A/G	-0.12	0.3761	6.632	3.927	9.338	1.603E-06	4.7345E-05
TG	PGS000312	rs1260333	2p23.3	GCKR;C2orf16	intergenic	A/G	-0.095	0.3744	6.219	3.348	9.091	0.00002238	0.0004704
TG	PGS000312	rs301	8p21.3	LPL	intronic	G/A	-0.12	0.223	-7.186	-10.38	-3.996	0.00001033	0.00023948
TG	PGS000312	rs326	8p21.3	LPL	intronic	G/A	-0.11	0.225	-7.278	-10.45	-4.104	7.154E-06	0.00018619
TG	PGS000312	rs13702	8p21.3	LPL	UTR3	G/A	-0.12	0.2219	-7.516	-10.7	-4.328	3.927E-06	0.00011124
TG	PGS000312	rs10096633	8p21.3	LPL;SLC18A1	intergenic	A/G	-0.15	0.1278	-8.238	-12.22	-4.255	0.00005123	0.00098033
TG	PGS000312	rs7350481	11q23.3	LINC02702;BUD13	intergenic	A/G	-0.23	0.247	9.85	6.823	12.88	1.987E-10	1.0796E-08
TG	PGS000312	rs11820589	11q23.3	BUD13	exonic	A/G	0.19	0.008633	28.35	13.82	42.89	0.000134	0.00199591
TG	PGS000312	rs10790162	11q23.3	BUD13	intronic	A/G	-0.26	0.2204	7.908	4.731	11.08	1.102E-06	0.0000341
TG	PGS000312	rs2075291	11q23.3	APOA5	exonic	A/C	0.4	0.03743	26	19.05	32.96	2.746E-13	2.2378E-11
TG	PGS000312	rs10047462	11q23.3	APOA1-AS	ncRNA_intro	C/A	-0.11	0.4824	5.223	2.595	7.851	0.00009969	0.00158304
TG	PGS000312	rs7412	19q13.32	APOE	exonic	A/G	0.12	0.08845	7.956	3.215	12.7	0.001012	0.0119784
TG	PGS000312	rs445925	19q13.32	APOE;APOC1	intergenic	A/G	0.12	0.1098	6.88	2.65	11.11	0.001443	0.01619643
TG	PGS000312	rs7679	20q13.12	PCIF1	UTR3	G/A	0.053	0.06387	10.84	5.477	16.21	0.00007588	0.00126695
TG	PGS000312	rs10889353	1p31.3	DOCK7	intronic	C/A	-0.077	0.2645	-2.042	-5.051	0.9677	0.1837	0.49101741
TG	PGS000312	rs4660293	1p34.3	PABPC4	intronic	G/A	0.024	0.1152	1.514	-2.675	5.703	0.4787	0.75273841
TG	PGS000312	rs12748152	1p36.11	PIGV;ZDHHC18	intergenic	A/G	0.031	0.02289	5.764	-3.078	14.61	0.2015	0.49951255
TG	PGS000312	rs1077514	1p36.12	ASAP3	intronic	G/A	0.019	0.4271	-0.5972	-3.259	2.065	0.6601	0.84094932
TG	PGS000312	rs1011731	1q24.3	DNM3	intronic	G/A	-0.015	0.1851	1.787	-1.632	5.206	0.3056	0.6233325
TG	PGS000312	rs2785980	1q41	LYPLAL1-AS1	ncRNA_intro	G/A	-0.016	0.2003	-1.301	-4.663	2.06	0.448	0.73025925
TG	PGS000312	rs4846914	1q42.13	GALNT2	intronic	A/G	-0.039	0.2439	-3.157	-6.262	-0.0522	0.04633	0.20765571
TG	PGS000312	rs10489615	1q42.13	GALNT2	intronic	G/A	-0.039	0.2668	-4.125	-7.146	-1.105	0.007455	0.05777625
TG	PGS000312	rs4245791	2p21	ABCG8	intronic	G/A	-0.019	0.02696	-1.566	-9.76	6.628	0.708	0.86636842
TG	PGS000312	rs1049817	2p23.3	GTF3C2	exonic	G/A	-0.056	0.2179	0.08024	-3.121	3.281	0.9608	0.99110379
TG	PGS000312	rs541041	2p24.1	APOB;TDRD15	intergenic	G/A	0.018	0.01989	12	2.513	21.48	0.0132	0.08262692
TG	PGS000312	rs676210	2p24.1	APOB	exonic	G/A	-0.071	0.3095	-0.003651	-2.873	2.866	0.998	0.998
TG	PGS000312	rs533617	2p24.1	APOB	exonic	G/A	-0.098	0.003382	-6.841	-29.73	16.05	0.5581	0.77813974
TG	PGS000312	rs1367117	2p24.1	APOB	exonic	A/G	0.023	0.1396	0.4966	-3.33	4.324	0.7992	0.89728193
TG	PGS000312	rs13389219	2q24.3	COBLL1	intronic	A/G	-0.037	0.109	-4.289	-8.518	-0.06015	0.04689	0.20765571
TG	PGS000312	rs12328675	2q24.3	COBLL1	UTR3	G/A	-0.045	0.0159	-1.564	-12.17	9.043	0.7726	0.89314024
TG	PGS000312	rs7607980	2q24.3	COBLL1	exonic	G/A	-0.045	0.01189	-6.988	-19.12	5.144	0.259	0.5789887
TG	PGS000312	rs1344642	2q35	STK36	exonic	A/G	-0.015	0.13	3.566	-0.3646	7.497	0.07544	0.28553163
TG	PGS000312	rs2943641	2q36.3	LOC646736;MIR5702	intergenic	A/G	0.033	0.1178	-0.4785	-4.55	3.592	0.8178	0.905975
TG	PGS000312	rs9311651	3p14.3	DNAH12	exonic	G/A	-0.021	0.009941	-4.773	-18.09	8.542	0.4823	0.75475313
TG	PGS000312	rs13326165	3p21.1	STAB1	intronic	A/G	0.02	0.05456	-5.434	-11.26	0.3916	0.06759	0.26797527
TG	PGS000312	rs645040	3q22.3	MSL2;PCCB	intergenic	C/A	0.023	0.1654	-0.3269	-3.89	3.236	0.8573	0.9301705
TG	PGS000312	exm384446	4p16.3	HGFAC	exonic	A/C	0.035	0.317	1.914	-0.9219	4.749	0.186	0.49101741
TG	PGS000312	rs16844401	4p16.3	HGFAC	exonic	A/G	0.03	0.07307	2.77	-2.37	7.91	0.2909	0.60947788
TG	PGS000312	rs6831256	4p16.3	DOK7	intronic	G/A	0.021	0.4068	0.8419	-1.861	3.545	0.5415	0.77813974

TG	PGS000312	rs442177	4q22.1	AFF1	intronic	C/A	0.031	0.4135	-3.313	-5.99	-0.6366	0.0153	0.09054818
TG	PGS000312	rs13107325	4q24	SLC39A8	exonic	A/G	0.034	0.000415	20.7	-53.04	94.44	0.5822	0.78960875
TG	PGS000312	rs6054	4q31.3	FGB	exonic	A/G	0.14	0.001128	-16.58	-58.79	25.63	0.4414	0.7274719
TG	PGS000312	rs459193	5q11.2	C5orf67	downstream	G/A	0.023	0.4378	2.455	-0.2233	5.133	0.07248	0.27593263
TG	PGS000312	rs9686661	5q11.2	C5orf67	ncRNA_intro	A/G	0.042	0.1961	0.576	-2.74	3.892	0.7335	0.87469215
TG	PGS000312	exm-rs4976033	5q13.1	PIK3R1;LINC02198	intergenic	G/A	0.018	0.4934	2.292	-0.3269	4.911	0.08635	0.31404385
TG	PGS000312	kgp10178030	5q31.1	FNIP1	exonic	A/G	-0.028	0.2568	0.2618	-2.717	3.241	0.8632	0.93346047
TG	PGS000312	rs6882076	5q33.3	TIMD4	upstream	A/G	0.038	0.2888	-0.4126	-3.373	2.548	0.7848	0.89314024
TG	PGS000312	rs6905288	6p21.1	VEGFA;LINC02537	intergenic	G/A	0.033	0.2989	-3.362	-6.256	-0.468	0.02284	0.124775
TG	PGS000312	rs998584	6p21.1	VEGFA;LINC02537	intergenic	C/A	0.034	0.4311	-2.114	-4.798	0.57	0.1227	0.39091602
TG	PGS000312	rs9472138	6p21.1	LINC02537	downstream	A/G	-0.02	0.1662	-0.9629	-4.511	2.585	0.5948	0.79640779
TG	PGS000312	rs9271366	6p21.32	HLA-DRB1;HLA-DQA1	intergenic	G/A	0.024	0.1898	2.655	-0.7686	6.078	0.1286	0.40249327
TG	PGS000312	rs1057373	6p21.32	PSMB8-AS1	ncRNA_exoni	A/C	0.03	0.1316	-2.779	-6.946	1.388	0.1913	0.49531512
TG	PGS000312	rs3130564	6p21.33	PSORS1C1	intronic	A/G	-0.033	0.000615	-7.838	-59.62	43.94	0.7667	0.89314024
TG	PGS000312	rs3873379	6p21.33	LINC02571	ncRNA_intro	G/A	0.028	0.2341	1.626	-1.828	5.08	0.3563	0.65895256
TG	PGS000312	exm-rs3128982	6p21.33	LINC01149;HCP5	intergenic	G/A	-0.027	0.02624	-3.227	-11.44	4.984	0.4411	0.7274719
TG	PGS000312	rs2844480	6p21.33	NCR3;AIF1	intergenic	A/G	0.023	0.2134	-0.471	-3.895	2.953	0.7875	0.89314024
TG	PGS000312	rs643381	6q24.1	LINC01625;FILNC1	intergenic	A/C	-0.023	0.3598	-2.394	-5.148	0.3607	0.08858	0.32036433
TG	PGS000312	rs12208357	6q25.3	SLC22A1	exonic	A/G	0.032	0.001051	35.46	-4.796	75.71	0.08434	0.30856669
TG	PGS000312	rs1564348	6q25.3	SLC22A1	intronic	G/A	0.02	0.01866	-3.355	-13.16	6.451	0.5025	0.76598972
TG	PGS000312	rs7758229	6q25.3	SLC22A3	intronic	A/C	0.018	0.3176	0.8783	-1.971	3.728	0.5458	0.77813974
TG	PGS000312	rs4722551	7p15.2	MIR148A;NFE2L3	intergenic	G/A	-0.026	0.02798	5.177	-2.832	13.19	0.2053	0.50342368
TG	PGS000312	rs4410790	7p21.1	AGR3;AHR	intergenic	G/A	0.015	0.4706	2.711	0.07345	5.348	0.04401	0.20144833
TG	PGS000312	rs2240466	7q11.23	BAZ1B	UTR3	A/G	-0.12	0.1069	-3.159	-7.44	1.121	0.1481	0.43429324
TG	PGS000312	rs1178979	7q11.23	BAZ1B	UTR3	G/A	-0.096	0.1076	-1.963	-6.236	2.31	0.368	0.66804568
TG	PGS000312	kgp4559893	7q31.1	GPR85	downstream	G/A	0.041	0.07778	1.77	-3.273	6.814	0.4915	0.761825
TG	PGS000312	rs38855	7q31.2	MET	intronic	A/G	-0.014	0.4435	0.4722	-2.192	3.136	0.7283	0.87469215
TG	PGS000312	rs4731702	7q32.2	KLF14;MIR29A	intergenic	A/G	-0.027	0.3215	-1.578	-4.408	1.252	0.2746	0.59322914
TG	PGS000312	rs972283	7q32.2	KLF14;MIR29A	intergenic	A/G	0.027	0.3289	-2.744	-5.565	0.07654	0.05661	0.23535516
TG	PGS000312	rs1801177	8p21.3	LPL	exonic	A/G	0.17	0.0008198	30.11	-14.67	74.88	0.1876	0.49125663
TG	PGS000312	rs268	8p21.3	LPL	exonic	G/A	0.24	0.0007181	14.84	-54.65	84.32	0.6756	0.84906486
TG	PGS000312	rs4921914	8p22	NAT2;PSD3	intergenic	G/A	-0.035	0.3933	-0.5943	-3.308	2.12	0.6678	0.84415107
TG	PGS000312	rs1495741	8p22	NAT2;PSD3	intergenic	G/A	-0.035	0.397	-0.555	-3.267	2.157	0.6883	0.853492
TG	PGS000312	rs4841132	8p23.1	LOC157273	ncRNA_exoni	A/G	-0.035	0.03069	8.891	1.059	16.72	0.02614	0.137235
TG	PGS000312	rs3947	8p23.1	CTSB	UTR3	A/G	0.024	0.01312	3.58	-8.125	15.29	0.5489	0.77813974
TG	PGS000312	rs2081687	8q12.1	UBXN2B;CYP7A1	intergenic	A/G	-0.019	0.2908	1.214	-1.697	4.125	0.4137	0.70426364
TG	PGS000312	rs2954033	8q24.13	TRIB1;LINC00861	intergenic	A/G	-0.082	0.2775	2.149	-0.7961	5.094	0.1528	0.44210133
TG	PGS000312	rs2954038	8q24.13	TRIB1;LINC00861	intergenic	C/A	-0.087	0.2754	1.803	-1.156	4.762	0.2325	0.54445144
TG	PGS000312	rs1883025	9q31.1	ABCA1	intronic	A/G	-0.022	0.2555	-0.6477	-3.68	2.385	0.6755	0.84906486
TG	PGS000312	rs1832007	10p15.1	AKR1C4	intronic	G/A	-0.033	0.09465	-2.65	-7.161	1.861	0.2496	0.57059579
TG	PGS000312	rs12355784	10q21.3	JMJD1C	intronic	A/C	-0.03	0.2788	-1.081	-4.032	1.871	0.473	0.74920438
TG	PGS000312	exm2259631	10q22.1	MCU	intronic	G/A	0.042	0.311	1.573	-1.286	4.433	0.2809	0.60351782
TG	PGS000312	rs2068888	10q23.33	CYP26A1;MYOF	intergenic	G/A	-0.032	0.1926	2.828	-0.5317	6.189	0.09905	0.33937658
TG	PGS000312	rs2255141	10q25.2	GPAM	intronic	A/G	0.019	0.1791	-1.067	-4.584	2.45	0.5522	0.77813974
TG	PGS000312	rs7940646	11p15.4	IRAG1	intronic	A/G	0.016	0.0565	-2.095	-7.935	3.745	0.4821	0.75475313
TG	PGS000312	rs174546	11q12.2	FADS1	UTR3	G/A	0.052	0.3075	-2.165	-5.074	0.7442	0.1447	0.42818045
TG	PGS000312	rs174547	11q12.2	FADS1	intronic	A/G	0.052	0.3076	-2.216	-5.123	0.6917	0.1353	0.41159019

TG	PGS000312	rs174550	11q12.2	FADS1	intronic	A/G	0.052	0.3076	-2.216	-5.123	0.6917	0.1353	0.41159019
TG	PGS000312	rs12801636	11q13.1	PCNX3	intronic	A/G	-0.018	0.3553	-1.052	-3.844	1.74	0.4602	0.74284901
TG	PGS000312	exm2264455	11q23.3	SIK3	intronic	C/A	-0.058	0.2457	-3.955	-7.031	-0.8793	0.01176	0.07599297
TG	PGS000312	exm957687	11q23.3	APOA5	exonic	A/G	0.085	0.08781	-1.089	-5.808	3.63	0.6511	0.83768004
TG	PGS000312	rs76353203	11q23.3	APOC3	exonic	A/G	-1.22	0.0001025	-24.51	-151	102	0.7041	0.86484736
TG	PGS000312	rs4149056	12p12.1	SLCO1B1	exonic	G/A	0.029	0.1138	2.604	-1.58	6.787	0.2226	0.53081538
TG	PGS000312	rs1106766	12q13.3	R3HDM2	intronic	A/G	-0.03	0.06723	-2.311	-7.597	2.975	0.3916	0.69274891
TG	PGS000312	rs7157785	14q23.2	SGPP1;SYNE2	intergenic	A/C	0.023	0.1096	2.137	-2.218	6.491	0.3363	0.64391559
TG	PGS000312	exm1149166	15q15.1	BAHD1	exonic	C/A	-0.017	0.2772	-1.527	-4.481	1.428	0.3113	0.62777835
TG	PGS000312	rs1800588	15q21.3	LIPC	upstream	A/G	0.047	0.4015	2.911	0.218	5.604	0.03418	0.17116292
TG	PGS000312	rs113298164	15q21.3	LIPC	exonic	A/G	0.12	0.0001025	167.3	40.84	293.8	0.009549	0.06756955
TG	PGS000312	rs10468017	15q21.3	AQP9;LIPC	intergenic	A/G	0.034	0.1941	2.764	-0.6073	6.135	0.1081	0.35805
TG	PGS000312	rs1532085	15q21.3	AQP9;LIPC	intergenic	A/G	-0.031	0.4751	1.625	-1.033	4.283	0.2309	0.54265668
TG	PGS000312	rs7200543	16p13.11	PDXDC1	exonic	G/A	0.024	0.3765	-1.359	-4.067	1.349	0.3255	0.63696647
TG	PGS000312	rs1421085	16q12.2	FTO	intronic	G/A	0.019	0.243	1.824	-1.313	4.961	0.2546	0.57351073
TG	PGS000312	rs9989419	16q13	HERPUD1;CETP	intergenic	A/G	-0.019	0.2533	-1.527	-4.58	1.525	0.3268	0.63696647
TG	PGS000312	rs173539	16q13	HERPUD1;CETP	intergenic	A/G	-0.034	0.2252	-1.362	-4.531	1.808	0.3998	0.70056676
TG	PGS000312	rs247616	16q13	HERPUD1;CETP	intergenic	A/G	-0.036	0.1807	-1.448	-4.886	1.99	0.4091	0.70426364
TG	PGS000312	rs3764261	16q13	HERPUD1;CETP	intergenic	A/C	-0.036	0.1807	-1.535	-4.979	1.909	0.3823	0.68533846
TG	PGS000312	rs9939224	16q13	CETP	intronic	A/C	-0.034	0.1386	1.84	-1.945	5.625	0.3408	0.6462689
TG	PGS000312	rs2925979	16q23.2	CMIP	intronic	A/G	-0.029	0.3874	-0.6558	-3.352	2.04	0.6336	0.8262492
TG	PGS000312	rs7946	17p11.2	PEMT	exonic	A/G	-0.016	0.1912	1.908	-1.461	5.277	0.267	0.58721959
TG	PGS000312	rs1801689	17q24.2	APOH	exonic	C/A	-0.047	0.0004099	-20.24	-83.58	43.1	0.5312	0.77813974
TG	PGS000312	exm2268053	17q24.2	BPTF	intronic	G/A	-0.02	0.1933	-0.9581	-4.311	2.395	0.5755	0.78543082
TG	PGS000312	rs2292642	17q25.3	PGS1	exonic	G/A	-0.02	0.4665	1.347	-1.272	3.965	0.3135	0.62777835
TG	PGS000312	rs4129767	17q25.3	PGS1	intronic	A/G	-0.017	0.4188	-1.266	-3.932	1.4	0.3519	0.654534
TG	PGS000312	rs489693	18q21.32	PMAIP1;MC4R	intergenic	A/C	0.015	0.1902	3.828	0.4322	7.225	0.0272	0.1416576
TG	PGS000312	rs12970134	18q21.32	PMAIP1;MC4R	intergenic	A/G	0.017	0.1454	5.098	1.302	8.895	0.00852	0.06229347
TG	PGS000312	rs58542926	19p13.11	TM6SF2	exonic	A/G	-0.12	0.1166	-5.15	-9.299	-1.002	0.015	0.08958716
TG	PGS000312	rs891088	19p13.2	INSR	intronic	G/A	-0.017	0.2487	-2.951	-5.987	0.08418	0.05676	0.23535516
TG	PGS000312	rs7248104	19p13.2	INSR	intronic	A/G	-0.02	0.2943	-0.5179	-3.413	2.377	0.7258	0.87469215
TG	PGS000312	rs731839	19q13.11	PEPD	intronic	A/G	-0.015	0.431	-0.8269	-3.49	1.836	0.5428	0.77813974
TG	PGS000312	rs8182584	19q13.11	PEPD	intronic	C/A	-0.016	0.3506	-0.8265	-3.583	1.93	0.5568	0.77813974
TG	PGS000312	rs439401	19q13.32	APOE;APOC1	intergenic	A/G	0.075	0.4959	-3.096	-5.79	-0.4021	0.02434	0.13095322
TG	PGS000312	exm1479047	19q13.32	BCAM	exonic	A/G	0.073	0.0002063	1.365	-88.25	90.98	0.9762	0.99463427
TG	PGS000312	rs157580	19q13.32	TOMM40	intronic	G/A	0.047	0.4829	-2.641	-5.313	0.03042	0.05273	0.22436098
TG	PGS000312	rs769449	19q13.32	APOE	intronic	A/G	0.066	0.1041	2.542	-1.787	6.871	0.2498	0.57059579
TG	PGS000312	rs492602	19q13.33	FUT2	exonic	G/A	0.018	0.02819	2.957	-5.099	11.01	0.4719	0.74920438
TG	PGS000312	rs2287922	19q13.33	RASIP1	exonic	A/G	0.019	0.03284	2.964	-4.602	10.53	0.4427	0.72777197
TG	PGS000312	rs2280401	19q13.33	RPS11	intronic	A/G	-0.02	0.2222	-1.358	-4.505	1.79	0.3979	0.70044081
TG	PGS000312	exm2268318	20q13.33	TCEA2	intronic	G/A	-0.018	0.4137	-1.407	-4.103	1.289	0.3064	0.6233325
TG	PGS000312	rs738322	22q13.1	PLA2G6	intronic	G/A	-0.02	0.4233	-2.22	-4.933	0.4937	0.1089	0.35805

Table S12 Significant SNPs associated T2D, CVD, and cardiometabolic traits (TG, TC, HDL-C, LDL-C, FBS, and HbA1c) from the GWAS

Trait	SNP ID	Cytoband	Gene	Genic region	Alleles	MAF	Beta/OR	p-value
T2D	rs58078949	2p25.3	TMEM18	intronic	A/G	0.0365854	2.16898	9.18E-06
T2D	rs1320333	2p25.3	TMEM18;LINC01115	intergenic	G/A	0.0308781	2.327	2.70E-06
T2D	rs35181861	2p25.3	TMEM18;LINC01115	intergenic	A/G	0.033511	2.3971	6.35E-07
T2D	rs2867116	2p25.3	TMEM18;LINC01115	intergenic	C/A	0.0334085	2.25795	5.03E-06
T2D	rs73130856	3p12.1	LINC00971	ncRNA_intro	T/C	0.0183439	2.90065	1.46E-06
T2D	rs114715418	3p12.1	LINC00971	ncRNA_intro	G/T	0.0183439	2.90065	1.46E-06
T2D	rs73130858	3p12.1	LINC00971	ncRNA_intro	G/A	0.0183477	2.90008	1.46E-06
T2D	rs77547468	3p12.1	LINC00971	ncRNA_intro	G/T	0.0183439	2.90065	1.46E-06
T2D	rs73130863	3p12.1	LINC00971	ncRNA_intro	T/C	0.0182414	2.91084	1.36E-06
T2D	rs73130864	3p12.1	LINC00971	ncRNA_intro	C/T	0.0183439	2.90065	1.46E-06
T2D	rs73130865	3p12.1	LINC00971	ncRNA_intro	C/A	0.0183439	2.90065	1.46E-06
T2D	rs10451955	3p12.1	LINC00971	ncRNA_intro	G/A	0.018139	2.94469	1.08E-06
T2D	rs73130872	3p12.1	LINC00971	ncRNA_intro	A/G	0.018139	2.94469	1.08E-06
T2D	rs73130876	3p12.1	LINC00971	ncRNA_intro	C/T	0.018139	2.94469	1.08E-06
T2D	rs115407725	3p12.1	LINC00971	ncRNA_intro	T/C	0.018139	2.94469	1.08E-06
T2D	rs73130884	3p12.1	LINC00971	ncRNA_intro	T/C	0.018139	2.94469	1.08E-06
T2D	rs73130886	3p12.1	LINC00971	ncRNA_intro	A/G	0.018139	2.94469	1.08E-06
T2D	rs73130888	3p12.1	LINC00971	ncRNA_intro	C/A	0.018139	2.94469	1.08E-06
T2D	rs73132954	3p12.1	LINC00971	ncRNA_intro	A/G	0.018139	2.94469	1.08E-06
T2D	rs73132966	3p12.1	LINC00971	ncRNA_intro	A/G	0.018139	2.94469	1.08E-06
T2D	rs73132968	3p12.1	LINC00971	ncRNA_intro	C/T	0.018139	2.94469	1.08E-06
T2D	rs73132977	3p12.1	LINC00971	ncRNA_intro	T/G	0.018139	2.94469	1.08E-06
T2D	rs73132979	3p12.1	LINC00971	ncRNA_intro	G/A	0.018139	2.94469	1.08E-06
T2D	rs73132980	3p12.1	LINC00971	ncRNA_intro	C/A	0.018139	2.94469	1.08E-06
T2D	rs115918885	3p12.1	LINC00971	ncRNA_intro	G/T	0.018139	2.94469	1.08E-06
T2D	rs115228467	3p12.1	LINC00971	ncRNA_intro	C/A	0.018139	2.94469	1.08E-06
T2D	rs115617478	3p12.1	LINC00971	ncRNA_intro	C/A	0.0180365	2.95522	1.00E-06
T2D	rs145862500	3p12.1	LINC00971	ncRNA_intro	G/A	0.018139	2.94469	1.08E-06
T2D	rs73134809	3p12.1	LINC00971	ncRNA_intro	C/T	0.018139	2.94469	1.08E-06
T2D	rs74703095	3p12.1	LINC00971	ncRNA_intro	G/A	0.0180365	2.95522	1.00E-06
T2D	rs73134822	3p12.1	LINC00971	ncRNA_intro	G/T	0.0180365	2.95522	1.00E-06
T2D	rs73134824	3p12.1	LINC00971	ncRNA_intro	C/T	0.018139	2.94469	1.08E-06
T2D	rs73134826	3p12.1	LINC00971	ncRNA_intro	A/C	0.018139	2.94469	1.08E-06
T2D	rs142758670	3p12.1	LINC00971	ncRNA_intro	T/C	0.0180365	2.95522	1.00E-06
T2D	rs73134840	3p12.1	LINC00971	ncRNA_intro	C/T	0.018139	2.94469	1.08E-06
T2D	rs74528939	3p12.1	LINC00971	ncRNA_intro	T/C	0.018139	2.94469	1.08E-06
T2D	rs117853703	3p12.1	LINC00971	ncRNA_intro	C/A	0.018139	2.94469	1.08E-06
T2D	rs73134860	3p12.1	LINC00971	ncRNA_intro	T/C	0.018139	2.94469	1.08E-06
T2D	rs73126057	3p12.1	LINC00971	ncRNA_intro	A/G	0.0187538	2.80863	2.86E-06
T2D	rs4599251	3q21.3	PLXNA1;C3orf56	intergenic	G/A	0.296535	0.591601	8.50E-07
T2D	rs9872573	3q22.3	LINC01210;CLDN18	intergenic	G/A	0.0593359	1.95434	4.27E-06
T2D	rs115555320	3q25.33	KPNA4;ARL14	intergenic	A/G	0.0339209	2.2517	8.05E-06
T2D	rs55773251	4q35.1	CLDN24;CDKN2AIP	intergenic	G/A	0.398786	1.51201	1.82E-06
T2D	rs2686485	7p15.3	RAPGEF5;STEAP1B	intergenic	A/G	0.0414104	2.10454	8.80E-06
T2D	rs2390634	7p15.3	RAPGEF5;STEAP1B	intergenic	T/C	0.0423242	2.09603	7.46E-06
T2D	rs4722136	7p15.3	RAPGEF5;STEAP1B	intergenic	C/T	0.0423242	2.09603	7.46E-06
T2D	rs75553117	7q21.13	CDK14	intronic	A/G	0.0313782	2.40079	4.10E-06
T2D	rs4870904	8q24.13	MTSS1	intronic	A/G	0.262046	1.50876	8.59E-06
T2D	rs2288663	8q24.13	MTSS1	intronic	G/A	0.260816	1.51662	6.48E-06
T2D	rs2933170	11p14.1	MIR8068;LINC02546	intergenic	A/G	0.219762	1.59585	1.17E-06
T2D	rs191099352	11q23.3	FXYD6;TMPRSS13	intergenic	G/A	0.0124001	3.36664	1.27E-06
T2D	rs144466082	11q23.3	FXYD6;TMPRSS13	intergenic	C/T	0.0124001	3.36664	1.27E-06
T2D	rs185487064	11q23.3	FXYD6;TMPRSS13	intergenic	T/C	0.0124001	3.36664	1.27E-06
T2D	rs17116412	14q21.2	LINC02303;LINC00871	intergenic	G/A	0.0953064	1.79972	3.35E-06
T2D	rs6498908	16q21	CDH8;CDH11	intergenic	G/A	0.288891	1.49231	9.61E-06
T2D	kgp5217289	16q21	CDH8;CDH11	intergenic	A/G	0.27845	1.50719	6.79E-06
T2D	kgp19375422	20p12.2	SNAP25	intronic	G/A	0.0295445	2.38145	6.27E-06
T2D	rs3787287	20p12.2	SNAP25	intronic	C/T	0.028387	2.41283	6.70E-06
CVD	1:80308260	1p31.1	ADGRL4;LINC01781	intergenic	G/A	0.056717	2.67604	9.26E-06
CVD	rs186530350	2p21	SOCS5	intronic	T/C	0.0251076	3.46084	6.52E-06
CVD	rs368983869	2q11.2	CRACDL	intronic	T/G	0.0116827	4.76741	7.64E-06
CVD	rs6749360	2q32.3	MYO1B	intronic	C/T	0.0114778	4.58213	8.57E-06
CVD	rs10172030	2q32.3	MYO1B	intronic	A/C	0.0114801	4.58152	8.57E-06
CVD	rs11898504	2q32.3	MYO1B	intronic	G/A	0.0114778	4.58213	8.57E-06
CVD	rs76562251	4q23	TSPAN5	intronic	A/C	0.0355606	3.00735	9.05E-06
CVD	GSA-rs76601894	4q28.3	LINC02485;LINC00613	intergenic	C/A	0.0131812	4.7642	4.52E-06
CVD	5:55639371	5q11.2	ANKRD55;LINC01948	intergenic	A/G	0.0121976	5.03074	1.26E-06
CVD	rs137914756	5q13.2	LINC02056	ncRNA_intro	T/G	0.0349457	2.89761	9.72E-06

CVD	rs144071442	5q13.2	LINC02056	ncRNA_intro	G/A	0.0349457	2.89761	9.72E-06
CVD	rs144758357	5q13.2	LINC02056	ncRNA_intro	G/A	0.0349457	2.89761	9.72E-06
CVD	rs140951508	5q13.2	LINC02056	ncRNA_intro	G/A	0.0349457	2.89761	9.72E-06
CVD	rs142127744	5q13.2	LINC02056	ncRNA_intro	C/A	0.0349457	2.89761	9.72E-06
CVD	rs147856269	5q13.2	LINC02056	ncRNA_intro	G/A	0.0347023	2.99801	7.10E-06
CVD	rs140593490	5q13.2	LINC02056	ncRNA_intro	G/A	0.0346382	3.00438	6.87E-06
CVD	rs146739317	5q13.2	LINC02056	ncRNA_intro	C/T	0.0346382	3.00438	6.87E-06
CVD	rs145619986	5q13.2	LINC02056;TNPO1	intergenic	T/C	0.0346382	3.00438	6.87E-06
CVD	rs147523281	5q13.2	LINC02056;TNPO1	intergenic	C/T	0.0344333	3.0224	6.12E-06
CVD	rs11758757	6p21.2	MOCS1;LINC00951	intergenic	C/T	0.0197786	4.03864	7.57E-06
CVD	rs117258052	6p21.2	MOCS1;LINC00951	intergenic	C/T	0.0199836	4.00575	8.49E-06
CVD	rs11756006	6p21.2	MOCS1;LINC00951	intergenic	A/G	0.0199836	4.00575	8.49E-06
CVD	rs116254668	6p21.2	MOCS1;LINC00951	intergenic	C/T	0.0199836	4.00575	8.49E-06
CVD	rs75343264	6p21.2	MOCS1;LINC00951	intergenic	G/A	0.0199836	4.00575	8.49E-06
CVD	rs11757716	6p21.2	MOCS1;LINC00951	intergenic	A/G	0.0200861	3.97998	9.25E-06
CVD	9:2044094	9p24.3	SMARCA2	intronic	G/A	0.0300266	3.03623	9.32E-06
CVD	rs12005594	9p24.3	SMARCA2	intronic	G/T	0.028182	3.36127	2.70E-06
CVD	rs142176660	9q21.32	SPATA31D1;RASEF	intergenic	A/G	0.0254568	3.47472	3.17E-06
CVD	rs77286104	9q21.32	SPATA31D1;RASEF	intergenic	G/A	0.025415	3.48051	3.09E-06
CVD	rs80340591	9q21.32	SPATA31D1;RASEF	intergenic	C/T	0.025415	3.48051	3.09E-06
CVD	rs78249385	9q21.32	SPATA31D1;RASEF	intergenic	T/C	0.025415	3.48051	3.09E-06
CVD	rs10115136	9q21.32	SPATA31D1;RASEF	intergenic	T/C	0.0278746	3.56381	5.77E-07
CVD	rs13440407	9q21.32	SPATA31D1;RASEF	intergenic	G/A	0.0268498	3.7091	2.58E-07
CVD	rs13439974	9q21.32	SPATA31D1;RASEF	intergenic	T/C	0.0237754	3.61018	1.23E-06
CVD	rs964395	9q21.32	SPATA31D1;RASEF	intergenic	T/C	0.0237754	3.61018	1.23E-06
CVD	kgp10629268	10p14	TAF3	intronic	G/A	0.0574148	2.63453	4.74E-06
CVD	rs11101383	10q11.22	ARHGAP22	intronic	C/T	0.0362779	3.15835	3.37E-06
CVD	rs12766573	10q11.22	ARHGAP22	UTR3	C/T	0.0269522	4.14567	2.22E-08
CVD	rs7919520	10q11.22	ARHGAP22	intronic	G/A	0.0269522	4.14567	2.22E-08
CVD	rs12800954	11p15.4	AMPD3	intronic	A/G	0.0298217	3.36253	2.18E-06
CVD	rs79761334	13q31.3	GPC5	intronic	A/G	0.0537326	2.65325	8.26E-06
CVD	rs2397362	16q12.2	IRX5;IRX6	intergenic	T/G	0.0250051	3.29117	5.94E-06
CVD	16:55220800	16q12.2	IRX5;IRX6	intergenic	A/G	0.0250103	3.29479	5.82E-06
CVD	rs62034695	16q12.2	IRX5;IRX6	intergenic	G/A	0.0227506	3.38419	7.02E-06
TG	kgp15300624	1q43	RYR2;LOC100130331	intergenic	C/A	0.232924	7.92295	7.71E-07
TG	rs13405305	2p25.3	SNTG2	intronic	C/T	0.0244755	22.0156	5.01E-07
TG	rs1260326	2p23.3	GCKR	exonic	G/A	0.376066	6.63226	1.60E-06
TG	rs780094	2p23.3	GCKR	intronic	G/A	0.359197	6.86548	1.06E-06
TG	rs780093	2p23.3	GCKR	intronic	G/A	0.35864	6.42403	4.58E-06
TG	rs79512070	3q26.32	LINC02015;LINC01014	intergenic	G/A	0.0112544	29.5852	3.47E-06
TG	rs77833848	5q14.3	EDIL3	intronic	G/A	0.0113087	29.7233	3.72E-06
TG	rs180728061	5q14.3	LINC02059;MIR4280	intergenic	C/T	0.0127841	26.9693	8.54E-06
TG	5:166635690	5q34	TENM2	intronic	G/A	0.0170455	23.2057	7.88E-06
TG	rs1766848	6p25.3	HUS1B	exonic	C/A	0.0748961	12.0578	3.81E-06
TG	rs1193738	6p25.3	EXOC2	intronic	C/T	0.0746285	11.8275	5.95E-06
TG	rs1201552	6p25.3	EXOC2	intronic	T/C	0.0746285	11.8275	5.95E-06
TG	rs1193737	6p25.3	EXOC2	intronic	A/C	0.0746285	11.8275	5.95E-06
TG	rs1211242	6p25.3	EXOC2	intronic	C/T	0.0745192	11.9252	5.05E-06
TG	rs1747579	6p25.3	EXOC2	intronic	A/G	0.0745192	11.9252	5.05E-06
TG	rs12527700	6p25.3	EXOC2	intronic	T/C	0.07441	12.0211	4.29E-06
TG	rs12333170	6p25.3	EXOC2	intronic	T/G	0.0826049	12.5065	5.75E-07
TG	rs6909741	6p25.3	EXOC2	intronic	G/A	0.0724432	12.0815	5.02E-06
TG	rs6940659	6p25.3	EXOC2	intronic	T/G	0.08184	12.4716	6.77E-07
TG	rs6927305	6p25.3	EXOC2	intronic	C/T	0.0822771	12.5501	5.47E-07
TG	rs35857639	6p25.3	EXOC2	intronic	C/T	0.0729895	12.0628	4.87E-06
TG	rs67934143	6p25.3	EXOC2	intronic	T/C	0.0729895	12.0628	4.87E-06
TG	rs11757546	6p25.3	EXOC2	intronic	C/T	0.0729895	12.0628	4.87E-06
TG	rs11757548	6p25.3	EXOC2	intronic	C/A	0.0822771	12.5501	5.47E-07
TG	rs11243065	6p25.3	EXOC2	intronic	T/C	0.0822771	12.5501	5.47E-07
TG	rs9504733	6p25.3	EXOC2	intronic	A/G	0.0868663	11.167	4.99E-06
TG	rs71550023	6p25.3	EXOC2	intronic	A/G	0.0868663	11.167	4.99E-06
TG	rs66650217	6p25.3	EXOC2	intronic	G/A	0.0868663	11.167	4.99E-06
TG	rs9504741	6p25.3	EXOC2	intronic	T/C	0.0868663	11.167	4.99E-06
TG	rs12527555	6p25.3	EXOC2	intronic	G/A	0.0868663	11.167	4.99E-06
TG	rs11243068	6p25.3	EXOC2	intronic	A/G	0.0822771	12.5501	5.47E-07
TG	rs4960183	6p25.3	EXOC2	intronic	T/C	0.0866477	11.1481	5.27E-06
TG	rs4959379	6p25.3	EXOC2	intronic	A/G	0.086776	11.1781	4.93E-06
TG	rs4960184	6p25.3	EXOC2	intronic	T/C	0.0868663	11.167	4.99E-06
TG	rs4960187	6p25.3	EXOC2	intronic	G/A	0.0868663	11.167	4.99E-06
TG	rs4960188	6p25.3	EXOC2	intronic	T/G	0.0868663	11.167	4.99E-06

TG	rs9502432	6p25.3	EXOC2	intronic	T/C	0.0868663	11.167	4.99E-06
TG	rs9504749	6p25.3	EXOC2	intronic	C/T	0.0868663	11.167	4.99E-06
TG	rs7768391	6p25.3	EXOC2	intronic	A/C	0.0868663	11.167	4.99E-06
TG	rs7768558	6p25.3	EXOC2	intronic	A/C	0.0868663	11.167	4.99E-06
TG	rs7773733	6p25.3	EXOC2	intronic	T/C	0.0868663	11.167	4.99E-06
TG	rs9504753	6p25.3	EXOC2	intronic	C/T	0.0868663	11.167	4.99E-06
TG	rs9502433	6p25.3	EXOC2	intronic	A/G	0.0868663	11.167	4.99E-06
TG	rs9504754	6p25.3	EXOC2	intronic	C/T	0.0868663	11.167	4.99E-06
TG	rs9504755	6p25.3	EXOC2	intronic	T/C	0.0868663	11.167	4.99E-06
TG	rs9504756	6p25.3	EXOC2	intronic	T/C	0.0868663	11.167	4.99E-06
TG	rs9504757	6p25.3	EXOC2	intronic	T/C	0.0868663	11.167	4.99E-06
TG	rs9504758	6p25.3	EXOC2	intronic	T/C	0.0868663	11.167	4.99E-06
TG	rs9504759	6p25.3	EXOC2	intronic	G/A	0.0868663	11.167	4.99E-06
TG	rs9504760	6p25.3	EXOC2	intronic	C/T	0.0868663	11.167	4.99E-06
TG	rs11752737	6p25.3	EXOC2	intronic	C/T	0.0729895	12.0628	4.87E-06
TG	rs9502435	6p25.3	EXOC2	intronic	T/C	0.0868663	11.167	4.99E-06
TG	rs9502436	6p25.3	EXOC2	intronic	T/C	0.086757	11.2165	4.57E-06
TG	rs13216502	6p25.3	EXOC2	intronic	T/C	0.0729895	12.0628	4.87E-06
TG	rs9502440	6p25.3	EXOC2	intronic	T/G	0.0868663	11.167	4.99E-06
TG	rs9504771	6p25.3	EXOC2	intronic	G/A	0.0868663	11.167	4.99E-06
TG	rs9328356	6p25.3	EXOC2	intronic	C/T	0.0868663	11.167	4.99E-06
TG	rs9504779	6p25.3	EXOC2	intronic	C/A	0.0868663	11.167	4.99E-06
TG	rs9504780	6p25.3	EXOC2	intronic	G/A	0.0868663	11.167	4.99E-06
TG	kgp4048230	6p22.2	CARMIL1	intronic	A/G	0.162065	-8.61217	3.75E-06
TG	rs1668689	6q15	MIR4464;MAP3K7	intergenic	T/C	0.0152972	27.5828	6.60E-07
TG	rs1753834	6q15	MIR4464;MAP3K7	intergenic	C/T	0.0152972	27.5828	6.60E-07
TG	rs2770660	6q15	MIR4464;MAP3K7	intergenic	A/G	0.0152972	27.5828	6.60E-07
TG	rs806273	6q15	MIR4464;MAP3K7	intergenic	A/G	0.0152972	27.5828	6.60E-07
TG	rs806275	6q15	MIR4464;MAP3K7	intergenic	T/G	0.0194493	26.684	4.88E-08
TG	rs806277	6q15	MIR4464;MAP3K7	intergenic	G/A	0.0187937	27.8687	2.05E-08
TG	rs116653477	7p22.3	FAM20C	intronic	G/A	0.0136582	27.5273	2.15E-06
TG	rs326	8p21.3	LPL	intronic	A/G	0.225464	-7.2776	7.15E-06
TG	rs331	8p21.3	LPL	intronic	G/A	0.222186	-7.37841	6.08E-06
TG	rs13702	8p21.3	LPL	UTR3	A/G	0.222453	-7.51633	3.93E-06
TG	rs15285	8p21.3	LPL	UTR3	G/A	0.222247	-7.42109	5.25E-06
TG	rs2083637	8p21.3	LPL;SLC18A1	intergenic	A/G	0.233064	-7.43978	3.46E-06
TG	rs1441756	8p21.3	LPL;SLC18A1	intergenic	A/C	0.231534	-7.2791	5.79E-06
TG	rs4424254	8p21.3	PEBP4	intronic	G/A	0.0194493	22.2703	5.82E-06
TG	rs4409399	8p21.3	PEBP4	intronic	C/T	0.0191215	22.4053	6.14E-06
TG	rs7823067	8p21.3	PEBP4	intronic	T/C	0.0197771	22.4339	4.25E-06
TG	rs56180498	8p21.3	PEBP4	intronic	A/C	0.0191215	22.4053	6.14E-06
TG	rs56235220	8p21.3	PEBP4	intronic	C/T	0.0191215	22.4053	6.14E-06
TG	rs55903357	8p21.3	PEBP4	intronic	C/T	0.0191215	22.4053	6.14E-06
TG	rs66865732	8p21.3	PEBP4	intronic	T/C	0.0192308	22.3789	5.94E-06
TG	rs34040712	8p21.3	PEBP4	intronic	A/G	0.0191215	22.4053	6.14E-06
TG	rs7823380	8p21.3	PEBP4	intronic	A/C	0.0197771	22.4339	4.25E-06
TG	rs36123552	8p21.3	PEBP4	intronic	G/A	0.0194493	22.2825	5.83E-06
TG	rs34834698	8p21.3	PEBP4	intronic	C/T	0.0194493	22.2825	5.83E-06
TG	rs71515804	8p21.3	PEBP4	intronic	A/C	0.01934	22.6067	4.51E-06
TG	rs34876649	8p21.3	PEBP4	intronic	C/T	0.01934	22.308	6.03E-06
TG	rs885327	8p21.3	PEBP4	intronic	G/T	0.0194493	22.2825	5.83E-06
TG	rs907888	8p21.3	PEBP4	intronic	T/C	0.0194493	22.2825	5.83E-06
TG	JHU_9.31968698	9p21.1	LINC01243;ACO1	intergenic	G/A	0.243934	7.25444	3.05E-06
TG	9:72558120	9q21.12	C9orf135;MAMDC2	intergenic	A/G	0.0649606	13.5241	8.30E-07
TG	rs185154492	10q23.33	PLCE1	intronic	T/G	0.0104895	30.076	6.24E-06
TG	rs12098339	10q23.33	PLCE1	intronic	A/G	0.0110358	29.5459	5.38E-06
TG	rs144896301	10q23.33	PLCE1	intronic	C/T	0.0110358	29.5459	5.38E-06
TG	rs139825096	10q23.33	PLCE1	intronic	C/A	0.0110358	29.5459	5.38E-06
TG	rs148218367	10q23.33	PLCE1	intronic	A/G	0.010708	31.2216	2.17E-06
TG	rs114010338	10q23.33	PLCE1	intronic	C/T	0.0110358	29.5459	5.38E-06
TG	rs61751499	10q23.33	PLCE1	exonic	C/T	0.0110358	29.5459	5.38E-06
TG	rs115282941	10q23.33	PLCE1	intronic	G/A	0.0110358	29.5459	5.38E-06
TG	rs115440842	10q23.33	PLCE1	intronic	G/A	0.0110358	29.5459	5.38E-06
TG	rs144369364	10q23.33	PLCE1	intronic	T/G	0.0110358	29.5459	5.38E-06
TG	rs114124771	10q23.33	PLCE1	intronic	G/A	0.0110358	29.5459	5.38E-06
TG	rs114775005	10q23.33	PLCE1	intronic	A/G	0.0110358	29.5459	5.38E-06
TG	rs7350481	11q23.3	LINC02702;BUD13	intergenic	G/A	0.247596	9.8501	1.99E-10
TG	rs1558861	11q23.3	LINC02702;BUD13	intergenic	A/G	0.225027	7.35632	4.92E-06
TG	rs74368849	11q23.3	BUD13	intronic	G/A	0.0393357	23.006	2.87E-11
TG	rs180326	11q23.3	BUD13	intronic	A/C	0.247268	7.42155	1.76E-06

TG	rs3825041	11q23.3	BUD13	intronic	G/A	0.221093	7.80222	1.54E-06
TG	rs10790162	11q23.3	BUD13	intronic	G/A	0.221154	7.90771	1.10E-06
TG	rs2160669	11q23.3	ZPR1	UTR3	A/G	0.222878	8.16704	4.75E-07
TG	rs964184	11q23.3	ZPR1	UTR3	G/C	0.231022	9.08692	1.25E-08
TG	rs75198898	11q23.3	ZPR1	intronic	G/A	0.0391171	24.2291	3.32E-12
TG	JHU_11.116650637	11q23.3	ZPR1	intronic	G/A	0.0387075	25.18	9.98E-13
TG	rs6589566	11q23.3	ZPR1	intronic	A/G	0.223316	8.02275	7.57E-07
TG	11:116656788-C-A	11q23.3	ZPR1	intronic	A/C	0.223376	7.89035	1.11E-06
TG	rs3741298	11q23.3	ZPR1	intronic	A/G	0.377022	6.40045	3.43E-06
TG	kgp12765201	11q23.3	ZPR1	intronic	G/A	0.0389071	24.8997	9.27E-13
TG	rs2266788	11q23.3	APOA5	UTR3	A/G	0.225175	8.15259	4.34E-07
TG	rs2075291	11q23.3	APOA5	exonic	C/A	0.0371747	26.0043	2.75E-13
TG	rs651821	11q23.3	APOA5	UTR5	A/G	0.264339	12.5977	2.25E-16
TG	rs662799	11q23.3	APOA5	upstream	A/G	0.264699	12.5291	2.97E-16
TG	rs1263177	11q23.3	APOA4	downstream	A/G	0.41408	6.83618	4.79E-07
TG	rs5104	11q23.3	APOA4	exonic	A/G	0.340074	7.48886	1.19E-07
TG	rs5092	11q23.3	APOA4	exonic	A/G	0.405491	6.87795	4.63E-07
TG	rs2542052	11q23.3	APOC3	upstream	C/A	0.463145	6.50892	1.20E-06
TG	rs2854117	11q23.3	APOC3	upstream	G/A	0.444151	6.19759	5.39E-06
TG	rs734104	11q23.3	APOC3	intronic	A/G	0.339418	6.85643	1.29E-06
TG	rs5128	11q23.3	APOC3	UTR3	G/C	0.330055	6.89233	1.32E-06
TG	kgp18765416	12q15	PTPRB	intronic	A/G	0.0488525	13.8181	8.80E-06
TG	rs147430337	13q14.11	VWA8	intronic	G/A	0.0323993	17.0809	8.01E-06
TG	rs9542941	13q21.33	DACH1;MZT1	intergenic	G/A	0.0282998	18.5393	5.55E-06
TG	rs74412765	14q13.1	EGLN3;SPTSSA	intergenic	G/A	0.0287432	18.0109	9.21E-06
TG	rs10135885	14q23.3	FUT8;CCDC196	intergenic	G/A	0.0124563	29.2887	1.85E-06
TG	rs74058666	14q23.3	FUT8;CCDC196	intergenic	C/T	0.0124563	29.2887	1.85E-06
TG	rs7155726	14q24.1	PLEK2;MIR5694	intergenic	G/A	0.0267603	21.3248	4.51E-07
TG	rs997912	14q31.3	LINC02301;SNORD3P3	intergenic	G/A	0.221967	7.37194	5.36E-06
TG	rs11638228	15q21.3	UNC13C	intronic	G/T	0.0469843	16.6915	1.76E-07
TG	rs17237494	15q21.3	UNC13C	intronic	A/G	0.0503282	15.6526	4.53E-07
TG	rs17818441	15q21.3	UNC13C	intronic	G/A	0.0512061	14.19	3.69E-06
TG	rs17237522	15q21.3	UNC13C	intronic	C/A	0.0511364	14.1913	3.92E-06
TG	rs17237564	15q21.3	UNC13C	intronic	A/G	0.0513549	14.5711	2.06E-06
TG	rs12902034	15q21.3	UNC13C	intronic	G/A	0.0508086	14.2432	4.23E-06
TG	rs12902248	15q21.3	UNC13C	intronic	C/T	0.0500437	14.3215	4.33E-06
TG	rs72732800	15q21.3	UNC13C	intronic	T/C	0.0513549	13.9614	5.93E-06
TG	rs11636575	15q21.3	UNC13C	intronic	T/C	0.0509178	14.0241	5.81E-06
TG	rs66602012	15q21.3	UNC13C	intronic	G/A	0.0499344	14.3561	4.18E-06
TG	rs10518765	15q21.3	UNC13C	intronic	A/C	0.0500437	14.2586	4.76E-06
TG	rs34817929	15q21.3	UNC13C	intronic	C/T	0.0499344	14.3561	4.18E-06
TG	rs35543750	15q21.3	UNC13C	intronic	G/A	0.0491696	14.9603	1.90E-06
TG	rs117773022	18q21.2	CCDC68;LINC01929	intergenic	G/A	0.0212953	21.9214	2.66E-06
TG	rs269909	19q13.42	NLRP2	intronic	G/C	0.0273284	18.9507	5.18E-06
TG	rs269910	19q13.42	NLRP2	intronic	T/G	0.0290647	18.5149	4.54E-06
TG	rs269912	19q13.42	NLRP2	exonic	G/A	0.029174	18.6086	3.92E-06
TG	rs269915	19q13.42	NLRP2	intronic	T/C	0.0290647	18.5149	4.54E-06
TG	rs34804158	19q13.42	NLRP2	exonic	A/G	0.0369318	16.013	7.10E-06
TG	rs11672240	19q13.42	NLRP2	intronic	C/A	0.0369318	16.013	7.10E-06
TG	rs11672325	19q13.42	NLRP2	intronic	G/A	0.0369318	16.013	7.10E-06
TG	rs35347198	19q13.42	NLRP2	intronic	T/G	0.0369318	16.013	7.10E-06
TG	rs1036231	19q13.42	NLRP2	intronic	A/G	0.0370411	15.9697	7.29E-06
TG	rs150809361	21q22.3	PCNT	intronic	A/G	0.0145419	27.2789	1.08E-06
TC	rs12206693	6q22.1	LINC02534;FRK	intergenic	G/A	0.0137645	13.2972	8.94E-06
TC	rs13214444	6q22.1	LINC02534;FRK	intergenic	C/T	0.0138737	13.2909	8.35E-06
TC	rs11967705	6q22.1	LINC02534;FRK	intergenic	A/G	0.0137645	13.372	7.95E-06
TC	rs7756213	6q22.1	LINC02534;FRK	intergenic	C/T	0.0138737	13.1995	9.61E-06
TC	rs4149268	9q31.1	ABCA1	intronic	G/A	0.362393	-3.35311	4.76E-06
TC	rs12686004	9q31.1	ABCA1	intronic	G/A	0.170278	-5.14216	1.89E-08
TC	rs2740494	9q31.1	ABCA1	intronic	C/G	0.36306	-3.31827	5.52E-06
TC	rs2244278	9q31.1	ABCA1	intronic	C/A	0.172384	-4.91152	6.66E-08
TC	rs1883025	9q31.1	ABCA1	intronic	G/A	0.254645	-4.93346	6.70E-10
TC	rs2575876	9q31.1	ABCA1	intronic	G/A	0.254645	-4.899	8.16E-10
TC	rs1800978	9q31.1	ABCA1	UTR5	C/G	0.185205	-5.61796	2.46E-10
TC	GSA-rs80077128	9q34.2	RXRA;COL5A1	intergenic	G/A	0.0576797	6.98405	2.79E-06
TC	rs651821	11q23.3	APOA5	UTR5	A/G	0.264281	3.88862	1.06E-06
TC	rs662799	11q23.3	APOA5	upstream	A/G	0.264642	3.9124	8.53E-07
TC	rs1263177	11q23.3	APOA4	downstream	A/G	0.413989	3.24289	3.95E-06
TC	rs5128	11q23.3	APOC3	UTR3	G/C	0.329873	3.26125	9.79E-06
TC	rs77850457	12p13.2	LOC440084	ncRNA_intro	T/G	0.0622679	-6.5014	7.04E-06

TC	rs1921320	12p13.2	LOC440084	ncRNA_intro	G/A	0.0633741	-6.42734	7.39E-06
TC	rs75780404	12p13.2	LOC440084	ncRNA_intro	A/G	0.0627048	-6.47691	7.20E-06
TC	rs11054301	12p13.2	LOC440084	ncRNA_intro	C/T	0.0628141	-6.39754	9.19E-06
TC	rs36086139	12p13.2	LOC440084	ncRNA_intro	T/C	0.0625956	-6.47866	7.27E-06
TC	rs146986976	14q21.2	LINC02303;LINC00871	intergenic	G/A	0.0375792	-8.28205	5.45E-06
TC	rs2043085	15q21.3	AQP9;LIPC	intergenic	G/A	0.470935	3.35908	1.70E-06
TC	rs1532085	15q21.3	AQP9;LIPC	intergenic	G/A	0.475093	3.44272	9.01E-07
TC	rs111604130	16p13.3	ADCY9	intronic	C/T	0.0172602	-12.9489	1.72E-06
TC	rs113111026	16p13.3	ADCY9	intronic	G/A	0.0173695	-12.5712	3.19E-06
TC	rs12933423	16p13.3	ADCY9	intronic	G/A	0.0225038	-11.5545	8.59E-07
TC	rs12917625	16p13.3	ADCY9	intronic	A/G	0.0225038	-11.5545	8.59E-07
TC	rs138192847	16q22.1	PDXDC2P-NPIPB14P	ncRNA_intro	A/G	0.0108244	15.9055	3.04E-06
TC	kgp16447600	16q22.1	AARS1	intronic	C/A	0.0105965	16.7671	1.08E-06
TC	rs1433099	19p13.2	LDLR	UTR3	G/A	0.26076	-3.56545	6.66E-06
TC	rs7258950	19p13.2	LDLR;SPC24	intergenic	G/A	0.203736	-4.46042	2.49E-07
TC	rs4804146	19p13.2	SPC24	UTR3	A/G	0.242954	-4.25586	1.50E-07
TC	19:45388130-G-A	19q13.32	NECTIN2	intronic	G/A	0.111208	6.10367	3.55E-08
TC	rs7254892	19q13.32	NECTIN2	intronic	G/A	0.0905615	-9.67176	3.59E-15
TC	rs6857	19q13.32	NECTIN2	UTR3	G/A	0.114521	6.63118	1.56E-09
TC	rs71352238	19q13.32	TOMM40	upstream	A/G	0.110031	6.13542	3.32E-08
TC	rs2075650	19q13.32	TOMM40	intronic	A/G	0.109679	6.19481	2.81E-08
TC	rs1160985	19q13.32	TOMM40	intronic	G/A	0.375985	-3.64048	5.21E-07
TC	rs769449	19q13.32	APOE	intronic	G/A	0.102906	6.98614	9.08E-10
TC	rs7412	19q13.32	APOE	exonic	G/A	0.0886805	-13.4045	4.79E-27
TC	19:45414399-C-A	19q13.32	APOE;APOC1	intergenic	C/A	0.106643	-10.0581	5.51E-19
TC	rs445925	19q13.32	APOE;APOC1	intergenic	G/A	0.110637	-9.61099	6.36E-18
TC	19:45426792-G-A	19q13.32	APOC1;APOC1P1	intergenic	G/A	0.125109	-7.83138	2.17E-13
HDL-C	rs75369757	1p36.13	IGSF21	intronic	G/T	0.0179353	5.75631	3.14E-06
HDL-C	rs78391202	1p36.13	IGSF21	intronic	C/T	0.0179353	5.75631	3.14E-06
HDL-C	rs147878115	2q31.1	DLX2-DT;ITGA6	intergenic	G/A	0.0446194	3.66439	4.25E-06
HDL-C	rs76235997	4q13.3	CXCL1;PF4	intergenic	C/T	0.0229659	4.93466	6.95E-06
HDL-C	rs2083637	8p21.3	LPL;SLC18A1	intergenic	A/G	0.23294	1.73856	7.52E-06
HDL-C	rs4149310	9q31.1	ABCA1	intronic	A/T	0.321554	-1.86219	8.16E-08
HDL-C	rs4743763	9q31.1	ABCA1	intronic	T/A	0.248025	-2.19592	4.24E-09
HDL-C	rs4149268	9q31.1	ABCA1	intronic	G/A	0.36279	-1.81745	1.11E-07
HDL-C	rs12686004	9q31.1	ABCA1	intronic	G/A	0.170574	-2.3037	7.38E-08
HDL-C	rs2740494	9q31.1	ABCA1	intronic	C/G	0.363457	-1.8196	9.84E-08
HDL-C	rs2244278	9q31.1	ABCA1	intronic	C/A	0.172682	-2.46404	6.93E-09
HDL-C	9:107662691-T-C	9q31.1	ABCA1	intronic	A/G	0.359799	-1.74283	3.30E-07
HDL-C	rs1883025	9q31.1	ABCA1	intronic	G/A	0.254814	-2.49018	2.70E-11
HDL-C	rs2575876	9q31.1	ABCA1	intronic	G/A	0.254923	-2.46605	3.92E-11
HDL-C	rs1800978	9q31.1	ABCA1	UTR5	C/G	0.185517	-2.50934	1.52E-09
HDL-C	rs4749830	10p14	LINC02676;LOC101928272	intergenic	A/G	0.042926	3.80252	3.13E-06
HDL-C	rs61840207	10p14	LINC02676;LOC101928272	intergenic	T/C	0.0430884	3.76768	3.62E-06
HDL-C	11:125945759	11q24.2	CDON;RPUSD4	intergenic	G/A	0.0253718	4.64955	8.21E-06
HDL-C	rs28564485	13q14.3	LINC01065;LINC00558	intergenic	T/C	0.0206693	5.35894	3.98E-06
HDL-C	rs1927859	13q14.3	LINC01065;LINC00558	intergenic	G/A	0.0206738	5.35709	4.01E-06
HDL-C	rs1927858	13q14.3	LINC01065;LINC00558	intergenic	C/A	0.0206693	5.35894	3.98E-06
HDL-C	rs6561730	13q14.3	LINC01065;LINC00558	intergenic	G/T	0.0206693	5.35894	3.98E-06
HDL-C	rs7337604	13q14.3	LINC01065;LINC00558	intergenic	G/T	0.0206693	5.35894	3.98E-06
HDL-C	rs2043085	15q21.3	AQP9;LIPC	intergenic	G/A	0.471232	1.60665	9.95E-07
HDL-C	rs1532085	15q21.3	AQP9;LIPC	intergenic	G/A	0.475394	1.65591	4.43E-07
HDL-C	rs8042174	15q21.3	AQP9;LIPC	intergenic	A/G	0.330672	-1.60484	3.75E-06
HDL-C	rs1077835	15q21.3	LIPC	upstream	A/G	0.42115	1.50406	4.71E-06
HDL-C	rs1077834	15q21.3	LIPC	upstream	A/G	0.420805	1.50894	4.39E-06
HDL-C	rs2070895	15q21.3	LIPC	upstream	G/A	0.419401	1.48595	6.21E-06
HDL-C	rs6494005	15q21.3	LIPC	intronic	A/G	0.205696	-2.02849	5.08E-07
HDL-C	rs8034802	15q21.3	LIPC	intronic	A/T	0.379895	-1.69235	3.94E-07
HDL-C	rs261334	15q21.3	LIPC	intronic	C/G	0.328481	1.67208	1.14E-06
HDL-C	rs588136	15q21.3	LIPC-AS1	ncRNA_intro	A/G	0.340097	1.59989	2.52E-06
HDL-C	16:56911640	16q13	SLC12A3	intronic	G/A	0.411739	-1.58609	1.41E-06
HDL-C	rs72786786	16q13	HERPUD1;CETP	intergenic	G/A	0.221675	2.45127	7.22E-10
HDL-C	rs173539	16q13	HERPUD1;CETP	intergenic	G/A	0.22221	2.66237	9.75E-12
HDL-C	rs247616	16q13	HERPUD1;CETP	intergenic	G/A	0.178298	3.7364	1.08E-18
HDL-C	rs247617	16q13	HERPUD1;CETP	intergenic	C/A	0.178954	3.74448	9.30E-19
HDL-C	rs183130	16q13	HERPUD1;CETP	intergenic	G/A	0.179262	3.76477	6.49E-19
HDL-C	rs3764261	16q13	HERPUD1;CETP	intergenic	C/A	0.178243	3.84448	1.37E-19
HDL-C	rs4783961	16q13	CETP	upstream	G/A	0.259571	2.46551	2.97E-11
HDL-C	rs1800775	16q13	CETP	upstream	C/A	0.450339	1.90939	4.81E-09
HDL-C	rs711752	16q13	CETP	intronic	G/A	0.369365	2.33756	5.78E-12

HDL-C	rs708272	16q13	CETP	intronic	G/A	0.369423	2.28869	1.46E-11
HDL-C	kgp1706776	16q13	CETP	intronic	G/A	0.139873	-2.10969	5.81E-06
HDL-C	rs9939224	16q13	CETP	intronic	C/A	0.138207	-2.31003	7.91E-07
HDL-C	rs1532624	16q13	CETP	intronic	C/A	0.314373	2.71406	1.63E-14
HDL-C	16:57006378-A-G	16q13	CETP	intronic	A/G	0.120735	-2.18606	9.94E-06
HDL-C	rs1800774	16q13	CETP	intronic	G/A	0.140123	-2.31258	9.44E-07
HDL-C	rs5882	16q13	CETP	exonic	A/G	0.421916	1.48211	8.36E-06
HDL-C	rs2303790	16q13	CETP	exonic	A/G	0.0202407	7.83221	1.42E-11
HDL-C	rs118153730	16q13	CETP;NLRC5	intergenic	G/A	0.0144357	7.51945	4.00E-08
HDL-C	rs7412	19q13.32	APOE	exonic	G/A	0.089007	3.22344	3.21E-08
HDL-C	19:45414399-C-A	19q13.32	APOE;APOC1	intergenic	C/A	0.10676	2.94061	2.85E-08
HDL-C	rs445925	19q13.32	APOE;APOC1	intergenic	G/A	0.110758	2.75771	1.27E-07
LDL-C	rs7578527	2p24.1	TDRD15;LINC01822	intergenic	G/A	0.0230011	-9.5474	5.28E-06
LDL-C	rs10169543	2p24.1	TDRD15;LINC01822	intergenic	T/C	0.0226725	-9.34284	9.70E-06
LDL-C	rs7599033	2q14.2	THORLNC;LOC101927709	intergenic	G/A	0.116893	4.33733	9.01E-06
LDL-C	rs80077128	9q34.2	RXRA;COL5A1	intergenic	G/A	0.0577218	5.98167	8.11E-06
LDL-C	rs569391082	11q22.1	TRPC6;ANGPTL5	intergenic	C/A	0.0106243	14.0902	4.73E-06
LDL-C	rs562710905	11q22.1	TRPC6;ANGPTL5	intergenic	T/C	0.0108434	13.4768	9.84E-06
LDL-C	rs543433741	11q22.1	TRPC6;ANGPTL5	intergenic	T/C	0.0108434	13.4768	9.84E-06
LDL-C	rs189902665	11q22.1	TRPC6;ANGPTL5	intergenic	C/T	0.0108434	13.4768	9.84E-06
LDL-C	rs191754438	11q22.1	TRPC6;ANGPTL5	intergenic	C/A	0.0108434	13.4768	9.84E-06
LDL-C	rs185863095	11q22.1	TRPC6;ANGPTL5	intergenic	G/A	0.0108434	13.4768	9.84E-06
LDL-C	rs534260525	11q22.1	TRPC6;ANGPTL5	intergenic	G/A	0.0108434	13.4768	9.84E-06
LDL-C	rs192817177	11q22.1	TRPC6;ANGPTL5	intergenic	C/T	0.0108434	13.4768	9.84E-06
LDL-C	rs190414287	11q22.1	TRPC6;ANGPTL5	intergenic	T/C	0.0108434	13.4768	9.84E-06
LDL-C	rs368811087	11q22.1	TRPC6;ANGPTL5	intergenic	G/A	0.0108434	13.4768	9.84E-06
LDL-C	rs185622959	11q22.1	TRPC6;ANGPTL5	intergenic	T/G	0.0108434	13.4768	9.84E-06
LDL-C	rs118170637	11q22.1	TRPC6;ANGPTL5	intergenic	G/A	0.0108434	13.4768	9.84E-06
LDL-C	rs192063723	11q22.1	TRPC6;ANGPTL5	intergenic	T/C	0.0108434	13.4768	9.84E-06
LDL-C	rs566900086	11q22.1	TRPC6;ANGPTL5	intergenic	G/T	0.0108434	13.4768	9.84E-06
LDL-C	rs532888756	11q22.1	TRPC6;ANGPTL5	intergenic	T/C	0.0108434	13.4768	9.84E-06
LDL-C	rs556098214	11q22.1	TRPC6;ANGPTL5	intergenic	T/G	0.0108434	13.4768	9.84E-06
LDL-C	rs190450540	11q22.1	TRPC6;ANGPTL5	intergenic	C/T	0.0108434	13.4768	9.84E-06
LDL-C	rs530311958	11q22.1	TRPC6;ANGPTL5	intergenic	G/A	0.0108434	13.4768	9.84E-06
LDL-C	rs182979830	11q22.1	TRPC6;ANGPTL5	intergenic	G/A	0.0108434	13.4768	9.84E-06
LDL-C	rs185075465	11q22.1	TRPC6;ANGPTL5	intergenic	C/A	0.0108434	13.4768	9.84E-06
LDL-C	rs111604130	16p13.3	ADCY9	intronic	C/T	0.0173056	-12.5348	2.53E-07
LDL-C	rs113111026	16p13.3	ADCY9	intronic	G/A	0.0174151	-12.1879	4.98E-07
LDL-C	rs12933423	16p13.3	ADCY9	intronic	G/A	0.022563	-11.3468	7.45E-08
LDL-C	rs12917625	16p13.3	ADCY9	intronic	A/G	0.022563	-11.3468	7.45E-08
LDL-C	rs138192847	16q22.1	PDXDC2P-NPIPB14P	ncRNA_intro	A/G	0.0108529	13.541	9.75E-06
LDL-C	rs117953939	16q22.1	AARS1	intronic	C/A	0.0106243	13.8496	7.37E-06
LDL-C	rs1433099	19p13.2	LDLR	UTR3	G/A	0.26057	-3.66872	2.55E-07
LDL-C	rs7258950	19p13.2	LDLR;SPC24	intergenic	G/A	0.203614	-4.25776	4.34E-08
LDL-C	rs4804146	19p13.2	SPC24	UTR3	A/G	0.242607	-4.21794	7.09E-09
LDL-C	rs395908	19q13.32	NECTIN2	intronic	G/A	0.16286	4.38621	1.99E-07
LDL-C	rs519113	19q13.32	NECTIN2	intronic	C/G	0.178626	3.99355	1.06E-06
LDL-C	rs387976	19q13.32	NECTIN2	intronic	C/A	0.375904	-3.83052	4.15E-09
LDL-C	rs369599	19q13.32	NECTIN2	intronic	A/G	0.376918	-3.94622	1.39E-09
LDL-C	rs34342646	19q13.32	NECTIN2	intronic	G/A	0.111172	6.53409	5.21E-11
LDL-C	rs7254892	19q13.32	NECTIN2	intronic	G/A	0.09069	-14.4766	1.12E-39
LDL-C	rs6857	19q13.32	NECTIN2	UTR3	G/A	0.114383	6.80899	5.16E-12
LDL-C	rs71352238	19q13.32	TOMM40	upstream	A/G	0.109991	6.69515	1.99E-11
LDL-C	rs2075650	19q13.32	TOMM40	intronic	A/G	0.109639	6.74151	1.77E-11
LDL-C	rs1160985	19q13.32	TOMM40	intronic	G/A	0.375878	-5.14113	2.96E-15
LDL-C	rs405509	19q13.32	APOE	upstream	A/C	0.38856	-3.97745	8.59E-10
LDL-C	rs769449	19q13.32	APOE	intronic	G/A	0.102957	7.75347	3.82E-14
LDL-C	rs7412	19q13.32	APOE	exonic	G/A	0.0886951	-18.3515	1.70E-61
LDL-C	rs75627662	19q13.32	APOE	downstream	G/A	0.210561	-4.5698	1.65E-09
LDL-C	rs72654473	19q13.32	APOE;APOC1	intergenic	C/A	0.106486	-14.3871	4.50E-46
LDL-C	rs445925	19q13.32	APOE;APOC1	intergenic	G/A	0.110489	-13.6955	4.94E-43
LDL-C	rs141622900	19q13.32	APOC1;APOC1P1	intergenic	G/A	0.125219	-10.8444	7.38E-30
LDL-C	rs77498029	19q13.43	ZNF446	intronic	G/A	0.0537788	6.36754	5.41E-06
LDL-C	rs73064302	19q13.43	ZNF446	intronic	G/A	0.0537788	6.28615	7.47E-06
LDL-C	rs73066211	19q13.43	ZNF446	intronic	C/T	0.0539978	6.25519	7.62E-06
LDL-C	rs11669464	19q13.43	ZNF446	intronic	C/T	0.0534502	6.47685	3.92E-06
LDL-C	rs11669526	19q13.43	ZNF446	intronic	G/A	0.0538883	6.43447	4.22E-06
LDL-C	rs11672609	19q13.43	ZNF446;SLC27A5	intergenic	C/T	0.0524644	6.31796	7.93E-06
LDL-C	rs11672614	19q13.43	ZNF446;SLC27A5	intergenic	C/T	0.0525739	6.36112	6.75E-06
LDL-C	rs11672730	19q13.43	ZNF446;SLC27A5	intergenic	G/A	0.0525739	6.36112	6.75E-06

LDL-C	rs55652736	19q13.43	ZNF446;SLC27A5	intergenic	G/T	0.0529025	6.23741	9.62E-06
LDL-C	rs11671092	19q13.43	SLC27A5	downstream	T/C	0.0529025	6.23741	9.62E-06
LDL-C	rs73066228	19q13.43	SLC27A5	intronic	A/G	0.0526835	6.23944	9.82E-06
FBS	rs373105180	1p35.2	COL16A1	intronic	C/T	0.0116022	8.3615	9.74E-07
FBS	rs10873707	1p22.3	DDAH1	intronic	A/C	0.147217	2.33982	2.47E-06
FBS	rs11580783	1p13.2	TSPAN2;NGF-AS1	intergenic	C/A	0.0397878	5.11881	4.43E-08
FBS	rs12727640	1p13.2	TSPAN2;NGF-AS1	intergenic	G/A	0.0390055	5.20165	3.50E-08
FBS	rs11581510	1p13.2	TSPAN2;NGF-AS1	intergenic	G/A	0.0390055	5.20165	3.50E-08
FBS	rs34857685	1p13.2	TSPAN2;NGF-AS1	intergenic	G/A	0.0390055	5.20165	3.50E-08
FBS	rs77969789	1p12	SPAG17	intronic	G/A	0.023351	6.11916	3.52E-07
FBS	rs76100211	1p12	SPAG17	intronic	T/C	0.0234254	6.08986	3.70E-07
FBS	rs144277211	1p12	SPAG17	intronic	C/T	0.0234254	6.08986	3.70E-07
FBS	rs112284550	1p12	SPAG17	intronic	C/T	0.0212155	6.81548	5.42E-08
FBS	1:165018767	1q23.3	PBX1;LMX1A	intergenic	G/A	0.0112807	7.79166	6.88E-06
FBS	exm217116	2q11.2	RFX8	exonic	G/A	0.0232507	5.76635	1.98E-06
FBS	rs149784455	2q14.1	DPP10;DDX18	intergenic	A/G	0.0163608	6.23852	9.59E-06
FBS	rs147031386	2q14.3	CNTNAP5;LINC01941	intergenic	C/A	0.0454144	4.04618	3.55E-06
FBS	rs141570158	2q14.3	CNTNAP5;LINC01941	intergenic	A/G	0.0443094	4.12532	3.01E-06
FBS	exm2261049	2q21.3	RAB3GAP1	exonic	G/A	0.018453	6.491	2.15E-06
FBS	rs11681248	2q21.3	DARS-AS1;CXCR4	intergenic	C/T	0.0121547	9.75284	5.94E-09
FBS	rs145271412	2q22.3	PABPC1P2;ACVR2A	intergenic	A/G	0.0127523	8.02725	7.32E-07
FBS	rs139823386	2q22.3	PABPC1P2;ACVR2A	intergenic	T/C	0.0128177	8.15269	4.21E-07
FBS	3:1798580	3p26.3	CNTN6;CNTN4	intergenic	A/G	0.0169098	8.57404	1.09E-09
FBS	rs116443983	3p24.3	LOC101927829;VENTXP7	intergenic	G/A	0.0113812	7.67385	8.76E-06
FBS	rs111606349	3p24.1	RBMS3	intronic	G/T	0.0187845	6.01109	8.52E-06
FBS	rs145473944	3p14.2	CADPS	intronic	T/G	0.0181215	6.27066	5.27E-06
FBS	rs17696120	3p14.2	CADPS	intronic	T/C	0.0181215	6.27066	5.27E-06
FBS	rs17696304	3p14.2	CADPS	intronic	T/C	0.0173481	6.62243	2.47E-06
FBS	rs17640193	3p14.2	CADPS	intronic	G/A	0.0174586	6.16128	9.72E-06
FBS	rs17640256	3p14.2	CADPS	intronic	T/C	0.0166851	6.76386	2.36E-06
FBS	rs9862010	3q13.33	CD86;CASR	intergenic	T/C	0.0107182	9.4705	5.95E-08
FBS	rs73858156	3q13.33	CASR	intronic	G/T	0.0106077	9.61343	4.36E-08
FBS	rs73858157	3q13.33	CASR	intronic	A/G	0.0107182	9.4705	5.95E-08
FBS	rs73858160	3q13.33	CASR	intronic	G/T	0.0107182	9.4705	5.95E-08
FBS	rs34765993	3q13.33	CASR	intronic	C/A	0.0107182	9.4705	5.95E-08
FBS	rs72967358	3q13.33	CASR	intronic	A/C	0.0107182	9.4705	5.95E-08
FBS	rs78822249	3q25.32	RSRC1	intronic	C/A	0.0262983	5.21325	5.56E-06
FBS	rs117139405	4p13	SLC30A9	intronic	G/A	0.020884	6.15864	1.32E-06
FBS	rs77671739	4q28.1	MIR2054;INTU	intergenic	C/T	0.0124862	7.43665	6.53E-06
FBS	5:31261865	5p13.3	CDH6	intronic	A/G	0.0182361	5.99557	9.92E-06
FBS	rs67932803	5p13.2	GDNF-AS1;LINC02110	intergenic	T/G	0.04	4.22393	6.10E-06
FBS	rs34277633	5p13.2	GDNF-AS1;LINC02110	intergenic	G/A	0.04	4.22393	6.10E-06
FBS	rs35843419	5p13.2	GDNF-AS1;LINC02110	intergenic	T/G	0.04	4.22393	6.10E-06
FBS	rs13187126	5p13.2	LINC02110	upstream	A/G	0.04	4.22393	6.10E-06
FBS	rs546049178	5p13.2	LINC02110	ncRNA_intro	C/T	0.04	4.22393	6.10E-06
FBS	rs17390031	5p13.2	LINC02110;LINC02107	intergenic	G/T	0.04	4.22393	6.10E-06
FBS	rs35689429	5p13.2	LINC02110;LINC02107	intergenic	T/C	0.04	4.22393	6.10E-06
FBS	rs17390108	5p13.2	LINC02110;LINC02107	intergenic	T/C	0.04	4.22393	6.10E-06
FBS	rs34463435	5p13.2	LINC02110;LINC02107	intergenic	A/G	0.04	4.22393	6.10E-06
FBS	rs2292261	5p13.2	LINC02110;LINC02107	intergenic	C/T	0.0401105	4.20455	6.56E-06
FBS	rs34340325	5p13.2	LINC02110;LINC02107	intergenic	A/G	0.0401105	4.20455	6.56E-06
FBS	rs72730663	5p13.2	LINC02110;LINC02107	intergenic	A/G	0.0407735	4.10576	8.74E-06
FBS	rs35260129	5p13.2	LINC02110;LINC02107	intergenic	T/C	0.0407735	4.14576	7.12E-06
FBS	rs35325156	5p13.2	LINC02110;LINC02107	intergenic	G/A	0.04	4.2092	6.57E-06
FBS	rs67066763	5p13.2	LINC02110;LINC02107	intergenic	C/T	0.04	4.2092	6.57E-06
FBS	kgp7965942	5q13.1	PIK3R1;LINC02198	intergenic	G/A	0.038011	4.72666	6.24E-07
FBS	rs185607651	5q32	ARHGEF37;PPARGC1B	intergenic	G/A	0.010723	10.0491	1.47E-08
FBS	rs185293650	5q34	TENM2	intronic	A/G	0.0272989	5.14354	4.60E-06
FBS	rs57777283	6p25.3	GMDS	intronic	G/T	0.0245304	5.88209	5.10E-07
FBS	rs59609802	6p25.3	GMDS	intronic	T/G	0.0246409	5.23286	9.26E-06
FBS	rs77302185	6p25.3	GMDS	intronic	T/G	0.0245304	5.3357	6.39E-06
FBS	rs77690599	6p25.3	GMDS	intronic	C/A	0.0245304	5.3357	6.39E-06
FBS	rs625781	6p25.3	GMDS	intronic	C/T	0.0253039	5.22169	7.47E-06
FBS	rs79409187	6p25.3	GMDS	intronic	C/T	0.0245304	5.3357	6.39E-06
FBS	rs11768344	7p21.2	LINC02587;CRPPA	intergenic	C/A	0.105967	2.7455	4.33E-06
FBS	rs10252766	7p15.3	LINC01162;SP4	intergenic	G/A	0.106329	2.7782	2.29E-06
FBS	rs116945428	8q23.3	CSMD3;TRPS1	intergenic	T/C	0.0144751	7.13787	3.43E-06
FBS	rs62529547	9p23	PTPRD	intronic	G/A	0.040442	4.21682	3.93E-06
FBS	rs62529548	9p23	PTPRD	intronic	A/C	0.040442	4.21682	3.93E-06
FBS	rs10511524	9p23	PTPRD	intronic	A/G	0.0409945	4.70807	3.18E-07

FBS	rs3904502	9p23	PTPRD	intronic	T/C	0.0474033	4.60183	9.46E-08
FBS	rs116951764	9q34.2	RNU6ATAC;LINC02247	intergenic	G/A	0.0282873	5.18424	1.96E-06
FBS	rs753934	10q22.1	ADAMTS14	intronic	G/T	0.0117127	7.68934	6.02E-06
FBS	rs375848759	10q25.1	SORCS3	intronic	G/A	0.0150276	6.69139	6.39E-06
FBS	rs148083093	10q25.1	SORCS3	intronic	T/C	0.0150276	6.69139	6.39E-06
FBS	rs187600588	10q25.1	SORCS3	intronic	T/G	0.0150276	6.69139	6.39E-06
FBS	rs182892918	12q13.2	PMEL	intronic	T/C	0.0117127	7.68326	6.23E-06
FBS	12:56355081	12q13.2	PMEL	intronic	A/G	0.0120469	7.44648	9.02E-06
FBS	rs138128600	13q22.1	KLF5;LINC00392	intergenic	A/C	0.0100552	10.4396	8.60E-09
FBS	15:46979646	15q21.1	LOC105370802;SEMA6D	intergenic	G/A	0.14538	2.25788	8.15E-06
FBS	rs80265779	15q22.31	ZNF609	intronic	A/G	0.06336	3.33309	9.41E-06
FBS	rs4777488	15q22.31	ZNF609	intronic	A/G	0.100376	3.13686	3.06E-07
FBS	kgp28360581	15q26.1	ST8SIA2	UTR3	A/G	0.0144751	6.85599	6.97E-06
FBS	rs7193144	16q12.2	FTO	intronic	A/G	0.251606	1.95711	4.27E-06
FBS	rs145166529	17q12	ASIC2	intronic	G/A	0.0107182	8.10351	3.99E-06
FBS	rs139937681	17q12	ASIC2	intronic	G/A	0.0116022	8.48327	4.13E-07
FBS	rs1724407	17q21.31	LINC02210-CRHR1	intronic	A/G	0.0155973	8.11069	2.08E-06
FBS	JHU_17.43749941	17q21.31	LINC02210-CRHR1	intronic	G/A	0.0158036	8.10928	1.80E-06
FBS	17:43750172	17q21.31	LINC02210-CRHR1	intronic	A/G	0.015701	7.84866	3.87E-06
FBS	rs1635291	17q21.31	LINC02210-CRHR1	intronic	A/G	0.0156975	8.02295	2.95E-06
FBS	17:43755857	17q21.31	LINC02210-CRHR1	intronic	A/C	0.0159116	7.79037	3.74E-06
FBS	rs1724400	17q21.31	LINC02210-CRHR1	intronic	C/A	0.0158186	7.85305	3.22E-06
FBS	rs1635288	17q21.31	LINC02210-CRHR1	intronic	G/A	0.0164371	7.35887	9.16E-06
FBS	rs57222984	17q21.31	LINC02210-CRHR1	intronic	A/G	0.0159116	7.54	7.60E-06
FBS	rs73317496	17q21.31	LINC02210-CRHR1	intronic	A/G	0.0112707	7.47892	8.92E-06
FBS	rs16941035	17q21.31	LINC02210-CRHR1	intronic	T/G	0.018011	7.41077	1.58E-06
FBS	rs16941038	17q21.31	LINC02210-CRHR1	intronic	T/C	0.0179006	7.35599	2.06E-06
FBS	17:43765881	17q21.31	LINC02210-CRHR1	intronic	A/G	0.018015	7.41093	1.58E-06
FBS	rs7215239	17q21.31	LINC02210-CRHR1	intronic	A/G	0.018232	7.31992	1.78E-06
FBS	rs112782432	17q21.31	LINC02210-CRHR1	intronic	C/T	0.0131492	6.89039	9.37E-06
FBS	rs78971836	17q21.31	LINC02210-CRHR1	intronic	G/A	0.0130387	7.02533	8.04E-06
FBS	JHU_17.43770711	17q21.31	LINC02210-CRHR1	intronic	G/A	0.0181215	7.5109	1.26E-06
FBS	rs74943545	18q21.2	DCC;LINC01919	intergenic	G/A	0.0181215	5.99412	7.08E-06
FBS	rs2067846	18q22.3	GTSCR1;LINC01541	intergenic	G/A	0.0767956	3.18605	3.59E-06
FBS	rs76462054	18q22.3	GTSCR1;LINC01541	intergenic	A/G	0.0767956	3.18605	3.59E-06
FBS	rs117786342	19q13.43	ZSCAN5A	intronic	A/G	0.0525967	3.69727	5.15E-06
FBS	rs6007430	22q13.31	PHF21B;NUP50-DT	intergenic	A/G	0.0459597	3.87587	9.35E-06
HbA1c	rs2611424	1p36.32	AJAP1;MIR4689	intergenic	G/A	0.0373717	0.19826	1.05E-06
HbA1c	kgp8104994	1p36.32	AJAP1;MIR4689	intergenic	C/A	0.0463875	0.169492	3.88E-06
HbA1c	rs1667056	2p25.3	LINC01250	ncRNA_exoni	A/G	0.0369458	0.180316	9.40E-06
HbA1c	rs823212	2p25.3	LINC01250	ncRNA_intro	C/T	0.0369458	0.180316	9.40E-06
HbA1c	rs823214	2p25.3	LINC01250	ncRNA_intro	A/C	0.0370484	0.189978	2.95E-06
HbA1c	rs823215	2p25.3	LINC01250	ncRNA_intro	A/G	0.0370484	0.189978	2.95E-06
HbA1c	rs1656330	2p25.3	LINC01250	ncRNA_intro	T/G	0.0405378	0.176357	5.98E-06
HbA1c	rs1667045	2p25.3	LINC01250	ncRNA_intro	G/A	0.0405378	0.176357	5.98E-06
HbA1c	rs1729888	2p25.3	LINC01250	ncRNA_intro	G/T	0.0424877	0.170648	6.29E-06
HbA1c	rs188911118	2q32.1	MIR548AE1;ZNF804A	intergenic	T/G	0.0186782	0.273048	1.40E-06
HbA1c	rs181907531	2q32.1	MIR548AE1;ZNF804A	intergenic	C/T	0.0186782	0.273048	1.40E-06
HbA1c	rs35993772	2q32.1	MIR548AE1;ZNF804A	intergenic	T/G	0.0508005	0.163703	3.14E-06
HbA1c	rs6747233	2q32.1	MIR548AE1;ZNF804A	intergenic	C/A	0.0508005	0.163703	3.14E-06
HbA1c	rs6721856	2q32.1	MIR548AE1;ZNF804A	intergenic	A/G	0.0508005	0.163703	3.14E-06
HbA1c	rs7584439	2q32.1	MIR548AE1;ZNF804A	intergenic	A/G	0.0508005	0.163703	3.14E-06
HbA1c	rs7558116	2q32.1	MIR548AE1;ZNF804A	intergenic	G/A	0.0508005	0.163703	3.14E-06
HbA1c	rs6434087	2q32.1	MIR548AE1;ZNF804A	intergenic	A/G	0.0508005	0.163703	3.14E-06
HbA1c	rs190492330	2q32.1	MIR548AE1;ZNF804A	intergenic	G/A	0.0188834	0.266094	2.31E-06
HbA1c	rs192419200	2q32.1	ZNF804A;LOC105373782	intergenic	T/C	0.0159072	0.310682	4.90E-07
HbA1c	3:1798580	3p26.3	CNTN6;CNTN4	intergenic	A/G	0.0178645	0.339361	4.47E-09
HbA1c	rs73125439	3p12.1	LINC02008;LINC00971	intergenic	T/C	0.0105706	0.357685	2.05E-06
HbA1c	rs73127212	3p12.1	LINC02008;LINC00971	intergenic	C/T	0.0105706	0.357685	2.05E-06
HbA1c	rs7659523	4q31.23	IQCM	intronic	G/A	0.220431	0.0863427	3.08E-06
HbA1c	rs185607651	5q32	ARHGEF37;PPARGC1B	intergenic	G/A	0.0106776	0.342814	4.74E-06
HbA1c	rs62441137	7p22.1	SLC29A4	intronic	G/A	0.0328702	0.192191	7.88E-06
HbA1c	rs62441138	7p22.1	SLC29A4	intronic	C/T	0.0326355	0.19321	7.95E-06
HbA1c	rs77366908	7p22.1	SLC29A4	intronic	C/T	0.034688	0.192688	4.66E-06
HbA1c	rs144497262	7q21.13	STEAP4;ZNF804B	intergenic	T/C	0.0126232	0.314088	5.46E-06
HbA1c	rs76896660	8q23.3	LINC01609	ncRNA_intro	C/T	0.0162151	0.286302	2.58E-06
HbA1c	rs76917556	8q23.3	LINC02237;CSMD3	intergenic	C/T	0.012931	0.304325	6.88E-06
HbA1c	rs74662396	8q23.3	LINC02237;CSMD3	intergenic	C/T	0.0132389	0.295826	9.82E-06
HbA1c	rs77219034	8q23.3	LINC02237;CSMD3	intergenic	C/A	0.0133415	0.298601	7.49E-06
HbA1c	kgp12312585	11p13	RCN1	intronic	A/C	0.0860016	0.125136	4.21E-06

HbA1c	rs191099352	11q23.3	FXYD6;TMPRSS13	intergenic	G/A	0.0124179	0.325658	2.35E-06
HbA1c	rs144466082	11q23.3	FXYD6;TMPRSS13	intergenic	C/T	0.0124179	0.325658	2.35E-06
HbA1c	rs185487064	11q23.3	FXYD6;TMPRSS13	intergenic	T/C	0.0124179	0.325658	2.35E-06
HbA1c	rs61963304	13q34	COL4A1	intronic	A/G	0.0276067	0.210591	7.72E-06
HbA1c	rs61963305	13q34	COL4A1	intronic	A/C	0.0276067	0.210591	7.72E-06
HbA1c	rs61963306	13q34	COL4A1	intronic	C/T	0.0276067	0.210591	7.72E-06
HbA1c	rs61963307	13q34	COL4A1	intronic	G/T	0.0276067	0.210591	7.72E-06
HbA1c	rs7193144	16q12.2	FTO	intronic	A/G	0.253294	0.0840762	2.52E-06
HbA1c	rs8050136	16q12.2	FTO	intronic	C/A	0.241995	0.0802745	9.75E-06
HbA1c	rs3751812	16q12.2	FTO	intronic	C/A	0.242194	0.0816465	7.08E-06
HbA1c	rs9936385	16q12.2	FTO	intronic	A/G	0.243584	0.0805088	8.99E-06
HbA1c	rs11075989	16q12.2	FTO	intronic	G/A	0.243429	0.0803132	9.47E-06
HbA1c	rs11075990	16q12.2	FTO	intronic	A/G	0.243584	0.0802475	9.61E-06
HbA1c	rs7185735	16q12.2	FTO	intronic	A/G	0.243732	0.080761	8.38E-06
HbA1c	rs17817964	16q12.2	FTO	intronic	G/A	0.250514	0.0849552	2.18E-06
HbA1c	rs35541683	18q21.2	LINC01630;DCC	intergenic	A/G	0.0191992	0.268501	1.16E-06
HbA1c	rs185255157	18q23	ATP9B	intronic	G/A	0.0100595	0.346511	6.12E-06
HbA1c	rs78760057	19q13.11	LINC01782;ZNF507	intergenic	C/T	0.0385878	0.185289	3.74E-06
HbA1c	rs79875889	19q13.11	LINC01782;ZNF507	intergenic	G/A	0.0397167	0.185371	2.60E-06
HbA1c	rs76824054	19q13.11	LINC01782;ZNF507	intergenic	G/A	0.0385878	0.185289	3.74E-06
HbA1c	rs76418736	19q13.11	LINC01782;ZNF507	intergenic	G/A	0.03828	0.188285	2.83E-06
HbA1c	rs78742072	19q13.11	LINC01782;ZNF507	intergenic	C/T	0.039101	0.184215	3.75E-06
HbA1c	rs75310087	19q13.11	LINC01782;ZNF507	intergenic	C/T	0.0389984	0.189924	1.79E-06
HbA1c	rs79864354	19q13.11	LINC01782;ZNF507	intergenic	T/C	0.0418719	0.183512	1.89E-06
HbA1c	kgp2681083	20q13.33	NTSR1	intronic	A/G	0.432252	-0.0705215	4.81E-06
HbA1c	rs5997304	22q11.21	LRRC74B;BCRP2	intergenic	C/T	0.0105706	0.388743	2.36E-07

Table S13 SNP matching summary of the best-performing PRSs

Trait	PGS ID	Total SNPs	Matched (no flip)	Matched (strand flip)	Excluded (ambiguous)	Unmatched	% Retained
T2D	PGS004106	35	15	9	0	11	68.57
CVD	PGS000059	46	13	14	4	15	58.7
TG	PGS000312	190	72	64	9	45	71.58
TC	PGS000311	234	86	75	12	61	68.8
HDL-C	PGS000309	247	92	75	14	66	67.61
LDL-C	PGS000310	194	67	66	11	50	68.56
FBS	PGS000305	31	9	8	2	12	54.84
HbA1c	PGS000131	19	9	7	1	2	84.21

Table S14 Correlation between PRS performance and SNP retention

Trait	Retention_Ty	Pearson_r	R_squared	p_value	n
CVD	simple	-0.335	0.1123	0.314	11
CVD	weighted	-0.2482	0.0616	0.462	11
FBS	simple	NA	NA	NA	2
FBS	weighted	NA	NA	NA	2
HDL	simple	-0.7044	0.4962	0.184	5
HDL	weighted	-0.6391	0.4084	0.246	5
HbA1c	simple	0.7608	0.5789	0.239	4
HbA1c	weighted	0.8509	0.7241	0.149	4
LDL	simple	-0.0775	0.006	0.811	12
LDL	weighted	-0.5281	0.2789	0.0776	12
T2D	simple	-0.2273	0.0516	0.335	20
T2D	weighted	0.0416	0.0017	0.862	20
TC	simple	-0.1793	0.0322	0.821	4
TC	weighted	0.4801	0.2305	0.52	4
TG	simple	-0.6904	0.4766	0.129	6
TG	weighted	-0.0235	0.0006	0.965	6
All traits	simple	0.087	0.007	0.496	64
All traits	weighted	0.088	0.008	0.491	64

Table S15 Performance of PRSs across traits by discovery GWAS ancestry

Trait	Discovery an	Number	Mean perforr	SD	P value
T2D	East Asian	1	0.6970692	NA	0.25408967
T2D	European	12	0.69886808	8.38E-04	0.25408967
T2D	Multiancestr	7	0.6990761	0.00162429	0.25408967
CVD	European	1	0.8067741	NA	0.20487092
CVD	Multiancestr	10	0.80228898	0.00379167	0.20487092
TG	East Asian	1	0.03326808	NA	0.53845701
TG	European	3	0.02636678	0.01047922	0.53845701
TG	Multiancestr	2	0.0201565	0.00347826	0.53845701
TC	East Asian	1	0.07007298	NA	0.40656966
TC	European	2	0.05735432	0.02867649	0.40656966
TC	Multiancestr	1	5.94E-04	NA	0.40656966
HDL-C	East Asian	2	0.04759386	0.0059489	0.30119421
HDL-C	European	1	0.05329971	NA	0.30119421
HDL-C	Multiancestr	2	0.04335297	0.00287225	0.30119421
LDL-C	East Asian	1	0.03238113	NA	0.28709181
LDL-C	European	8	0.03724346	0.03024453	0.28709181
LDL-C	Multiancestr	3	0.05357155	0.02851148	0.28709181
FBS	European	2	0.00715195	0.00311349	NA
HbA1c	African	1	0.02229605	NA	0.39162518
HbA1c	East asian	1	0.02027866	NA	0.39162518
HbA1c	European	1	0.01687882	NA	0.39162518
HbA1c	Multiancestr	1	0.01457626	NA	0.39162518
